# Dinuclear Copper Complex with a Pyridazine‐Bridged Octadentate Ligand: Monooxygenase Activity and Characterization of Copper‐Oxygen Intermediates

**DOI:** 10.1002/chem.202501659

**Published:** 2025-06-03

**Authors:** Alexander Stüber, Ramona Jurgeleit, Benjamin Grimm‐Lebsanft, Sören Buchenau, Ina Kellner, Yannik Appiarius, Christian Näther, Jan Krahmer, Ivana Ivanović‐Burmazović, Michael Rübhausen, Maria A. Naumova, Felix Tuczek

**Affiliations:** ^1^ Institute of Inorganic Chemistry Christian‐Albrechts‐University of Kiel Max‐Eyth‐Straße 2 24118 Kiel Germany; ^2^ Institut für Nanostruktur‐ und Festkörperphysik, Center for Free Electron Laser Science (CFEL) Universität Hamburg Luruper Chaussee 149 22761 Hamburg Germany; ^3^ Department Chemie Ludwig‐Maximilians‐Universität München Butenandtstrasse 5–13, Haus D 81377 München Germany; ^4^ Institute for Organic and Analytical Chemistry University of Bremen Leobener Strasse 7 28359 Bremen Germany; ^5^ MAPEX Center for Materials and Processes University of Bremen Bibliothekstraße 1 28359 Bremen Germany; ^6^ DESY, Deutsches Elektronen‐Synchrotron (DESY) Notkestrasse 85 22607 Hamburg Germany

**Keywords:** catalysis, copper, EXAFS spectroscopy, metalloenzymes, oxygenation

## Abstract

Copper‐containing enzymes catalyze the mono‐oxygenation of aromatic and aliphatic substrates in nature. Herein, we report on the synthesis of a new dinuclear copper complex supported by an octadentate ligand with a pyridazine backbone. Low‐temperature oxygenation leads to a *μ*‐1,1‐hydroperoxo dicopper(II) (**Cu_2_OOH**) complex, which in turn stoichiometrically converts anthrone (AT) to anthraquinone (AQ). Oxygenation at room temperature, by contrast, leads to a new species that mediates the conversion of AT to AQ in a catalytic fashion, but neither corresponds to a *μ*‐peroxo nor a mono *μ*‐oxo dicopper complex. For further analysis, a *μ*‐hydroxo dicopper(II) (**Cu_2_OH**) complex is synthesized by oxidation of the copper(I) complex with AgOTf. The electronic and geometric structures of the **Cu_2_OOH** and **Cu_2_OH** intermediates, as well as the structure of the room‐temperature oxygenation product are elucidated by UV/Vis, Raman, X‐ray absorption spectroscopy (XAS), and mass spectrometry, coupled to DFT.

## Graphical abstract

Access to various copper‐oxygen intermediates: Depending on the reaction conditions, different copper‐oxygen intermediates can be obtained with our new dinuclear complex. Whereas low‐temperature oxygenation leads to a *μ*‐1,1 hydroperoxo complex, oxygenation at room temperature generates a “green species” that looks like a mono *μ*‐oxo complex but rather corresponds to a dihydroxo complex. The reactivities of all of these intermediates as well as the *μ*‐hydroxo complex toward hydrocarbons are investigated.

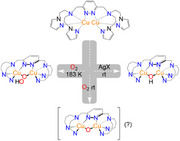

## 1 Introduction

The transformation of inert C─H bonds to useful products is one of the biggest challenges of chemistry.^[^ ^1^ ^]^ In this regard, copper‐dependent monooxygenases play an important role, as exemplified by the enzyme particulate methane monooxygenase (pMMO) that converts methane to methanol in methanotrophic bacteria.^[^ ^2^ ^]^ However, the identity of the active site in pMMO is still subject to controversy, and details of the reaction mechanism have not fully been elucidated yet.^[^ ^2^, ^3^ ^]^ On the other hand, mono‐*μ*‐oxo dicopper(II) (**Cu_2_O**) species have been found to perform this challenging conversion in the inorganic solid‐state system Cu‐ZSM‐5.^[^ ^4^, ^5^ ^]^ This structural motif, therefore, is currently considered as one of the important copper‐oxygen species besides the common **Cu_2_O_x_** cores.^[^ ^5^, ^6^, ^7^, ^8^, ^9^, ^10^, ^11^ ^]^ However, only a few small‐molecule **Cu_2_O** model complexes have been established to date,^[^ ^6^, ^11^, ^12^, ^13^ ^]^ and a comprehensive understanding of how the ligand sphere influences their electronic and reactive properties is lacking.^[^ ^6^, ^7^, ^11^, ^12^, ^13^, ^14^, ^15^ ^]^

Recently we reported on dicopper(I) complexes supported by hexadentate ligands with a pyridazine backbone.^[^ ^11^, ^13^ ^]^ Notably, these systems were found to exhibit catalytic monooxygenation activity, transferring both oxygen atoms of O_2_ to aliphatic substrates without the need of additional reductant. Using a combination of UV/Vis, resonance‐Raman (rR), and X‐ray absorption spectroscopy (XAS) complemented by UHR‐ESI mass spectrometry, we were able to show that dioxygen initially is bound as *μ*_4_‐peroxide in a tetranuclear Cu(I)_2_Cu(II)_2_ cluster. Subsequent O─O bond cleavage leads to two mono‐*μ*‐oxo dicopper(II) complexes, which in turn oxygenate a variety of hydrocarbons with bond dissociation enthalpies (BDEs) below 82 kcal mol^−1^.^[^ ^11^ ^]^ However, these **Cu_2_O** intermediates exhibit a low thermal stability (up to ‐35°C), limiting the temperature range in which monooxygenation reactions can be conducted and, thus, their substrate scope.^[^ ^11^ ^]^ Hence, we wanted to modify the ligand sphere in order to make the **Cu_2_O** species thermally more robust.

**Scheme 1:**
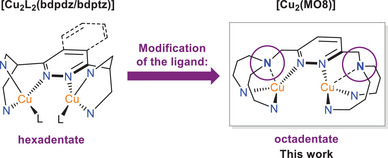
**Ligand design of MO8**. The pyridine moieties of the (pyridazine‐based) **bdpdz** and (phthalazine‐based) **bdptz** systems (left) and the pyrazole rings of the **MO8** system (right) have been omitted for clarity and simplified by the *N*‐donor atoms.

On this background, we synthesized a modified dinucleating ligand in which the two bidentate arms attached to the central pyridazine moiety of the original ligand are replaced by two tridentate NNN units, bound to the pyridazine via their central amines (cf Scheme 1). This modification of the parent system,^[^ ^11^ ^]^ which was based on the literature‐known hexadentate ligands **bdpdz** ^[^ ^16^ ^]^ and **bdptz,** ^[^ ^17^ ^]^ leads to the octadentate ligand **MO8** (Scheme 1). In contrast to the terminal pyridine donors of the related octadentate, pyridazine‐bridged ligand **BPMPD**,^[^ ^18^ ^]^ **MO8** is furnished with terminal pyrazole groups. We thought that the reduced σ‐donor strength of pyrazole compared to pyridine may be beneficial for a more electrophilic and thus more reactive **Cu_2_O** unit.^[^ ^19^ ^]^ Moreover, we expected that, due to its higher flexibility compared to **bdpdz/bdptz**, it might enable access to other copper‐oxygen (**Cu_2_O_x_**) intermediates. Finally, we assumed that due to the two additional *N*‐donor atoms, coordination of solvent ligands (as observed for the original system)^[^ ^11^ ^]^ might be reduced, thus enabling easier access of substrate to the **Cu_2_O** moiety.

Having prepared **MO8**, we found that oxygenation of its dicopper(I) complex strongly depends on the temperature: upon reaction with O_2_ at 183 K, a *μ*‐1,1‐hydroperoxo dicopper(II) complex (**Cu_2_OOH**) is formed that decomposes at higher temperatures. By contrast, reaction with O_2_ at room temperature leads to a new species that was first interpreted as a mono‐*μ*‐oxo dicopper(II) (**Cu_2_O**) intermediate, based on its UV/Vis spectrum. Herein, the **Cu_2_OOH** and **Cu_2_OH** derivatives of the Cu_2_(**MO8**) system as well as the room‐temperature oxygenation product are structurally and/or spectroscopically characterized, and their monooxygenation activities toward various aliphatic substrates are examined. The results are compared with analogous data obtained on the original hexadentate **bdpdz/bdptz** system, and the electronic and structural factors determining the formation and stabilities of the copper‐oxygen intermediates accessible in the **MO8** system as well as their respective reactivities are defined.

## 2 Results and Discussion

### 2.1 Synthesis and Structural Characterization

#### 2.1.1 Synthesis of the Ligand 1 and the Dicopper(I) Complex 2

**Scheme 2:**
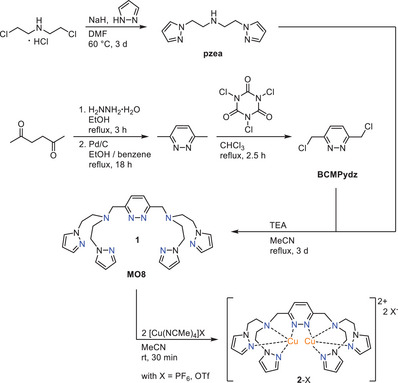
Synthesis of the new octadentate *N*‐donor ligand **MO8 1** and formation of the corresponding dicopper(I) complexes **2**‐PF_6_ and **2**‐OTf.

The new multidentate *N*‐donor ligand 3,6‐bis(di(2‐(1*H*‐pyrazol‐1‐yl)ethyl)aminomethyl)pyridazine (**1**), denoted as **MO8,** was synthesized in a five‐step procedure following the route shown in Scheme 2. The easily accessible *N*,*N*‐bis((1*H*‐pyrazol‐1‐yl)methyl)propane‐1‐amine (**pzea**)^[^ ^20^ ^]^ exhibits a central amine function, which is used to connect the two tridentate units to the 3,6‐positions of a pyridazine spacer using 3,6‐bis(chloromethyl)‐pyridazine (**BCMPydz**)^[^ ^21^ ^]^ (Scheme 2).

Addition of two equivalents of [Cu(NCMe)_4_]PF_6_ or [Cu(NCMe)_4_]OTf to the ligand in acetonitrile provided the complexes [Cu^I^_2_(**MO8**)](PF_6_)_2_ (**2**‐PF_6_) and [Cu^I^_2_(**MO8**)](OTf)_2_ (**2**‐OTf) in almost quantitative yields (Scheme 2 and Section S2.3).

#### 2.1.2 Crystal Structure of 2‐PF_6_

**Figure 1:**
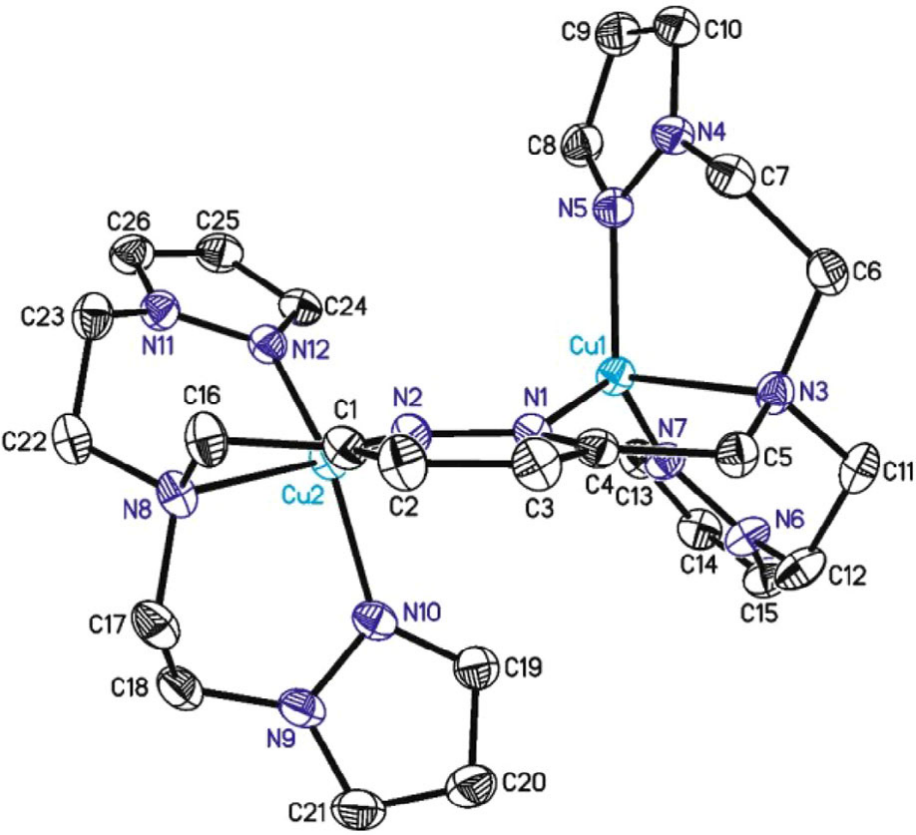
**Crystal structure of the dicopper(I) complex** [Cu^I^_2_(**MO8**)](PF_6_)_2_ (**2**‐PF_6_). ORTEP plot of the cation in the crystal structure of compound **2**‐PF_6_ with labeling and displacement ellipsoids drawn at the 50 % probability level. For clarity, the two crystallographically independent hexafluorophosphate anions and the hydrogen atoms are not shown.

Layering a solution of **2**‐PF_6_ in acetone with diethyl ether yielded red crystals, which were investigated by single‐crystal X‐ray diffraction analysis.^[^ ^22^ ^]^ The obtained molecular structure reveals the expected tetracoordinated geometry for the copper(I) centers (see below, Figure 1, Section S3). Importantly, it does not show any additional solvent coligands, in contrast to the original **bdpdz/bdptz** system. This may also apply to the corresponding copper‐oxygen intermediates (vide infra), potentially allowing easier substrate access to the active site than in the parent system.

The coordination sphere of each copper center is distorted tetrahedral (Figure S21) and consists of four *N*‐donor atoms, two of which derive from the pyrazole moieties and two from the amine and the pyridazine group, respectively (Figure 1). **2**‐PF_6_ crystallizes in the monoclinic space group P2_1_/n with four units per unit cell. The average N‐Cu‐N angle is 106.38°, and the average Cu‐N bond length is 2.0420 Å, ranging from 1.9424 to 2.2229 Å.^[^ ^22^ ^]^

#### 2.1.3 DFT Calculations

**Figure 2:**
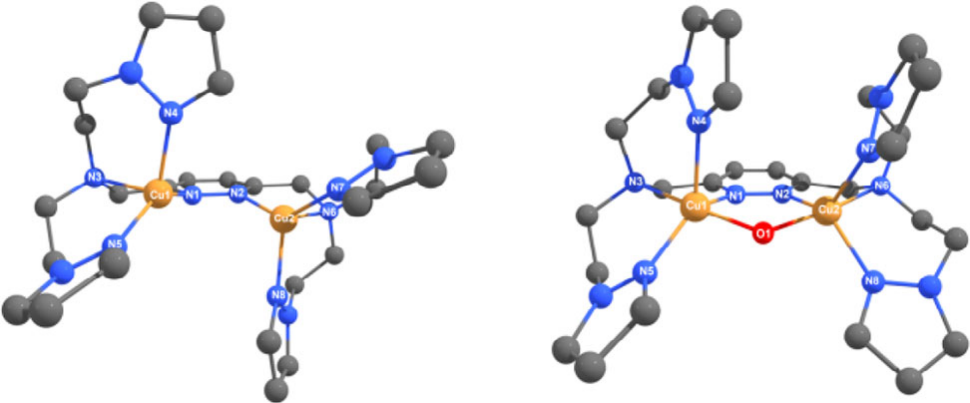
**Geometry optimization of 2‐PF_6_ (left) and the corresponding Cu_2_O species 4 (right)**. The hydrogen atoms have been omitted for clarity. DFT: RI‐PBE‐D3(BJ)/def2‐SVP.^[^ ^23^, ^24^ ^].^

Geometry optimization of **2**‐PF_6_ leads to a structure with Cu─Cu and Cu─N bond lengths comparable to the crystal structure of **2**‐PF_6_ (see Figure 2, left, Table S14). Geometry optimization of the **Cu_2_O** complex **4** of **2**‐PF_6_, on the other hand, indicates that a dicopper mono‐*μ‐*oxo core can be accommodated by the octadentate system of **MO8** (see Figure 2, right). Interestingly, the bond lengths of the Cu─N_Amine_ bond are about 0.2–0.3 Å longer than those of the other Cu─N bonds, suggesting a hemi‐labile character of this bond (see Table S11).

#### 2.1.4 Generation and Properties of the *μ*‐Hydroxo Dicopper(II) Complex 3

**Scheme 3:**
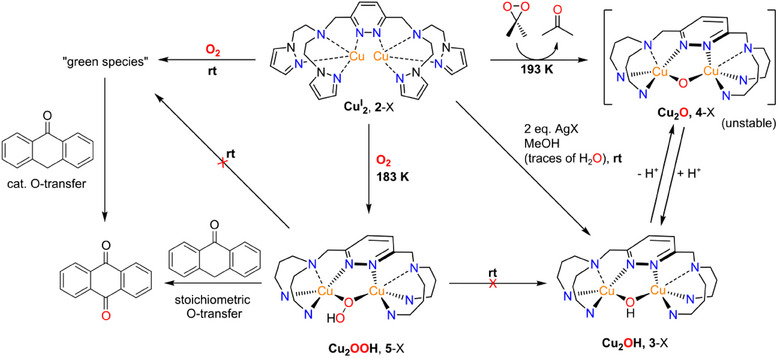
Formation of different copper‐oxygen intermediates of the new **MO8** model system and associated reactivities. The counterions X are hexafluorophosphate (PF_6_) or triflate (OTf). The charges of all complexes shown have been omitted for clarity, and the pyrazole units in **3**‐X, **4**‐X, and **5**‐X have been simplified by the *N*‐donor atoms.

To gain further structural insight into our system, we prepared the *μ*‐hydroxo dicopper(II) complexes [Cu^II^_2_(**MO8**) (OH)]X_3_ (**3‐X,** X = OTf or PF_6_; **Cu_2_OH**) by reaction of **2**‐OTf with AgOTf (or **2**‐PF_6_ with AgPF_6_, respectively), in methanol, analogous to the procedure of Kuzelka et al.^[^ ^25^ ^]^ (cf Scheme 3). After the addition of 2 eq. of AgX to the respective Cu(I) complex **2**‐X, the solution turned greenish within a few seconds.^[^ ^25^ ^]^ The resulting complexes were characterized by x‐ray powder diffraction, UV/Vis‐, NMR‐, and IR‐spectroscopy, as well as HR‐ESI mass spectrometry and XAS. It should be noted that no additional source of oxygen was added so that the hydroxido ligand probably originates from traces of water being present in the solvent, as assumed by Kuzelka. The remaining proton then presumably forms HOTf (or HPF_6_), which is eliminated upon drying in vacuo.

#### 2.1.5 Crystal Structure of 3‐PF_6_

**Figure 3:**
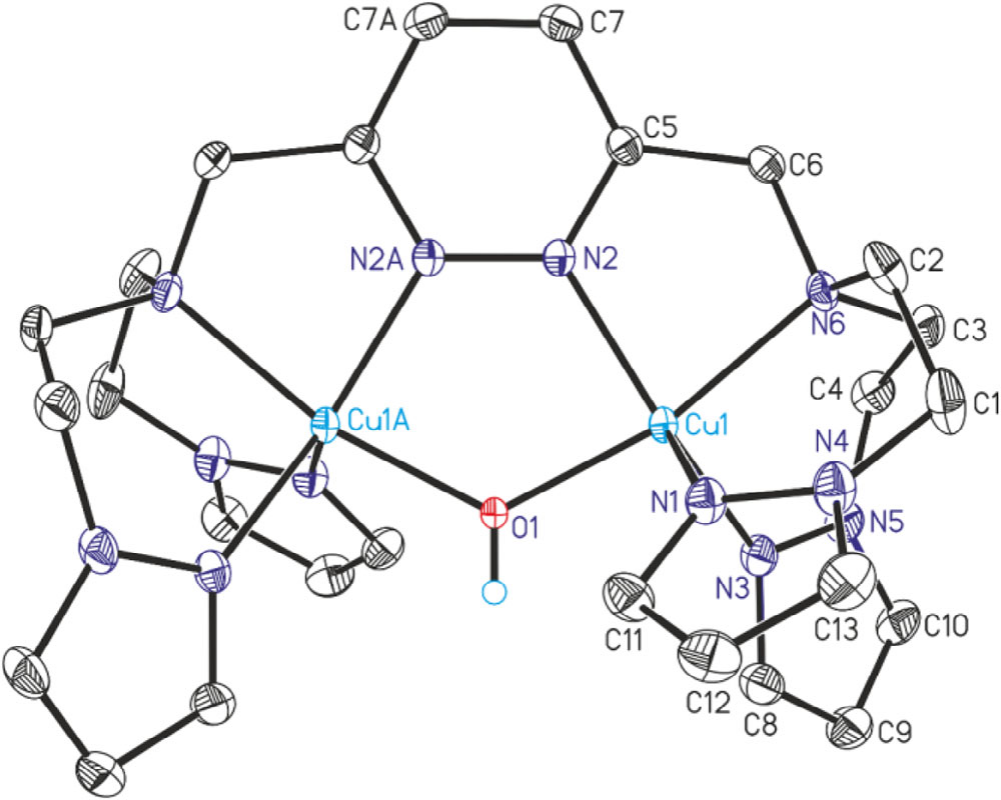
**Crystal structure of the Cu_2_OH complex** [Cu_2_(**MO8**) (OH)](PF_6_)_3_ (**3**‐PF_6_). ORTEP plot of the cation in the crystal structure of compound **3**‐PF_6_ with labeling and displacement ellipsoids drawn at the 50 % probability level. For clarity the three crystallographically independent hexafluorophosphate anions and the hydrogen atoms (except from the OH group) are not shown.

Turquoise‐colored crystals were obtained by layering a solution of **3**‐PF_6_ in acetone with *n*‐hexane, which were investigated by single‐crystal X‐ray diffraction analysis. The crystal structure shows that each copper is pentacoordinated in **3**‐PF_6_ and that the two copper centers are bridged via a hydroxido ligand (Figure 3, see below). As in the Cu(I)‐complex **2**‐PF_6_, no additional solvent coligands coordinate to copper.

**3**‐PF_6_ crystallizes in the orthorhombic space group Pbcn with four units per unit cell. Both copper centers in **3**‐PF_6_ exhibit a distorted square‐pyramidal geometry (Figure S22). The average N‐Cu‐N angle is 104.70° and the average Cu‐N bond length is 2.0522 Å, ranging from 1.9843 to 2.1296 Å. The Cu‐O‐Cu angle is 124.74° and the Cu‐O bond length 1.9301 Å.^[^ ^22^ ^]^ Bulk **3**‐PF_6_ is crystalline, exhibiting a powder diffractogram that matches the corresponding crystal structure (Figure S23), whereas **3**‐OTf is found to be amorphous (Figure S24).

#### 2.1.6 Spectroscopic Characterization of 3

**Figure 4:**
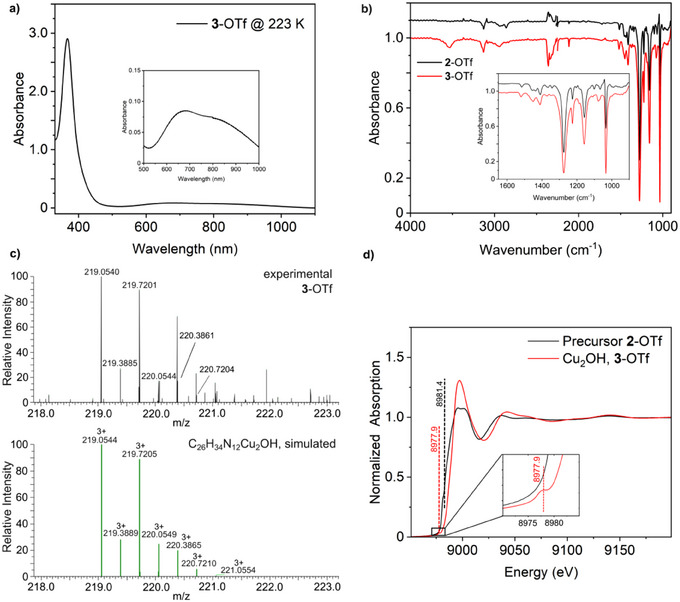
**Characterization of the *μ*‐hydroxo dicopper(II) complex 3**. **a)** Absorption spectra of an acetone solution of **3**‐OTf at 223 K. **b)** IR spectra of **2**‐OTf (black) and **3**‐OTf (red). **c)** Characteristic cutout of the HR‐ESI mass spectrum of **3**‐OTf at room temperature, indicating the formation of the **Cu_2_OH** complex. **d)** Cu K‐edge XANES spectra of the precursor **2**‐OTf (black) and the **Cu_2_OH** complex **3**‐OTf (red) at 293 K with enlarged pre‐edge region. General remarks: UV/Vis: l  =  1 cm.

The solution of the **Cu_2_OH** complex **3**‐OTf in acetone exhibits three absorption bands in the UV/Vis spectrum at 367 nm (*ε* = 5800 m ^−1^cm^−1^), 680 nm (*ε* = 170 m ^−1^cm^−1^), and 860 nm (*ε* = 120 m ^−1^cm^−1^) (Figure 4a). The spectrum of **3**‐PF_6_ is very similar to that of **3**‐OTf. However, this compound has a significantly lower solubility than **3**‐OTf (Figure S26).

Paramagnetic broadening of the signals in the ^1^H‐NMR spectrum of **3**‐OTf and **3**‐PF_6_ (Figure S15,S17) indicates the presence of Cu(II). Compared to the Cu(I) complex **2,** some signals have shifted in the ^1^H‐NMR‐spectrum of **3**. Apart from small shifts of some signals, the ^1^H‐NMR spectra of **3**‐OTf and **3**‐PF_6_ are very similar. In the ^13^C‐NMR spectrum of **3** (Figure S16, S18), no signals are visible due to the paramagnetic broadening, which makes it difficult to assign the signals of the ^1^H‐NMR spectrum.

From the solution of **3‐**OTf in acetonitrile‐d3 used for NMR spectroscopy, an IR spectrum was recorded as well. To ensure that the sample was not affected by oxygen or humidity from the air, a sealed cell was used, which was prepared under an inert atmosphere in the glovebox. The IR spectra of the **3**‐OTf and **2**‐OTf are very similar (Figure 4b/ Section S7.2). However, there is an additional absorption band at 3533 cm^−1^ in the IR spectrum of **3**‐OTf (3530 cm^−1^ for **3**‐PF_6_) which lies in the range of an OH stretch.^[^ ^25^, ^26^, ^27^ ^]^ Bands corresponding to a symmetric (*v*_s_) or antisymmetric (*v*_as_) Cu‐O stretch could not be identified clearly. DFT calculations indicate that *v*_s_ should be around 423 cm^−1^ and *v*_as_ around 494 cm^−1^. However, it was not possible to determine the Cu‐O stretches using Raman or rRaman spectroscopy on solid samples due to the strong fluorescence.

#### 2.1.7 HR‐ESI‐MS and XAS of 3

Since the **Cu_2_OH** complex **3** is stable at room temperature, it was further investigated with HR‐ESI mass spectrometry. In the mass spectrum of **3**‐OTf, a peak can be detected (calc. *m/z* 219.0544, obs. *m/z* 219.0540), the isotopic pattern and m/z value of which are consistent with the [Cu_2_(**MO8**) (OH)]^3+^ species [**3**]^3+^(Figure 4c). This trication is observed in the mass spectrum of **3**‐PF_6_ as well (Figure S63). In addition, the bare [Cu_2_(**MO8**)]^2+^ complex [**2**]^2+^ (calc. *m/z* 320.0805, obs. *m/z* 320.0801) and a peak that can be assigned to a [**3**‐H_2_O]^3+^ complex (calc. *m/z* 213.0509, obs. *m/z* 213.0506) (Figure S58,S59) are detected. These findings indicate that the **Cu_2_OH** complex **3** might be protonated and eliminate the hydroxo ligand as H_2_O in the mass spectrometer. Notably, a mono *μ*‐oxo [**Cu_2_** (**MO8**)**O**]^2+^ species [**4**]^2+^ (calc. m/z 328.0780, obs. m/z 328.0774) (Figure S60) is detected, too, which derives from deprotonation of the hydroxo complex **3** in the gas phase.

In order to differentiate a possible **Cu_2_O** intermediate **4** present in homogeneous solution (cf Section 3) from the **Cu_2_OH** complex **3**, X‐ray absorption near‐edge structure spectroscopy (XANES) and extended X‐ray absorption fine structure (EXAFS) analysis were employed. The XANES spectra of precursors **2**‐PF_6_ and **2**‐OTf are typical for Cu(I) complexes (Figure 4d, 6d, 7b, 8b)^[^ ^28^ ^]^ and very similar to the **bdpdz/bdptz** system.^[^ ^11^ ^]^ For **2**‐OTf, the edge position measured at 50% of the edge jump (E½) is at 8983.4 eV. A pronounced shoulder at ∼8981.5 eV and a weaker shoulder at ∼ 8985 eV are assigned to the electric‐dipole allowed transitions from Cu 1s to Cu 4p orbitals.^[^ ^28^, ^29^, ^30^, ^31^ ^]^ The EXAFS and XANES of **3**‐OTf are compatible with the presence of Cu(II) centers (Figure 4d). The edge position is shifted to a higher energy compared to **2**‐OTf (E½ = 8987.7 eV), and there is a pre‐edge peak at 8977.9 eV. The emergence of a pre‐edge is characteristic for both Cu(II) and Cu(III). However, Cu(III) compounds have a pre‐edge feature at ∼ 2 eV higher energies (∼8980 eV) than Cu(II) (∼8978 eV).^[^ ^32^, ^33^ ^]^ Thus, the Cu(II) valence state of this complex is confirmed. Moreover, the XAS data of **3**‐OTf are consistent with the crystal structure of **3**‐OTf and the DFT‐calculated **Cu_2_OH** structure (Section S11.4).

#### 2.1.8 EPR Spectroscopy

Apart from a small paramagnetic impurity (g = 2.045), which could already be observed in the Cu(I) complex **2**‐PF_6_ at 77 K (Figure S89a, black), the **Cu_2_OH** complex **3**‐PF_6_ is EPR‐silent (X‐band, 77 K; Figure S89a, blue). This is due to an antiferromagnetic coupling of the Cu(II) centers in the **Cu_2_OH** complex, as often observed for such systems.^[^ ^34^, ^35^, ^36^, ^37^ ^]^

### 2.2 Low‐Temperature Oxygenation of 2‐PF_6_

For the previously investigated dicopper complexes supported by the hexadentate ligands **bdpdz/bdptz**, it was shown that the formation of the **Cu_2_O** species proceeds via homolytic bond cleavage of a tetranuclear mixed‐valent *μ*_4_‐peroxo [Cu(I)/Cu(II)]_2_ complex (**Cu_4_O_2_**) that can be detected at low temperature (183 K) upon reaction of the Cu(I)‐precursor with O_2_.^[^ ^11^ ^]^ To check whether our new Cu_2_(**MO8**) system behaves similarly, **2**‐PF_6_ was exposed to O_2_ at 183 K, and the reaction was monitored by UV/Vis, rRaman, and XAS, as well as cryo‐UHR‐ESI mass spectrometry (cf Scheme 3, middle).

#### 2.2.1 UV/Vis Spectrum

**Figure 5:**
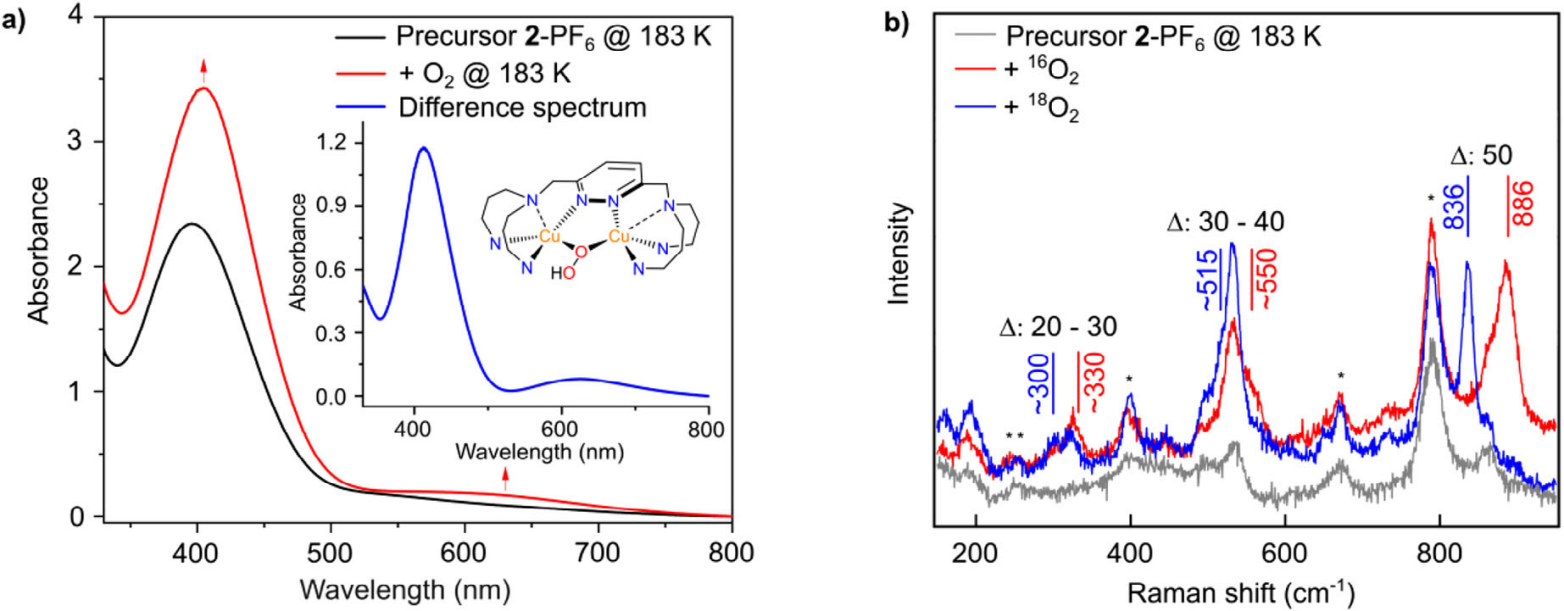
**Identifying the *μ*‐1,1‐hydroperoxo dicopper(II) intermediate 5. a)** Absorption spectra of an acetone solution of **2**‐PF_6_ before (black) and upon reaction with O_2_ (red) at 183 K. Inset: Difference Spectrum **b)** rR spectra of **2**‐PF_6_ before (gray) and after the reaction with ^16^O_2_ (red) and ^18^O_2_ (blue) at 183 K. General remarks: UV/Vis: l  =  1 cm. Raman: The asterisks mark solvent signals of acetone. The laser excitation wavelength was 420 nm.

Upon reaction of **2**‐PF_6_ with dioxygen at 183 K, the color of the solution changes from red to an intense darker red, going along with a change of the initial spectrum of the precursor (Figure 5a, black, *λ*_max_ = 396 nm and *ε* = 4700 m ^−1^cm^−1^) to a product spectrum containing a band at 412 nm (*ε* = 2400 m ^−1^cm^−1^) and a band at 625 nm (*ε* = 160 m ^−1^cm^−1^) (Figure 5a, inset). These bands can be assigned as a π*(OOH)→Cu(II) CT (412 nm) and a d‐d transition (625 nm) of a *μ*‐1,1‐hydroperoxo dicopper(II) (**Cu_2_OOH**) species **5**. Notably, similar absorption features around 400 nm and 600–650 nm have been observed for other **Cu_2_OOH** complexes as well, whereas *μ*‐1,2 peroxo dicopper complexes give qualitatively different spectra.^[^ ^7^, ^9^, ^27^, ^36^, ^38^, ^39^, ^40^, ^41^, ^42^, ^43^, ^44^ ^]^ The proton of the hydroperoxido ligand probably originates from traces of H_2_O in the solvent or from the solvent itself. To judge whether in principle a *trans μ*‐1,2 dicopper (**Cu_2_O_2_**) species could form at low temperature, we also employed DFT. Geometry optimization led to a *μ*‐1,2 peroxo structure (Figure S112) with a Cu‐O‐O‐Cu torsion angle of 89°, similar to the pyrazolate‐bridged system of Meyer et al.^[^ ^38^ ^]^ However, such a species would exhibit a two‐band UV/Vis spectrum with an additional absorption band at around 500 nm.^[^ ^38^ ^]^ We therefore can exclude a **Cu_2_O_2_** species resulting from low‐temperature oxygenation of the **MO8** system. Notably, electronic spectroscopy also excludes the formation of a **Cu_4_O_2_** intermediate, which had been observed upon low‐temperature oxygenation of the Cu(I) complexes supported by the **bdpdz**/**bdptz** ligands (see above). This marks an important difference between these systems and the new **MO8** complex. Finally, warming of the **Cu_2_OOH** complex of **MO8** does not lead to a **Cu_2_O** complex via O‐O cleavage, as observed for the **Cu_4_O_2_** species in the **bdpdz**/**bdptz** system. Rather, a thermal decay of the **Cu_2_OOH** complex is observed (Figure S30).

#### 2.2.2 rRaman

To further support formation of the **Cu_2_OOH** complex, rR spectroscopy with an irradiation wavelength of 420 nm was employed. Upon reaction of **2**‐PF_6_ with dioxygen (^16^O_2_ and ^18^O_2_) at 183 K, three isotope‐sensitive peaks emerge at 886 cm^−1^ (Δ = 50 cm^−1^), at around 550 cm^−1^ (Δ = 30–40 cm^−1^) and around 330 cm^−1^ (Δ = 20–30 cm^−1^; Figure 5b). DFT calculations performed for the **Cu_2_OOH** complex predict a symmetric and an antisymmetric Cu‐O stretching vibration at 282 cm^−1^ (Δ = 10–30 cm^−1^) and 495 cm^−1^ (Δ = 2 cm^−1^), respectively. The O‐O vibration is predicted to be at 936 cm^−1^ (Δ = 51–68 cm^−1^; Section S6.1). Calculations were performed without any coligands like acetone or acetonitrile; values with acetone or with acetonitrile can be found in Table S6. Correspondingly, the peak at 886 cm^−1^ is attributed to the O‐O stretch. Based on their frequencies, the observed modes around 550 cm^−1^ and 330 cm^−1^ are assigned to the antisymmetric and symmetric vibration of the **Cu_2_OOH** species **5**, respectively.

The vibrational frequencies and isotope shifts of the observed peaks are in good agreement with literature data for **Cu_2_OOH** complexes.^[^ ^7^, ^9^, ^27^, ^36^, ^38^, ^39^, ^40^, ^41^, ^42^, ^43^, ^44^ ^]^ Specifically, the O‐O stretch at ∼ 890 cm^−1^ rules out the formation of a *μ*‐1,2 **Cu_2_O_2_** complex, as such complexes exhibiting twisted Cu‐O‐O‐Cu units (see above) have O‐O stretching vibrations about 70–80 cm^−1^ lower in frequency.^[^ ^7^ ^]^

#### 2.2.3 Cryo‐UHR‐ESI‐MS

As a complement to the low‐temperature spectroscopic data, cryo‐UHR‐ESI MS data were collected. Upon reaction of an acetone solution of **2**‐PF_6_ with O_2_ at 183 K, the mass spectrum shows a peak with an isotopic pattern and *m/z* value corresponding to a doubly positively charged [**Cu_2_O_2_(MO8)**]^2+^ species (calc. *m/z* 336.0754, obs. *m/z* 336.0922). This is superimposed by a mixed‐valent *μ*‐chlorido dicopper(I/II) species of **2**‐PF_6_ (calc. *m/z* 337.5649, obs. *m/z* 337.5651; Section S9.1 / Figure S68) whereby the origin of the chloride is unknown. In the case of **Cu_2_OH** complex **3**, we have already observed that a **Cu_2_O** complex was detectable under the conditions of mass spectrometry. It is therefore conceivable that the observed **Cu_2_O_2_** species is formed by deprotonation of the **Cu_2_OOH** complex **5** under the same conditions. Finally, we obtained a spectrum that is in excellent agreement with the calculated spectrum and isotopic distribution pattern of a doubly positively charged [**Cu_2_** (**MO8**)**O**]^2+^ species [**4**]^2+^ (calc. *m/z* 328.0780, obs. *m/z* 328.0785; (Section S9.2/ Figure S70). This indicates that also a **Cu_2_OOH** complex is partly converted to a **Cu_2_O** complex in the gas phase. By contrast, a **Cu_4_O_2_** complex could not be detected for **2**‐PF_6_ in the MS experiment, in agreement with the UV/vis and Raman data. We assume that, due to the sterically more demanding ligand design of **MO8** compared to **bdpdz**/**bdptz**, this intermediate is not stable. DFT calculations support this hypothesis; that is, geometry optimization of a **Cu_4_O_2_** starting structure leads to a unit of the dicopper(I) complex **2** moving away from the **Cu_4_O_2_** core, leaving behind a *μ*‐1,1‐peroxo dicopper (**Cu_2_O_2_**) species (Section S12.1/ Figure S109).

#### 2.2.4 XAS

To characterize the oxidation state and structure of the species generated by the addition of oxygen to the precursor **2**‐PF_6_ at ∼ 190 K, XAS measurements were performed. They showed that upon addition of oxygen, Cu(I) was partially oxidized (Section S11.2, Figure S90). However, since the conversion to the oxidized species was not complete, structural analysis of this species could not be performed.

#### 2.2.5 Reactivity of the Cu_2_OOH Complex

The UV/Vis and Raman spectroscopic results presented above indicate that upon low‐temperature oxygenation (183 K) of the Cu_2_(**MO8**) system, a *μ*‐1,1‐hydroperoxo dicopper(II) intermediate (**5**) is formed (Scheme 3, middle). The ability of **5** to catalyze the monooxygenation of hydrocarbons was evaluated with 9,10‐dihydroanthracene (DHA; BDE = 78 kcal mol^−1^) and anthrone (AT; BDE = 76 kcal mol^−1^). Upon addition of 5 equiv. of DHA to the **Cu_2_OOH** complex of Cu_2_(**MO8**), no reaction was observed. However, the reaction of **5**‐PF_6_ and **5**‐OTf with 5 equiv. of AT gave 9,10‐anthraquinone (AQ) with a TON of 1.1 (for **5**‐PF_6_) and 1.0 (for **5**‐OTf), corresponding to a stoichiometric conversion. Blind reactions were also performed using [Cu(NCMe)_4_]PF_6_ or [Cu(NCMe)_4_]OTf and O_2_ instead of **2**, which led to a TON of 0.3 (for [Cu(NCMe)_4_]PF_6_) and a TON of 0.4 (for [Cu(NCMe)_4_]OTf); that is, to poorer results compared to the activity of **5** (see Table S18).

Oxygen transfer to substrates by **Cu_2_OOH** complexes has been described in the literature. For example, oxygen could be transferred stoichiometrically to PPh_3_ or tetramethylene sulfide by a **Cu_2_OOH** species, whereby O  =  PPh_3_ or tetramethylene sulfoxide are formed.^[^ ^36^, ^45^ ^]^ Furthermore, it was observed that in some ligand systems the **Cu_2_OOH** species led to arene hydroxylation of the aromatic xylene backbone.^[^ ^40^, ^46^ ^]^ The **Cu_2_OOH** species of Cu_2_(**MO8**) may behave similarly toward AT.

**Scheme 4:**
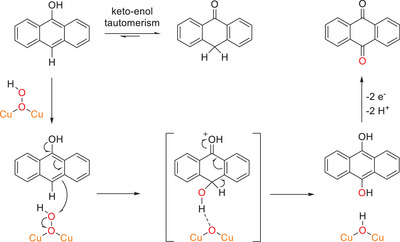
**Reactivity of the Cu_2_OOH complex of MO8 toward** AT. In solution, AT is in equilibrium with the aromatic 9‐anthrol via keto‐enol tautomerism. Oxygenation through the **Cu_2_OOH** species **5** could proceed via aromatic hydroxylation (see text).

In solution, a keto‐enol equilibrium between AT and 9‐anthrol is present, depending on the solvent.^[^ ^47^ ^]^ (Scheme 4, top). The *para* position of the hydroxyl group in 9‐anthrol is probably the most favorable for electrophilic attack by the **Cu_2_OOH** species, since this position is activated by the OH group and the other positions are blocked by the neighboring benzene rings. Assuming that the reaction proceeds as an aromatic hydroxylation (cf Scheme 4, bottom), a **Cu_2_OH** species would result that is inactive toward further oxygen transfer (see below).^[^ ^40^ ^]^ This would explain the fact that the reaction is stoichiometric under these conditions. Further oxidation of the anthrahydroquinone leads to AQ.

### 2.3 Room Temperature Oxygenation of 2‐PF_6_

#### 2.3.1 UV/Vis

**Figure 6:**
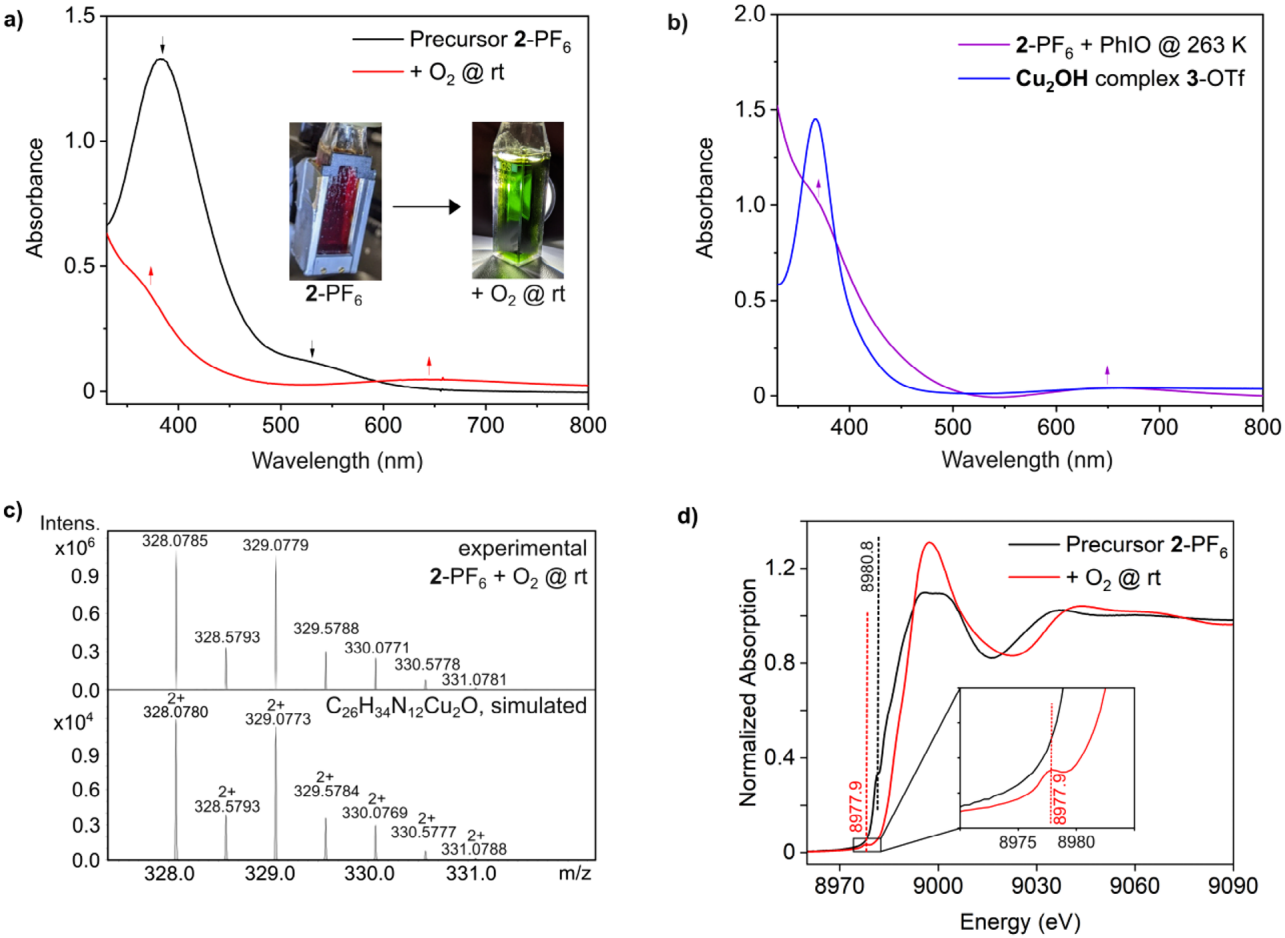
**Reaction of 2‐PF_6_ with O_2_ at room temperature**. **a)** Absorption spectra of an acetone solution of **2**‐PF_6_ before (black) and upon reaction with O_2_ (red) at room temperature. **b)** The reaction of **2**‐PF_6_ dissolved in acetone with an excess of PhIO (violet) at 263 K resulted in a green solution and comparable absorption features to the spectrum obtained after reaction with O_2_ at room temperature. The spectrum of the **Cu_2_OH** complex **3**‐OTf (blue) differs from the spectra of the room temperature oxygenation experiments. **c)** Characteristic cutout of the UHR‐ESI mass spectrum obtained upon reaction of **2**‐PF_6_ with ^16^O_2_ at room temperature, showing the formation of the mono *μ*‐oxo complex. The corresponding species is also detected in the experiment with ^18^O_2_ and upon reaction with PhIO at 263 K and N_2_O at 293 K (see Section S9, Figures S73‐S77). **d)** Cu K‐edge XANES spectra of the precursor **2**‐PF_6_ before (black line, Cu(I)) and after the reaction with dioxygen at 293 K (red line) with enlarged pre‐edge region. General remarks: UV/Vis: l  =  1 cm.

Upon oxygenation of a solution of the dicopper(I) complex **2**‐PF_6_ in acetone at 293 K, a color change from red to an intense green is observed within 24 hours, going along with a change of the initial spectrum of the precursor (Figure 6a, black) to a product spectrum containing one shoulder at 360 nm (*ε* = 1900 m ^−1^cm^−1^) and a band at 645 nm (*ε* = 200 m ^−1^cm^−1^) (Figure 6a, red). Because the intense green color (Figure 6a, inset) has been associated with *μ*‐oxo moieties in dicopper complexes,^[^ ^6^, ^11^, ^46^ ^]^ we initially assumed that a **Cu_2_O** core is formed in our Cu_2_(**MO8**) system as well. However, this hypothesis had to be abandoned upon closer examination. For simplicity, the room‐temperature oxygenation product of **2** is therefore denoted as “green species” in the following (cf Scheme 3 top left).

To obtain further information, **2**‐PF_6_ was also reacted with the common oxygen‐atom transfer (OAT) reagent iodosobenzene (PhIO)^[^ ^6^, ^11^ ^]^ in acetone at 263 K. In this case, the color changed from red to intense green within 2 hours, and a spectrum emerged (Figure 6b, violet) that was very similar to that obtained with O_2_, exhibiting one shoulder at 360 nm (*ε* = 1300 m ^−1^cm^−1^) and a distinct absorption band at 651 nm (*ε* = 50 m ^−1^cm^−1^). In addition, we investigated whether the same species can also be obtained using nitrous oxide (N_2_O) as an OAT reagent.^[^ ^48^ ^]^ For this purpose, **2**‐PF_6_ was reacted with N_2_O for 48 hours at 308 K (Figure S33, red). Again, a color change from red to intense green occurred, and the resulting spectrum was found to be similar to that obtained for the reaction with O_2_ and PhIO, exhibiting one shoulder at 355 nm (*ε* = 4800 M^−1^cm^−1^) and a distinct absorption band at 645 nm (*ε* = 90 M^−1^cm^−1^). It should be noted that the ratio of these two extinction coefficients differs depending on the oxygen reagent being used. While the reaction with PhIO is completed within 2 hours at 263 K, the reaction with O_2_ takes 24 hours at 293 K and with N_2_O 48 hours at 308 K. Therefore, the different ratios of the extinction coefficients might reflect differences in the completeness of the oxygenations. In any case, however, the obtained spectra are different from that of the **Cu_2_OH** complex **3** (Figure 6b).

Absorption features around 600 nm have been observed for and associated with **Cu_2_O** complexes.^[^ ^6^, ^11^, ^12^, ^15^, ^35^, ^49^ ^]^ Compared with the Cu_2_(**bdpdz**/**bdptz**) systems studied earlier,^[^ ^11^ ^]^ however, significantly lower *ε* values are observed for the **MO8** complex. Moreover, in the case of the former systems^[^ ^11^ ^]^ the **Cu_2_O** intermediates were only stable up to ‐35°C whereas the “green species” is stable at room temperature.

#### 2.3.2 rRaman

In order to check whether the “green species” corresponds to a **Cu_2_O** complex or not, rR spectroscopy was employed, using an irradiation wavelength of 360 nm. However, upon reaction of **2**‐PF_6_ with dioxygen at room temperature, no isotope‐sensitive peak around 600–630 cm^−1^,^[^ ^4^, ^6^, ^10^, ^11^, ^12^, ^15^, ^50^ ^]^ as expected for a **Cu_2_O** species, could be detected. Instead, a spectrum was obtained that is consistent with the Cu(I) precursor (Figure S36). Nevertheless, the UV/Vis spectra shown in Figure 6a,b indicate that the species obtained after oxygenation is not the precursor complex, and the green color of this intermediate could also be observed when recording the rR‐data. We thus conclude that the “green species” is photolabile under the conditions of the Raman experiment.

#### 2.3.3 IR Spectroscopy

As Raman spectroscopy proved to be ineffective, IR spectroscopy was employed to obtain information regarding the nature of the “green species.” Interestingly, the obtained spectrum (Figure S47) shows two bands in the frequency range of O‐H stretches, suggesting the presence of two hydroxo ligands. Based on DFT, a *μ*‐hydroxo hydroxo complex **Cu_2_(*μ*‐OH)OH** (**6**) (structure cf. Figure S51) exhibits two O‐H bands in the IR spectrum with the observed splitting (Table S8). Such a species could result from hydration of a mono *μ*‐oxo dicopper (**Cu_2_O**) complex formed initially by oxygenation of the Cu(I)_2_ **MO8** complex **2**. Alternatively, it could derive from the reaction of an initially formed **Cu_2_OOH** complex with a residual Cu(I) complex. Importantly, EXAFS data do not exclude the possibility that the green species corresponds to **6** (see below).

#### 2.3.4 Cryo‐UHR‐ESI‐MS

Reaction of **2**‐PF_6_ with O_2_ at 293 K provides the mass spectrum shown in Figure 6c, which is in excellent agreement with the calculated spectrum and isotopic distribution pattern of a doubly positively charged **Cu_2_O** species [**4**]^2+^ (calc. *m/z* 328.0780, obs. *m/z* 328.0785; similar data obtained with PhIO and N_2_O are shown in Sections S9.3, S9.4). Upon reaction with ^18^O_2_, the peak shifts by one mass unit to *m/z* 329.0781, as expected (calc. *m/z* 329.0801, Figure S73). It should be noted that acetonitrile had to be used as a solvent instead of acetone to detect the Cu_2_^18^O species. In acetone, even when using ^18^O_2_, only the Cu_2_^16^O complex could be detected (see Section S9.2). We ascribe this to the exchange of ^18^O with the solvent. Moreover, we observed a mass spectrum that is in excellent agreement with a doubly positively charged **Cu_2_O_2_** species [**Cu_2_(MO8) (O_2_)**]^2+^ (calc. *m/z* 336.0754, obs. *m/z* 336.0759; Section S9.6).

To conclude, cryo‐UHR ESI‐MS does provide evidence for the formation of a **Cu_2_O** complex (**4**) resulting from room‐temperature oxygenation of the Cu(I)_2_ **MO8** precursor **2**. However, as **4** has also been observed by ESI‐MS under conditions where it clearly is *not* present in homogeneous solution (see above), we cannot take this result as proof for the contention that the ´green species´ corresponds to **4**. If the ´green species´ instead corresponds to the dihydroxo complex **6**, as suggested by IR (see above), the **Cu_2_O** species observed by MS might derive from dehydration of **6** in the gas phase.

#### 2.3.5 XAS

Upon oxygenation of the copper(I) precursor (**2**‐PF_6_) with O_2_ at room temperature for 24 hours, E½ in the Cu K‐edge XANES spectra shifts to higher energy by 3.9 eV (8983.4 eV to 8987.3 eV), reflecting the transition from Cu(I) to Cu(II). Concomitantly, a pre‐edge feature appears at 8977.9 eV, reflecting the emergence of Cu(II) character (Figure 6d).^[^ ^29^, ^30^, ^32^ ^]^ However, of several different models tested (Figure S91), EXAFS is not pointing to one model with certainty. In particular, it is not possible by detailed EXAFS analysis to associate a **Cu_2_O** structure to the Cu(II) species formed (Section S11.2).

#### 2.3.6 EPR Spectroscopy

In contrast to the **Cu_2_OH** complex **3**‐PF_6_, a frozen solution of the “green species” exhibits an EPR signal with g = 2.092 (Figure S89b) in the X‐band EPR spectrum measured at 77 K, which suggests the presence of Cu(II).^[^ ^51^ ^]^ The signal also indicates a weaker antiferromagnetic coupling of the Cu(II) centers for the “green species” compared to the **Cu_2_OH** complex **3**‐PF_6_. The reason for this could be a geometric distortion of the system caused by the second hydroxido ligand that is present in the dihydroxo complex **6** (see above and Figure S51). Notably, the observation of an EPR signal is also incompatible with the presence of a **Cu_2_O** species, which exhibits strong antiferromagnetic coupling.^[^ ^11^ ^]^

### 2.4 Attempt to Generate the Mono *μ*‐oxo Dicopper Complex with dimethyldioxirane (DMDO) at Low Temperature

Since generation of a **Cu_2_O** species with our Cu_2_(**MO8**) system at room temperature could not be demonstrated so far, we tried in the next step to generate a **Cu_2_O** species at low temperatures using the highly reactive oxygen atom transfer reagent DMDO.^[^ ^52^ ^]^ We anticipated that the reaction of **2**‐PF_6_ with DMDO leads to the **Cu_2_O** complex **4**‐PF_6_. The potential higher stability of a **Cu_2_O** species at low temperature could facilitate spectroscopic characterization of this complex. UV/Vis spectroscopy, Cryo‐UHR‐ESI‐MS, and XAS were employed to investigate this reaction (Scheme 3, right).

#### 2.4.1 UV/Vis

**Figure 7:**
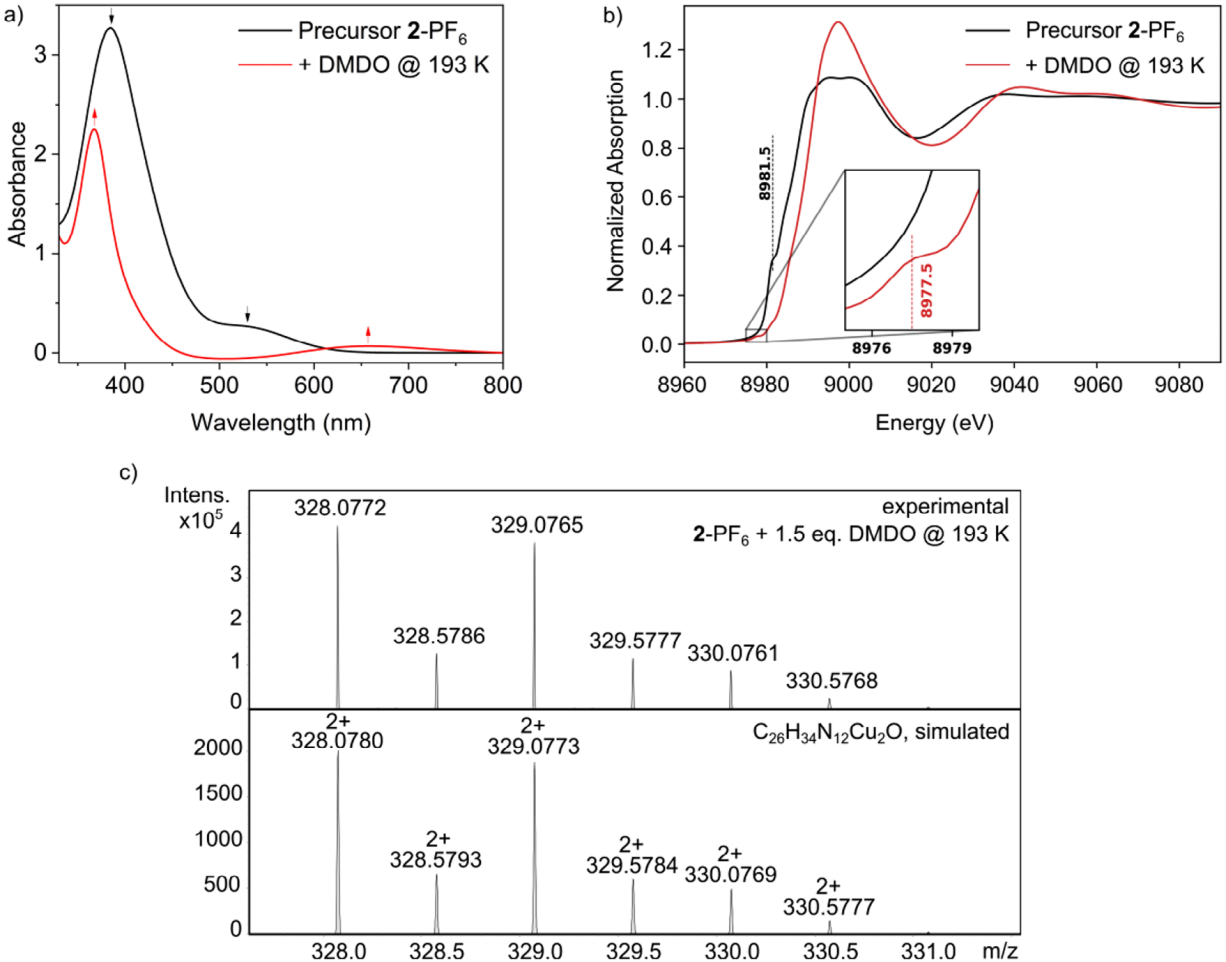
**Reaction of 2‐PF_6_ with DMDO at 193 K. a)** UV/Vis spectrum obtained after reaction of **2**‐PF_6_ with DMDO at 193 K. The spectrum shows comparable features to the experiments with O_2_ (at rt), PhIO, and N_2_O. **b)** Cu K‐edge XANES spectra of the precursor **2**‐PF_6_ before (black line, Cu(I)) and after the reaction with DMDO at 193 K (red line) with enlarged pre‐edge region. **c)** Characteristic cutout of the UHR‐ESI mass spectrum obtained upon reaction of **2**‐PF_6_ with DMDO at 193 K, indicating the formation of the mono‐*μ*‐oxo complex.

Analogous to the reaction with oxygen at room temperature, the solution of **2**‐PF_6_ changed its color to green after an addition of DMDO at 193 K. The UV/Vis spectrum revealed an absorption band at 368 nm (*ε* = 4500 m ^−1^cm^−1^) and a band at 660 nm (*ε* = 135 m ^−1^cm^−1^) (Figure 7a). The UV/Vis bands are in the same position as in the experiments with O_2_, PhIO, and N_2_O, which indicates that the same copper‐oxygen intermediate could have been generated in this experiment. In the case of oxygenation with DMDO, however, the bands are more pronounced, possibly due to a better stabilization of this species at the lower temperature. Warming up to room temperature led to a decrease of both bands in the UV/Vis spectrum and to a fading of the green color of the solution, indicating a decay of the complex.

#### 2.4.2 Cryo‐UHR‐ESI‐MS

To gain further information in the generated copper‐oxygen intermediate, Cryo‐UHR‐ESI‐MS measurements were performed. After the reaction of **2**‐PF_6_ with DMDO at 193 K, a mass spectrum (Figure 7c) was obtained which, as in the experiments with O_2_, PhIO, and N_2_O, corresponds to the calculated spectrum and isotopic distribution pattern of the **Cu_2_O** species [**4**]^2+^ (calc. *m/z* 328.0780, obs. *m/z* 328.0772). As pointed out before, however, this species may result from deprotonation of the **Cu_2_OH** complex **3** the presence of which in homogeneous solution is indicated by XAS (see below). The addition of a large excess of DMDO or the addition of DMDO at rt resulted in a relatively smaller peak of the **Cu_2_O** species [**4**]^2+^ as well as more decomposition products, probably due to the high reactivity of this OAT (Figure S87).

#### 2.4.3 XAS

**2**‐PF_6_ was also reacted with DMDO at 193 K, and the reaction was monitored by XAS. Upon oxygenation of **2**‐PF_6_ with DMDO, the intensity of the shoulder at 8981.5 eV in the Cu K‐edge XANES spectrum is strongly reduced, resulting in the shift of the edge position E½ to higher energy by 3.6 eV (8983.9 eV to 8987.5 eV). A pre‐edge feature appears at 8977.5 eV (Figure 7b, S92). Thus, we can identify the oxidized species as a Cu(II) complex. Comparison of spectra obtained by oxidation of **2**‐PF_6_ with oxygen and with DMDO is shown in Figure S94.

Further structural insights are provided by extended EXAFS analysis. Comparison of the fit results for different models is shown in Section S11.3 (Figures S95–S99). It follows that the copper‐oxygen intermediate that best fits the EXAFS data is the crystal structure of the *μ*‐hydroxo dicopper(II) (**Cu_2_OH**) species (**3**). Specifically, for the **Cu_2_O** species, a copper‐oxygen distance of Cu‐O  =  1.89 Å (about the same value as determined for the **Cu_2_O** intermediate of the **bdpdz**/**bdptz** system)^[^ ^11^ ^]^ and Cu‐N_Amine_ distances of 2.29 Å and 2.36 Å are calculated. For the **Cu_2_OH** complex **3,** the corresponding values are Cu‐O  =  1.95 Å; Cu‐N_Amine_  =  2.17 Å and 2.18 Å (RI‐PBE‐D3(BJ)/def2‐SVP^[^ ^23^, ^24^ ^]^). Notably, there is also evidence for a **Cu_2_OH** species in the Cryo‐UHR‐ESI‐MS (see Figure S79), but the intensity of this species is much smaller than that of the **Cu_2_O** complex.

In summary, the spectroscopic data indicate that targeted oxygen transfer to **2**‐PF_6_ is possible with DMDO at low temperatures, but the characterization with XAS rather indicates the presence of a **Cu_2_OH** complex that may result from protonation of a **Cu_2_O** complex formed initially (cf Scheme 3, right).

### 2.5 Attempt to Generate the Mono *μ*‐oxo Dicopper Complex by Deprotonation of the *μ*‐Hydroxo Complex

It has been shown in the literature that a **Cu_2_O** species can be formed by deprotonation of a **Cu_2_OH** complex.^[^ ^53^ ^]^ Therefore, we wanted to investigate if the **Cu_2_OH** complex **3** can be deprotonated with the base diazabicycloundecene (DBU) to generate a **Cu_2_O** species (Scheme 3, right). The reaction was monitored using UV/Vis spectroscopy and XAS.

#### 2.5.1 UV/Vis

For the deprotonation experiment, a solution of **3**‐OTf in acetone was cooled to 223 K, and 1.5 eq. DBU was added. The 0.5 mM solution used for low‐temperature UV/Vis spectroscopy changed its color from green to orange within 90 minutes after adding DBU and warming to 233 K. This resulted in a UV/Vis spectrum with bands at 370 nm (*ε* = 5300 m ^−1^cm^−1^), 550 nm (*ε* = 210 m ^−1^cm^−1^), 680 nm (*ε* = 125 m ^−1^cm^−1^) and 860 nm (*ε* = 90 m ^−1^cm^−1^). The color of the solution and the shape of the spectrum are similar to Cu(I) complex **2**.

**Figure 8:**
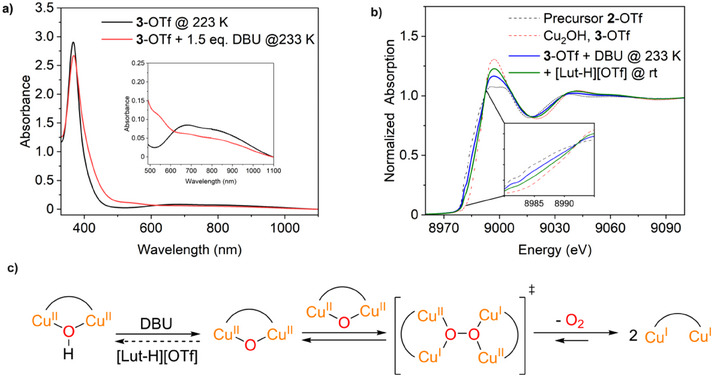
**Deprotonation of the Cu_2_OH species 3. a)** UV/Vis spectra of the **Cu_2_OH** complex **3**‐OTf before (black) and after deprotonation with DBU (red). **b)** EXAFS spectra of the Cu(I) complex **2**‐OTf (black) and **Cu_2_OH** complex **3**‐OTf (red) from Figure 4 and spectra obtained after the deprotonation with DBU at 233 K (blue) and after addition of [Lut‐H][OTf] at rt (green). **c)** Proposed reaction sequence: At first the deprotonation of the **Cu_2_OH** complex might lead to a **Cu_2_O** species. However, when two **Cu_2_O** cores interact, an O‐O bond might be formed and generate a **Cu_4_O_2_** transition state, which immediately releases O_2_.

Furthermore, the band at 680 nm, which indicates a Cu(II) d‐d transition, has decreased significantly (Figure 8a, red). These findings suggest that a Cu(I) complex is formed when DBU is added to the **Cu_2_OH** species. We then checked whether the observed reaction was reversible by adding an acid. For this purpose we used 2,6‐lutidinium triflate ([Lut‐H][OTf]). After the addition of 1.5 eq. [Lut‐H][OTf] and warming to room temperature, the initial spectrum of **Cu_2_OH** complex **3** could only be partially restored (Figure S25). The color of the solution was paler than the **Cu_2_OH** complex before the addition of DBU and the bands were not as intense, which indicates that irreversible processes take place during deprotonation.

#### 2.5.2 XAS

The deprotonation of the **Cu_2_OH** complex was also monitored using XAS with a 16 mM solution of **3**‐OTf in acetone. To this end, 1.5 eq. DBU was added to an acetone solution of **3**‐OTf at 233 K, and the reaction mixture was stirred for 2 hours. The XAS measurement showed that a large part of the Cu(II) complex was converted to Cu(I) after reaction with DBU, which supports the observed results from the low‐temperature UV/Vis spectroscopy. Linear combination fit (LCF) of the XANES spectrum confirmed that the resulting solution consisted of **2**‐OTf (64%) and **3**‐OTf (36%) (Figure 8b, blue, Figure S103). As in the UV/Vis experiment, the addition of 1.5 eq. [Lut‐H][OTf] only partially restored the Cu(II) species (according to LCF, the resulting solution consisted of 35% **2**‐OTf and 65% **3**‐OTf) (Figure 8b, green, Figure S104). The reaction of the **Cu_2_OH** complex with DBU could initially form a **Cu_2_O** complex. Reaction between two **Cu_2_O** units may form an O‐O bond and a transient **Cu_2_O_4_** species. Subsequent release of O_2_ could then lead to a Cu(I) complex (Figure 8c). Notably, this reaction sequence corresponds to the reversal of the reaction of the former **bdpdz**/**bdptz** system with O_2_, *generating* mono *μ*‐oxo dicopper complexes from the Cu(I) precursor and O_2_.^[^ ^11^ ^]^ In summary, this experiment indicates that a **Cu_2_O** species is unstable in the **MO8** system as it decays to Cu(I) and O_2_.

### 2.6 Reactivity of the “Green Species”

In the last step, we investigated the ability of the “green species” to catalyze the monooxygenation of hydrocarbons. This was evaluated with a range of aliphatic substrates exhibiting bond dissociation energies (BDEs) from 75 to 82 kcal mol^−1^; that is, 9*H*‐xanthene (XEN; BDE = 75 kcal mol^−1^),^[^ ^54^, ^55^ ^]^ 10*H*‐anthracen‐9‐one also denoted as (AT; BDE = 76 kcal mol^−1^),^[^ ^56^ ^]^ 9,10‐dihydroanthracene (DHA; BDE = 78 kcal mol^−1^)^[^ ^34^, ^55^, ^57^ ^]^ and diphenylmethane (DPM; BDE = 82 kcal mol^−1^)^[^ ^54^ ^]^ at room temperature (Table 1 and Section S13.1). Specifically, oxygenation of 10 equiv. of xanthene with the “green species” resulted in no conversion to 9*H*‐xanthen‐9‐one (XON) at all. A negative result was also obtained when the conversion of DHA to 9,10‐AQ was tested; that is, upon addition of 10 equiv. of DHA to the “green species,” no reaction was observed (Table 1). These results already indicated that the monooxygenation activity of the Cu_2_(**MO8**) system is lower than that of the original **bdpdz/bdptz** complexes. Correspondingly, no conversion to benzophenone (Ph_2_CO) was observed upon addition of 10 equiv. of diphenylmethane to the “green species.”

**Table 1:** **Catalytic activities for the oxygenation of hydrocarbons**. Overview of the catalytic activities of the new model system Cu_2_(**MO8**) after oxygenation at room temperature compared to the activities of our initial systems [Cu_2_(**bdpdz**) (NCMe)_2_]X_2_ and [Cu_2_(**bdptz**) (NCMe)_2_]X_2_ toward various substrates.

BDE/kcal mol^−1^	Substrate→Product	TON^[a]^ bdpdz/bdptz	TON^[b]^ 2‐PF_6_/‐OTf
75	XEN→XON	3/2^[^ ^11^ ^]^	0/0
76	AT→AQ	2/2	9/9
78	DHA→AQ^[c]^	3/5^[^ ^11^ ^]^	0/0
82	DPM→Ph_2_CO	2/2^[^ ^11^ ^]^	0/0

General Remarks: Conditions:1 equiv. of **2** and 10 equiv. of 9*H*‐xanthene (XEN), 10*H*‐anthracen‐9‐one (anthrone /AT), 9,10‐dihydroanthracene (DHA), or 50 equiv. of diphenylmethane (DPM) were applied (cf. Section S13.1). Blind reactions were also performed using [Cu(NCMe)_4_]PF_6_ or [Cu(NCMe)_4_]OTf and O_2_ instead of **2**. All control experiments lead to poorer results compared to the activity with **2** (see Table S17). For abbreviations, see text.

^[a]^ The **Cu_2_O** complex was reacted at ‐35°C with the substrate for 2 hours and additional an 2 hours at room temperature.^[^ ^11^ ^]^

^[b]^ Conditions for the Cu_2_(**MO8**) model systems: +35 °C, 2 hours of O_2_ bubbling in the beginning; total time of the reaction: 24 hours.

^[c]^ 10 equiv. of DHA were used, representing a 20‐fold excess of substrate. The given TON refers to the initially formed disecondary alcohol and does not include the subsequent oxidation.

Finally, AT was investigated as a substrate. Here we found that oxygenation of 10 equiv. of AT leads to 9,10‐AQ with a TON of 9 (∼90% conversion) (Table 1). This clearly demonstrates that **2**‐PF_6_/OTf is principally able to convert aliphatic substrates like AT to the corresponding ketones after oxygenation at room temperature, although its activity is much lower than that of the original **bdpdz/bdptz** system.^[^ ^11^ ^]^ It should be mentioned that AT is already partially oxidized in solution with O_2_ to AQ, but only to a very small extent (6% at rt (see Table S17).

**Scheme 5:**
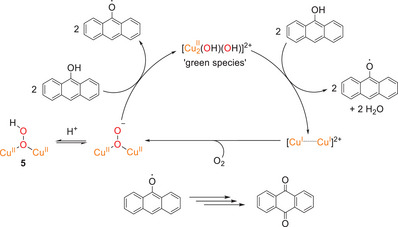
**Reactivity of the** “**green species” of MO8 toward** AT. At room temperature an initially formed **Cu_2_OO** species might form a **Cu_2_(OH)OH** complex which generates an anthroxyl radical via O‐H abstraction of anthrol. AQ could then be formed by a further reaction of the anthroxyl radical with atmospheric O_2_.

As indicated above (Scheme 4), AT is subject to a ketol‐enol tautomerism. The resulting enol (9‐anthrol) has a lower BDE (O‐H) of 72 kcal mol^−1 [^ ^47^, ^56^ ^]^ than the keto form (BDE (C‐H) = 76 kcal mol^−1 [^ ^47^, ^56^ ^]^), which could enable a reaction via an initial O‐H abstraction (Scheme 4, left). This would also explain why no reaction is observed for **2**‐PF_6_/OTf with xanthene, although its BDE (C‐H) is lower than that of AT (see Table 1). Thus, starting from a **Cu_2_(*μ*‐OH)OH** species (**6**), two anthroxyl radicals may be generated via H‐atom abstraction from anthrol, whereby a dicopper(I) complex is formed and water is eliminated. The anthroxyl radical might then react with atmospheric O_2_ to form AQ. The Cu(I) complex could react with O_2_ to form a *μ*‐1,1 peroxo complex, which, by two‐electron reduction from further anthrol molecules, converts to **6**, thus closing the catalytic cycle (Scheme 5). To compare this result with our former **Cu_2_O** models, the activities of the **bdpdz** and **bdptz** systems toward the conversion of AT to AQ were determined as well. Interestingly, employing 10 equiv. of AT led to a TON of 2; that is, a *lower* yield than obtained with the Cu_2_(**MO8**) system.

For comparison, we also investigated the reaction of the **Cu_2_OH** complex **3**‐OTf toward AT. The reaction was carried out at room temperature for 24 hours in analogy to the experiments with the “green species.” In a first attempt, the reaction was carried out under an inert atmosphere and in a second attempt, with an O_2_‐saturated solution (under the same conditions as the “green species.”) Under an inert atmosphere, only 0.1 eq. of the substrate was converted to AQ. In the O_2_‐saturated solution 0.6 eq. of AQ were formed, similar to the reactivity of the [Cu(NCMe)_4_]OTf precursor toward AT (see Table S19). The reactivity of the **Cu_2_OH** complex **3**‐OTf toward AT thus is significantly lower than observed for the **Cu_2_OOH** complex **5** and the “green species.”

In conclusion, the Cu_2_(**MO8**) system shows weaker C‐H activation than our earlier model systems based on the ligands **bdpdz** and **bdptz**.^[^ ^11^ ^]^ However, it is capable of almost quantitative conversion of AT to AQ (TON = 9 for 10 equivalents), whereas the corresponding reaction catalyzed by **bdpdz** or **bdptz** only leads to a TON of 2.

## 3 Conclusion

Herein the synthesis of a new dinuclear Cu‐monooxygenase model system based on the octadentate ligand **MO8** was described. Compared to our previous systems, which were based on the hexadentate ligands **bdpdz** and **bdptz**, no additional coligands coordinate to copper in the Cu(I) complex, which could be shown by the crystal structure of **2**‐PF_6_. Depending on the oxygenation conditions, different copper‐oxygen intermediates are generated. In contrast to the **bdpdz**/**bdptz** system, the reaction of **2**‐PF_6_ with O_2_ at 183 K does not lead to a **Cu_4_O_2_** complex. DFT calculations suggest that such a tetranuclear core is not stable in the Cu_2_(**MO8**) system due to steric reasons. Instead, the spectroscopic data reveal the formation of a **Cu_2_OOH** complex. A reactivity study of **Cu_2_OOH** toward aliphatic hydrocarbons showed that (AT, 76 kcal mol^−1^) is stoichiometrically converted to AQ.

Oxygenation of **2**‐PF_6_ with O_2_ at room temperature (or PhIO at 263 K, or N_2_O at 308 K) generates a solution containing the “green species,” which we initially assumed to be a **Cu_2_O** complex. This hypothesis could not be verified spectroscopically, in contrast to the **bdpdz** and **bdptz** systems investigated earlier.^[^ ^11^ ^]^ Notably, the IR spectrum of the “green species” shows two bands in the typical frequency range of O‐H stretching vibrations, which could be due to a *μ*‐hydroxo/hydroxo (**Cu_2_(*μ*‐OH)OH**) complex. This assumption was supported by DFT calculations, and an EXAFS analysis also indicates that this might be a possibility.

The reactivity of the “green species” toward monooxygenation of aliphatic hydrocarbons was investigated with various substrates exhibiting BDEs from 75 to 82 kcal mol^−1^ at room temperature. The system was only able to convert the substrate AT to AQ, but with an almost quantitative yield. Overall, however, the monooxygenase activity of the **MO8** system is distinctly lower than that of the **bdpdz**/**bdptz** systems forming **Cu_2_O** intermediates upon oxygenation.

Nevertheless, an attempt was made to generate, stabilize, and characterize a **Cu_2_O** species at low temperatures, using the highly reactive OAT reagent DMDO. While UV/Vis and Cryo‐UHR‐ESI‐MS support the formation of a **Cu_2_O** species, XAS data are rather compatible with the presence of a **Cu_2_OH** species. This complex was synthesized independently and employed for an attempt to generate the **Cu_2_O** complex by deprotonation with diazabicycloundecene (DBU). The UV/Vis and XAS data, however, revealed that a Cu(I) complex is formed after the addition of DBU. Thus, deprotonation might initially lead to a **Cu_2_O** species, which, however, decays to Cu(I) and O_2_.

In contrast to the **bdpdz**/**bdptz** systems investigated earlier, it thus appears that the **Cu_2_O** species of the **MO8** system is unstable in two ways: in the presence of base (i.e., when protons are absent), it decays into Cu(I) and O_2_. In the presence of H^+^ (probably deriving from water), it either gets protonated, leading to a **Cu_2_OH** complex, or even hydrated, leading to a dihydroxo complex, supposedly a **Cu_2_(*μ*‐OH)OH** species. This reflects an increased Brønsted basicity of the **Cu_2_O** core with respect to the **bdpdz**/**bdptz** systems, which probably derives from the octadentate constitution of the **MO8** ligand, providing two nitrogen donors in *trans*‐position to the *μ*‐oxo unit. In the hexadentate **bdpdz**/**bdptz** systems, by contrast, these additional nitrogen atoms were absent, giving rise to a more electrophilic character of the **Cu_2_O** core. These results establish an important structure‐function correlation in copper‐oxygen chemistry regarding the formation and stability of the rare and much sought‐after mono *μ*‐oxo dicopper cores.

Although we found that the targeted **Cu_2_O** core cannot be stabilized in the **MO8** system, access to other reactive copper‐oxygen species is possible. This is evidenced by catalytic conversion of the organic substrate AT to AQ that does not depend upon a mono *μ*‐oxo dicopper intermediate.

## Conflict of Interests

The authors declare no conflict of interest.

## Supporting information



Supporting Information

## Data Availability

The data that support the findings of this study are available in the supplementary material of this article.
