# Supplementary material for: Dinuclear Copper Complex with a Pyridazine‐Bridged Octadentate Ligand: Monooxygenase Activity and Characterization of Copper‐Oxygen Intermediates
Source: Chemistry. 2025 Jun 3;31(37):e202501659. doi: 10.1002/chem.202501659 (PMC12223343; doi:10.1002/chem.202501659)
Supplement: Supplementary file 1 — Supporting Information [file CHEM-31-e202501659-s001.pdf]

Supporting Information
©Wiley-VCH 2025
69451 Weinheim, Germany

Dinuclear Copper Complex with a Pyridazine-Bridged Octadentate Ligand: Monooxygenase Activity and Characterization of Copper-Oxygen Intermediates

Alexander Stüber, Ramona Jurgeleit, Benjamin Grimm-Lebsanft, Sören Buchenau, Ina Kellner, Yannik Appiarius, Christian Näther, Jan Krahmer, Ivana Ivanović-Burmazović, Michael Rübhausen, Maria A. Naumova and Felix Tuczek*

Table of Contents

1. Instrumentation and Physical Methods 4
- 1.1. General Information
- 1.2. Single Crystal Structure Determination
- 1.3. X-ray powder diffraction (XRD)
- 1.4. Resonance Raman Spectroscopy
- 1.5. Cryo-UHR-ESI Mass Spectrometry
- 1.6. X-ray Absorption Spectroscopy (XAS)
- 1.7. GC-MS
- 1.8. HR-EI Mass Spectrometry
- 1.9. HR-ESI Mass Spectrometry
- 1.10. Electron Paramagnetic Resonance Spectroscopy (EPR)
- 1.11. Computational Details

2. Experimental Procedures 10
- 2.1. Preparation of the precursors: General remarks
- 2.2. Preparation of the octadentate ligand **MO8**
  - 2.2.1. Step 1: Synthesis of pzea
  - 2.2.2. Step 2 and 3: Preparation of MePydz
  - 2.2.3. Step 4:Synthesis of BCMPydz
  - 2.2.4. Step 5: Connecting the building blocks
- 2.3. Preparation of the copper(I) complexes
  - 2.3.1. General procedure
  - 2.3.2. Synthesis of **2**-PF_6
  - 2.3.3. Synthesis of **2**-OTf
  - 2.3.4. Synthesis of **3**-OTf
  - 2.3.5. Synthesis of **3**-PF_6
- 2.4. Synthesis of oxygen-atom transfer reagents
  - 2.4.1. Synthesis of iodosobenzene (PhIO)
  - 2.4.2. Synthesis of dimethyldioxirane (DMDO)

3. Additional Information about the Crystal Structures of 2-PF_6 and 3-PF_6 24

4. X-ray powder diffraction 26
- 4.1. Diffractograms of the **Cu_2OH** complexes **3**-PF_6 and **3**-OTf

5. UV/Vis Spectroscopy 27
- 5.1. Overview of the obtained results with O_2, PhIO, N_2O, DMDO and AgOTf/$AgPF_6$
- 5.2. Additional UV/Vis spectra for the **Cu_2OH**
- 5.3. Additional UV/Vis spectra for the **Cu_2OOH** complex
- 5.4. Additional UV/Vis spectra for the green species

6. Resonance Raman Spectroscopy 34
- 6.1. Spectroscopic data and calculated vibrations
- 6.2. Theoretical data derived from DFT
- 6.3. Additional resonance Raman spectra
  - 6.3.1. Green species: Measurements at room temperature (293 K) using O_2
  - 6.3.2. Green species: Measurements at +35 °C (308 K) using O_2

7. IR-Spectroscopy 36
- 7.1. Theoretical data for the **Cu_2OH** complex derived from DFT
- 7.2. IR spectrum of **2**-OTf and **3**-OTf
- 7.3. IR spectrum of **3**-PF_6
- 7.4. IR spectrum of the green species and possible structure

8. HR-ESI Mass Spectrometry 45
- 8.1. Mass spectrum of the **Cu_2OH** complex **3**-OTf
- 8.2. Mass spectrum of the **Cu_2OH** complex **3**-PF_6

9. Cryo-UHR-ESI Mass Spectrometry 60
- 9.1. Obtained mass spectra upon reaction with $^{16}O_2$ at -90 °C
- 9.2. Obtained mass spectra upon reaction with $^{16}O_2$ and $^{18}O_2$ at room temperature
- 9.3. Obtained mass spectra upon reaction with PhIO
- 9.4. Obtained mass spectra upon reaction with N_2O
- 9.5. Obtained mass spectra upon reaction with DMDO
- 9.6. Obtained mass spectra upon reaction with $^{16}O_2$ at room temperature and at 308 K

9.7. Ligand fragments as decomposition species upon reaction with O_2 and DMDO

10. Electron Paramagnetic Resonance……71
10.1. EPR spectrum of **2**-PF_6, **3**-PF_6 and the green species

11. X-ray Absorption Spectroscopy…… 72
11.1. Precursor **2**-PF_6
11.2. Reaction of **2**-PF_6 with oxygen
11.3. Reaction of **2**-PF_6 with DMDO
11.4. **Cu_2OH** complex **3**-OTf and reaction with DBU and [Lut-H][OTf]

12. Additional DFT Calculations…… 84
12.1. **Cu_2O**, **Cu_2OH** and **Cu_2OOH**: Geometry optimization, selected bond lengths and angles
12.2. Comparison with the crystal structures **2**-PF_6 and **3**-PF_6
12.3. Geometry optimization, selected bond lengths and angles of a μ-1,2 peroxo complex

13. Reactivity of the Model Complex …… 92
13.1. Reactivity of the green species at rt: General Procedure
13.2. Reactivity of the green species with Anthrone (AT)
13.3. Reactivity of the **Cu_2OOH** complex: General Procedure
13.4. Reactivity of the **Cu_2OOH** complex with Anthrone (AT)
13.5. Reactivity of the **Cu_2OH** complex with Anthrone (AT)

14. Appendix ……97
14.1. Data Availability
14.2. Author Contributions

15. References……98

Part I

1. Instrumentation and Physical Methods

1.1. General Information

Syntheses with oxygen- and moisture-sensitive substances were carried out with dried solvents (vide infra) using Schlenk technique (N_2 or argon atmosphere) and a glovebox (O_2 < 1 ppm and H_2O < 1 ppm) under N_2 atmosphere. The solvents were dried (acetone with Drierite™; acetonitrile with CaH_2; ethanol with NaOEt; methanol with magnesium) and freshly distilled under argon or N_2 before use. Anhydrous deuterated solvents were degassed by freeze-pump-thaw and dried over molecular sieves (3 Å). Chemicals and solvents were purchased from Sigma-Aldrich, Merck, abcr, Deutero, Fisher Scientific and BLDPharm in reagent grade and used without further purification.

Flash Column chromatography was performed with an Isolera One Spectra by co. Biotage with an UV detector (λ = 200-400 nm) and prepacked SNAP Ultra cartridges (25 g and 50 g). The R_f-values were determined by thin-layer chromatography using Polygram Sil G/UV254 TLC polyester sheets with silica gel 60 and fluorescent indicator (Macherey-Nagel, 0.2 mm particle size) and an UV lamp (λ = 254 nm) by co. Camag. Elemental analysis were carried out with a vario MICRO elemental analyzer from Elementar Analysensysteme GmbH; the samples were prepared in tin vessels and burnt in an oxygen stream. NMR-spectroscopic measurements were performed at 300 K with a Bruker DRX 500 (^{1}H NMR (500.1 MHz), ^{13}C NMR (125.8 MHz)) and a Bruker AVANCE III HD Pulse Fourier Transform spectrometer. Frequencies of 400.13 MHz (^{1}H NMR) and 100.62 MHz (^{13}C NMR) were used with TMS as internal standard. Infrared spectra were recorded on a Bruker ALPHA FT-IR Spectrum with a Platinum ATR setup. Solutions were measured using a Bruker Vertex 70 MIR/FIR spectrometer (spectral range of 6000-80 cm^{-1}), that was equipped with a broadband spectral range extension. Oxygen and moisture-sensitive compounds were dissolved and transferred into an inert cell for the measurements. UV/Vis measurements at room temperature were performed with an Agilent 8453 spectrometer and a quartz cuvette (l = 0.2 cm or 1.0 cm) was used. For low temperature UV/Vis measurements an Agilent Cary 5000 spectrophotometer was used with a CryoCAV KONTI cryostat and a quartz cuvette (l = 1 cm). The temperature is adjusted with the CryoVac temperature measurement control unit TIC304-MA (enables gas flow control of the liquid nitrogen in combination with the CryoVac L-2033 gas lances).

1.2. Single Crystal Structure Determination

Data collection was performed with a XtaLAB Synergy, Dualflex, HyPix diffractometer using Cu-Kα radiation (λ = 1.54184 Å). The structure was solved with SHELXT[1] using Intrinsic Phasing and refined with SHELXL[2] using Least Squares minimisation. All non-hydrogen atoms were refined anisotropic. The C-H hydrogen atoms were positioned with idealized geometry and were refined isotropically with U_{iso} (H) = 1.2 U_{eq} (C).

Selected crystal data are given in Table S1 and the ORTEP plots can be found in Figure S1 and Figure S2. Selected bond lengths and angles are given in Table S2 and Table S3.

CCDC-2149824 (compound **2**-PF_6) and CCDC-2419891 (**3**-PF_6) contain the supplementary crystallographic data for this paper. These data can be obtained free of charge from the Cambridge Crystallographic Data Centre via http://www.ccdc.cam.ac.uk/data_request/cif.

Table S1: Selected crystal data and results of the structure refinement for compound **2**-PF_6 and **3**-PF_6.

Compound	**2**-PF_6	**3**-PF_6
Empirical formula	$C_{26}H_{34}Cu_2F_{12}N_{12}P_2$	$C_{26}H_{35}Cu_2F_{18}N_{12}OP_3$
Formula weight	931.67	1093.65
Temperature/K	100(2)	100(2)
Crystal system	monoclinic	orthorhombic
Space group	$P2_1/n$	*Pbcn*
a/Å	13.71360(10)	17.39692(8)
b/Å	14.26250(10)	13.61333(7)
c/Å	18.82440(10)	16.28654(7)
α/°	90	90
β/°	103.9850(10)	90
γ/°	90	90
Volume/Å^3	3572.73(4)	3857.14(3)
Z	4	4

ρ_{calc}g/cm^3	1.732	1.883
μ/mm^{-1}	3.259	3.763
2Θ range for data collection/°	7.21 to 159.682	8.25 to 160.53
Reflections collected	77451	42855
Independent reflections	7732	4174
R_{int}	0.0173	0.0223
Reflections with [I>=2σ (I)]	7650	4130
Parameters	487	284
Goodness-of-fit on F^2	1.066	1.061
Final R indexes [I>=2σ (I)]	R_1 = 0.0253, wR_2 = 0.0662	R_1 = 0.0301, wR_2 = 0.0799
Final R indexes [all data]	R_1 = 0.0254, wR_2 = 0.0664	R_1 = 0.0303, wR_2 = 0.0801
Largest diff. peak/hole / e Å^{-3}	0.37/-0.38	0.58/-0.41

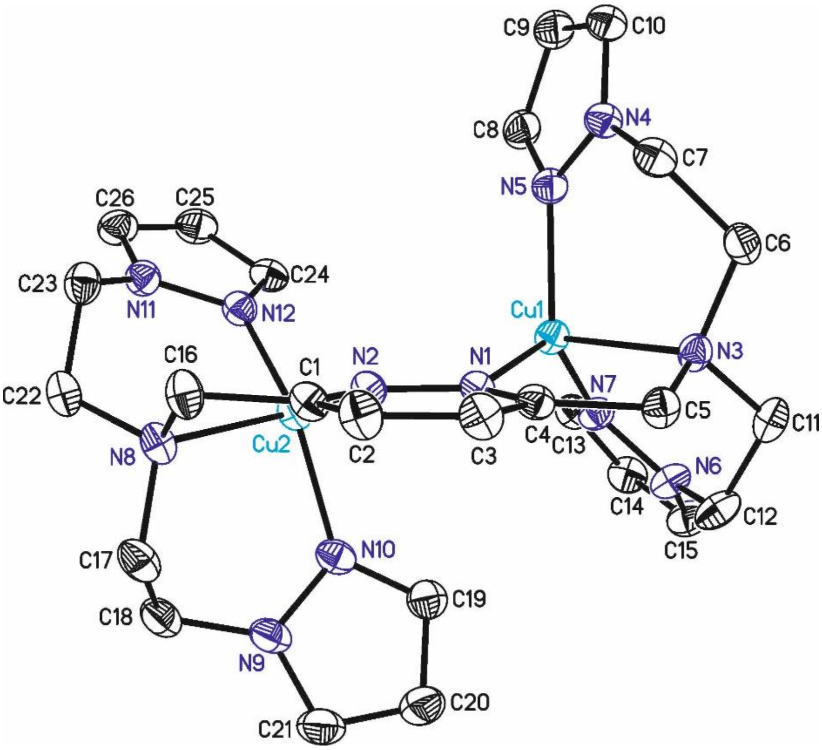

Figure S1: ORTEP plot of the cation in the crystal structure of compound **2**-PF_6 with labeling and displacement ellipsoids drawn at the 50 % probability level. For clarity the two crystallographically independent hexafluorophosphate anions and the hydrogen atoms are not shown.

Table S2: Selected bond lengths (Å) and angles (°) of compound **2**-PF_6.

Cu1	N7		1.9515(12)	Cu2	N12		1.9424(13)
Cu1	N5		1.9771(13)	Cu2	N10		2.0052(13)
Cu1	N1		1.9862(12)	Cu2	N2		2.0340(12)
Cu1	N3		2.2229(12)	Cu2	N8		2.2164(12)
N7	Cu1	N5	117.18(5)	N2	Cu2	N8	79.93(5)

N7	Cu1	N1	128.78(5)	N12	Cu2	N10	134.04(5)
N5	Cu1	N1	112.94(5)	N12	Cu2	N2	125.76(5)
N7	Cu1	N3	100.17(5)	N10	Cu2	N2	98.62(5)
N5	Cu1	N3	99.18(5)	N12	Cu2	N8	100.89(5)
N1	Cu1	N3	81.31(5)	N10	Cu2	N8	97.79(5)

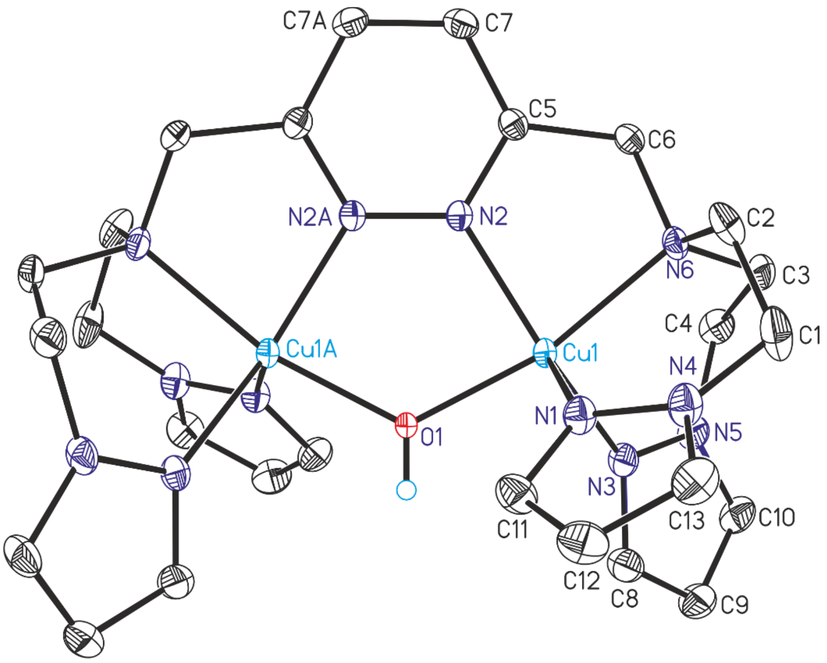

Figure S2: ORTEP plot of the cation in the crystal structure of compound **3**-PF_6 with labeling and displacement ellipsoids drawn at the 50 % probability level. For clarity the three crystallographically independent hexafluorophosphate anions and the hydrogen atoms (except from the OH group) are not shown.

Table S3: Selected bond lengths (Å) and angles (°) of compound **3**-PF_6. Symmetry codes for the generation of equivalent atoms: A: 1-x,+y,1/2-z.

Cu1	O1		1.9301(8)	Cu1	N3		1.9843(15)
Cu1	N1		2.1296(15)	Cu1	N6		2.0984(14)
Cu1	N2		1.9964(14)				
O1	Cu1	N1	89.38(4)	N3	Cu1	N1	99.09(6)
O1	Cu1	N2	85.84(6)	N3	Cu1	N2	149.49(6)
O1	Cu1	N3	97.89(6)	N3	Cu1	N6	93.34(6)
O1	Cu1	N6	167.46(6)	N6	Cu1	N1	94.34(6)
N2	Cu1	N1	111.26(6)	Cu1	O1	Cu1A	124.74(9)
N2	Cu1	N6	81.65(6)				

1.3. X-ray powder diffraction (XRD)

X-ray powder diffraction measurements were performed with an Empyrean diffractometer from Malvern Panalytical with Cu-Kα-radiation (λ = 1.5406). The samples were prepared in a glovebox under N_2 atmosphere. Air and moisture sensitive samples were sealed in Kapton tape.

1.4. Resonance Raman Spectroscopy

For Raman measurements, the custom-built UT-3 Raman spectrometer was used,[3] which was combined with a frequency doubled Ti:sapphire laser (Tsunami model 3960C-15HP, Spectra Physics Lasers Inc.) to obtain an excitation wavelength of 397 nm with a pulse width of 1.8 ps. A 1.4 mL screw cap Suprasil cuvette with septum (117104F-10-40, Hellma Analytics) was used for oxygenation with the previously described cryostat.[4] The cuvette was equipped with a Peltier element (QC-127-1.4-6.0MS, QuickCool) and a cooling copper block which covered three sides of the cuvette. The laser beam was widened with a spatial filter and then focused on the cuvette inside the cryostat with a focus spot size of 20 µm in diameter. The focus was positioned 30 µm inside the cuvette with a micrometer screw. With the entrance optics of the UT-3 triple monochromator spectrometer, Raman scattered light was captured.

A 12.5 mM solution of the precursor in acetone was cooled below -90 °C in the cuvette cryostat. Dioxygen was added *via* a cannula through the septum (0.02 bar overpressure for 15 min) resulting in a color change from red to an intense darker red. With a Pt100 sensor, the temperature of the solution inside the cuvette was measured. For the measurements above 193 K, the ethanol bath was warmed up and the cooling power of the Peltier element was adjusted accordingly, to reach the respective temperatures. The used laser power in front of the entrance optics was between 10 to 15 mW and data was accumulated up to 900 s. The oxygenation at room temperature was performed in a flask under a fume hood, resulting in a color change of the solution from red to green. After oxygenation, the solution was transferred into the cuvette.

1.5. Cryo-UHR-ESI Mass Spectrometry

Cryospray-ionization mass spectrometry (CSI-MS) measurements were performed using an UHR-TOF Bruker Daltonik maXis plus, an ESI-quadrupole time-of-flight (qToF) mass spectrometer capable of a resolution of at least 60000 (FWHM), which was coupled to a Bruker Daltonik Cryospray unit.
Detection was in positive ion mode, the source voltage was 3.5 kV. The flow rates were 240 µL/hour. The drying gas (N_2), to achieve solvent removal, was held at -40 °C (or at -90 °C) and the spray gas was held at -40 °C (or at -90 °C).
The machine was calibrated prior to every experiment *via* direct infusion of Agilent ESI-TOF low concentration tuning mixture, which provided an *m/z* range of singly charged peaks up to 2700 Da in both ion modes.
Analyzing of the measured spectra was performed with Bruker Compass DataAnalysis 5.2.

General sample preparation for CSI-MS:
In an argon atmosphere glove box Cu(I) complex (1.2 mg) was dissolved in dry acetone (1.0 mL). The complex solution was cooled to -10 °C (or to -80 °C or -90 °C) or heated to 35 °C and for room temperature reactions left as they were.

Oxygenation of Cu(I) complexes:
The cooled/heated/room temperature Cu(I) solutions were bubbled with excess $^{16}O_2$ (or $^{18}O_2$). After bubbling, the samples were directly injected into the mass spectrometer. In case of the reactions at room temperature and at 35 °C the samples were injected after a reaction time of 3 h.

Reaction of Cu(I) complexes with iodosobenzene:
In an argon atmosphere glovebox iodosobenzene (50 mg) was suspended in dry acetone (3 mL). The suspension was supersonicated for better solving of iodosobenzene. Iodosobenzene solution (500 µL) was added to the cooled Cu(I) solutions. The reaction solutions were constantly held at -10 °C for 3 h of reaction time, diluted with cooled, dry acetone and then injected into the mass spectrometer.

Reaction of Cu(I) complexes with N_2O:
The Cu(I) solutions were bubbled with excess N_2O at room temperature. After bubbling, the samples were directly injected into the mass spectrometer.

Reaction of Cu(I) complexes with DMDO:

After cooling the Cu(I) solutions to -80 °C, 1.5 eq. (or 3 eq.) of a DMDO solution in acetone was added. After 1 minute reaction time at -80 °C, the solutions were diluted with cooled dry acetone and injected directly into the mass spectrometer.

1.6. X-ray Absorption Spectroscopy (XAS)

Experimental:
Cu K-edge XAS spectra were measured at beamline P65 of the PETRA III synchrotron light source at Deutsches Elektronen Synchrotron (DESY) in Hamburg.[5] A Si(111) double-crystal monochromator was used for energy selection, providing a flux of ~10^{12} ph/s. Spectra were measured in transmission mode. The X-ray beam was cut with the slits to the size of 1.7 mm (horizontally) by 0.3 mm (vertically). Under inert conditions (oxygen and water free) the precursor **2**-PF_6 was prepared and transferred into a custom-made PEEK cuvette with septum and two Kapton® windows, which were sealed by a Teflon O-ring. The cuvette was placed into a closed-cycle He cryostat. For measurements the temperature was set to be in the 183-203 K range. The energy was scanned from -200 eV to 1200 eV around the Cu K-edge. Several scans were done to increase the signal-to-noise ratio. The precursor did not show signs of radiation damage. For some of the oxidized samples, the edge position in the consecutive scans was shifting towards low energies. In this case, spectra were taken on several fresh spots and averaged. The copper foil was measured concomitantly, and the energy position of the maximum of the first derivative of its absorption coefficient was set to 8979.0 eV. Accordingly, all measurements were calibrated to this value. Oxidation of **2**-PF_6 (~20 mM solution in acetone) was performed with two different agents: 1) by bubbling oxygen and 2) *via* the addition of DMDO solution in acetone. More detailed information about the oxidation process is given in section S7
Data reduction and Analysis:
Data was processed using Athena[6] and XAESA[7] software packages. Modeling of EXAFS was done using Larch[8] and EvAX[9] software packages.

1.7. GC-MS

GC-MS investigations were performed on an Agilent 7890B GC System with an Agilent 5977A MSD mass spectrometer. As column we used a HP-5M5 (5%-phenyl)-methylpolysiloxane (29.5 m x 250 µm x 0.25 µm). The inlet temperature was 250 °C at 9.3 psi. We used a temperature program which started with keeping the temperature at 45 °C for 30 s, heated to 175 °C over the course of 3.75 min (40 °C/min), then heated to 250 °C over the course of 22.5 min (4 °C/min) and maintained at 250 °C for 5 min (9.3 psi, 1.2 mL/min). As internal standard we used mesitylene (t_R = 3.13 min).

1.8. HR-EI Mass Spectrometry

HR-EI MS measurements were carried out with an AccuTOF^{TM} GCv 4G time-of-flight mass spectrometer from JOEL capable of a resolution of 80000. A potential of 70 V was applied (in the positive ion mode EI+) to accelerate electrons to a kinetic energy of 70 eV to produce positive ions. For the measurements, solid samples dichloromethane solutions were used and directly injected.

1.9. HR-ESI Mass Spectrometry

HR-ESI MS measurements were performed using a Q Exactive^{TM} Plus Orbitrap from Thermo Fisher Scientific with an electrospray ionization (ESI). The samples were dissolved in dichloromethane, acetone, methanol or acetonitrile and directly injected for the measurement.

1.10. Electron Paramagnetic Resonance Spectroscopy (EPR)

EPR spectroscopic measurements were performed with the EMXplus spectrometer from Bruker using a PremiumX microwave bridge and a Bruker ER-4116DM dual-mode cavity. X-band microwave radiation with a frequency of 9.86 GHz was used for the measurements.

1.11. Computational Details

All calculations were performed with ORCA 4.2.1.[10] For geometry optimizations and frequency calculations the PBE[11] functional and the def2-SVP[12] basis set were used in conjunctions with D3BJ[13] dispersion correction and the RI[14] approximation with the appropriate fitting basis set. [15] None of the optimized geometries showed imaginary frequencies.
Single point energies were computed using the B3LYP[16] functional, the def2-TZVPP[12] basis set, the CPCM[17] continuum solvation model with the appropriate setting for the respective solvent and the RIJCOSX[18] approximation for calculation speedup.
All systems with two unpaired electrons were treated with the broken symmetry approach.

Part II: Experimental Procedures

2. Experimental Procedures

2.1. Preparation of the precursors: General remarks

The multidentate *N*-donor ligand **MO8** was synthesized following the procedures described in Section S2.2. Addition of two equiv. of tetrakis(acetonitrile)copper(I)-hexafluorophosphate ($[Cu(NCMe)_4]PF_6$) or -triflate ($[Cu(NCMe)_4]OTf$) to a solution of **MO8** in acetonitrile provided the dinuclear copper(I) complexes **2** in excellent yields (89-94 %).[19] **2** shows distinct sensitivity against moisture and dioxygen. Slow evaporation of an acetone solution of **2**-PF_6 yielded red crystals suitable for investigation by single-crystal X-ray diffraction analysis (see below).

2.2. Preparation of the octadentate ligand MO8

2.2.1.Step 1: Synthesis of pzea

The known pzea was synthesized according to literature [20] The published procedure was slightly modified. 110 ml dry DMF was added to 4.40 g (110 mmol) 60% sodium hydride in mineral oil under N_2-atmosphere. 5.00 g (73.4 mmol) 1*H*-pyrazole was slowly added at 0 °C. The grey suspension was stirred for 2 h at room temperature. Then 6.56 g (36.7 mmol) bis(2-chloroethyl)amine hydrochloride was added in portions and the resulting yellow suspension was stirred at 60 °C for 3 d. The solvent was removed at 70 °C i. vac. Afterwards 100 ml brine and 25 ml water were added to the residue. The aqueous phase was extracted with 6 x 50 ml toluene. After removing the solvent i. vac, the residue was dissolved in 40 mL methanol and washed with *n*-hexane 2 x 40 ml. The removal if the methanol i. vac. resulted in a yellow liquid with traces of a colourless crystalline solid. The crude product was purified by column chromatography on silica gel (dichloromethane/methanol) to obtain 2.48 g (12.1 mmol, 33 %) of pzea as a light yellow liquid.

^{1}H NMR (200 MHz, $CDCl_3$, 300 K): δ = 7.48 (dd, J = 1.8 Hz, J = 0.5 Hz, 2H, Pz-CH), 7.35 (dd, J = 2.3 Hz, 0.5 Hz, 2H, Pz-CH), 6.21 (t, J = 2.1 Hz, 2H, Pz-CH),4.27-4.08 (m, 4H, CH_2), 3.12-2.92 (m, 4H, CH_2) ppm; ^{13}C NMR (126 MHz, $CDCl_3$, 300 K): δ = 139.7 (Pz-CH), 129.8 (Pz-CH), 105.5 (Pz-CH), 52.0 (CH_2), 49.1 (CH_2) ppm; IR (neat): $\tilde{\nu}$ = 3307 (w), 3107 (w), 2942 (w), 2843 (w), 1513 (m), 1443 (m), 1397 (s), 1279 (m), 1130 (w), 1089 (s), 1045 (m), 967 (m), 918 (m), 747 (s), 652 (m), 618 (s) cm^{-1}; HRMS (EI) *m/z* calcd. for $C_{10}H_{16}N_5$: 206.14057 [*M*+H]$^+$; found: 206.14062; Elemental analysis calcd. for $C_{10}H_{15}N_5$: C 58.51, H 7.37, N 34.12, found: C 57.00, H 7.10, N 32.98.

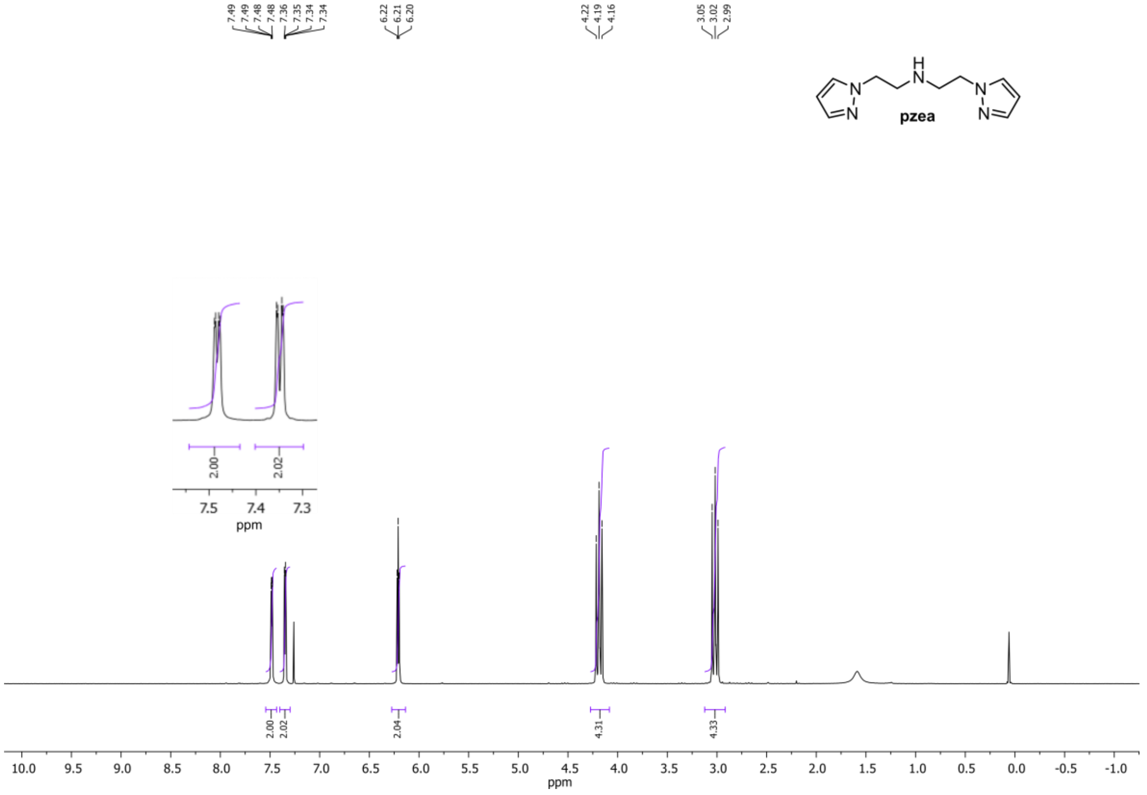

Figure S3: ^{1}H-NMR-spectrum of pzea.

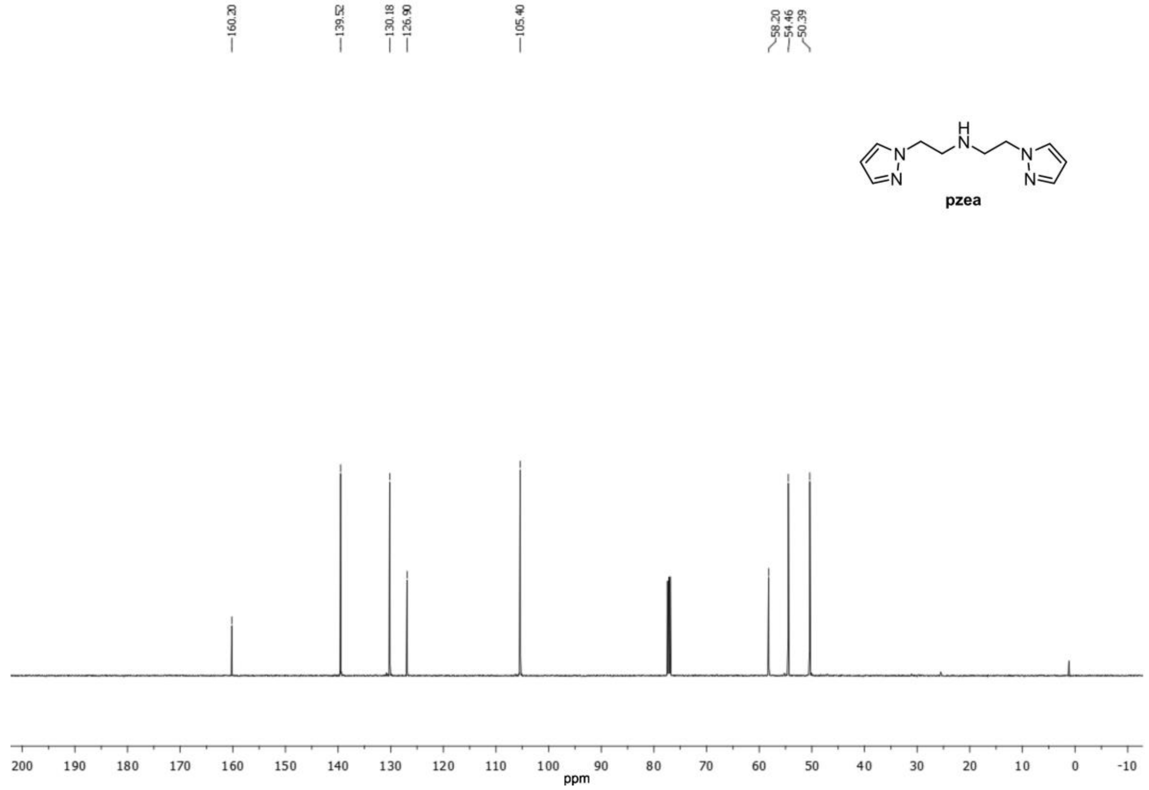

Figure S4: ^{13}C-NMR-spectrum of pzea.

2.2.2. Step 2 and 3: Preparation of MePydz

MePydz was synthesized according to literature. [21] The published procedure was slightly modified. To a solution of 6.0 mL (51.1 mmol) 2,5-hexanedione in 60 mL dry EtOH were slowly added 2.9 mL (51.1 mmol) of hydrazine hydrate (50-60 %). The reaction mixture was stirred at 90 °C for 3 h. After cooling to room temperature the solvent was removed i. vac. The light yellow residue was dissolved in 10 mL dry benzene and then added to a suspension of 1.14 g (10.3 mmol) Pd/C in 140 mL dry benzene. Afterwards the reaction mixture was stirred for 14 h at 90 °C. The suspension was cooled down to room temperature, filtered over celite and the solvent was removed i. vac. to obtain 5.10 g (47.2 mmol, 92 %) of MePydz as a yellow liquid which turned red after one day.

^{1}H NMR (500 MHz, $CDCl_3$, 300 K): δ = 7.20 (s, 2H, Pydz-CH), 2.66 (s, 6H, CH_3) ppm; ^{13}C NMR (126 MHz, $CDCl_3$, 300 K): δ = 157.8 (Pydz-C_q), 127.0 (Pydz-CH), 22.1 (CH_3) ppm; IR (neat): $\tilde{\nu}$ = 3398 (w), 3055 (w), 2964 (w), 2926 (w), 1712 (w), 1592 (m), 1553 (m), 1428 (s), 1377 (m), 1255 (m), 1077 (m), 832 (s), 742 (m) cm^{-1}; HRMS (EI) *m/z* calcd. for $C_6H_8N_2$: 108.06875 $[M]^+$; found: 108.06864; Elemental analysis calcd. for $C_6H_8N_2$: C 66.64, H 7.46, N 25.90, found: C 53.95, H 7.23, N 17.90.

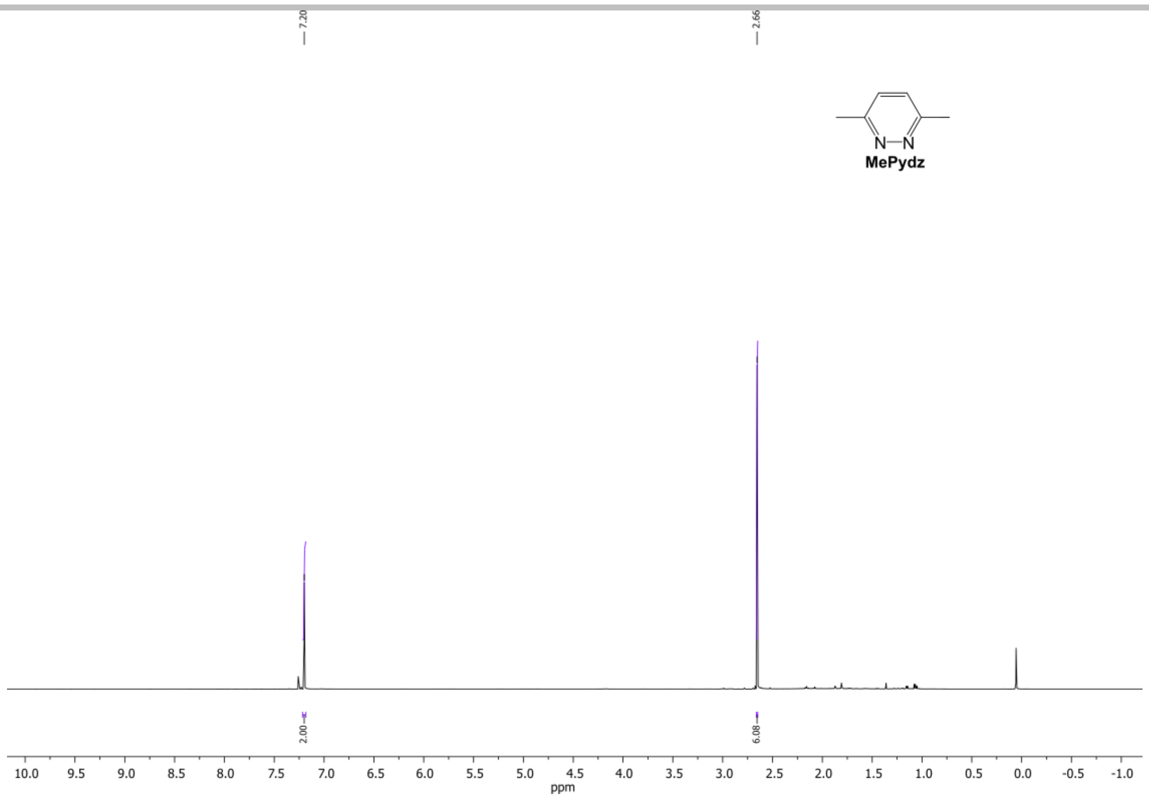

Figure S5: ^{1}H-NMR-spectrum of MePydz.

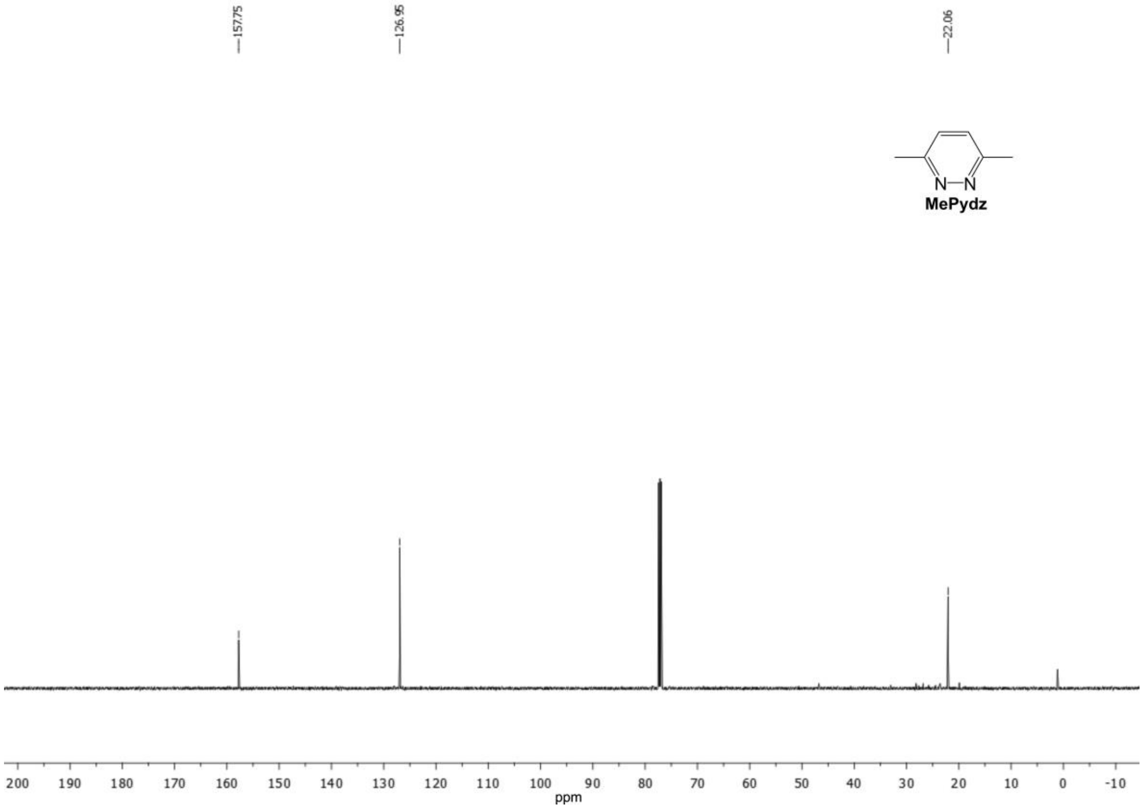

Figure S6: ^{13}C-NMR-spectrum of MePydz.

2.2.3.Step 4: Synthesis of BCMPydz

BCMPydz was synthesized according to literature.[22] MePydz 3.51 g (32.4 mmol) was dissolved in 370 mL chloroform and heated to 75 °C. Within 30 min 6.40 g (27.6 mmol) trichloroisocyanuric acid was added in portions. The reaction mixture was stirred for 2.5 h at 75 °C. After cooling to room temperature, the light brown suspension was filtered over hyflo-celite. The red-brown filtrate was washed 2 times with 100 mL of 0.2 M sodium hydroxide solution, twice with 125 mL water, once with 100 mL 0.2 M hydrochloric acid solution and again with 125 mL water. Then the organic phase was dried over sodium sulphate and filtered. The solvent was removed under ice cooling in vacuo. The resulting brown viscous substance was stored under nitrogen atmosphere at -32 °C until the next day to prevent decomposition. The crude product was purified by column chromatography on silica gel (acetone/petroleum ether) and the solvent was removed under ice cooling i. vac. to obtain a light yellow solid 1.82 g (10.3 mmol, 32 %). The product was stored under nitrogen atmosphere at -32 °C to prevent decomposition.

^{1}H NMR (500 MHz, $CDCl_3$, 300 K): δ = 7.77 (s, 2H, Pydz-CH), 4.91 (s, 4H, CH_2) ppm; ^{13}C NMR (126 MHz, $CDCl_3$, 300 K): δ = 159.1 (Pydz-C_q), 127.3 (Pydz-CH), 44.4 (CH_2) ppm; IR (neat): $\tilde{\nu}$ = 3069 (w), 3015 (w), 2966 (w), 1588 (w), 1555 (w), 1444 (m), 1435 (m), 1282 (m), 1134 (w), 1091 (w), 859 (m), 840 (m), 803 (m), 774 (s), 710 (s), 691 (m), 593 (w) cm^{-1}; HRMS (EI) *m/z* calcd. for $C_6H_6N_2Cl_2$: 175.99080 $[M]^+$; found: 175.99073; Elemental analysis calcd. for $C_6H_6N_2Cl_2$: C 40.71, H 3.42, N 15.82, found: C 40.45, H 3.73, N 15.70.

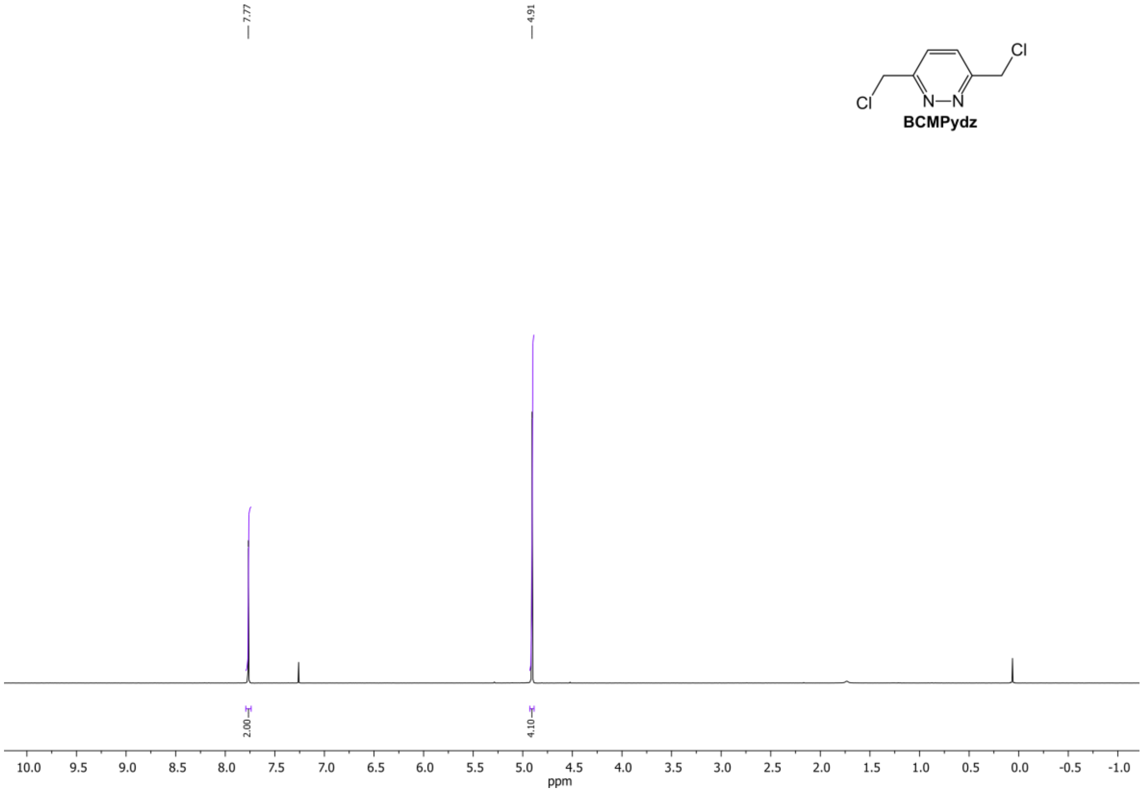

Figure S7: ^{1}H-NMR-spectrum of BCMPydz.

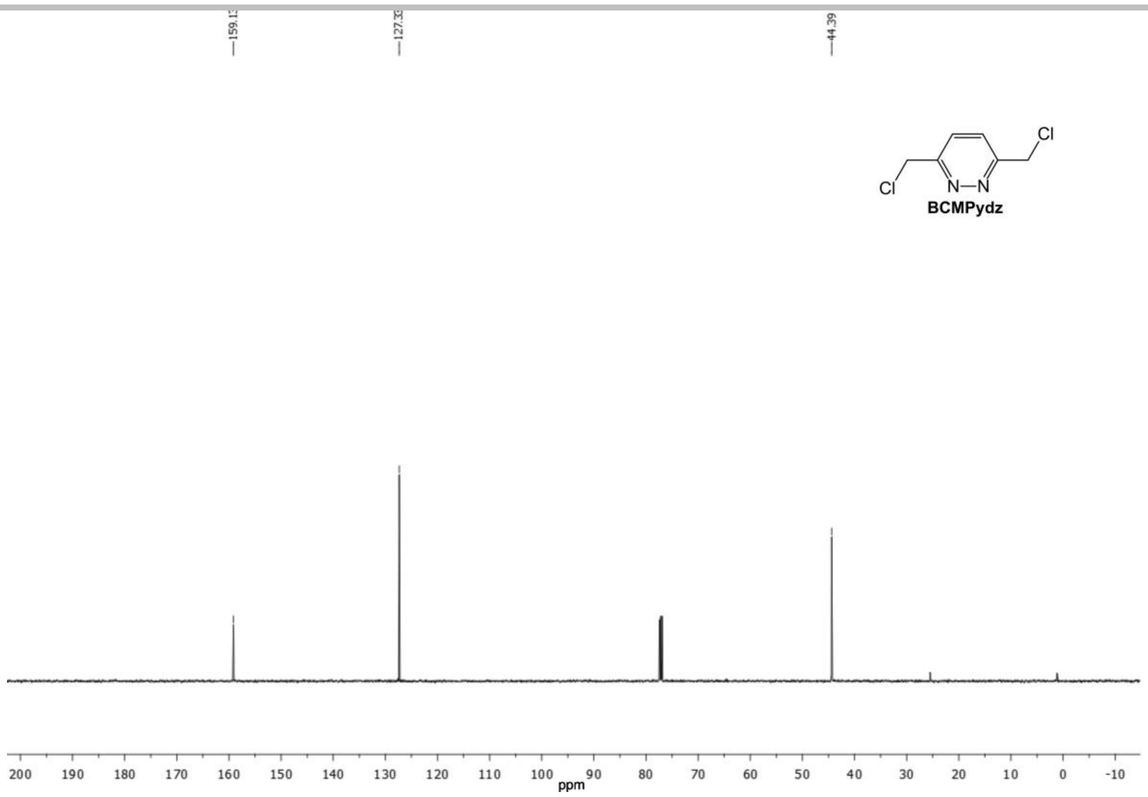

Figure S8: ^{13}C-NMR-spectrum of BCMPydz.

2.2.4. Step 5: Connecting the building blocks

The connection of pzea and BCMPydz to the ligand **MO8** was carried out after slight modification of the literature.[23] Under N_2-atmosphere, 1.14 g (5.55 mmol) of pzea was dissolved in 20 mL dry acetonitrile. 0.84 mL (8.31 mmol) dry triethylamine was added to the yellow solution. Subsequently, 455 mg (2.57 mmol) of BCMpydz was dissolved in 5 mL of dry acetonitrile, added to the pzea solution and a further 10 mL of dry acetonitrile was added. The brown solution was then heated for 3 d at 86 °C. After cooling to room temperature, the solvent was removed in vacuo and to the residue 30 mL water and 30 mL dichloromethane were added. The aqueous phase was extracted with 8 x 25 mL dichloromethane. Afterwards the organic phase was washed with 3 x 50 mL water, dried over sodium sulphate and filtered. The solvent was removed i. vac. and the crude product was purified by column chromatography on silica gel (dichloromethane/methanol) to obtain 176 mg (0.34 mmol, 13 %) of a brown viscous substance, which became solid after a short time.

^{1}H NMR (500 MHz, $CDCl_3$, 300 K): δ = 7.48 (d, J = 1.9 Hz, 4H, Pz-CH), 7.14 (d, J = 2.2 Hz, 4H, Pz-CH), 6.73 (s, 2H, Pydz-CH), 6.21 (t, J = 2.1 Hz, 4H, Pz-CH), 4.11 (t, J = 5.9 Hz, 8H, CH_2), 3.88 (s, 4H, CH_2), 2.99 (t, J = 5.9 Hz, 8H, CH_2) ppm; ^{13}C NMR (126 MHz, $CDCl_3$, 300 K): δ = 160.2 (Pydz-C_q), 139.5 (Pz-CH), 130.2 (Pz-CH), 126.9 (Pydz-CH), 105.4 (Pz-CH), 58.2 (CH_2), 54.5 (CH_2), 50.4 (CH_2) ppm; IR (neat): $\tilde{\nu}$ = 3119 (w), 3086 (w), 2939 (w), 2860 (w), 2832 (w), 2809 (w), 1512 (m), 1398 (s), 1357 (w), 1282 (m), 1183 (w), 1131 (w), 1091 (m), 1046 (m), 967 (m), 918 (w), 881 (w), 756 (s), 652 (m), 618 (s) cm^{-1}; HRMS (ESI) *m/z* calcd. for $C_{26}H_{35}N_{12}$: 515.31022 [*M*+H]$^+$; found: 515.30995 Elemental analysis calcd. for $C_{26}H_{34}N_{12}$: C 60.68, H 6.66, N 32.66, found: C 61.07, H 7.62, N 31.97.

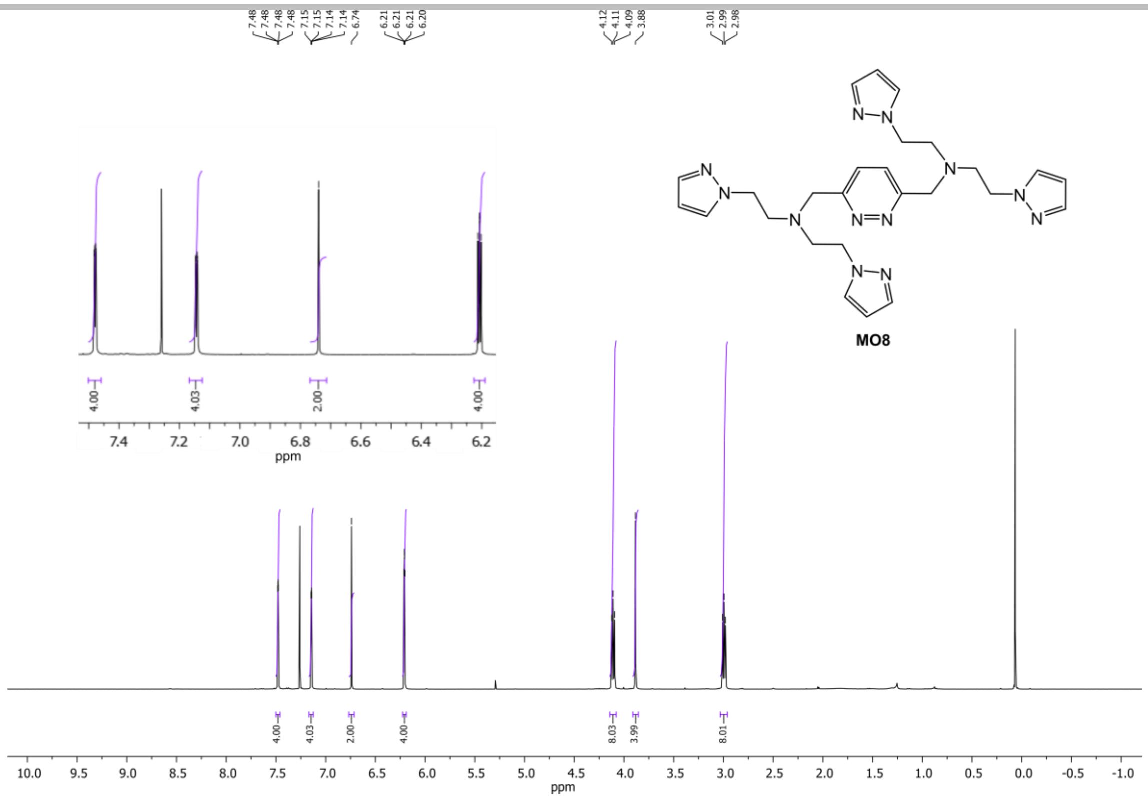

Figure S9: ^{1}H-NMR-spectrum of **MO8**.

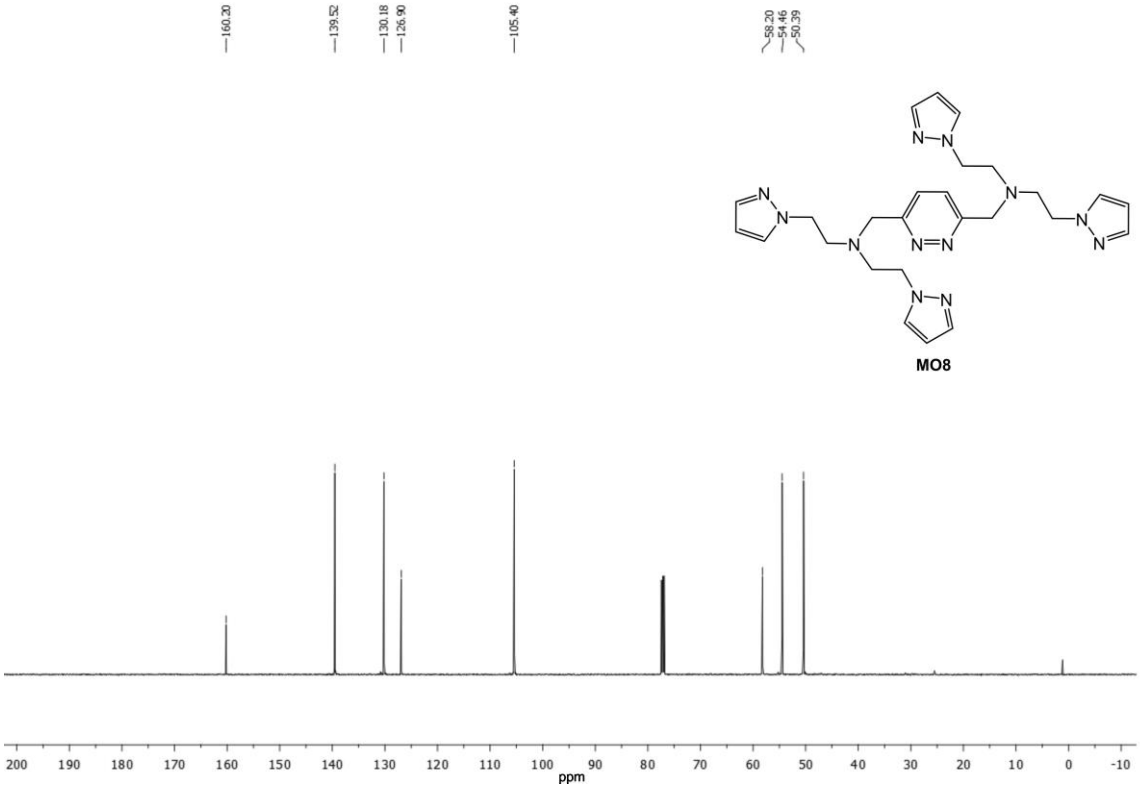

Figure S10: ^{13}C-NMR-spectrum of **MO8**.

2.3. Preparation of the copper(I) complexes

2.3.1. General Procedure

To a colorless solution of tetrakis(acetonitrile)copper(I)-hexafluorophosphate [Cu(NCMe)$_4$]PF$_6$ or -triflate [Cu(NCMe)$_4$]OTf (2 eq.) in MeCN (4 mL) was added a solution of **MO8** (1 eq.) in MeCN (4 mL) to generate a homogenous red solution. The solution was stirred for 30 min at room temperature. After removing the solvent under reduced pressure **2**-PF$_6$ or **2**-OTf were obtained as red microcrystalline solids (89-94 %).

2.3.2. Synthesis of [Cu$_2$(MO8)](PF$_6$)$_2$ (2-PF$_6$)

In the same manner as described above, **2**-PF$_6$ was obtained as a red microcrystalline solid.

To a colorless solution of 228 mg (611 µmol) tetrakis(acetonitrile)copper(I)-hexafluorophosphate dissolved in 4 mL acetonitrile was added a solution of 157 mg (306 µmol) **MO8** in 4 mL acetonitrile. The resulting red solution was stirred for 30 min at room temperature. After removing the solvent under reduced pressure 267 mg (287 µmol, 94 %) **2**-PF$_6$ was obtained as a red microcrystalline solid.

^{1}H NMR (400 MHz, CD$_3$CN, 300 K): δ = 7.69 (dd, J = 2.1 Hz, J = 0.6 Hz 4H, Pz-CH), 7.66 (s, 2H, Pydz-CH), 7.62-7.59 (m, 4H, Pz-CH), 6.32 (t, J = 2.3 Hz, 4H, Pz-CH), 4.29 (s, 4H, CH$_2$), 4.23-4.14 (m, 8H, CH$_2$), 3.21-3.11 (m, 8H, CH$_2$) ppm; ^{13}C NMR (101 MHz, CD$_3$CN, 300 K): δ = 160.1 (Pydz-C$_q$), 141.4 (Pz-CH), 132.8 (Pz-CH), 129.3 (Pydz-CH), 106.6 (Pz-CH), 60.5 (CH$_2$), 59.6 (CH$_2$), 50.3 (CH$_2$) ppm; ^{19}F NMR (376 MHz, CD$_3$CN, 300 K): δ = -72.82 (d, J = 706.6 Hz, PF$_6$) ppm; ^{31}P NMR (162 MHz, CD$_3$CN, 300 K): δ = -143.34 (hept, J = 706.5 Hz, PF$_6$) ppm; IR (neat): 3150 (w), 3135 (w), 2960 (w), 2935 (w), 2854 (w), 1516 (w), 1436 (m), 1408 (m), 1300 (w), 1203 (w), 1186 (w), 1101 (m), 1066 (m), 825 (s), 757 (s), 615 (m), 556 (s) cm^{-1}; UV/Vis (acetone): λ_{max} (ε) = 383 nm (6053 M^{-1} cm^{-1}), 535 nm (482 M^{-1} cm^{-1}); HRMS (ESI) *m/z* calcd. for $C_{26}H_{34}N_{12}Cu_2PF_6$: 785.1258 [M-PF$_6$]$^+$; found: 785.1268; *m/z* calcd. for $C_{26}H_{34}N_{12}Cu_2$: 320.0805 [M-2PF$_6$]$^{2+}$; found: 320.0808; Elemental analysis calcd. for $C_{26}H_{34}N_{12}Cu_2P_2F_{12}$: C 33.52, H 3.68, N 18.04, found: C 33.50, H 3.78, N 17.88.

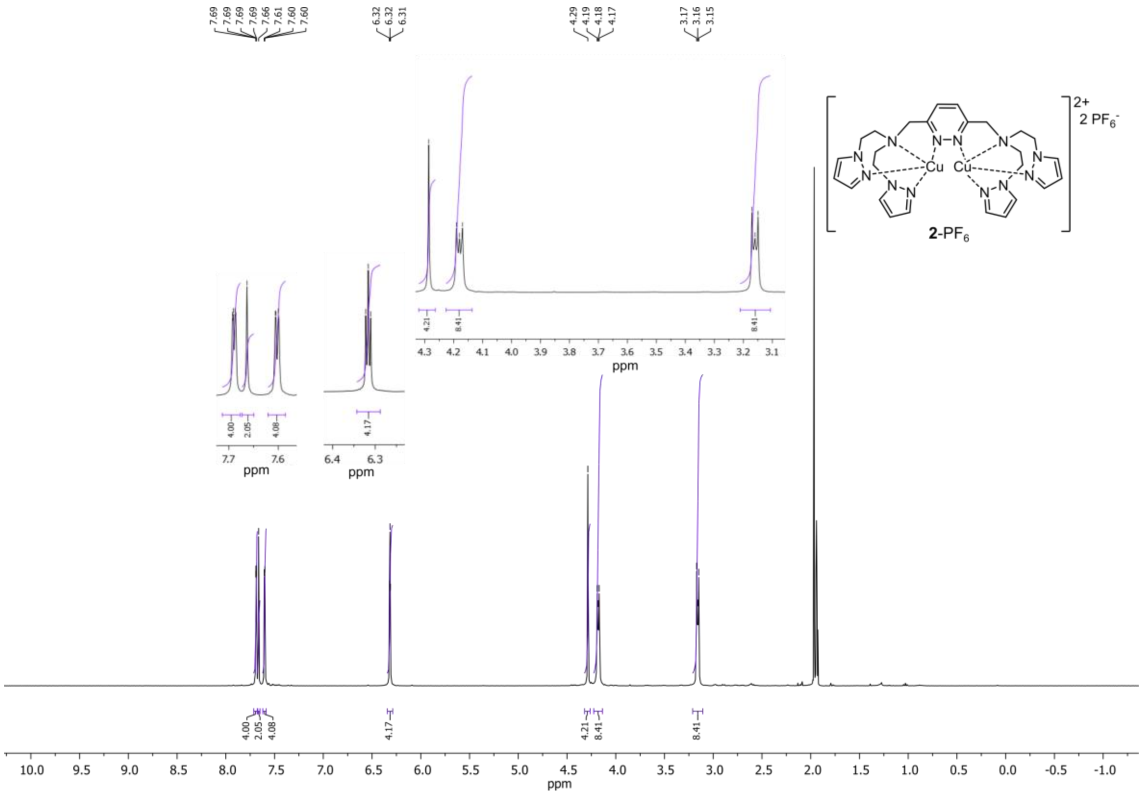

Figure S11: ^{1}H-NMR-spectrum of **2**-PF$_6$.

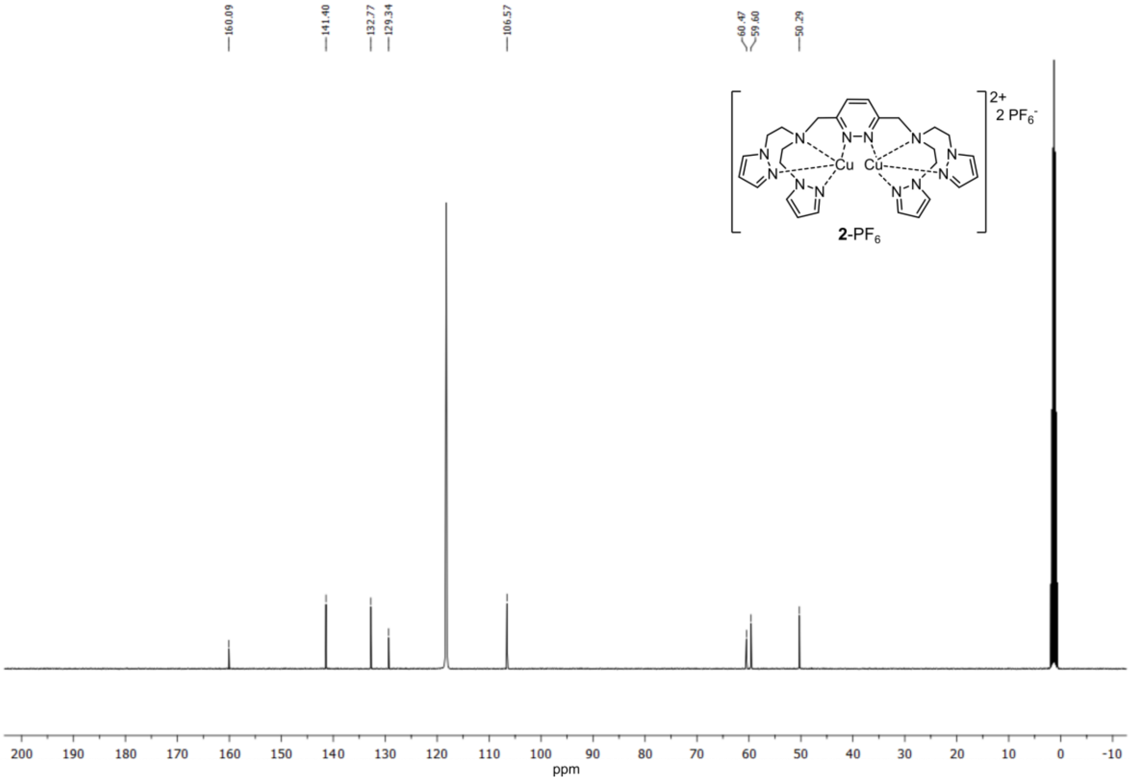

Figure S12: ^{13}C-NMR-spectrum of **2**-PF_6.

2.3.3.Synthesis of $[Cu_2(MO8)](OTf)_2$ (2-OTf)

In the same manner as described above, **2**-OTf was obtained as a red microcrystalline solid.

To a colorless solution of 211 mg (560 µmol) tetrakis(acetonitrile)copper(I)-trifluoromethanesulfonate dissolved in 4 mL acetonitrile was added a solution of 144 mg (280 µmol) **MO8** in 4 mL acetonitrile. The resulting red solution was stirred for 30 min at room temperature. After removing the solvent under reduced pressure 235 mg (250 µmol, 89 %) **2**-OTf was obtained as a red microcrystalline solid.

^{1}H NMR (400 MHz, CD_3CN, 300 K): δ = 7.70 (dd, J = 2.2 Hz, J = 0.7 Hz 4H, Pz-CH), 7.67 (s, 2H, Pydz-CH), 7.61 (dd, J = 2.5 Hz, J = 0.7 Hz, 4H, Pz-CH), 6.33-6.30 (m, 4H, Pz-CH), 4.30 (s, 4H, CH_2), 4.22-4.15 (m, 8H, CH_2), 3.20-3.12 (m, 8H, CH_2) ppm; ^{13}C NMR (101 MHz, CD_3CN, 300 K): δ = 160.1 (Pydz-C_q), 141.4 (Pz-CH), 132.8 (Pz-CH), 129.4 (Pydz-CH), 122.1 (q, $J_{C\text{-}F}$ = 320.8 Hz, CF_3), 106.6 (Pz-CH), 60.5 (CH_2), 59.6 (CH_2), 50.3 (CH_2) ppm; ^{19}F NMR (376 MHz, CD_3CN, 300 K): δ = -78.86 (s, CF_3) ppm; IR (neat): 3123 (w), 2958 (w), 2932 (w), 2854 (w), 1515 (w), 1436 (m), 1408 (m), 1366 (w), 1342 (w), 1275 (s), 1225 (m), 1157 (s), 1101 (m), 1065 (m), 1034 (s), 1001 (w), 950 (w), 939 (w), 921 (w), 897 (w), 868 (s), 848 (m), 831 (m), 817 (m), 802 (m), 789 (m), 781 (m), 770 (m), 754 (m), 743 (m), 733 (m), 725 (s), 716 (s), 704 (m), 690 (m), 675 (m), 663 (m), 654 (m), 646 (m), 623 (m), 608 (m), 598 (s), 582 (s), 573 (s), 565 (s), 552 (s), 546 (s), 527 (m) 507 (s), 500 (m), 484 (m), 473 (w), 465 (m), 457 (w), 448 (w), 438 (w) 422 (w), 411 (m), 390 (m), 374 (m) cm^{-1}; UV/Vis (acetone): λ_{max} (ε) = 382 nm (6066 M^{-1} cm^{-1}), 535 nm (460 M^{-1} cm^{-1}); HRMS (ESI) *m/z* calcd. for $C_{26}H_{34}N_{12}Cu_2$: 320.08052 $[M\text{-}2OTf]^{2+}$; found: 320.08084; Elemental analysis calcd. for $C_{28}H_{34}N_{12}Cu_2O_6S_2F_6$: C 35.78, H 3.65, N 17.88, found: C 33.50, H 3.71, N 17.81.

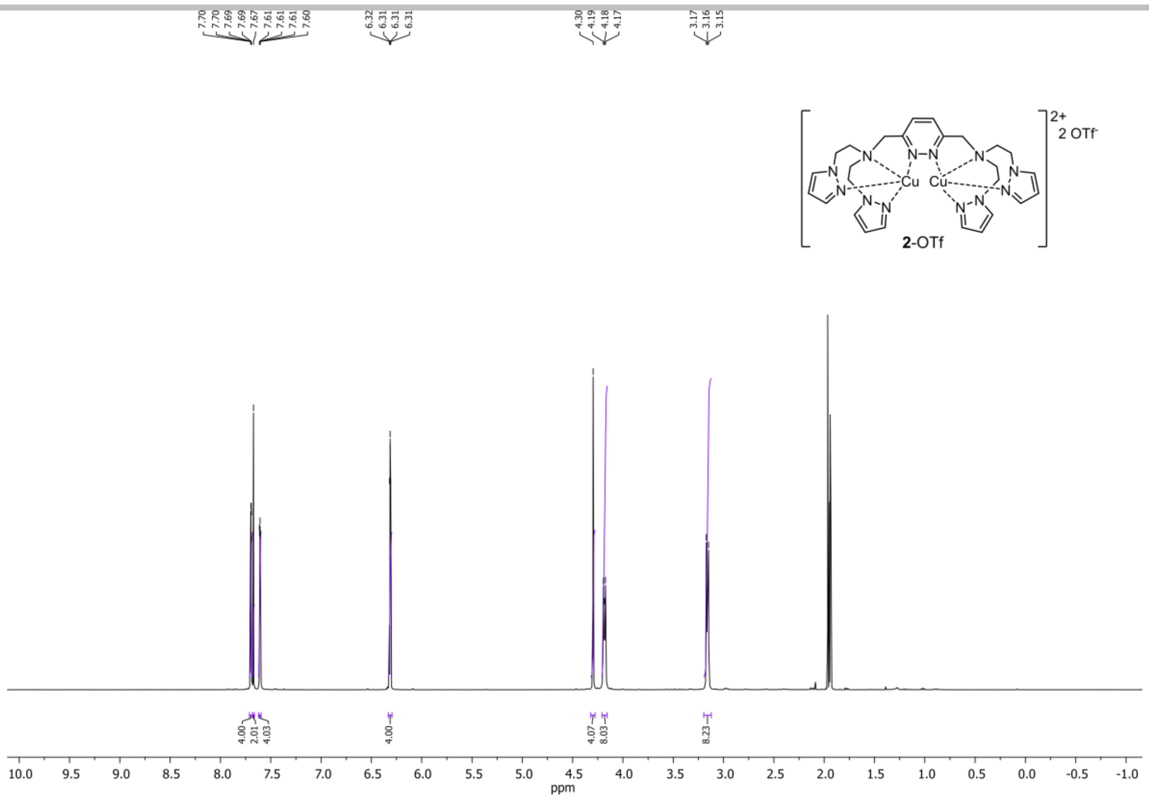

Figure S13: ^{1}H-NMR-spectrum of **2-**OTf.

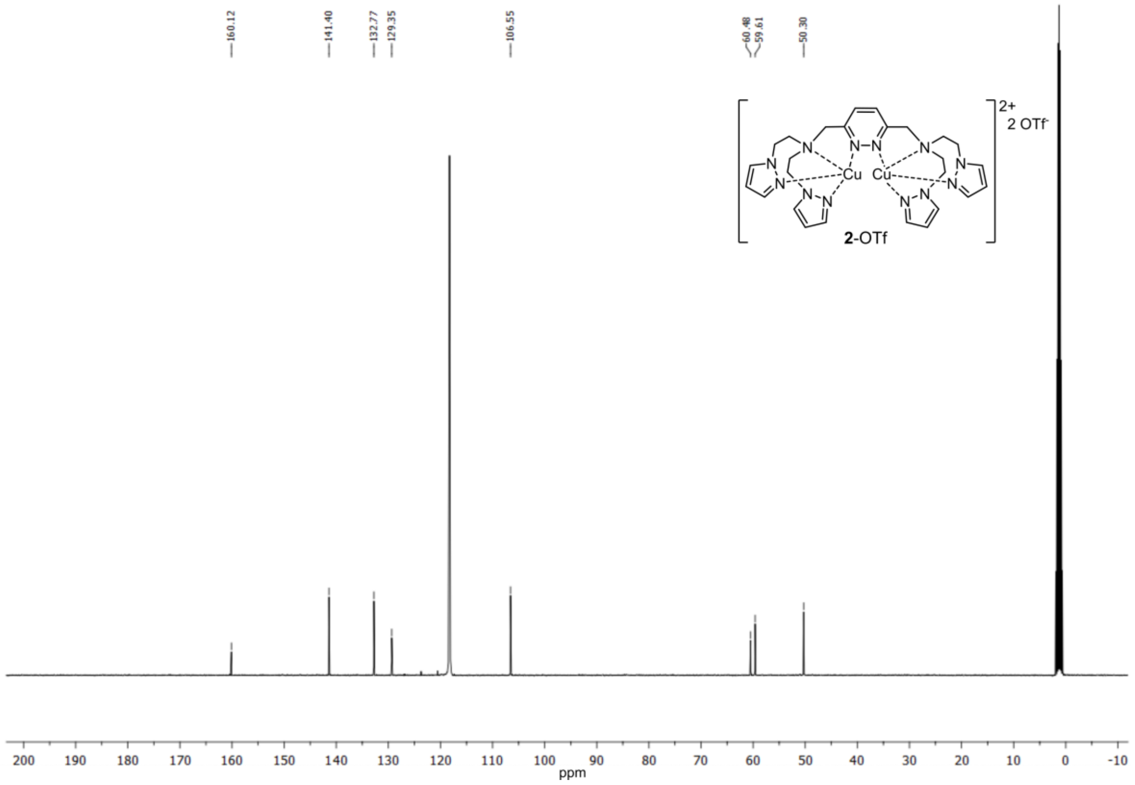

Figure S14: ^{13}C-NMR-spectrum of **2-**OTf.

2.3.4. Synthesis of [Cu_2(OH)(MO8)](OTf)$_3$ (3-OTf)

The synthesis of the [Cu_2(OH)(**MO8**)](OTf)$_3$ complex **3**-OTf was based on the literature of KUZELKA *et al*.[24] 99.9 mg (106 µmol) of the copper(I)-complex **2**-OTf were dissolved in 3 mL methanol. 55.9 mg (2. eq., 218 µmol) of AgOTf dissolved in 3 mL methanol was added to the red solution, resulting in a green suspension after a few seconds. After stirring for 30 min at room temperature, the formed elemental silver was filtered off using a syringe filter. The solvent of the dark green solution was removed under reduced pressure. 90 mg (81.4 µmol, 77 %) **3**-OTf was obtained as a dark green, microcrystalline solid. ^{1}H NMR (400 MHz, CD_3CN, 300 K): δ = 9.49 (s), 8.52 (s), 7.59 (s), 6.83 (s), 6.50 (s), 5.72 (s), 4.47 (d, J = 169.8 Hz), 3.43 (s), 2.97 (s) ppm; ^{19}F NMR (376 MHz, CD_3CN, 300 K): δ = -78.66 (s, CF_3) ppm; IR (neat): 3533 (w), 3130 (w), 2941 (w), 1520 (w), 1450 (m), 1412 (m), 1371 (w), 1348 (w), 1275 (s), 1225 (m), 1157 (s), 1109 (w), 1076 (m), 1034 (s), 999 (w), 953 (w), 939 (w), 921 (w), 897 (w), 860 (s), 847 (m), 837 (m), 829 (m), 818 (m), 804 (m), 789 (m), 783 (s), 771 (m), 756 (m), 733 (m), 725 (m), 716 (m), 704 (m), 686 (m), 675 (m), 665 (m), 652 (m), 646 (w), 640 (w), 621 (m), 613 (w), 606 (w), 600 (m), 582 (s), 569 (s), 559 (s), 546 (s), 530 (m), 525 (m), 507 (m), 500 (m), 491 (w), 484 (w), 475 (w), 467 (m), 457 (m), 447 (w), 440 (w), 422 (m), 411 (w), 388 (m), 374 (m) cm^{-1}; UV/Vis (acetone): λ_{max} (ε) = 325 nm (2496 M^{-1} cm^{-1}), 368 nm (5812 M^{-1} cm^{-1}), 680 nm (168 M^{-1} cm^{-1}), 860 nm (121 M^{-1} cm^{-1}); HRMS (ESI) *m/z* calcd. for $C_{26}H_{34}N_{12}Cu_2OH$: 219.0544 [M-3OTf]$^{3+}$; found: 219.0540. In this case, accurate measurement of the elemental composition was not feasible due to the high fluorine content of the sample. Given these constraints, MS was employed as alternative characterization technique to confirm the identity of the compound.

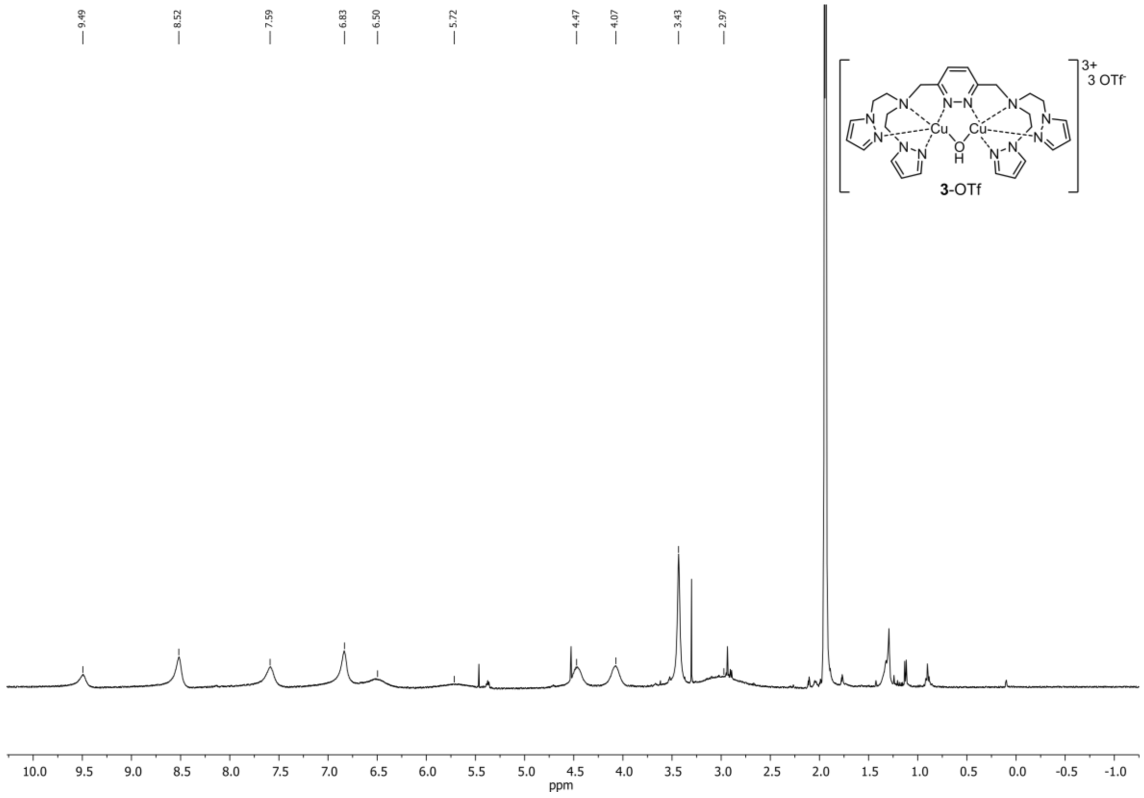

Figure S15: ^{1}H-NMR-spectrum of **3-**OTf.

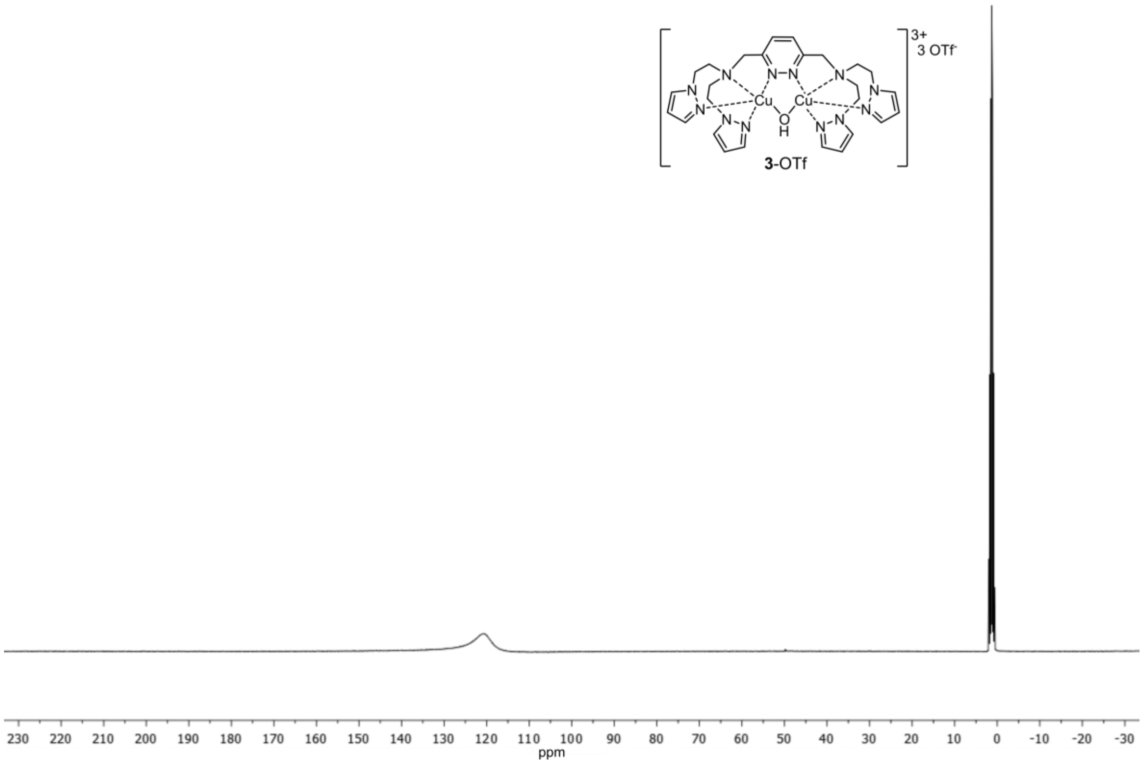

Figure S16: ^{13}C-NMR-spectrum of **3-**OTf.

2.3.5.Synthesis of [Cu_2(OH)(MO8)](PF_6)$_3$ (3-PF_6)

The synthesis of the [Cu_2(OH)(**MO8**)](PF_6)$_3$ complex **3**-PF_6 was also based on the literature of KUZELKA *et al*.[24] with the difference that $AgPF_6$ was used instead of AgOTf. 88.4. mg (94.9 µmol) of the copper(I)-complex **2**-PF_6 were dissolved in 3 mL methanol. Afterwards 48.0 mg (2 eq., 190 µmol) of $AgPF_6$ were dissolved in 2 ml methanol and added to the **2**-PF_6 solution, resulting in a green suspension after 5 minutes. The suspension was stirred for 45 minutes at room temperature and then filtered using a syringe filter. As the resulting complex seemed to be much less soluble in methanol than **3**-OTf, 5 mL acetone and 5 mL acetonitrile were used to rinse the complex out of the syringe filter. The solvent of the green solution was removed under reduced pressure and a green solid 61.1 mg (56.1 µmol, 59 %) was obtained.

^{1}H NMR (400 MHz, CD_3CN, 300 K): δ = 10.15(s), 9.11(s), 8.34 (s), 7.42 (s), 6.74 (s), 5.84 (s), 4.32 (m), 2.79 (s), 2.25 (s) ppm; ^{19}F NMR (376 MHz, CD_3CN, 300 K): δ = -72.57 (d, J = 706.8 Hz, PF_6) ppm; ^{31}P NMR (162 MHz, CD_3CN, 300 K): δ = -144.54 (hept, J = -144.54 Hz, PF_6) ppm; IR (neat): 3530 (w), 3132 (w), 2945 (w), 1713 (w), 1520 (w), 1412 (m), 1298 (w), 1277 (w), 1225 (w), 1190 (m), 1137 (w), 1107 (w), 1074 (m), 1040 (w), 1016 (w), 999 (w), 918 (m), 895 (w), 872 (s), 849 (w), 833 (w), 816 (m), 796 (w), 787 (w), 771 (m), 735 (m), 719 (m), 704 (s), 687 (s), 675 (w), 665 (m), 656 (s), 630 (s), 604 (s), 588 (s), 557 (s), 542 (w), 523 (m), 511 (m), 501 (m), 484 (w), 470 (m), 455 (m), 424 (w), 397 (w), 374 (m) cm^{-1}; UV/Vis (acetone): λ_{max} (ε) = 321 nm (4155 M^{-1} cm^{-1}), 365 nm (6099 M^{-1} cm^{-1}), 695 nm (157 M^{-1} cm^{-1}), 860 nm (119 M^{-1} cm^{-1}); HRMS (ESI) *m/z* calcd. for $C_{26}H_{34}N_{12}Cu_2OH$: 219.0544 [M-3PF_6]$^{3+}$; found: 219.0547, *m/z* calcd. for $C_{26}H_{34}N_{12}PF_6Cu_2OH$: 401.0640 [M-2$PF_6$]$^{2+}$; found: 401.0641, *m/z* calcd. for $C_{26}H_{34}N_{12}P_2F_{12}Cu_2OH$: 947.0927 [M-$PF_6$]$^{+}$; found: 947.0978. In this case, accurate measurement of the elemental composition was not feasible due to the high fluorine content of the sample. Given these constraints, MS was employed as alternative characterization technique to confirm the identity of the compound.

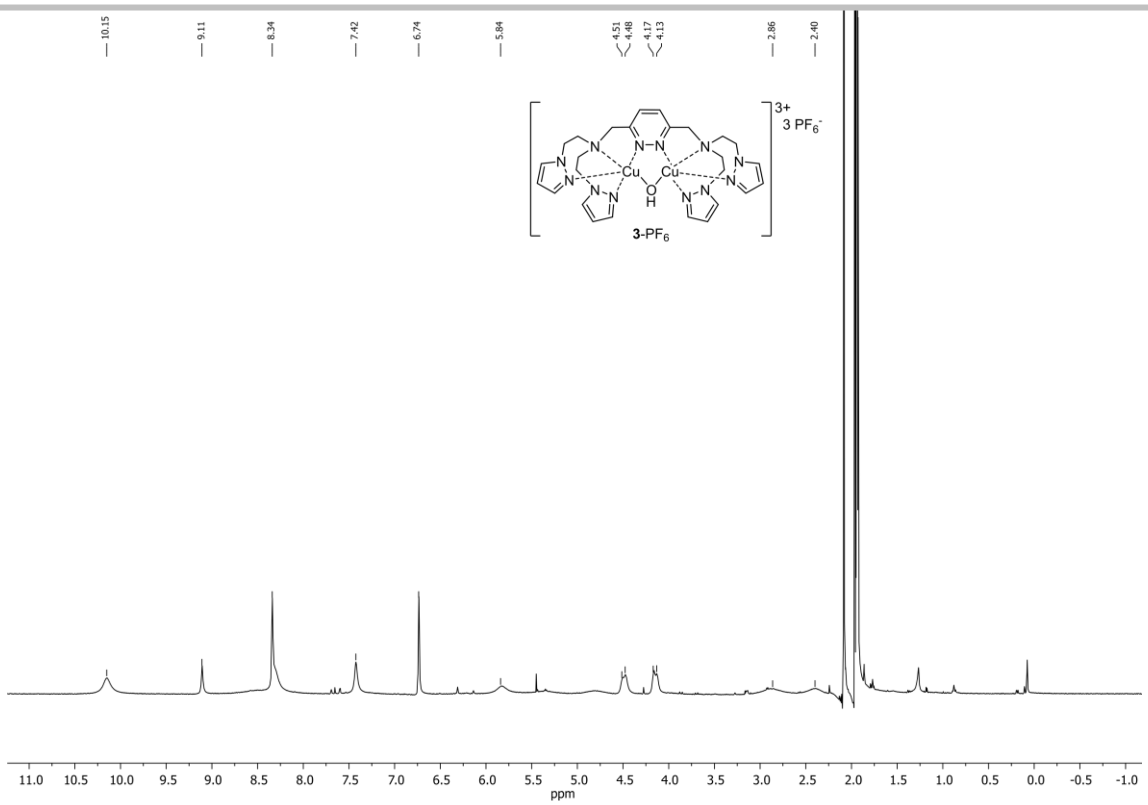

Figure S17: ^{1}H-NMR-spectrum of **3**-PF_6.

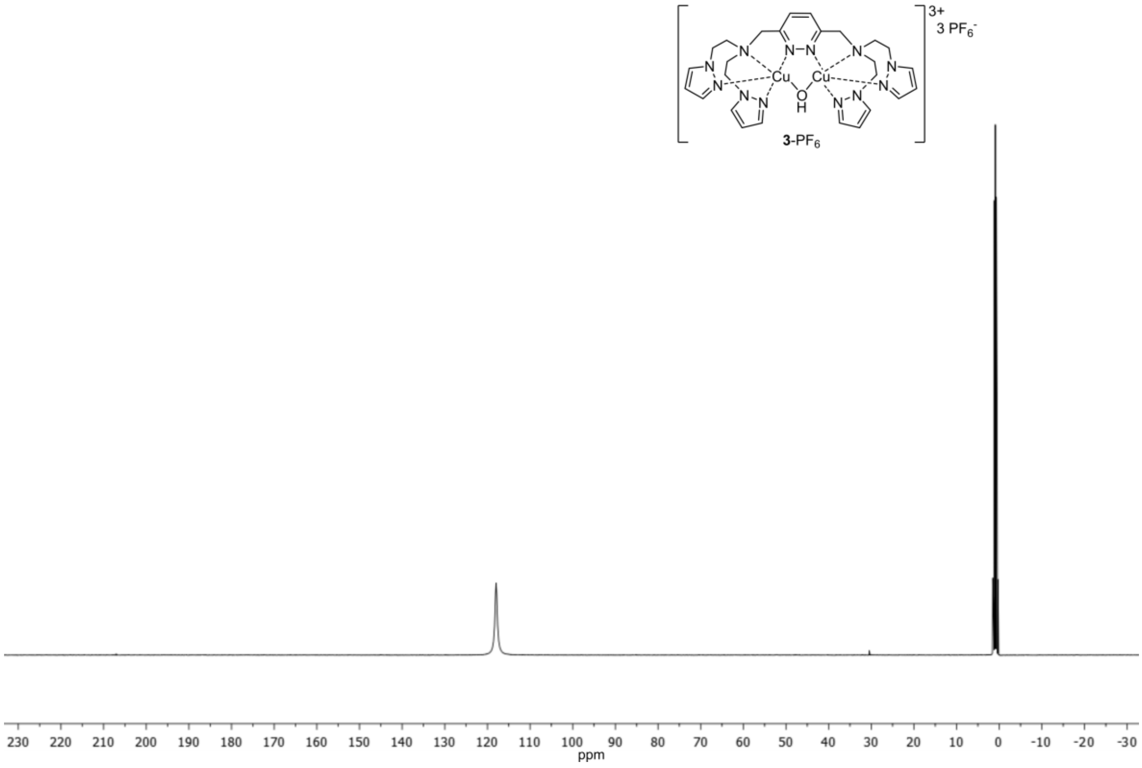

Figure S18: ^{13}C-NMR-spectrum of **3**-PF_6.

2.4. Synthesis of oxygen atom-transfer reagents

2.4.1.Synthesis of iodosobenzene (PhIO)

Iodosobenzene was synthesized based on literature.[25] To 5.00 g (15.5 mmol) of diacetoxyiodobenzene, 28 mL of a 3 M sodium hydroxide solution was added at room temperature within 10 min. The yellow reaction mixture was stirred for 1 h and 45 min at room temperature and then 25 mL of water was added. After further stirring for 1 h and 30 min at room temperature, the solid was filtered off, washed with 180 mL chloroform, and dried in vacuo. 3.10 g (14.1 mmol, 91 %) of PhIO was obtained. The light yellow solid was stored at -32 °C to avoid decomposition.

^{1}H-NMR (500 MHz, Methanol-d_4, 300 K): δ = 8.03 (m, 2H), 7.58 (m, 3H) ppm; ^{13}C NMR (126 MHz, Methanol-d_4, 300 K): δ = 133.3, 132.2, 132.0, 122.6 ppm.

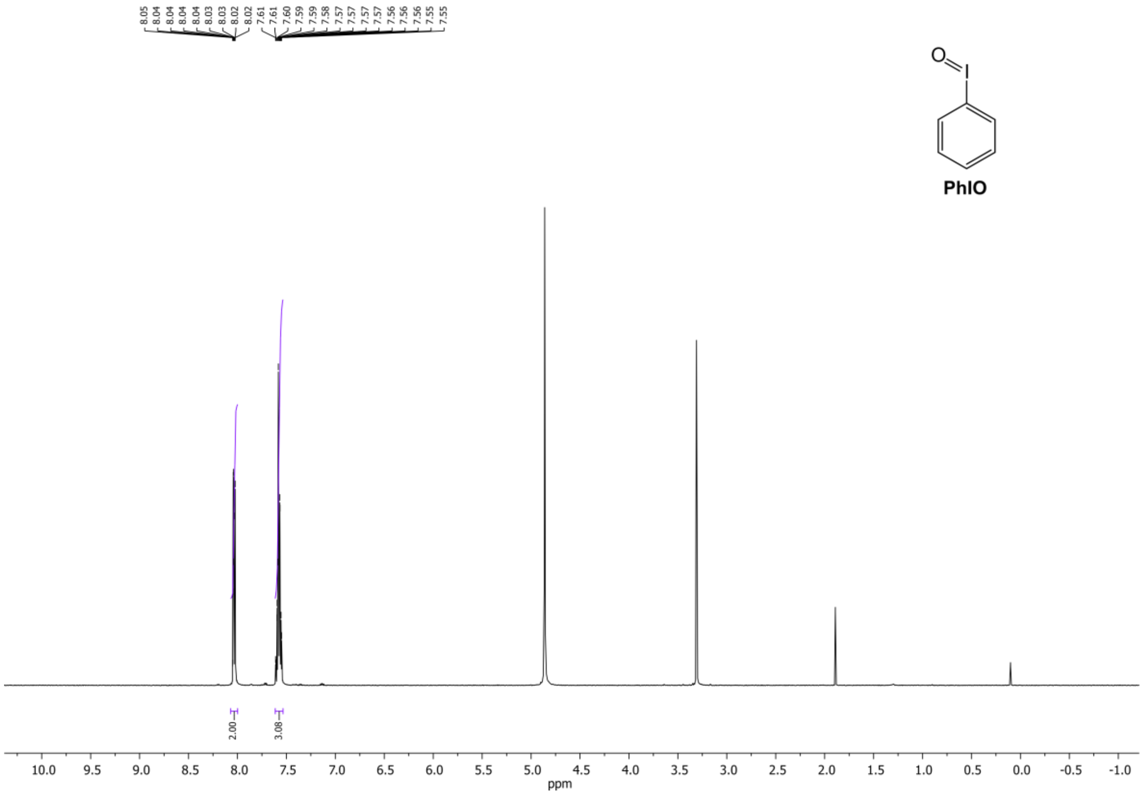

Figure S19: ^{1}H-NMR-spectrum of **PhIO**.

2.4.2.Synthesis of dimethyldioxirane (DMDO)

Dimethyldioxirane was synthesized according to literature.[26] To a 1000 mL three-necked flask, 25.4 mL water, 19.2 mL acetone, and 5.8 g sodium bicarbonate were added and the mixture was cooled to 5-10 °C with an ice/water bath. Subsequently, 12.0 g (19.5 mmol) of oxone were added in five portions at 3-min intervals, resulting in a colorless suspension. 3 min after the last addition, the cooling bath was removed and the product was distilled into a receiving flask connected *via* a U tube at 110 mbar, which had been cooled down to -78 °C by an ethanol cooling bath beforehand. The distillate was dried over sodium sulfate and decanted into a new flask. A pale-yellow solution (25 mL) of DMDO in acetone resulted, which was stored over molecular sieves (4 Å) at -32 °C. According to literature [27], the concentration of DMDO was determined by reacting a defined volume of the DMDO solution with an excess of thioanisole and following analysis of the reaction mixture by ^{1}H-NMR spectroscopy: 0.6 mL of a 0.7 M thioanisole solution in acetone-d_6 were cooled to 10 °C in an ice/water bath. Then, 3.0 mL of the DMDO solution was added and the mixture was stirred for 10 min. Subsequently, a portion of the solution was transferred into an NMR tube and a ^{1}H-NMR spectrum was measured. Based on the ratio of sulfoxide phenyl protons (δ = 7.5 - 7.7 ppm) and thioanisole phenyl protons (δ = 7.1 - 7.3 ppm), the amount of methyl phenyl sulfoxide formed can be determined and thus a conclusion can be drawn about the concentration of the DMDO solution. Typically, solutions with a concentration of 0.06 M – 0.09 M were obtained. Due to the thermal instability of DMDO, the solutions were freshly prepared and the concentration determined shortly before use.

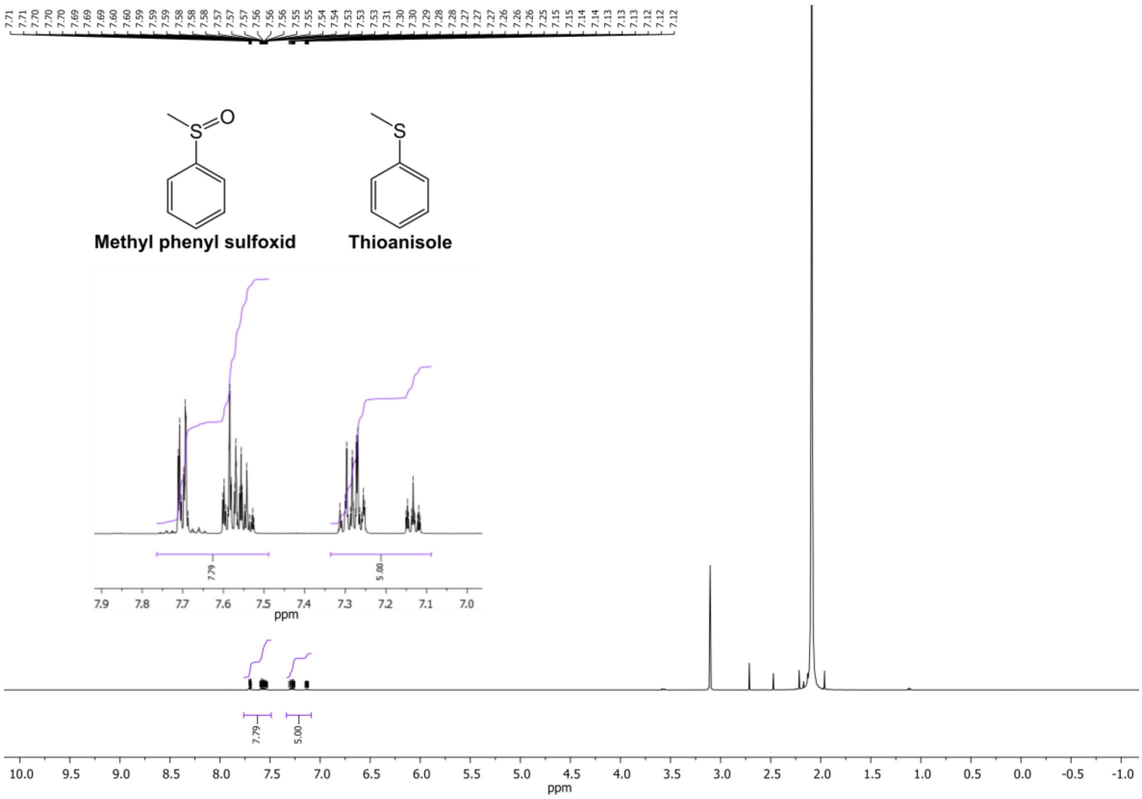

Figure S20: ^{1}H-NMR spectrum obtained after reaction of the DMDO solution with an excess of thioanisole. The sulfoxide phenyl proton signals (δ = 7.5 - 7.7 ppm) and thioanisole phenyl proton signals (δ = 7.1 - 7.3 ppm) are shown in the inset.

Part III: Results and Discussion

3. Additional Information about the Crystal Structure of 2-PF_6 and 3-PF_6

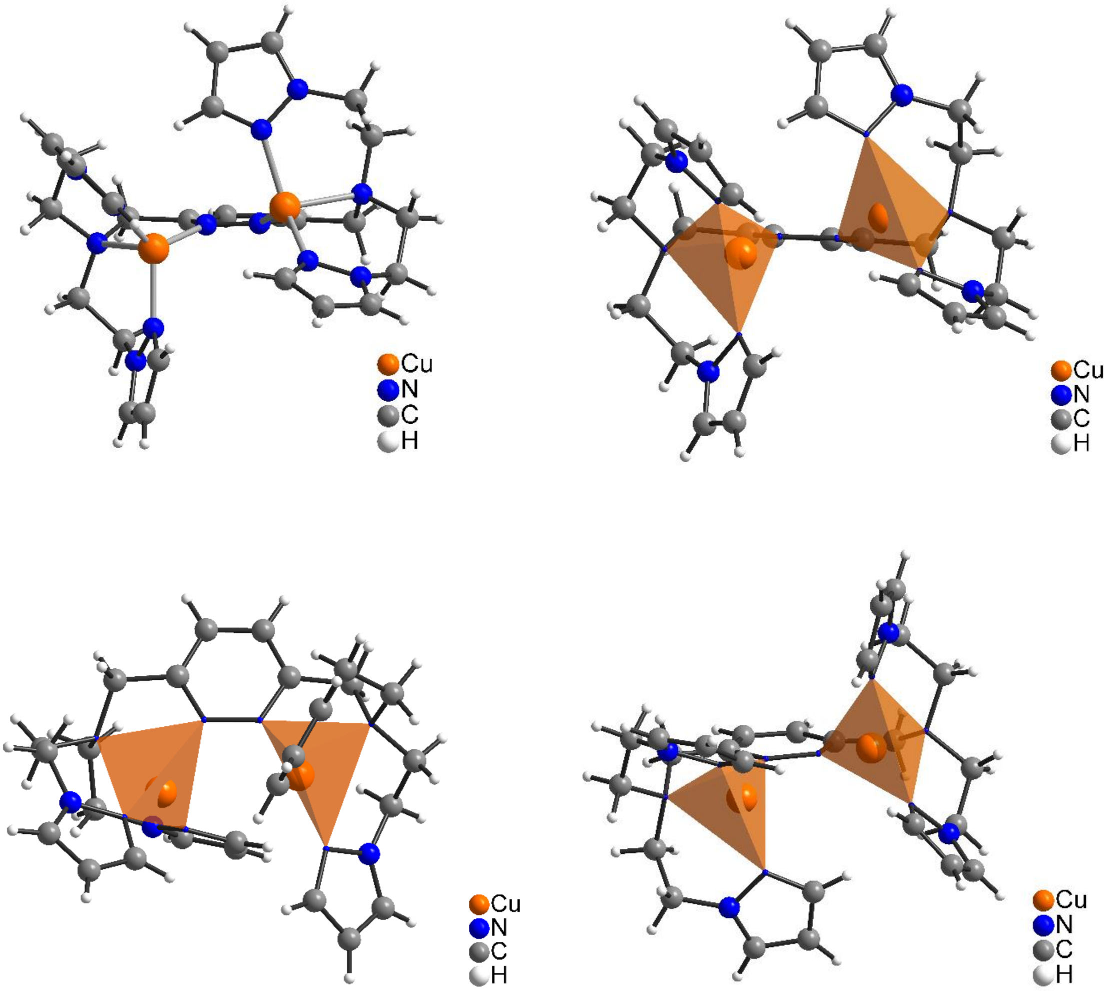

Figure S21: Different perspectives of the molecular structure of the dinuclear copper(I) complex bearing **MO8** as ligand (= **2**-PF_6). Crystal system: monoclinic. Space group: $P2_1/n$. The shown polyhedral (orange) represent tetrahedrons for the two Cu(I) units (the copper ion lies within the plane of the triangle). Notably, no additional acetonitrile or acetone molecules complete the coordination sphere of the copper(I) centers. For clarity the two crystallographically independent hexafluorophosphate anions are not shown.

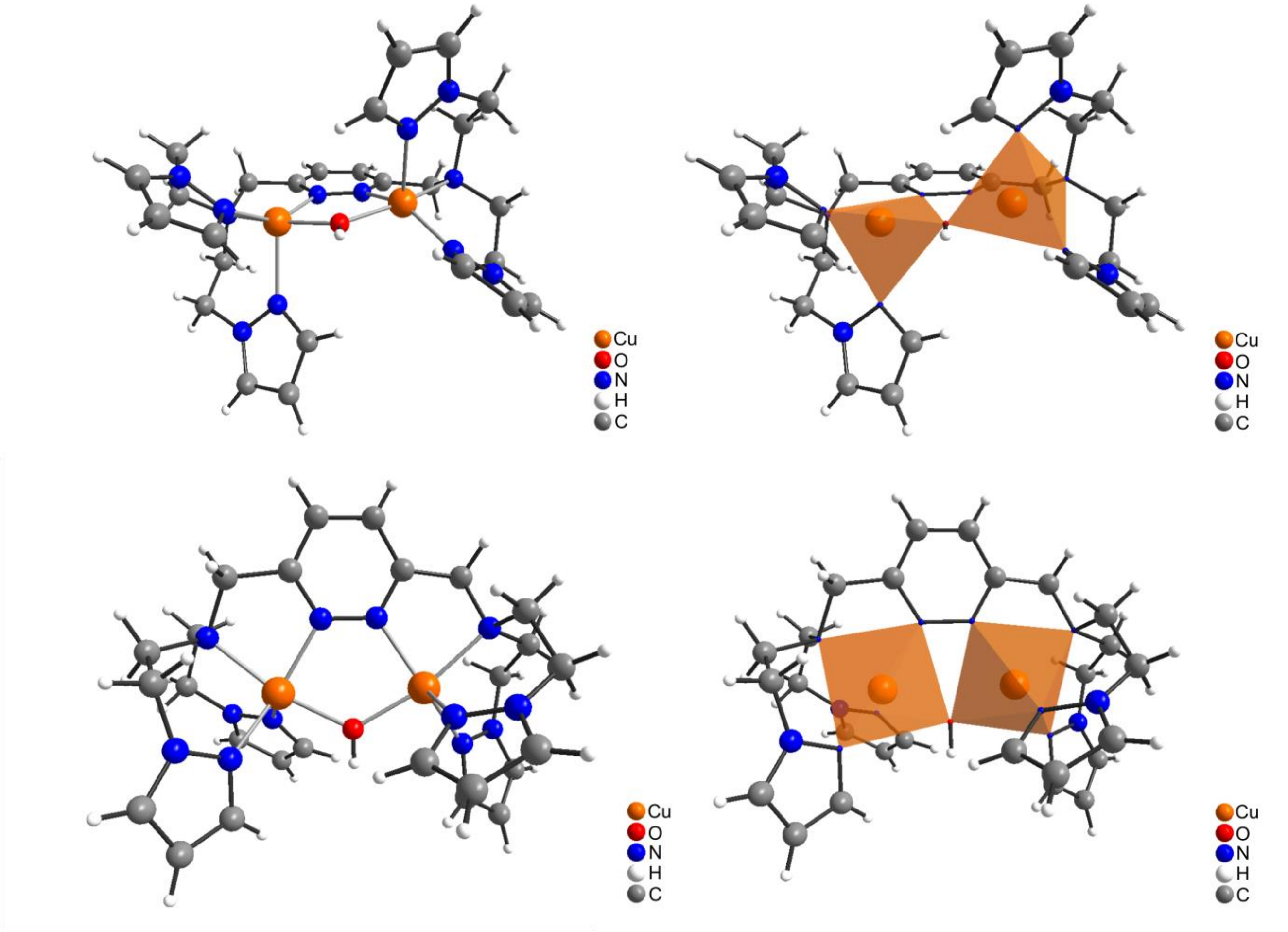

Figure S22: Different perspectives of the molecular structure of the **Cu_2OH** complex **3**-PF_6. Crystal system: orthorhombic. Space group: Pbcn. The shown polyhedral (orange) represent the distorted square pyramidal geometry for the two Cu(II) units. As in the Cu(I) complex, no additional solvent coligands coordinate to copper. For clarity the three crystallographically independent hexafluorophosphate anions are not shown.

4. X-ray powder diffraction

4.1. Diffractograms of the Cu_2OH complexes 3-PF_6 and 3-OTf

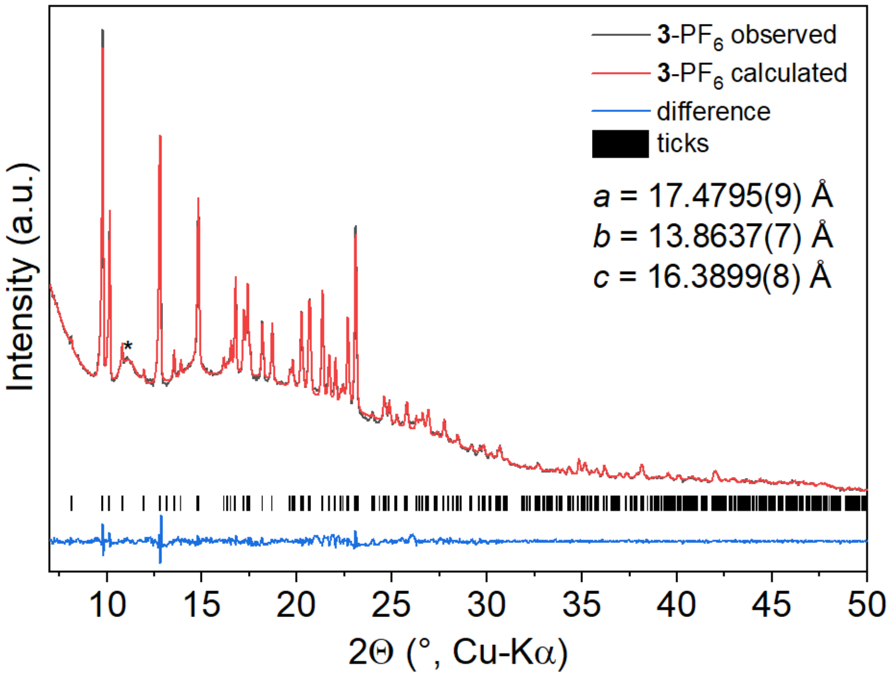

Figure S23: Pawley plot of the **Cu_2OH** complex **3**-PF_6: Observed (black), calculated (red) and difference (blue). The obtained data match the crystal structure of **3**-PF_6 well.

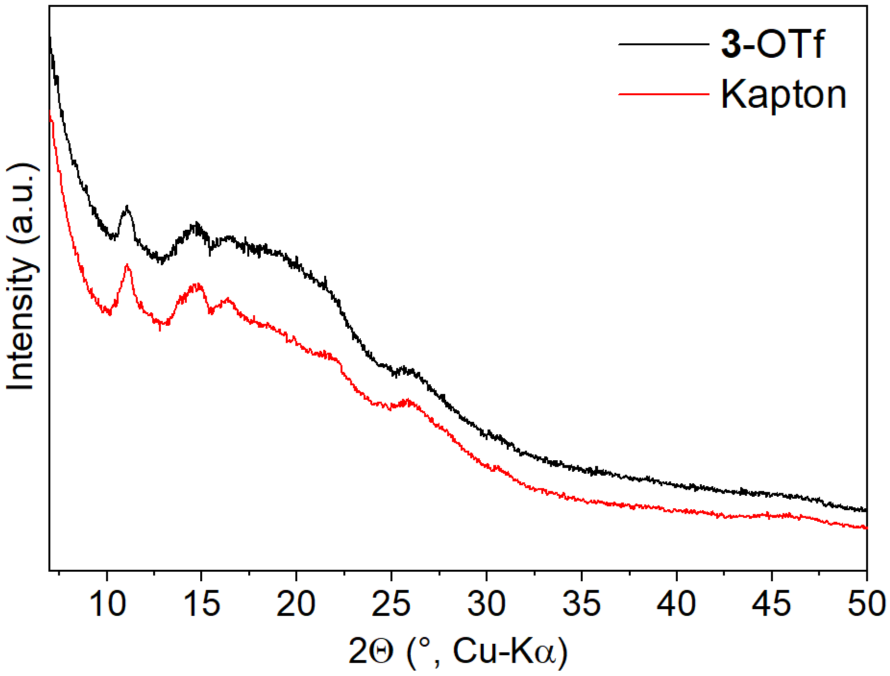

Figure S24: Powder diffractogram of the **Cu_2OH** complex **3**-OTf (black) and of the Kapton foil (red). The diffractograms show that **3**-OTf is amorphous.

5. UV/Vis Spectroscopy

5.1. Overview of the obtained results with O_2, PhIO, N_2O, DMDO and AgOTf/$AgPF_6$

Table S4 shows an overview of the UV/Vis data obtained after the reaction of **2**-PF_6 with O_2 and the oxygen-atom transfer reagents iodosobenzene (PhIO), nitrous oxide (N_2O) and dimethyldioxirane (DMDO).

Table S4. Overview of the obtained data for the Cu_2(**MO8**) model system using different oxygen transfer reagents. The presented values are given in the following form: λ / nm (ε / M^{-1} cm^{-1}).

Model system	[O]	Green species[a]	Green species[b]	Green species[c]	Cu_2OOH[d]	Cu_2OH[e]
**2**-PF_6	O_2	360 (1927)<br>645 (199)	360 (2383)<br>645 (211)	N.A.	412 (2362)<br>625 (163)	-
**2**-PF_6	PhIO[f]	360 (1335)<br>651 (51)	N.A.	N.A.	N.A.	-
**2**-PF_6	N_2O	N.A.	355 (4780)<br>645 (88)	N.A.	N.A.	-
**2**-PF_6	DMDO	N.A.	N.A.	368 (4498)<br>658 (134)	N.A	-
**2**-OTf	AgOTf + MeOH	N.A.	N.A.	N.A.	N.A.	368 (5344)<br>680 (168)<br>860 (121)
**2**-PF_6	$AgPF_6$ + MeOH	N.A.	N.A.	N.A.	N.A.	365 (6099)<br>695 (157)<br>860 (119)

General remarks: Solvent: acetone; c = 0.50 – 1.61 mM. [a] Formation of the green species at room temperature (293 K). [b] Formation of the green species at +35 °C (308 K).[c] Formation of the green species at -80 °C (193 K). [d] Formation of the **Cu_2OOH** species at -90 °C (183 K).[e] Formation of the **Cu_2OH** species at room temperature. [f] An excess of PhIO was used; T = -10 °C (263 K).

5.2. Additional UV/Vis spectra for the Cu_2OH complex

The UV/Vis spectra of the deprotonation reaction of the **Cu_2OH** complex **3** indicate that the deprotonation seems to be only partially reversible. The addition of diazabicycloundecene (DBU) to the **Cu_2OH** complex **3** (Figure S25, black) at -50 °C lead to a decrease of the UV/Vis bands at 680 nm and 860 nm. Furthermore, a new shoulder appeared at 550 nm and the color of the solution turned orange (Figure S25, red). Next 2,6-lutidinium triflate ([Lut-H][OTf]) was added at -40 °C to investigate whether the deprotonation reaction is reversible. At first no reaction was observed but after warming the solution to room temperature, the shoulder at 550 nm disappeared and the bands at 680 nm and 860 nm increased again slightly (Figure S25, blue). At the end of the experiment, the bands at 368 nm showed a different intensity compared to the beginning. The color of the solution was green again but paler than that of the origin **Cu_2OH** species **3**. This indicates that irreversible processes might occur during the deprotonation.

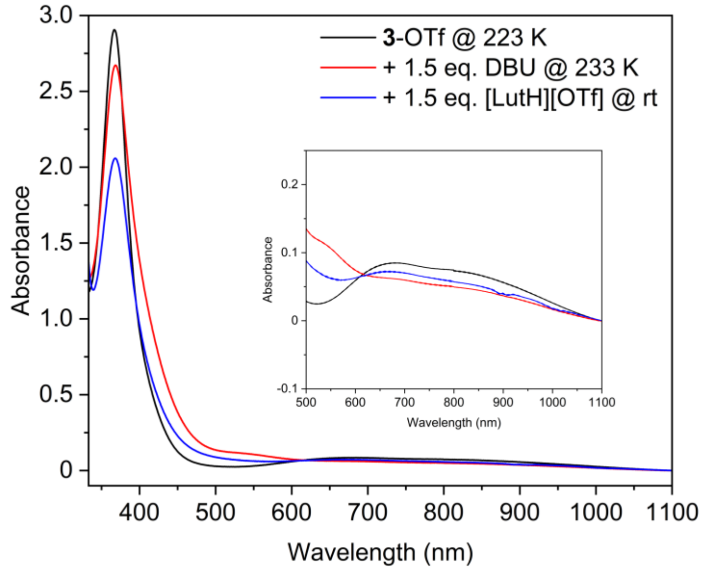

Figure S25: UV/Vis spectrum of the **Cu_2OH** complex **3**-OTf at 223 K (-50 °C) before addition of DBU (black), after addition of 1.5 eq. DBU (red) at 233 K (-40 °C) and after addition of 1.5 eq. [Lut-H][OTf] and warming to rt (blue).

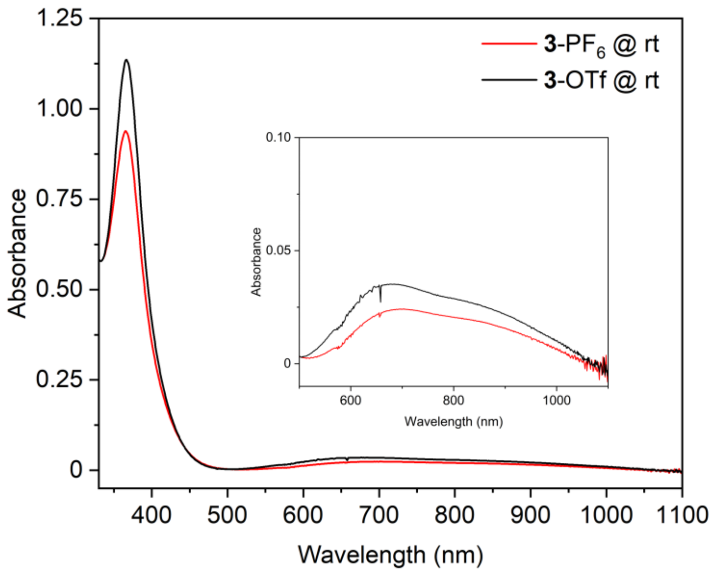

Figure S26: UV/Vis spectrum of the **Cu_2OH** complex **3**-PF_6 at rt (red) and the **Cu_2OH** complex **3**-OTf at rt (black). The spectra show nearly the same band structure.

5.3. Additional UV/Vis spectra for the Cu_2OOH complex

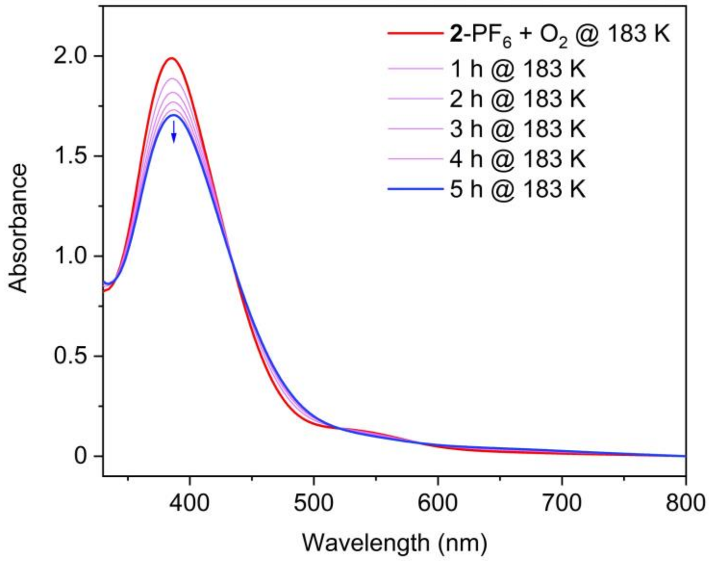

Figure S27: Stability of the $\mathbf{Cu_2OOH}$ complex of **2**-PF_6 in acetone for 300 min at 183 K (-90 °C).

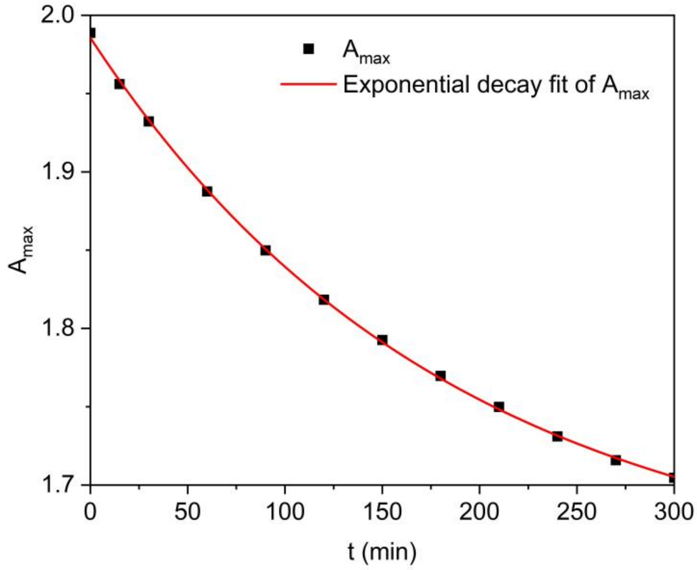

Figure S28: Thermal decay of the $\mathbf{Cu_2OOH}$ complex of **2**-PF_6 in acetone at 183 K (-90 °C); Plot A_{max} against t.

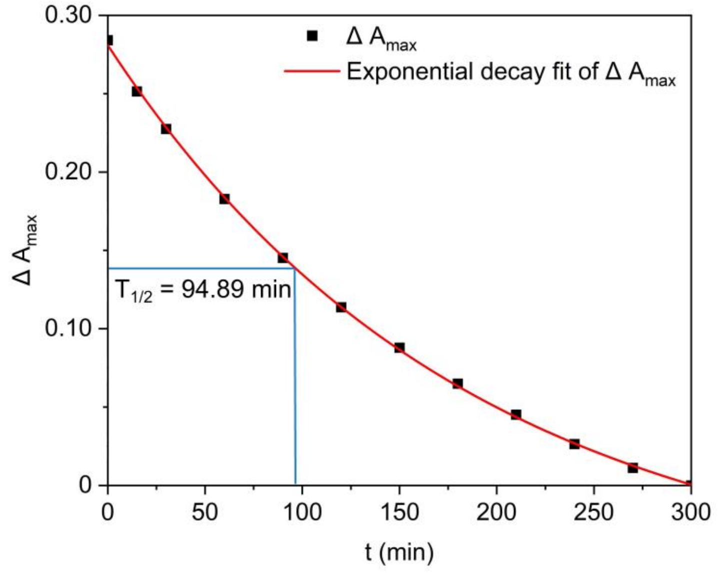

Figure S29: Determination of the half-life of the **Cu_2OOH** complex of **2**-PF_6 in acetone at 183 K (-90 °C) from the plot in Figure S28. The A_{max} value at 300 min was set to 0.

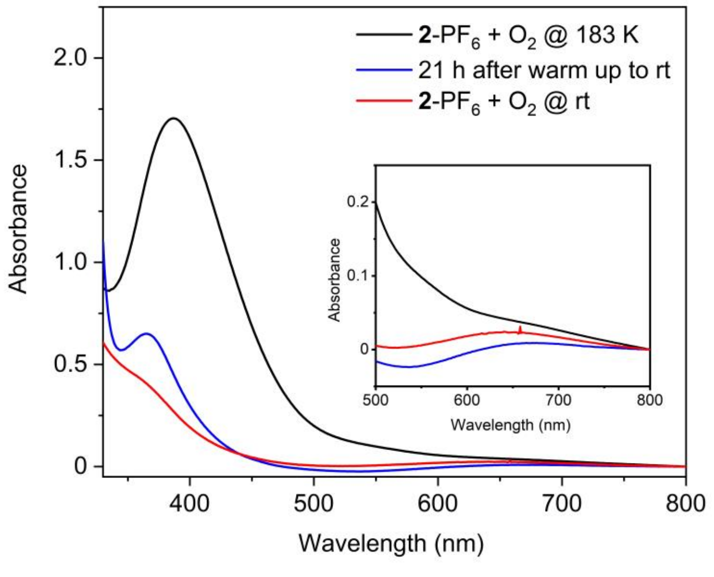

Figure S30: Warming up the **Cu_2OOH** from **2**-PF_6 (Figure S27) to room temperature. The warm up leads to a decay of the **Cu_2OOH** complex and a decrease of the UV/Vis bands.

5.4. Additional UV/Vis spectra for the green species

The reaction of **2**-PF_6 with O_2 for 8 h at 308 K leads to a spectrum comparable to that of the 24 h experiment at rt (see Figure S31)

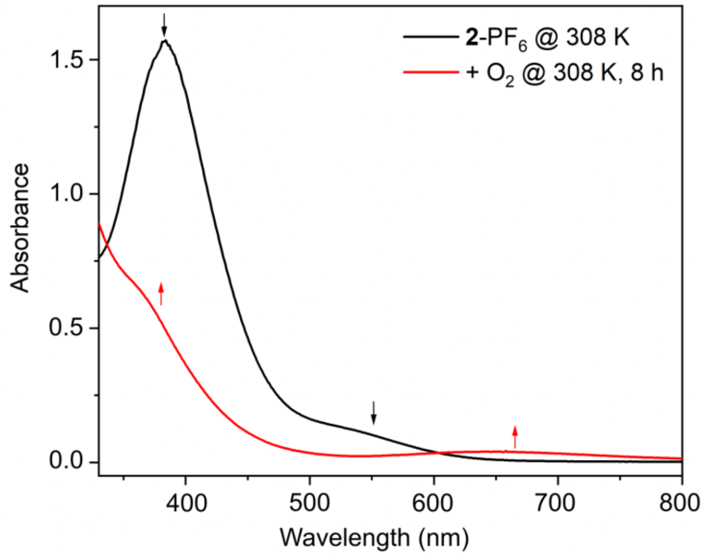

Figure S31: Absorption spectra of an acetone solution of **2**-PF_6 before (black) and upon with O_2 (red) at 308 K (+35 °C).

The reaction of **2**-PF_6 with O_2 in acetonitrile (Figure S32) resulted in the same color change of the solution to green and absorption bands. However, the absorption bands are weaker than in acetone.

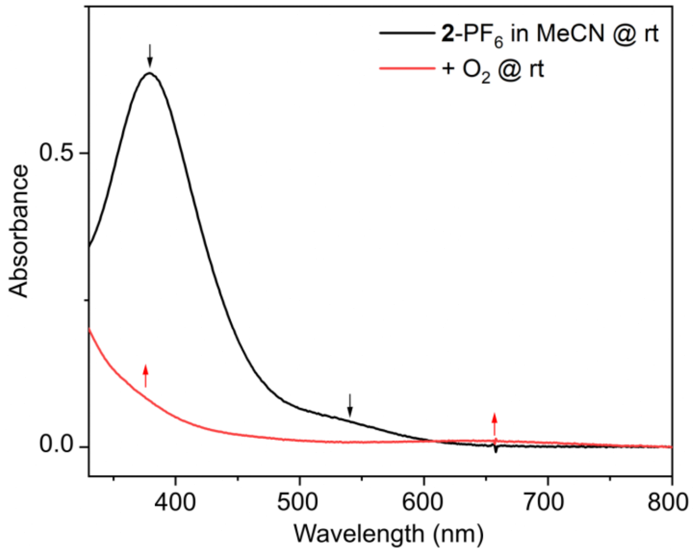

Figure S32: Absorption spectra of an acetonitrile solution of **2**-PF_6 before (black) and upon reaction with O_2 (red) at rt.

The reaction of **2**-PF_6 with N_2O (Figure S33) led to a similar band formation in the UV/Vis spectrum as the reaction with O_2. In contrast, the reaction with N_2O is slower and a reaction temperature of 308 K is required. In addition the band at 355 nm is more pronounced than in the reaction with O_2.

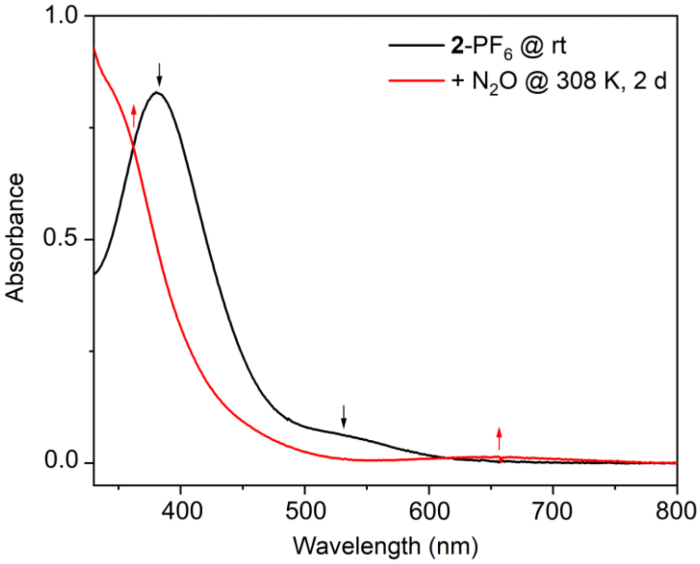

Figure S33: Absorption spectra of an acetone solution of **2**-PF_6 before (black) and upon reaction with N_2O (red) at 308 K (+35 °C).

Figure S34 shows a comparison of the UV/Vis spectra of **2**-PF_6 obtained after a reaction with O_2 at rt (red) and after a reaction with DMDO at 193 K (blue). The bands are in a similar position, but in the experiment with DMDO the bands are more distinct, which could be caused by the possibly better stabilization of the copper-oxygen intermediate at low temperatures.

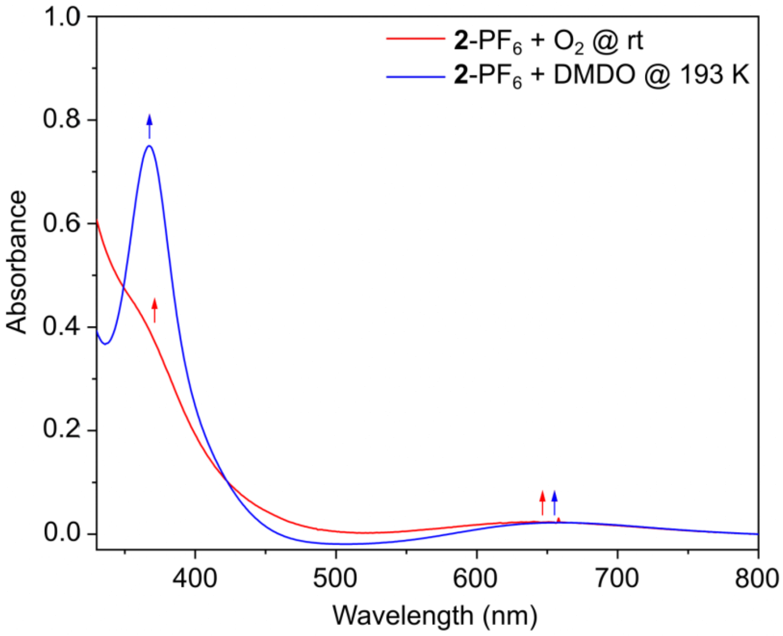

Figure S34: Oxygenation of a solution of **2**-PF_6 in acetone with O_2 at rt (red) and with DMDO at 193 K (blue). The bands have the same position and thus indicate the formation of the same copper-oxygen intermediate (**Cu_2O**). In the case of oxygenation with DMDO, however, the bands are more distinct due to the possibly better stabilization of this species at the lower temperature.

When the solution of **2**-PF_6 after reaction with DMDO was warmed up to room temperature, the bands decreased which shows that the copper-oxygen intermediate is probably not stable at elevated temperatures (Figure S35).

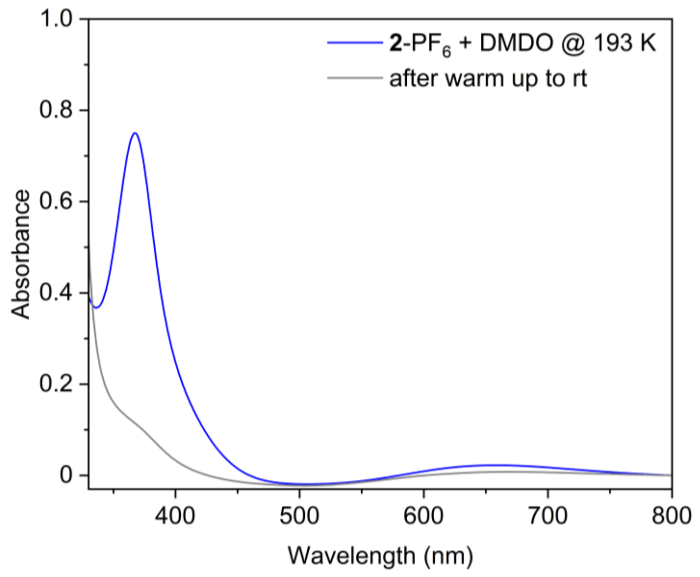

Figure S35: UV/Vis spectrum of **2**-PF_6 after reaction with DMDO at 193 K (blue) and resulting spectrum after warming to rt (grey).

6. Resonance Raman Spectroscopy

6.1. Spectroscopic data and calculated vibrations

Table S5: Overview of the obtained Data for the measured frequencies and DFT-expected vibrations of the Cu_2(**MO8**) model system **2** for the **Cu_2OOH** species **5** and the **Cu_2O** species **4** which was discussed as a possible copper-oxygen intermediate at rt (green species).

Vibration mode ($\Delta^{18}O_2$) / cm^{-1}	Cu_2OOH		Cu_2O	
	DFT	experimental data	DFT	experimental data
$vs_{Cu\text{-}O}$	282 (10-30)[a]<br>278 (9-31)[b	330 (20-30)	402 (18)[a]<br>403 (19)[b]	N. A.
$vas_{Cu\text{-}O}$	495 (22)[a]<br>482 (23-31)[b]	550 (30-40)	533 (24)[a]<br>525 (21)[b]	N. A.
$v_{O\text{-}O}$	936 (51-68)[a]<br>901 (54)[b	886 (50)	-	-

General remarks: DFT: The frequency calculations were performed without any solvent molecule attached (naked **Cu_2O** and **Cu_2OOH** complexes). The O-H stretching vibration could not be observed experimentally. UV/Vis (experimental data): Solvent: acetone. [a] DFT: RI-PBE-D3(BJ)/def2-SVP. [b] DFT: RI-PBE-D3(BJ)/def2-TZVP(-f).

6.2. Theoretical data derived from DFT

Table S6: Overview of the theoretical obtained Raman features for the Cu_2(**MO8**) model system **5** (**Cu_2OOH**) and **4** (**Cu_2O**) using DFT.

Vibration mode ($\Delta^{18}O_2$) / cm^{-1}	Cu_2OOH			Cu_2O		
	naked	acetone[d]	MeCN	naked	acetone	MeCN[c]
$vs_{Cu\text{-}O}$	282 (10-30)	273/275<br>(10-12)	247	402 (18)	420 (16)[a]<br>455 (19)[b]	394
$vas_{Cu\text{-}O}$	495 (22)	447/453<br>(19-25)	456	533 (24)	549 (36)[a]<br>587 (28)[b]	598
$v_{O\text{-}O}$	936 (51-68)	935 (58)	901/915	-	-	-
$v_{O\text{-}O\text{-}H}$	1358 (8)	1532 (3)	1340/1343	-	-	-
$v_{O\text{-}H}$	3521 (12)	2635 (11)	3516	-	-	-

General remarks: DFT: RI-PBE-D3(BJ)/def2-SVP. [a] One acetone molecule is coordinated. [b] Two acetone coligands are coordinated. [c] Only one acetonitrile molecule is coordinated; the second acetonitrile is too far away from any of the copper centers (d > 3.24 Å). [d] Only one acetone molecule is coordinated; the second acetone molecule is close to the bridging μ-OOH ligand and is bound via hydrogen bonds.

6.3. Additional resonance Raman spectra

6.3.1. Measurements at room temperature using O_2

After reaction of **2**-PF_6 with O_2 at rt, no new signals can be detected in the resonance Raman spectrum that can be assigned to a Cu-O vibration (Figure S36).

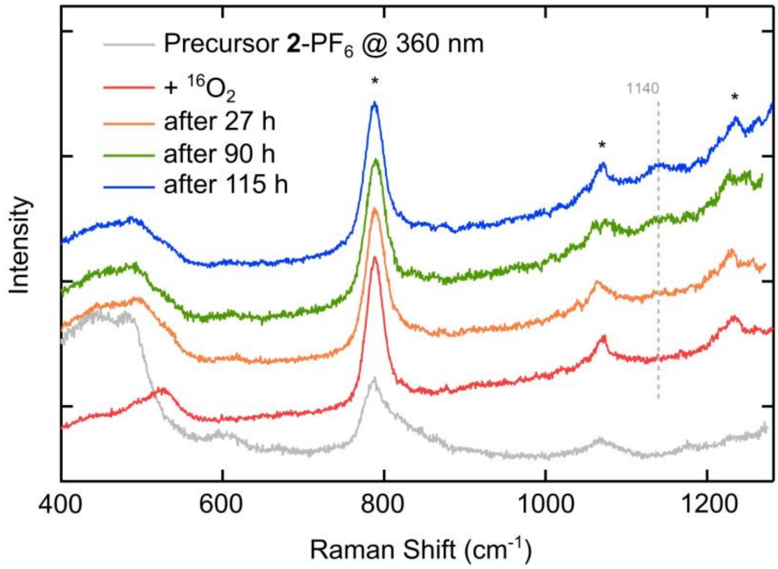

Figure S36: Resonance Raman spectra of **2**-PF_6 before (grey) and upon reaction with $^{16}O_2$ (red to blue) at room temperature. Raman: The asterisks mark solvent signals of acetone. The laser excitation wavelength was 360 nm.

6.3.2. Measurements at +35 °C (308 K) using O_2

No isotope-sensitive signal could be found in an experiment with $^{18}O_2$ (Figure S37).

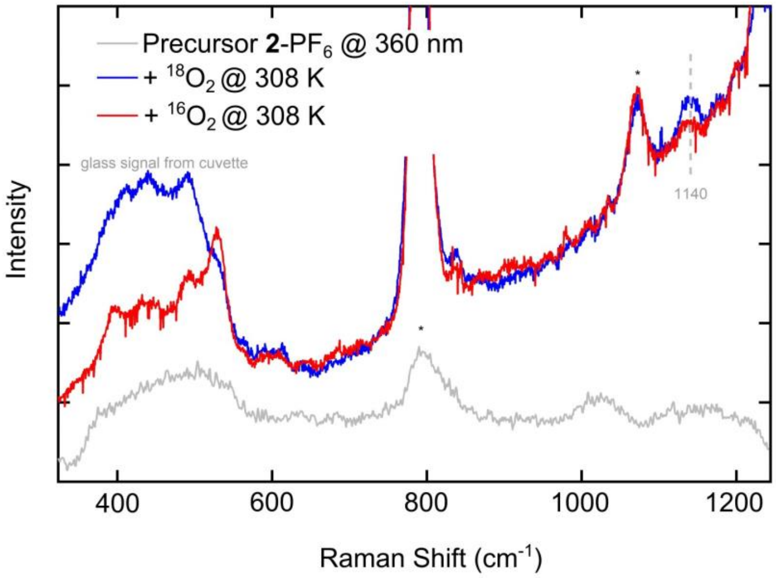

Figure S37: Resonance Raman spectra of **2**-PF_6 before (grey) and upon reaction with $^{16}O_2$ (red) and $^{18}O_2$ (blue) at +35 °C. Raman: The asterisks mark solvent signals of acetone. The laser excitation wavelength was 360 nm.

7. IR-Spectroscopy

7.1. Theoretical data for the Cu_2OH complex derived from DFT

Table S7: Overview of the DFT calculated IR-vibrations for the **Cu_2OH** species **3** compared to the experimental data of **3**-OTf.

Vibration mode / cm^{-1}	Cu_2OH	
	DFT	experimental data
$\nu_{O\text{-}H}$	3929	3533
$\nu s_{Cu\text{-}O}$	423	-
$\nu as_{Cu\text{-}O}$	495	-
$\nu_{out\text{-}of\text{-}plane}$	131	-

General remarks: DFT: The frequency calculations were performed without any solvent molecule attached (naked **Cu_2OH** complex). Symmetric Cu-O vibration = $\nu s_{Cu\text{-}O}$ and antisymmetric Cu-O vibration = $\nu as_{Cu\text{-}O}$. The Cu-O stretching vibrations could not be observed experimentally. DFT: RIJCOSX-PBE0-D3(BJ)/def2-TZVPP.

The IR-vibrations of $\nu s_{Cu\text{-}O}$ and $\nu as_{Cu\text{-}O}$ could not be identified clearly since the IR spectra of **3**-OTf shows many weakly intense bands in the expected range (Figure S41).

7.2. IR spectrum of 2-OTf and 3-OTf

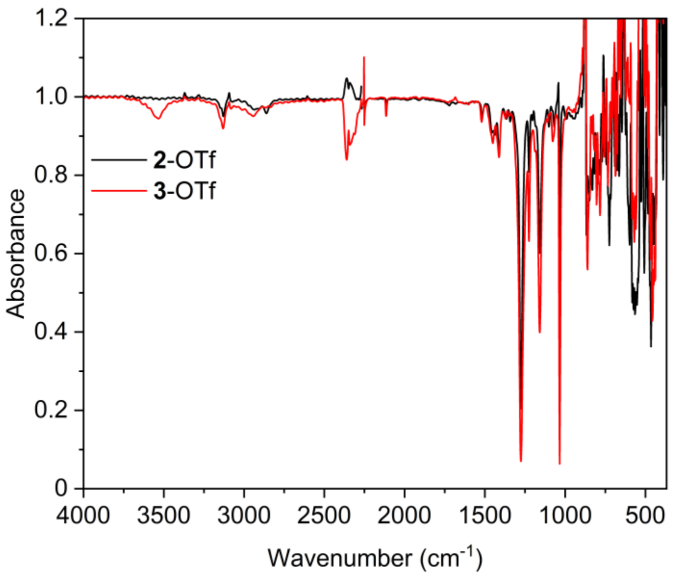

Figure S38: IR spectra of the Cu(I)-complex **2**-OTf (black) and the **Cu_2OH** species **3**-OTf (red).

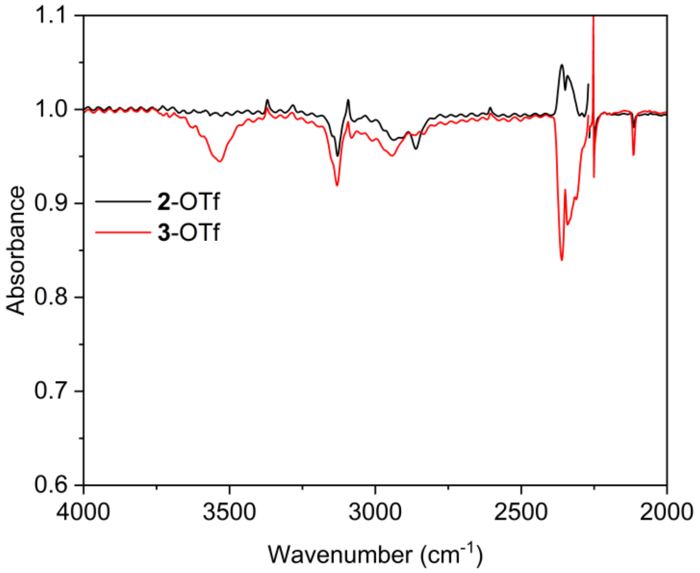

Figure S39: IR spectra of the Cu(I)-complex **2**-OTf (black) and the **Cu_2OH** species **3**-OTf (red) from Figure S38 from 4000 – 2000 cm^{-1}.

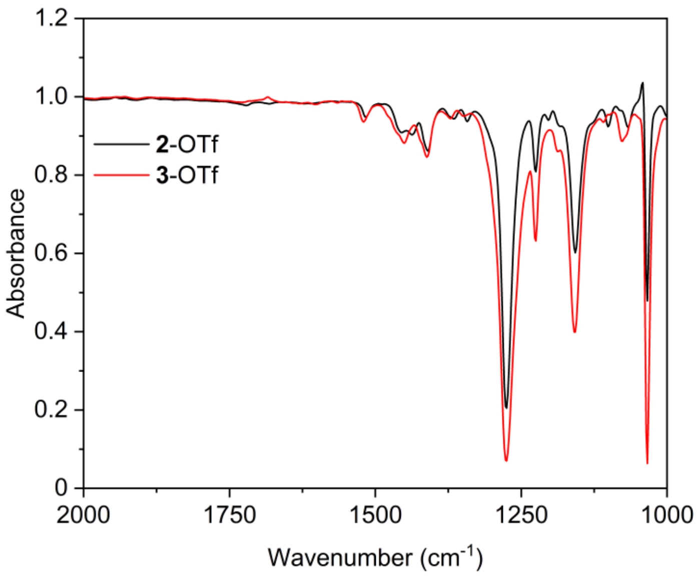

Figure S40: IR spectra of the Cu(I)-complex **2**-OTf (black) and the **Cu_2OH** species **3**-OTf (red) from Figure S38 from 2000 – 1000 cm^{-1}.

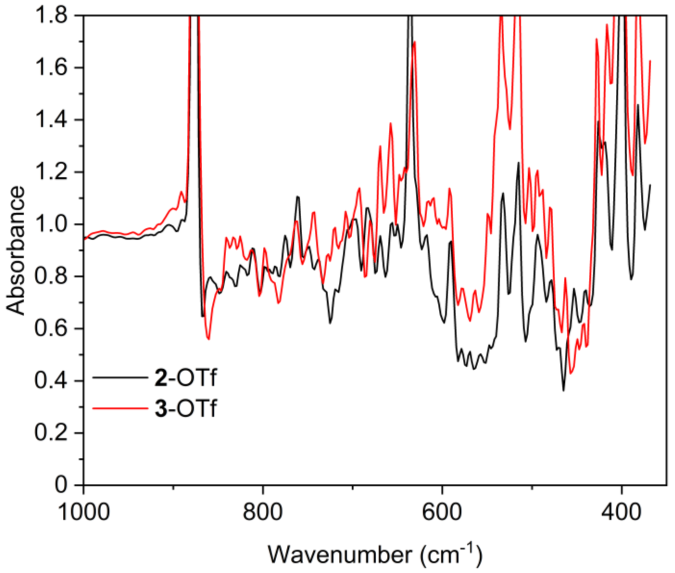

Figure S41: IR spectra of the Cu(I)-complex **2**-OTf (black) and the **Cu_2OH** species **3**-OTf (red) from Figure S38 from 1000 – 350 cm^{-1}.

7.3. IR spectrum of 3-PF_6

In the IR spectrum of **3**-PF_6 (Figure S42), a band at 3530 cm^{-1} is present, which indicates the presence of an O-H group as in **3**-OTf.

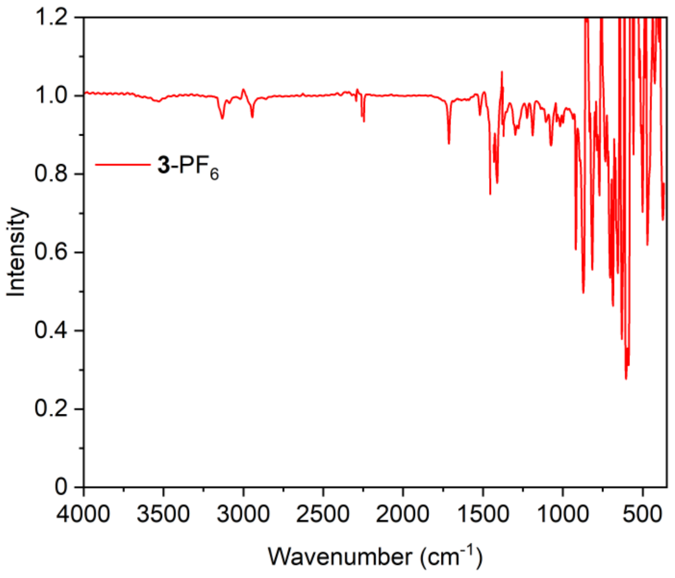

Figure S42: IR spectrum of the **Cu_2OH** complex **3**-PF_6 (red).

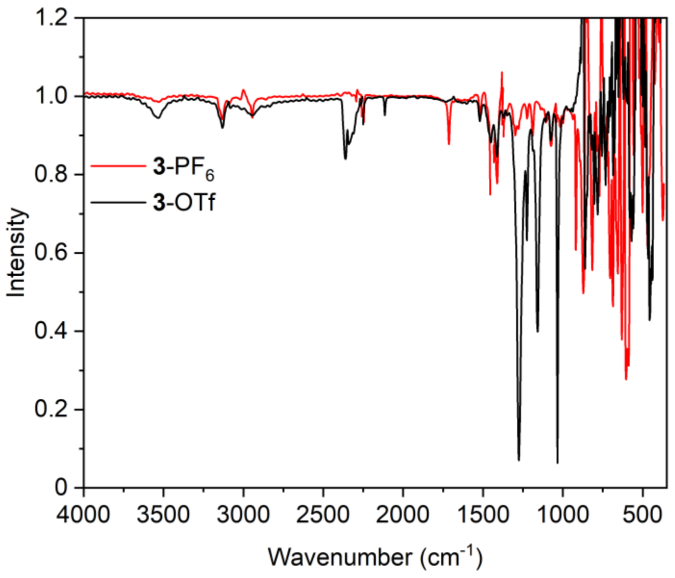

Figure S43: Comparison of the IR spectra of the **Cu_2OH** complexes **3**-PF_6 (red) and **3**-OTf (black).

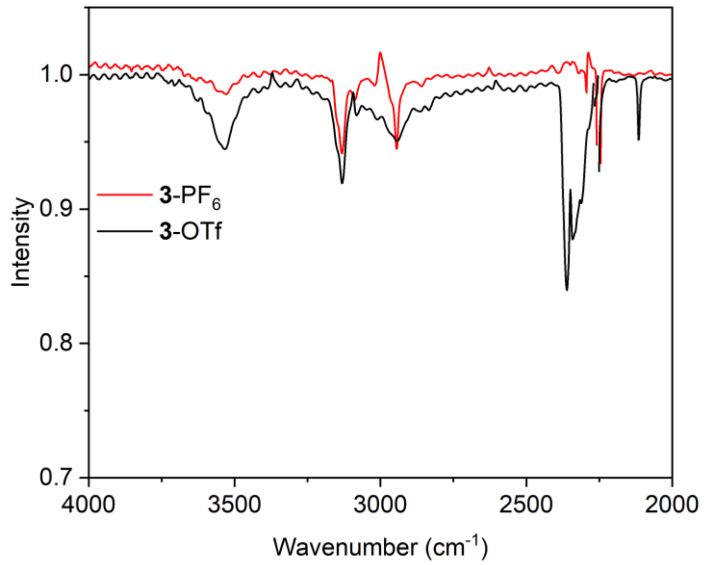

Figure S44: Comparison of the IR spectra of the **Cu_2OH** complexes **3**-PF_6 (red) and **3**-OTf (black) from Figure S43 from 4000 – 2000 cm^{-1}.

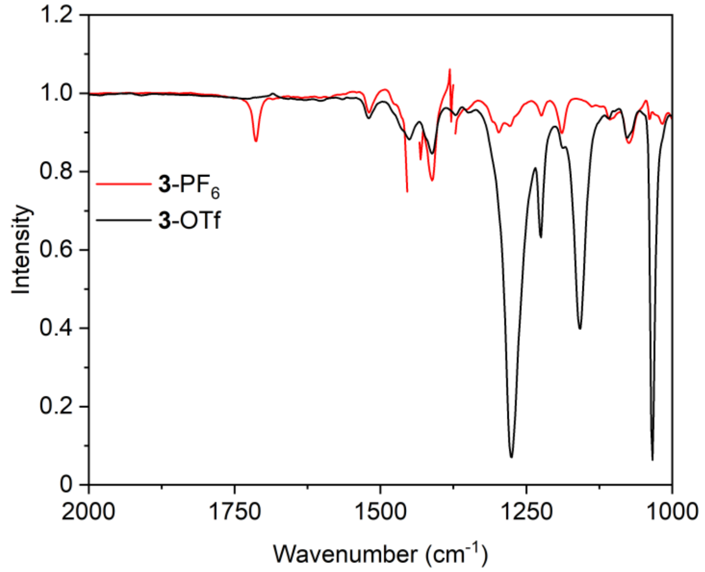

Figure S45: Comparison of the IR spectra of the **Cu_2OH** complexes **3**-PF_6 (red) and **3**-OTf (black) from Figure S43 from 2000 – 1000 cm^{-1}. The band structure around 1450 cm^{-1} seems to be an artifact from the measurement.

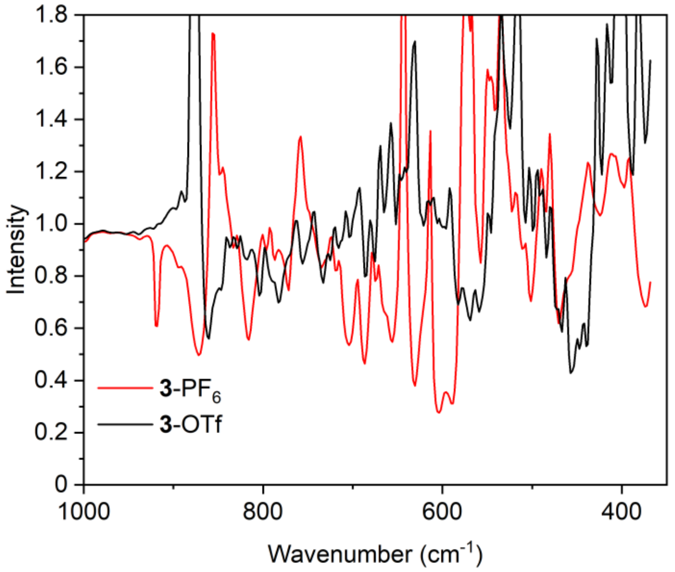

Figure S46: Comparison of the IR spectra of the **Cu_2OH** complexes **3**-PF_6 (red) and **3**-OTf (black) from Figure S43 from 1000 – 350 cm^{-1}.

7.4. IR spectrum of the green species and possible structure

The IR spectrum of the green species (Figures S47 – S50, red) shows two absorptions bands at 3612 cm^{-1} and 3530 cm^{-1}, that lie in the expected range of an O-H band. These bands might origin from a *μ*-hydroxo hydroxo complex **Cu_2(*μ*-OH)OH**, which exhibits two different O-H groups.

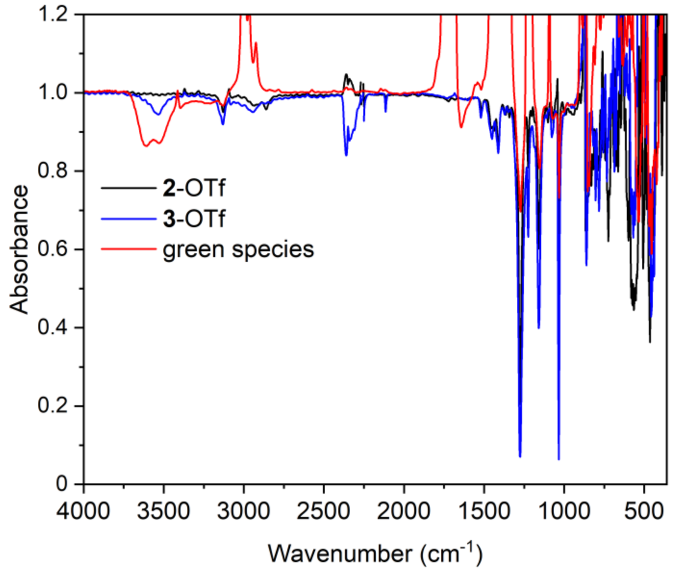

Figure S47: IR spectra of the Cu(I)-complex **2**-OTf (black) the **Cu_2OH** species **3**-OTf (blue) and green species obtained after oxygenation of **2**-OTf with O_2 at room temperature (red).

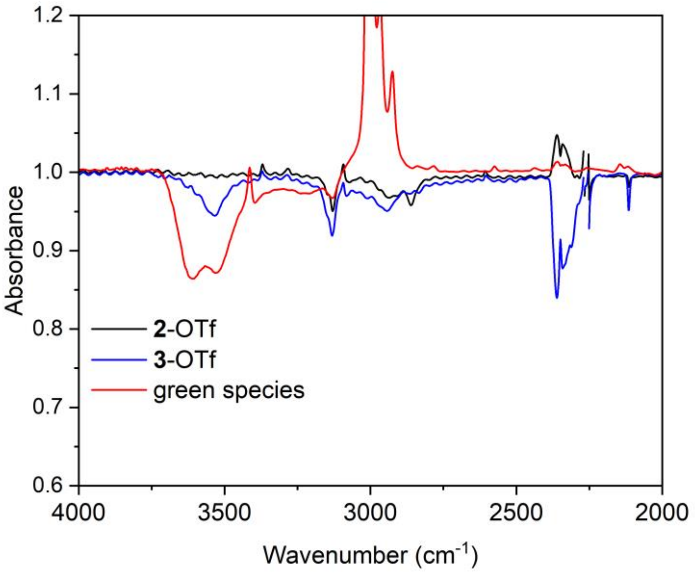

Figure S48: IR spectra of the Cu(I)-complex **2**-OTf (black) the **Cu_2OH** species **3**-OTf (blue) and green species obtained after oxygenation of **2**-OTf with O_2 at room temperature (red) from Figure S47 from 4000 – 2000 cm^{-1}.

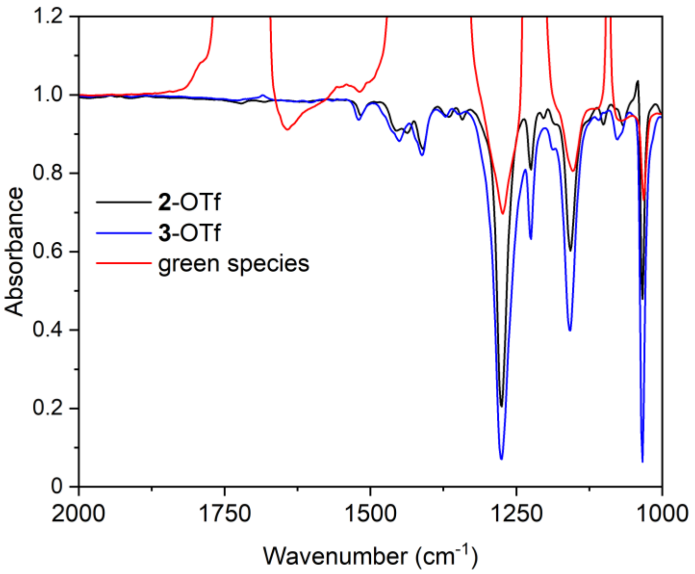

Figure S49: IR spectra of the Cu(I)-complex **2**-OTf (black) the **Cu_2OH** species **3**-OTf (blue) and green species obtained after oxygenation of **2**-OTf with O_2 at room temperature (red) from Figure S47 from 2000 – 1000 cm^{-1}.

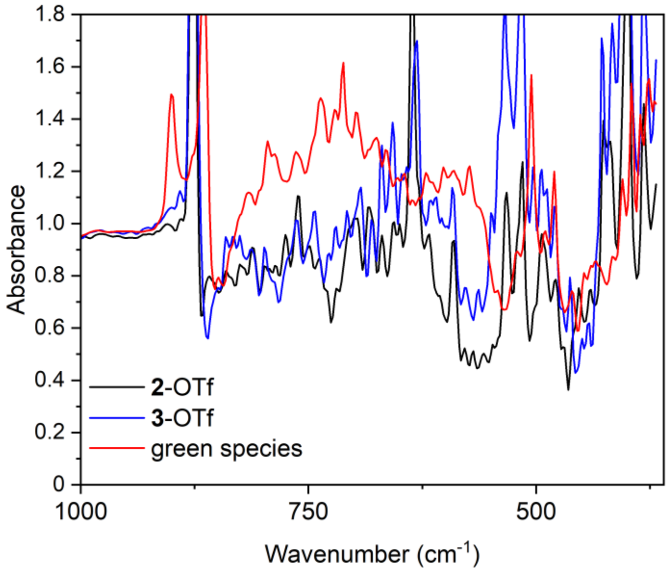

Figure S50: IR spectra of the Cu(I)-complex **2**-OTf (black) the **Cu_2OH** species **3**-OTf (blue) and green species obtained after oxygenation of **2**-OTf with O_2 at room temperature (red) from Figure S47 from 1000 – 350 cm^{-1}.

To support the assumption of the **Cu_2(*μ*-OH)OH** species, we performed DFT calculations of this intermediate. The geometry optimization (Figure S51) indicates that, in principle, such an intermediate can be stabilized with our **MO8** ligand system and the calculated frequencies for the O-H vibrations of the **Cu_2(*μ*-OH)OH** species are in good agreement with the possible O-H bands of the green species (see Table S8).

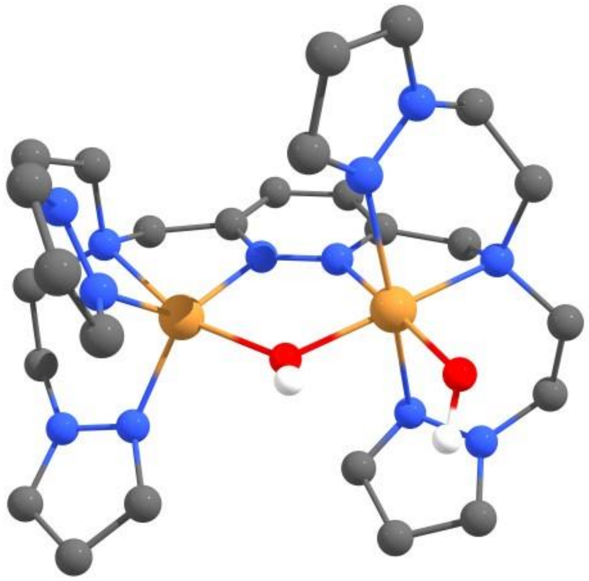

Figure S51: Geometry optimization of the **Cu_2(*μ*-OH)OH** complex. All hydrogen atoms attached to carbon atoms have been omitted for clarity. DFT: RI-PBE-D3(BJ)/def2-TZVP.

Table S8: Comparison of the DFT calculated O-H IR-vibrations for the **Cu_2(*μ*-OH)OH** complex with the observed frequencies of the green species. DFT: RI-PBE-D3(BJ)/def2-TZVP.

**Vibration mode / cm^{-1}**	
**DFT<br>Cu_2(*μ*-OH)OH**	**experimental data<br>green species**
$\nu_{\mu\text{-O-H}}$ / 3630	3530
$\nu_{\text{O-H}}$ / 3661	3612

8. HR ESI Mass Spectrometry

8.1. Mass spectrum of the Cu_2OH complex 3-OTf

The mass spectra obtained are strongly dependent on the solvent in which **3** is dissolved. When **3**-OTf is dissolved in MeOH for the measurement, the main species in the mass spectrum can be assigned to a methanolate complex [**2**(MeO)-H]$^{2+}$ (calc. *m/z* 335.0858, obs. *m/z* 335.0853). In addition, a **Cu_2O** complex [**4**]$^{2+}$ (calc. *m/z* 328.0780, obs. *m/z* 328.0774) and the Cu(I) complex [**2**]$^{2+}$ (calc. *m/z* 320.0805, obs. *m/z* 320.0801) can be detected (Figure S52-S55). A peak indicating the presence of the **Cu_2OH** complex [**3**]$^{3+}$ (calc. *m/z* 219.0544, obs. *m/z* 219.0541) can also be found, but the intensity is very low and not all expected isotopes can be detected (Figure S56). Under the conditions in the spectrometer, the **Cu_2OH** complex **3** might be deprotonated, resulting in the formation of **Cu_2O** complex **4**. Also the hydroxo ligand might be removed in the mass spectrometer, leading to the Cu(I) complex **2**.

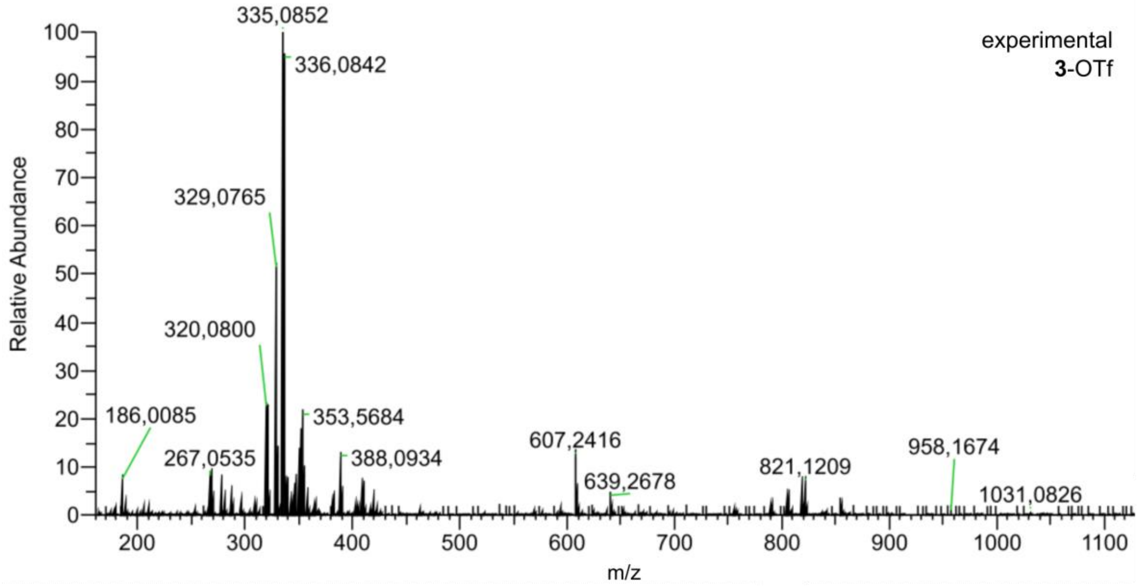

Figure 52: HR-ESI mass spectrum of **3**-OTf when the complex is dissolved in MeOH for the measurement.

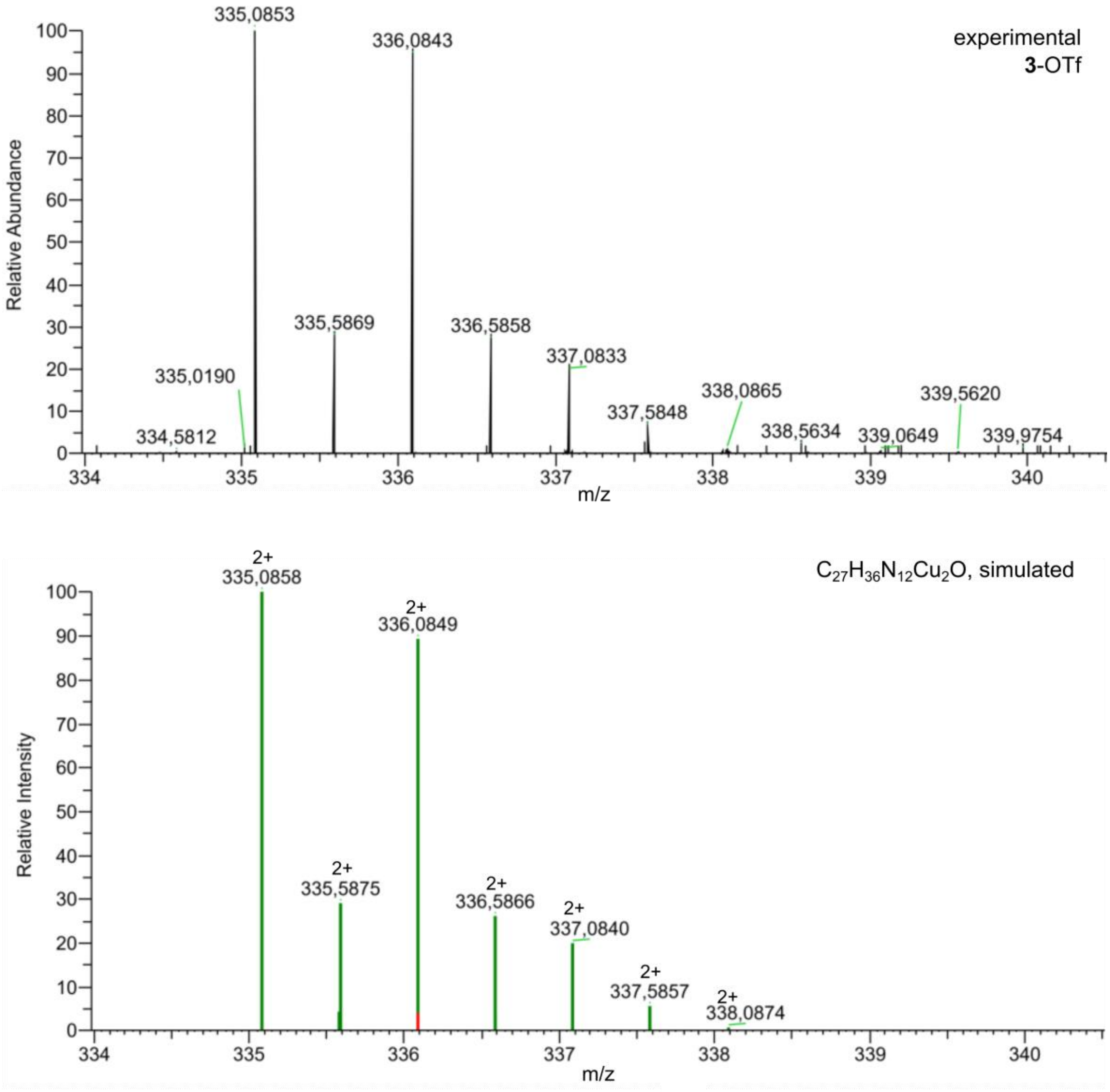

Figure 53: HR-ESI mass spectrum of **3**-OTf. The isotopic pattern and corresponding *m/z* value (top, experimental spectrum) match the simulated spectrum of the $[Cu_2(\textbf{MO8})(MeO)\text{-}H]^{2+}$ species on the bottom. This species might be formed in the mass spectrometer, as the complex was dissolved in methanol for the measurement.

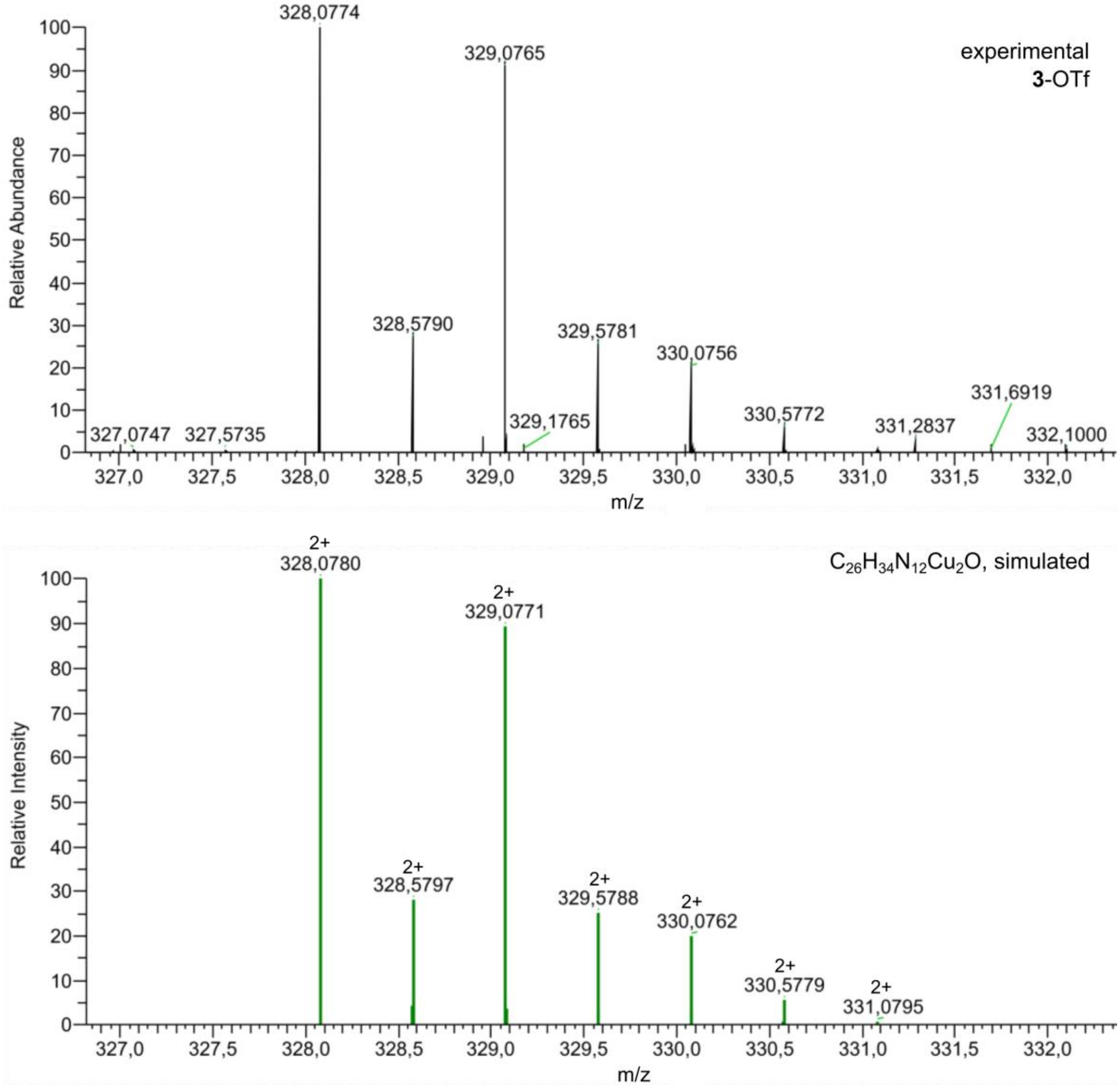

Figure 54: HR-ESI mass spectrum of **3**-OTf. The isotopic pattern and corresponding *m/z* value (top, experimental spectrum) match the simulated spectrum for the **Cu_2O** species [**4**]$^{2+}$ (bottom, simulated spectrum).

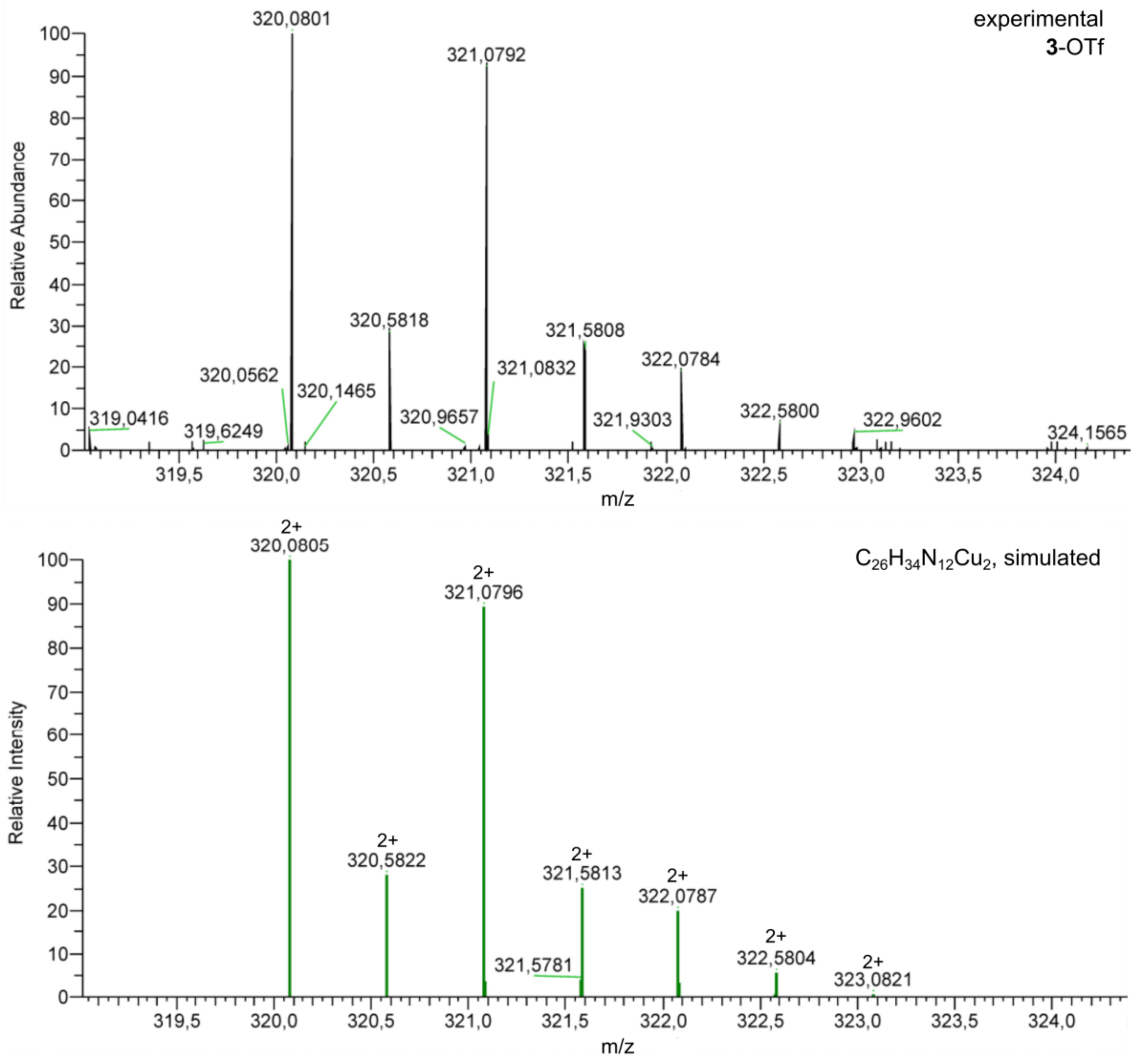

Figure 55: HR-ESI mass spectrum of **3**-OTf. The isotopic pattern and corresponding *m/z* value (top, experimental spectrum) match the simulated spectrum for the Cu(I) complex [**2**]$^{2+}$ (bottom, simulated spectrum).

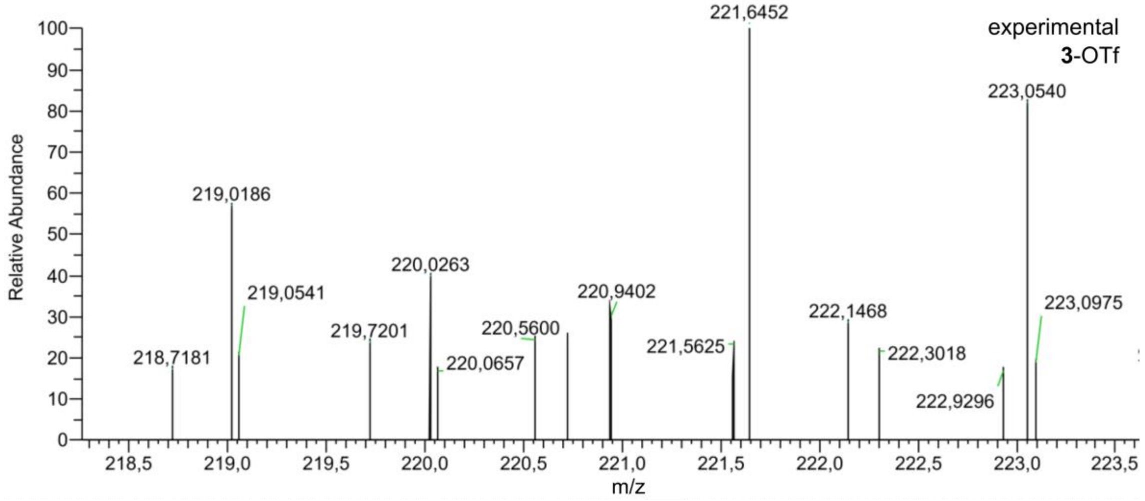

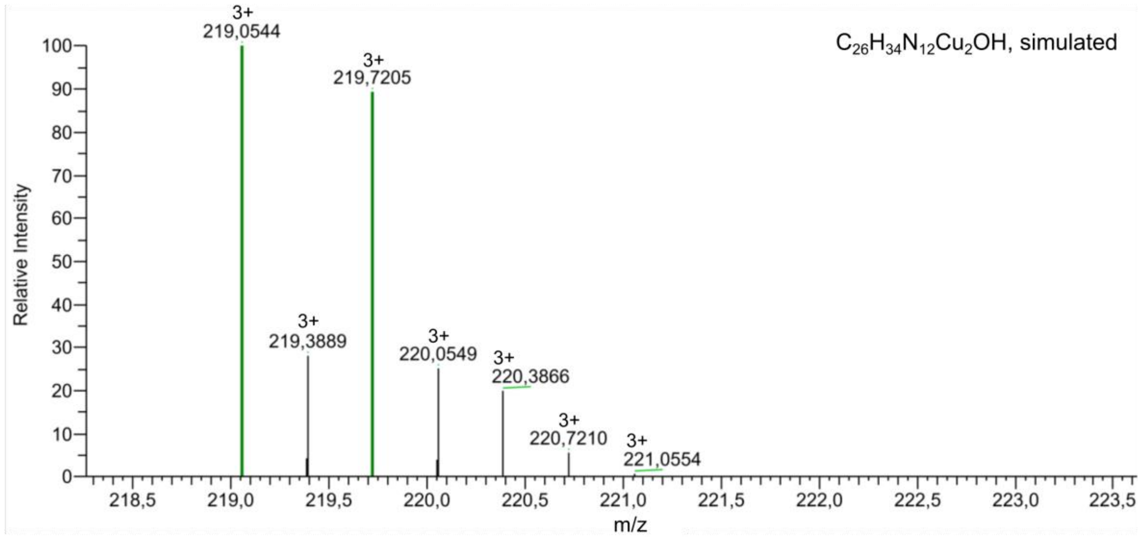

Figure 56: HR-ESI mass spectrum of **3**-OTf. The isotopic pattern and corresponding *m/z* value (top, experimental spectrum) match the simulated spectrum for the **Cu_2OH** species [**3**]$^{3+}$ (bottom, simulated spectrum).

If the complex **3**-OTf is dissolved in acetonitrile instead of MeOH for the ESI-MS measurement, the peak at m/z = 335.0853 (Figure S57) is not observed. Instead, a peak at m/z = 213,0506 is detected, with an isotopic pattern that is very consistent with a $[Cu_2C_{26}H_{33}N_{12}]^{+3}$ species, i.e. a **Cu_2OH** complex that might have eliminated H_2O under the conditions of the mass spectrometer [**3**-H_2O]$^{3+}$.

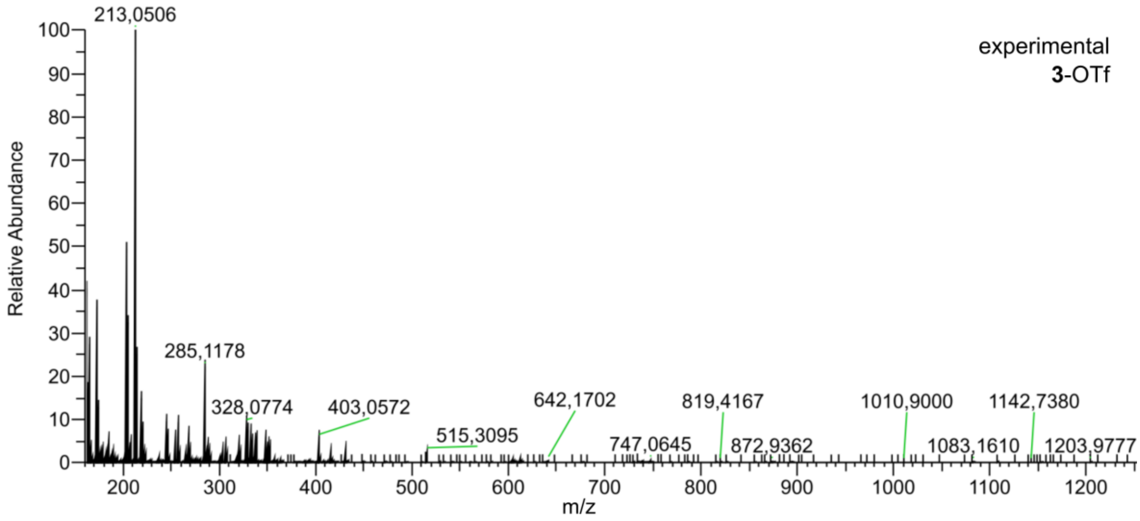

Figure 57: HR-ESI mass spectrum of **3**-OTf when the complex is dissolved in acetonitrile for the measurement.

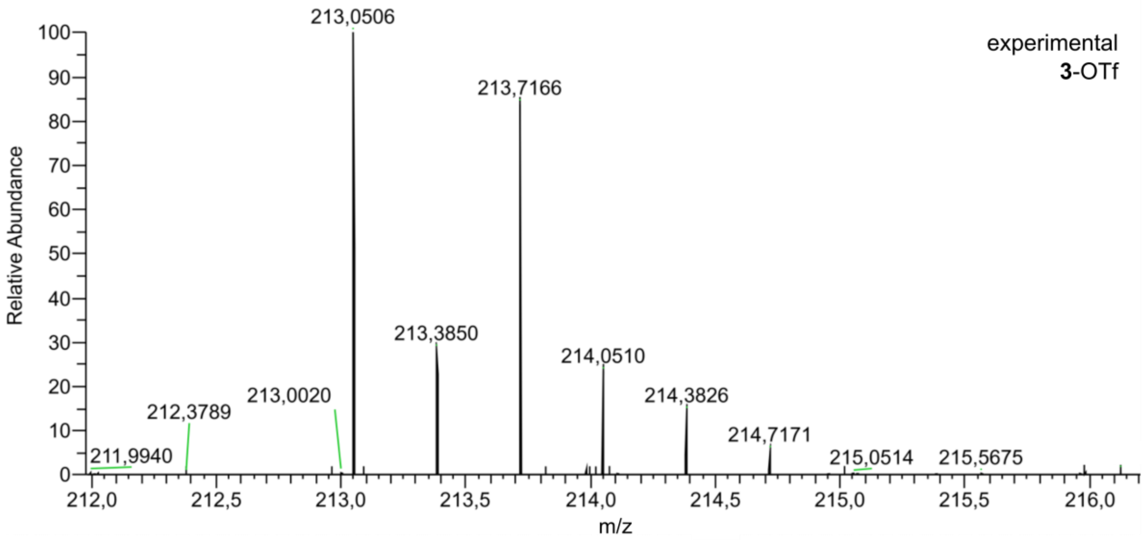

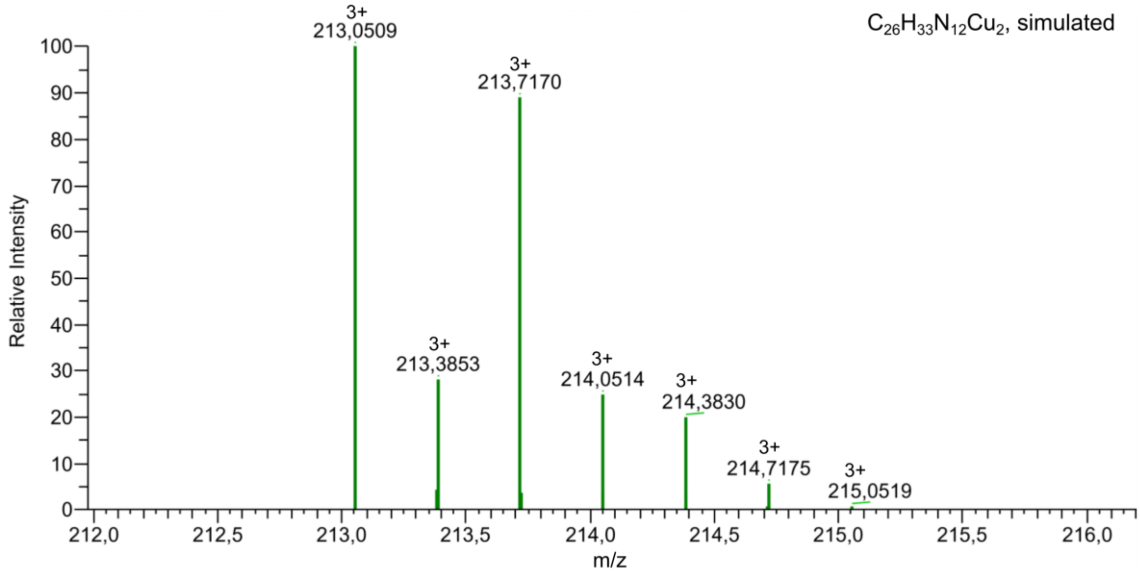

Figure 58: HR-ESI mass spectrum of **3**-OTf dissolved in acetonitrile. The isotopic pattern and corresponding *m/z* value (top, experimental spectrum) match the simulated spectrum for the $[Cu_2C_{26}H_{33}N_{12}]^{3+}$ species $[\mathbf{3}\text{-}H_2O]^{3+}$ (bottom, simulated spectrum).

In the mass spectrum of the **Cu_2OH** complex **3**-OTf dissolved in acetonitrile, the Cu(I) complex **2** and the **Cu_2O** species **4** are detectable as in the complex dissolved in MeOH (Figure S59 and Figure S60). In addition, the **Cu_2OH** complex can also be detected in acetonitrile (Figure S61).

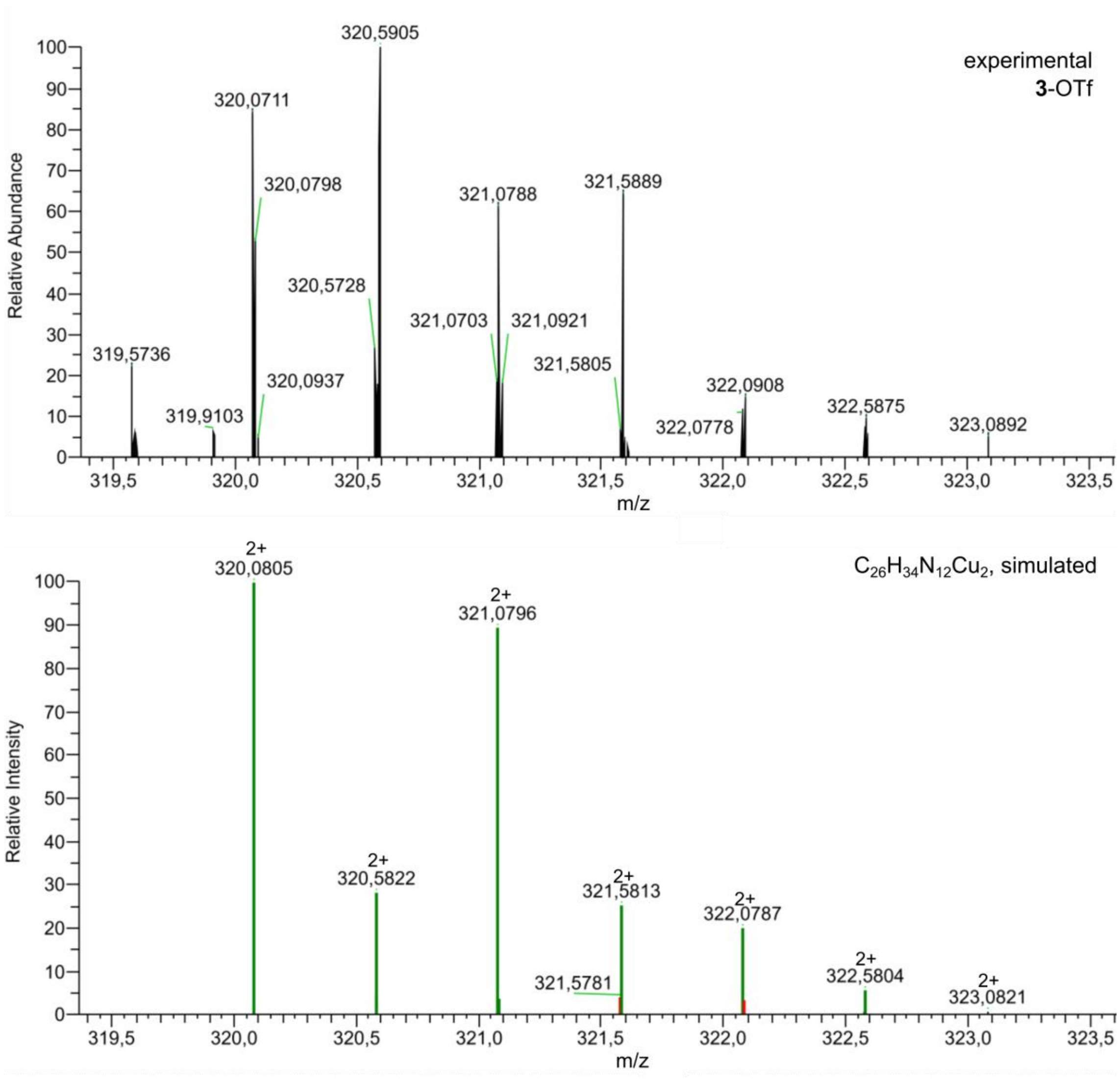

Figure 59: HR-ESI mass spectrum of **3**-OTf dissolved in acetonitrile. The isotopic pattern and corresponding *m/z* value (top, experimental spectrum) match the simulated spectrum for the Cu(I) complex **2** (bottom, simulated spectrum).

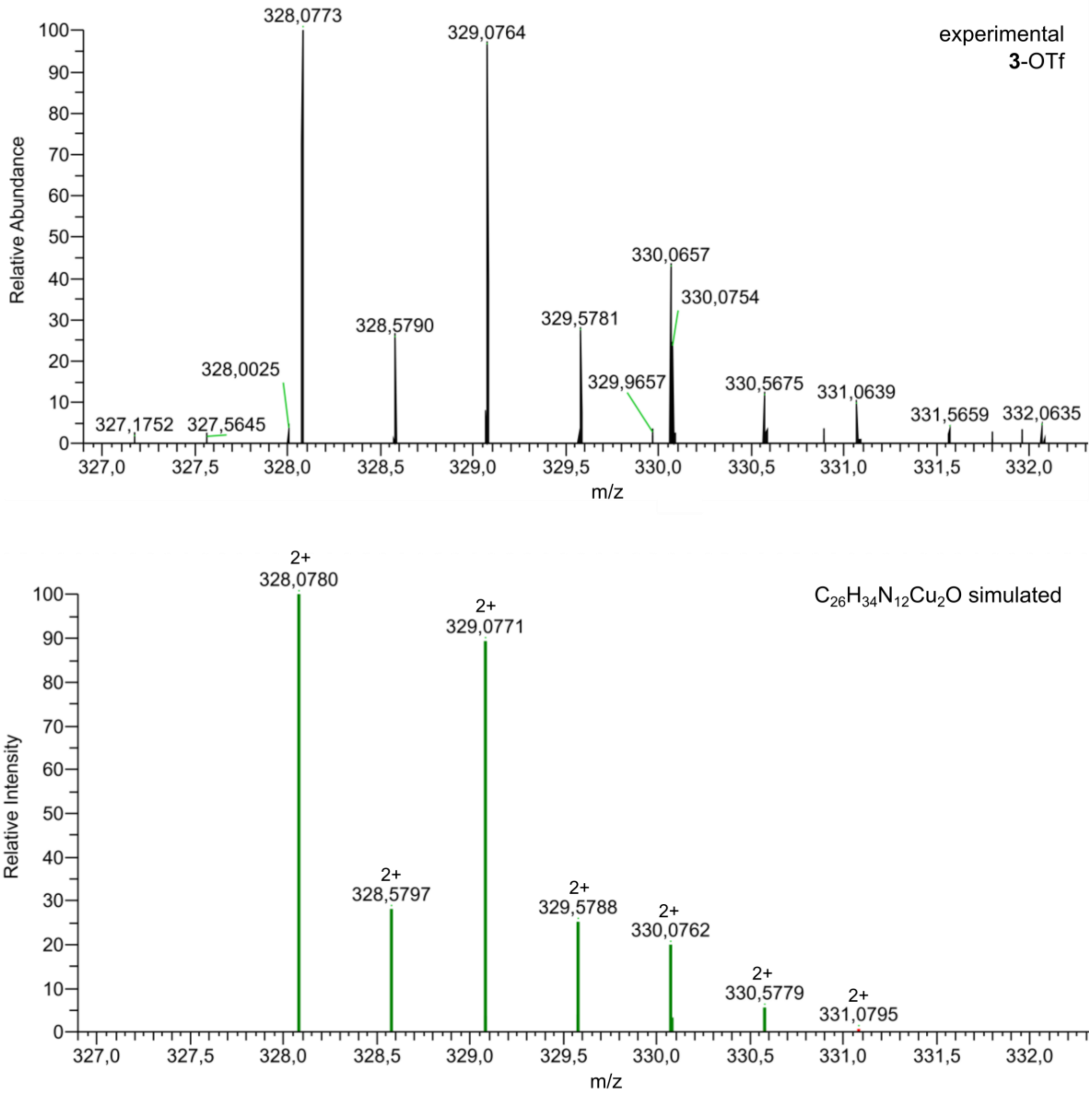

Figure 60: HR-ESI mass spectrum of **3**-OTf dissolved in acetonitrile. The isotopic pattern and corresponding *m/z* value (top, experimental spectrum) match the simulated spectrum for a **Cu_2O** species **4** (bottom, simulated spectrum).

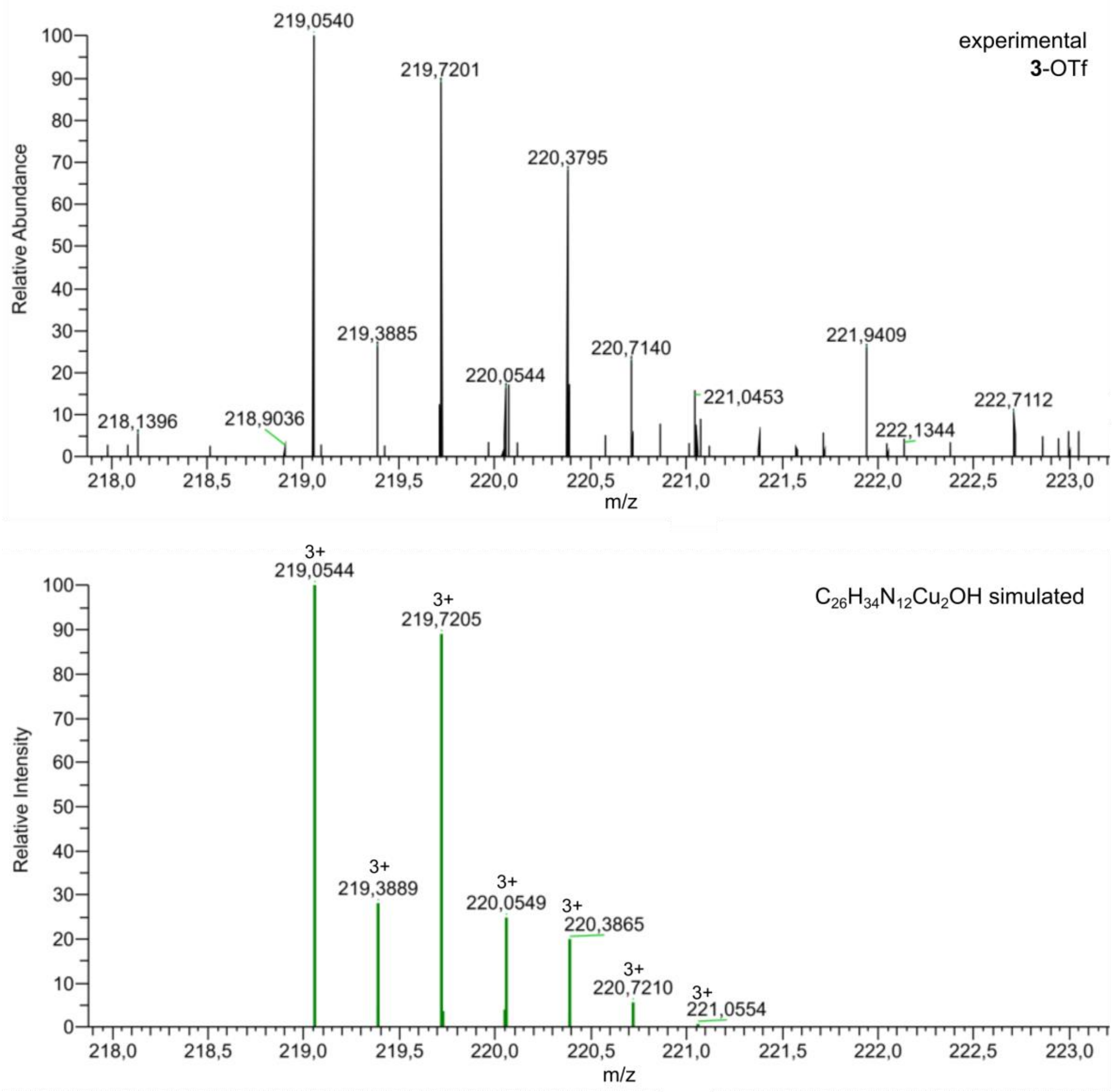

Figure 61: HR-ESI mass spectrum of **3**-OTf dissolved in acetonitrile. The isotopic pattern and corresponding *m/z* value (top, experimental spectrum) match the simulated spectrum for the **Cu_2OH** species **3** (bottom, simulated spectrum).

8.2. Mass spectrum of the Cu_2OH complex 3-PF_6

The **Cu_2OH** complex **3** can also be detected in the mass spectrum of **3**-PF_6 (Figure S63). In addition a species with one hexafluorophosphate anion and two hexafluorophosphate anions can be found (Figure S64 + S65). The **Cu_2O** species [**4**]$^{2+}$ is also present in the mass spectrum of **3**-PF_6 (Figure S66). In contrast to the **Cu_2OH** complex **3**-OTf, there is a peak at m/z = 288,6150 in the mass spectrum of **3**-PF_6 which matches a [**2**-Cu]$^{2+}$ species (Figure S67).

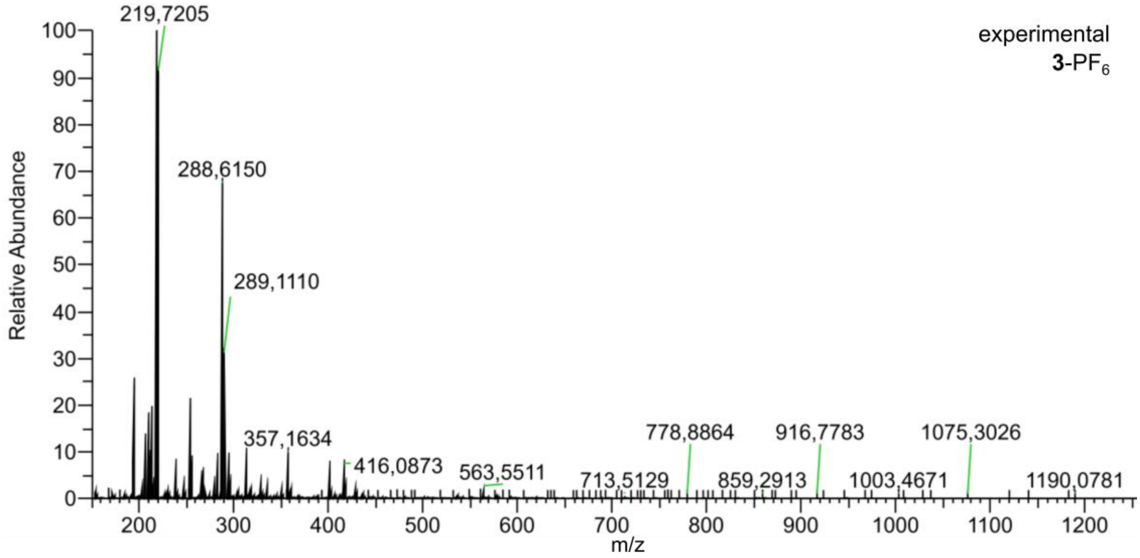

Figure 62: HR-ESI mass spectrum of **3**-PF_6 dissolved in acetonitrile for the measurement.

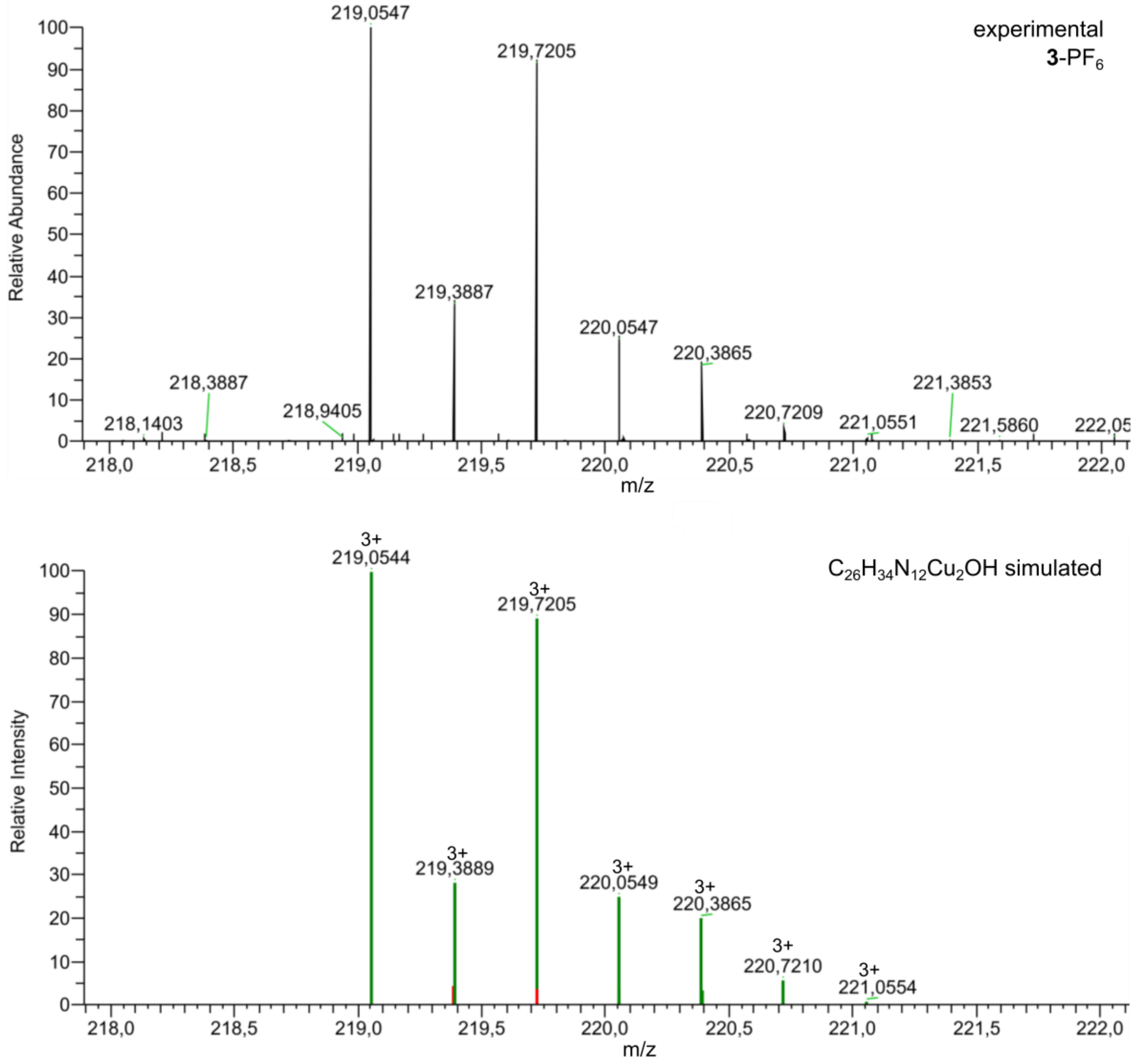

Figure 63: HR-ESI mass spectrum of **3**-PF_6. The isotopic pattern and corresponding *m/z* value (top, experimental spectrum) match the simulated spectrum for a **Cu_2OH** species (bottom, simulated spectrum).

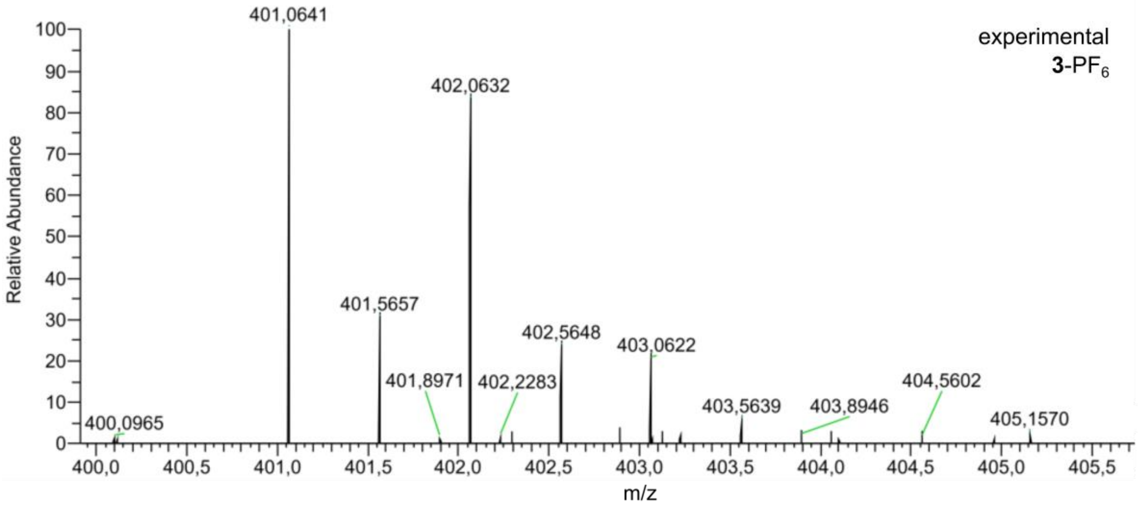

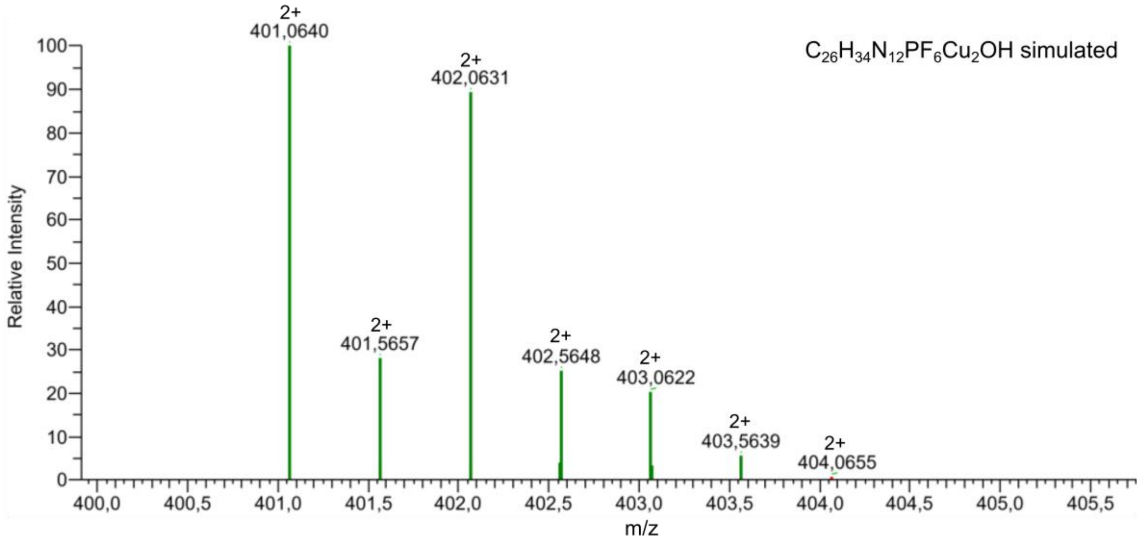

Figure 64: HR-ESI mass spectrum of **3**-PF_6. The isotopic pattern and corresponding *m/z* value (top, experimental spectrum) match the simulated spectrum for a **Cu_2OH** species with one hexafluorophosphate anion (bottom, simulated spectrum).

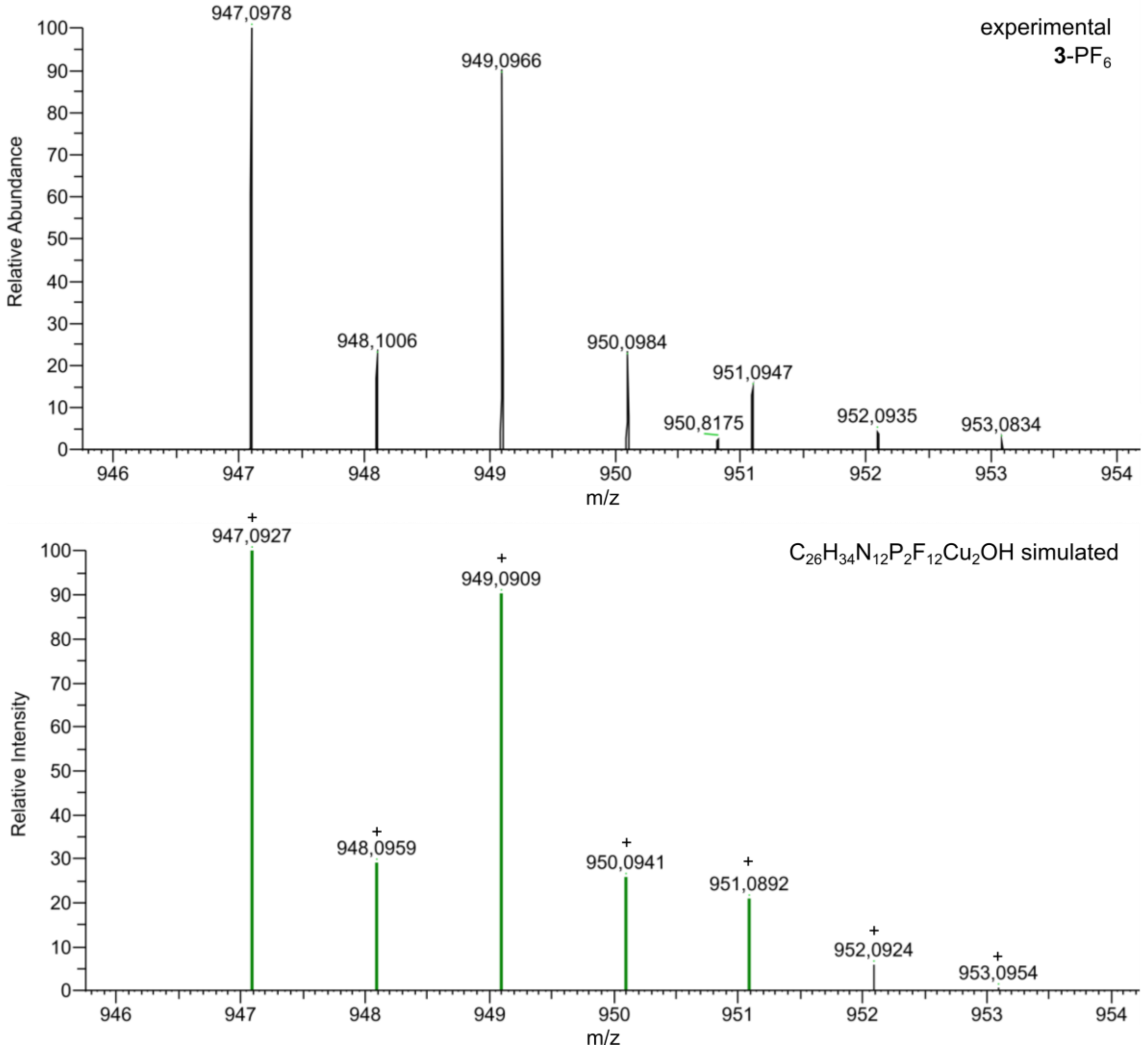

Figure 65: HR-ESI mass spectrum of **3**-PF_6. The isotopic pattern and corresponding *m/z* value (top, experimental spectrum) match the simulated spectrum for a $\mathbf{Cu_2OH}$ species with two hexafluorophosphate anions (bottom, simulated spectrum).

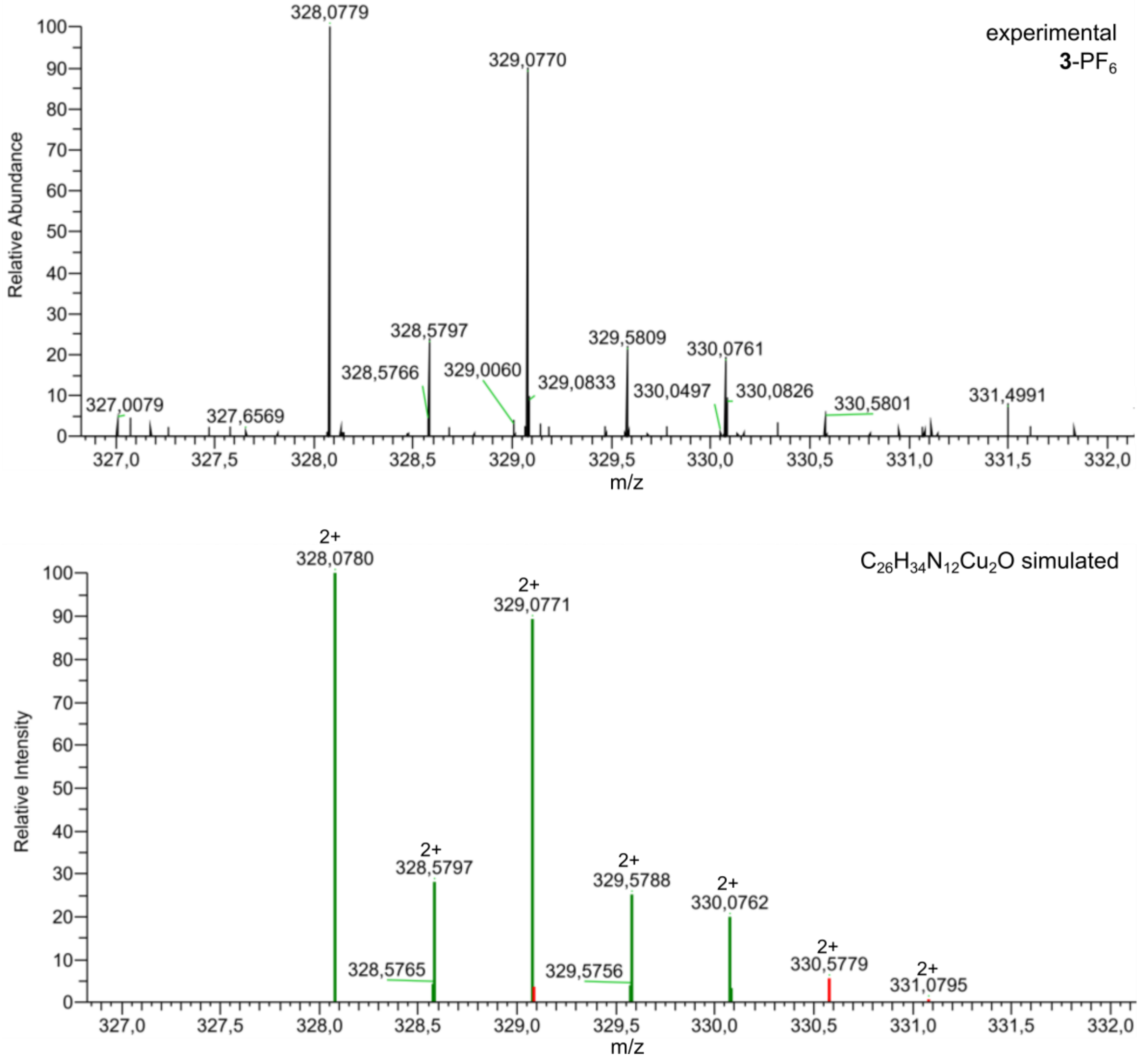

Figure 66: HR-ESI mass spectrum of **3**-PF_6. The isotopic pattern and corresponding *m/z* value (top, experimental spectrum) match the simulated spectrum for a **Cu_2O** species [**4**]$^{2+}$ (bottom, simulated spectrum).

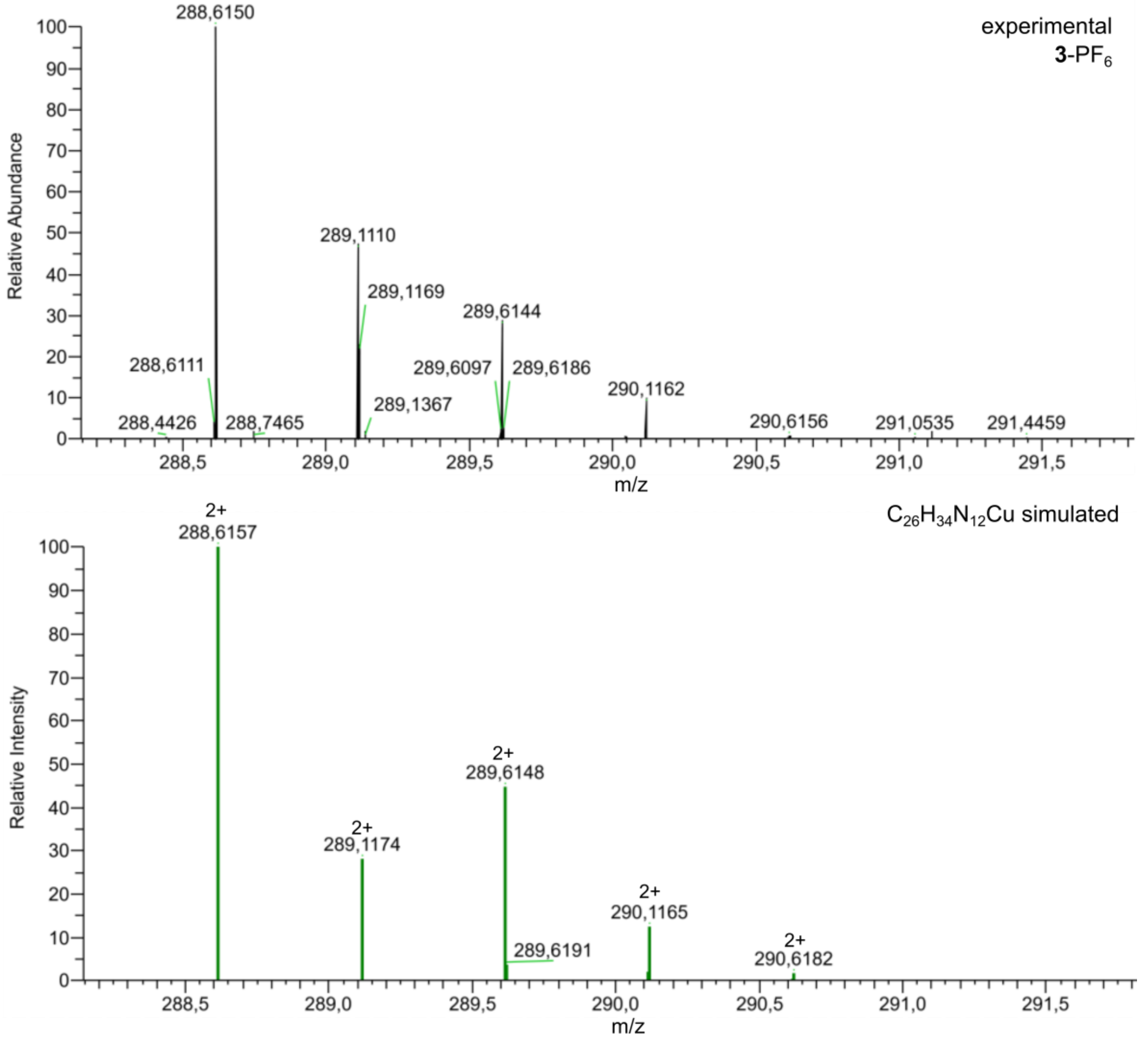

Figure 67: HR-ESI mass spectrum of **3**-PF_6. The isotopic pattern and corresponding *m/z* value (top, experimental spectrum) match the simulated spectrum for a [**2**-Cu]$^{2+}$ species (bottom, simulated spectrum).

9. Cryo-UHR-ESI Mass Spectrometry

9.1. Obtained mass spectra upon reaction with $^{16}O_2$ at 183 K

In contrast to the characteristic UV/Vis and rRaman spectra of the **Cu_2OOH** complex **3**, we do not have an UHR-ESI mass spectrum of this species. Instead, a **Cu_2O_2** species could be detected, which is superimposed by a mixed-valent *μ*-chlorido species. However, the origin of the chloride is unknown. Furthermore, a **Cu_2O** species [**4**]$^{2+}$ could be detected under these conditions.

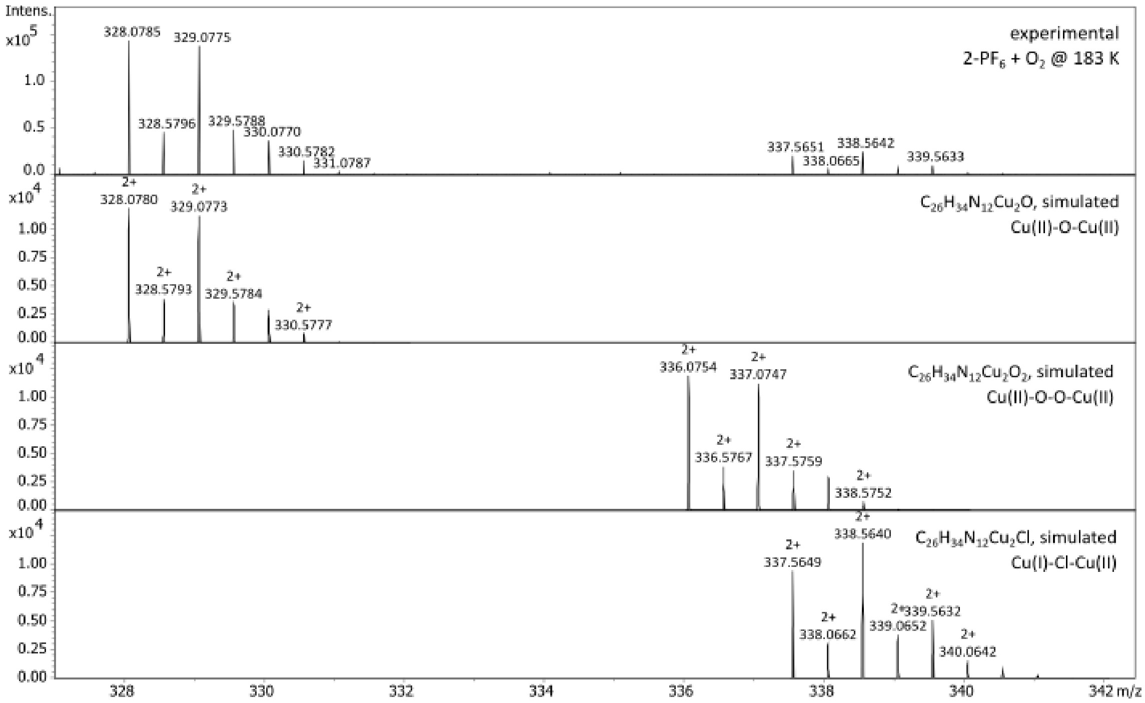

Figure S68: Cryo-UHR-ESI mass spectrum of **2**-PF_6 after reaction with $^{16}O_2$ at 183 K in acetone. The isotopic pattern and corresponding *m/z* value (top, experimental spectrum) match the simulated spectrum for a **Cu_2O** species [**4**]$^{2+}$ (middle, simulated spectrum). The detected pattern for the **Cu_2O_2** complex is a superposition of the **Cu_2O_2** (middle, simulated spectrum) and the mixed-valent mono-*μ*-chlorido copper(I) copper(II) complex (bottom, simulated spectrum). The same result was obtained for **2**-OTf (not shown).

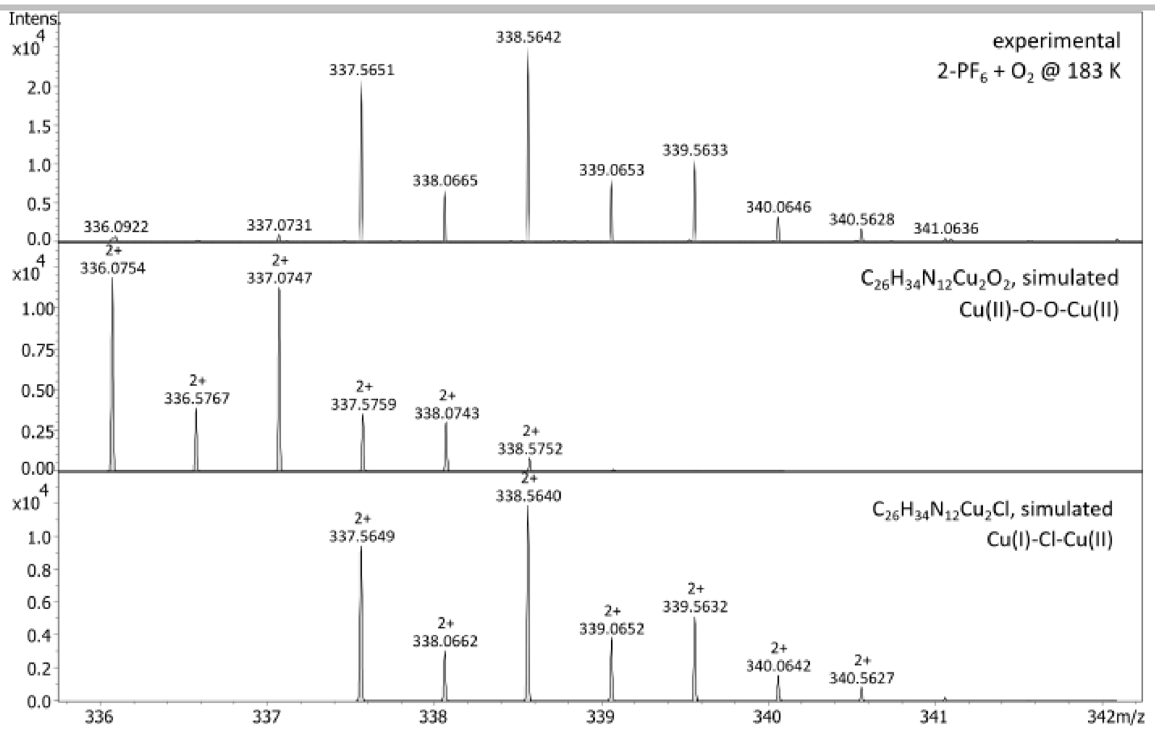

Figure S69: Cryo-UHR-ESI mass spectrum of **2**-PF_6 after reaction with $^{16}O_2$ at 183 K in acetone. The isotopic pattern and corresponding *m/z* value (top, experimental spectrum) match a superposition of the $\mathbf{Cu_2O_2}$ (middle, simulated spectrum) and the mixed-valent mono-*µ*-chlorido copper(I) copper(II) complex (bottom, simulated spectrum). The same result was obtained for **2**-OTf (not shown).

9.2. Obtained mass spectra upon reaction with $^{16}O_2$ and $^{18}O_2$ at room temperature

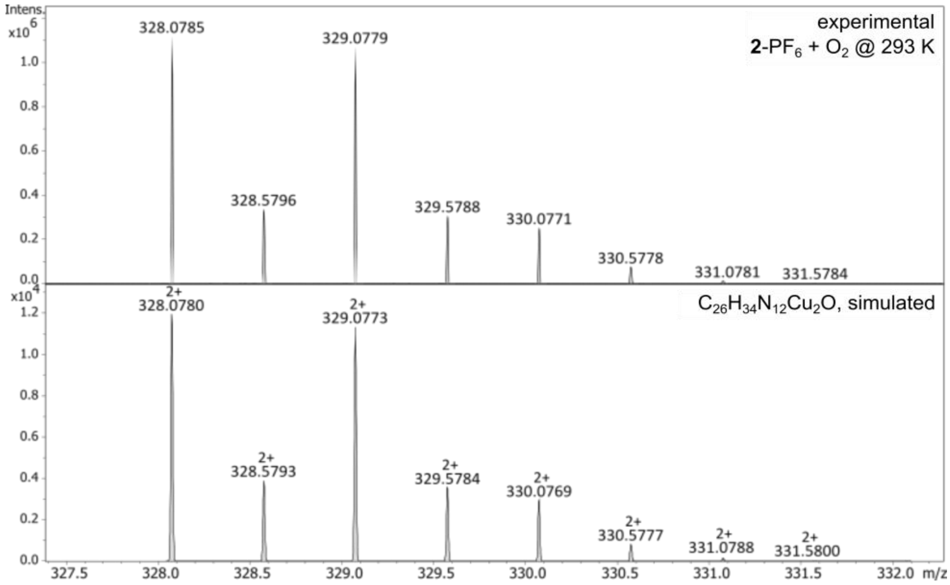

Figure S70: Cryo-UHR-ESI mass spectrum of **2**-PF_6 after reaction with $^{16}O_2$ at room temperature in acetone. The isotopic pattern and corresponding *m/z* value (top, experimental spectrum) match the simulated spectrum for a $\mathbf{Cu_2O}$ species [**4**]$^{2+}$ (bottom, simulated spectrum).

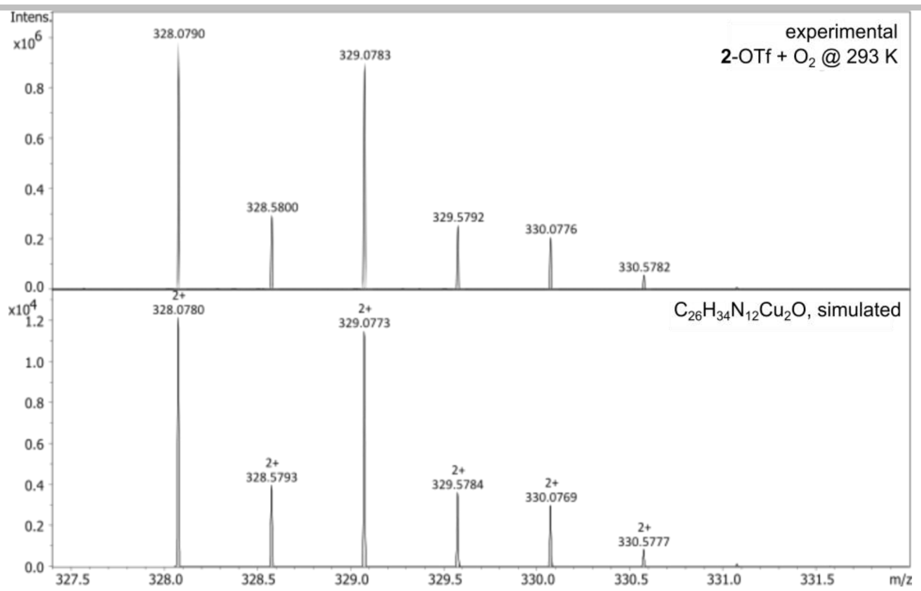

Figure S71: Cryo-UHR-ESI mass spectrum of **2**-OTf after reaction with $^{16}O_2$ at room temperature in acetone. The isotopic pattern and corresponding *m/z* value (top, experimental spectrum) match the simulated spectrum for a **Cu₂O** species [**4**]$^{2+}$ (bottom, simulated spectrum).

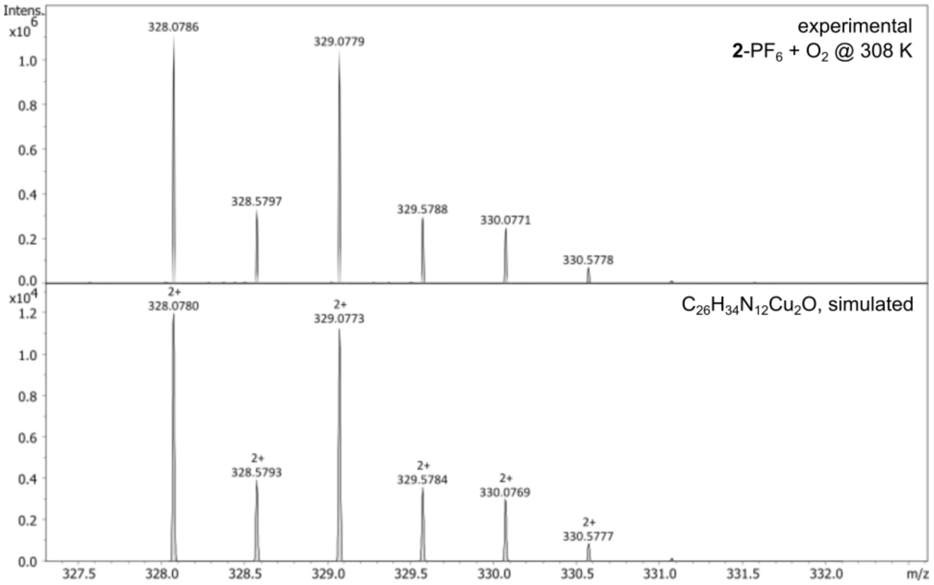

Figure S72: Cryo-UHR-ESI mass spectrum of **2**-PF_6 after reaction with $^{16}O_2$ at 308 K in acetone. The isotopic pattern and corresponding *m/z* value (top, experimental spectrum) match the simulated spectrum for a **Cu_2O** species [**4**]$^{2+}$ (bottom, simulated spectrum). The same result was obtained for **2**-OTf (not shown).

We assumed that acetone causes an exchange of the *μ*-oxo ligand of the **Cu_2O** species with oxygen from the acetone solvent. To confirm our hypothesis, we also recorded an EI mass spectrum. We were able to detect small amounts of ^{18}O-acetone (*m/z* 60 together with the characteristic fragments *m/z* 45 and *m/z* 30) as well as much ^{16}O-acetone (*m/z* 58 together with the characteristic fragments *m/z* 43 and *m/z* 28) by use of EI-MS. Nevertheless, to confirm our assumption by UHR-ESI-MS, we had to change the solvent. Instead of acetone, we tested acetonitrile. We had seen evidence that the mono-*μ*-oxo dicopper(II) complex is also formed in this solvent by UV/Vis spectroscopy (Figure S32). Figure S73 shows the cutoff of the UHR-ESI mass spectrum of **2**-PF_6 after reaction with $^{18}O_2$ at room temperature in acetonitrile instead of acetone, showing the presence of a **$Cu_2{}^{18}O$** complex.

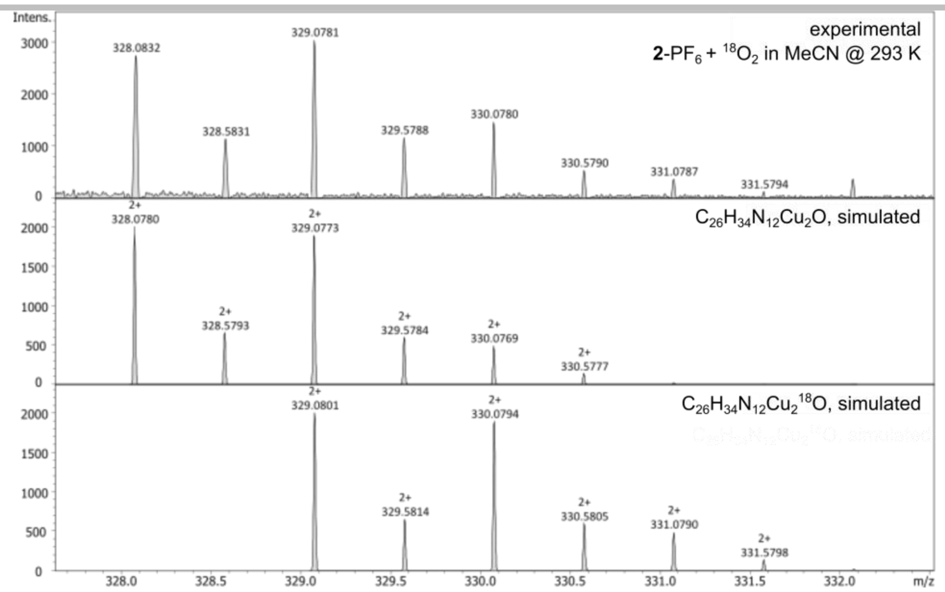

Figure S73: Cryo-UHR-ESI mass spectrum of **2**-PF_6 after reaction with $^{18}O_2$ at room temperature in acetonitrile instead of acetone. The isotopic pattern and corresponding *m/z* value (top, experimental spectrum) match the simulated spectrum for a **$Cu_2{}^{18}O$** species (bottom, simulated spectrum). The detected pattern is a superposition of the **$Cu_2{}^{18}O$** (bottom, simulated spectrum) and **$Cu_2{}^{16}O$** (middle, simulated spectrum) species. The same result was obtained for **2**-OTf (not shown).

9.3. Obtained mass spectra upon reaction with PhIO

After reaction of **2**-PF_6 or **2**-OTf with the oxygen-atom transfer reagent PhIO, a **Cu_2O** species [**4**]$^{2+}$ is present in the mass spectra (Figure S74 and Figure S75).

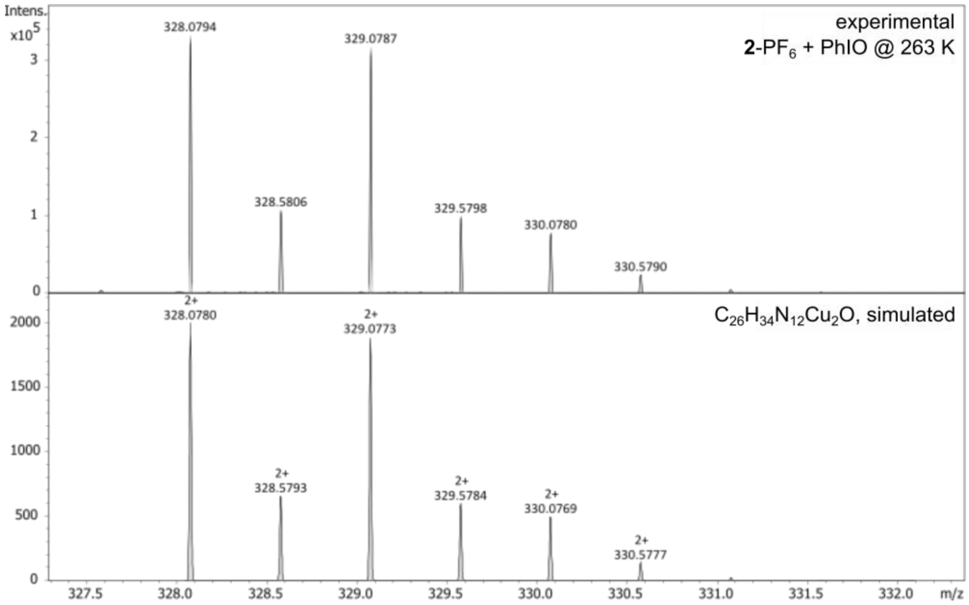

Figure S74: Cryo-UHR-ESI mass spectrum of **2**-PF_6 with excess PhIO at 263 K after 3 h reaction time. The isotopic pattern and corresponding *m/z* value match the simulated spectrum for a **Cu_2O** species.

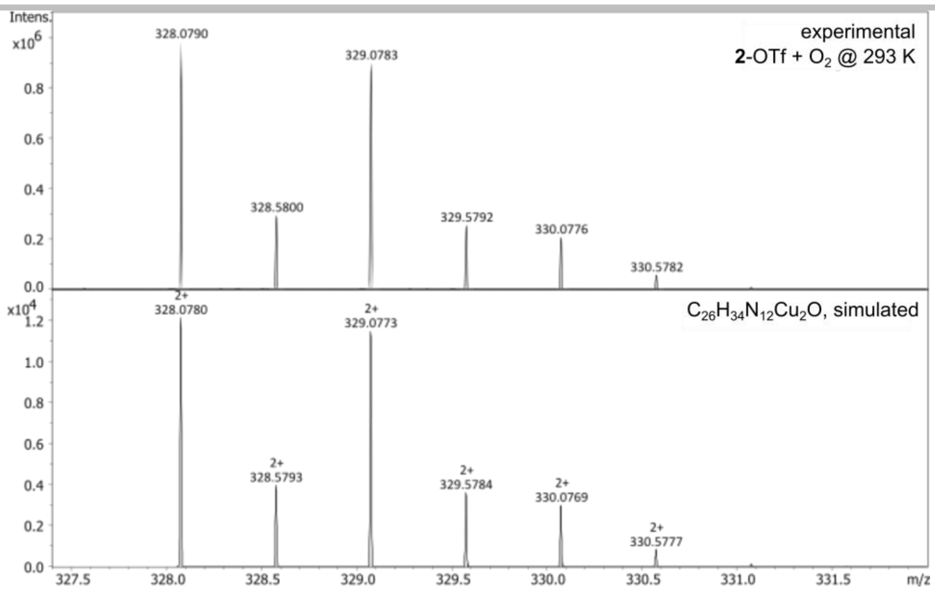

Figure S75: Cryo-UHR-ESI mass spectrum of **2**-OTf with excess PhIO at 263 K after 3 h reaction time. The isotopic pattern and corresponding *m/z* value match the simulated spectrum for a **Cu_2O** species.

9.4. Obtained mass spectra upon reaction with N_2O

When N_2O is used as an oxygen-atom transfer reagent for **2**-PF_6 or **2**-OTf, the **Cu_2O** species [**4**]$^{2+}$ can also be detected in the mass spectra (Figure S76 and Figure S77).

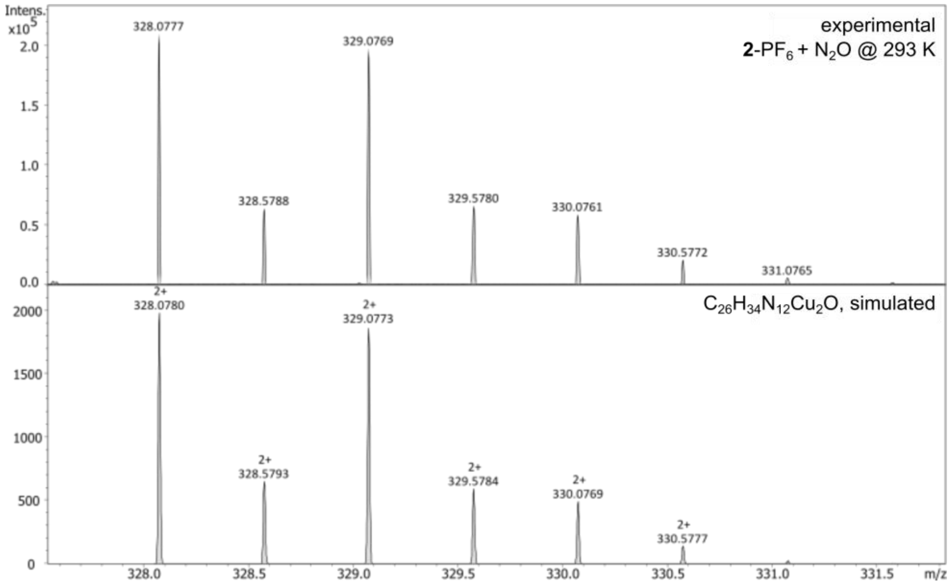

Figure S76: Cryo-UHR-ESI mass spectrum of **2**-PF_6 with N_2O at room temperature. The isotopic pattern and corresponding *m/z* value match the simulated spectrum for a **Cu_2O** species [**4**]$^{2+}$.

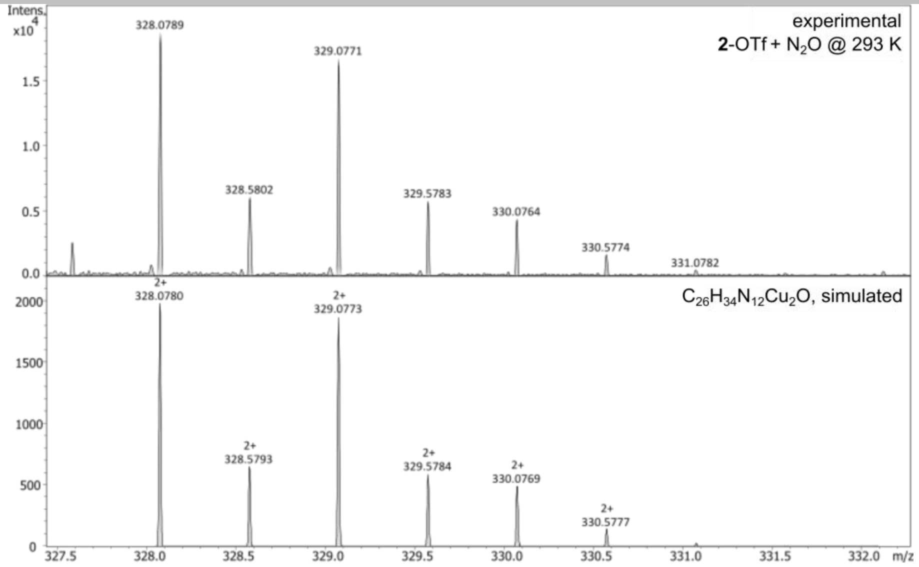

Figure S77: Cryo-UHR-ESI mass spectrum of **2**-OTf with N_2O at room temperature. The isotopic pattern and corresponding *m/z* value match the simulated spectrum for a $\mathbf{Cu_2O}$ species [**4**]$^{2+}$.

9.5. Obtained mass spectra upon reaction with DMDO

The usage of the highly reactive oxygen-atom transfer reagent DMDO lead to a mass spectrum containing a $\mathbf{Cu_2O}$ species [**4**]$^{2+}$, the Cu(I)-complex [**2**]$^{2+}$, a $\mathbf{Cu_2O_2}$ species and a weak peak of the $\mathbf{Cu_2OH}$ species [**3**]$^{3+}$ (Figure S78-S81).

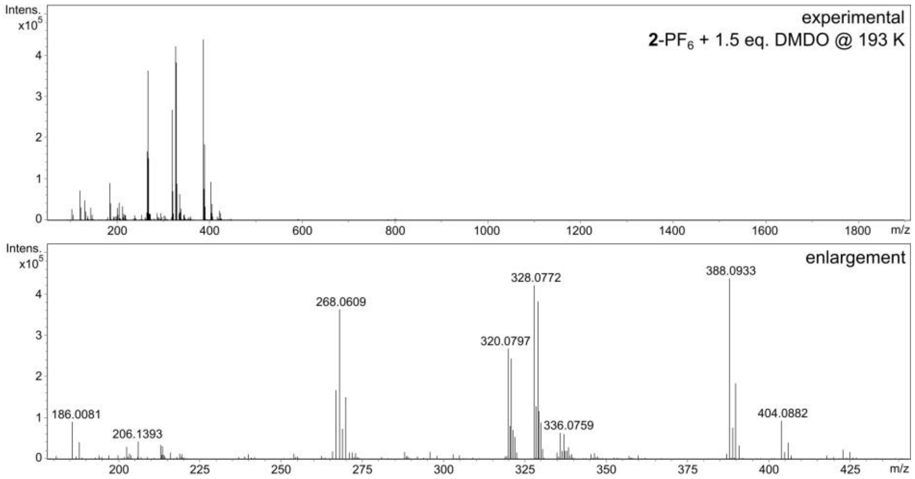

Figure S78: Cryo-UHR-ESI mass spectrum of **2**-PF_6 with 1.5 eq. DMDO at 193 K. Full spectrum (top) and enlargement (bottom).

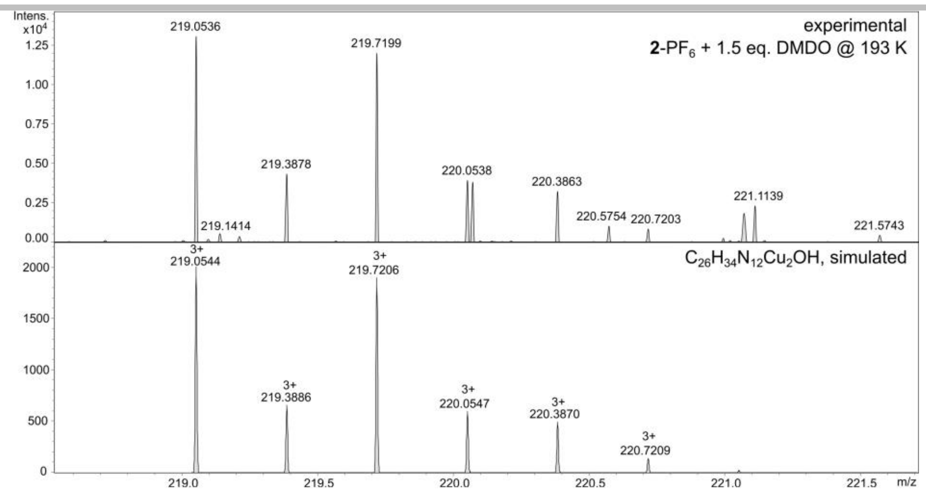

Figure S79: Cryo-UHR-ESI mass spectrum of **2**-PF_6 with DMDO at 193 K. The isotopic pattern and corresponding *m/z* value match the simulated spectrum of the **Cu_2OH** species [**3**]$^{3+}$.

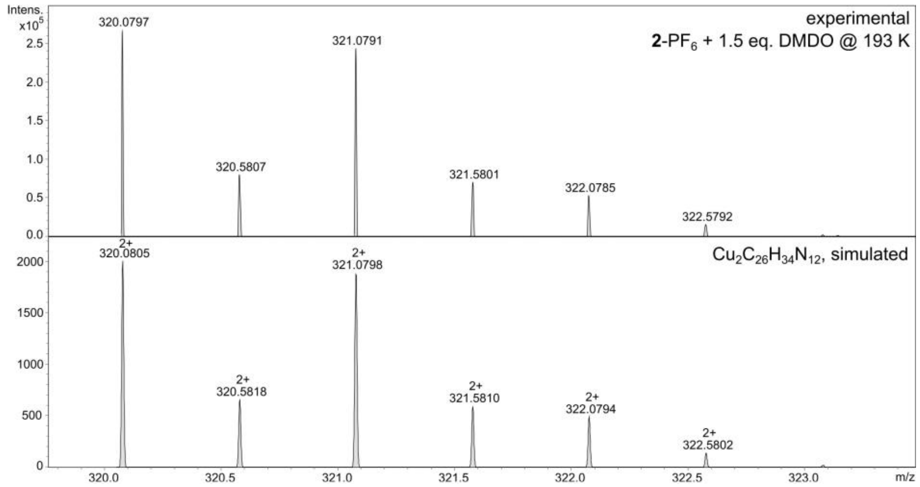

Figure S80: Cryo-UHR-ESI mass spectrum of **2**-PF_6 with DMDO at 193 K. The isotopic pattern and corresponding *m/z* value match the simulated spectrum of the Cu(I)-complex [**2**]$^{2+}$.

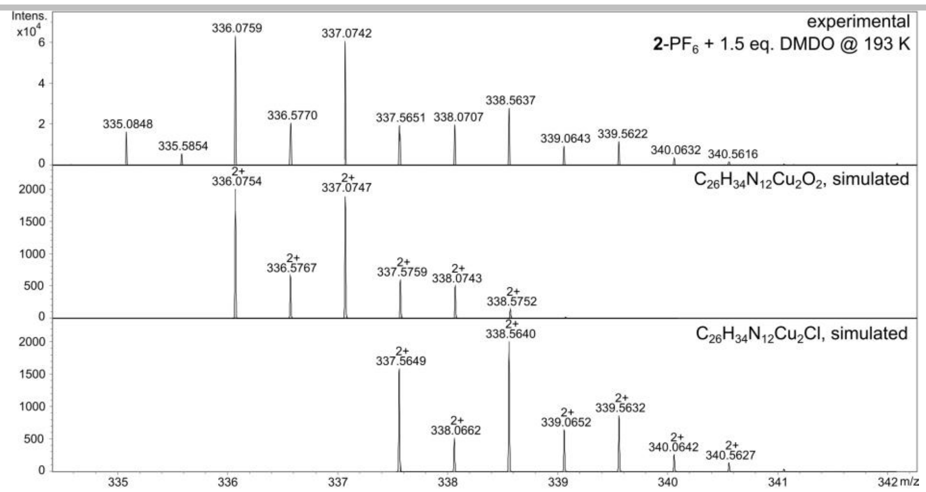

Figure S81: Cryo-UHR-ESI mass spectrum of **2**-PF_6 with DMDO at 193 K. The isotopic pattern and corresponding *m/z* value match the simulated spectrum of a **Cu_2O_2** species (middle) which is superimposed by a **Cu_2Cl** species (bottom).

9.6. Obtained mass spectra upon reaction with $^{16}O_2$ at room temperature and at 308 K: Cu_2O_2 species

Cu_2O_2 species were observed at room temperature and at 308 K upon reaction of **2**-PF_6 or **2**-OTf with dioxygen (Figure S82 – S84).

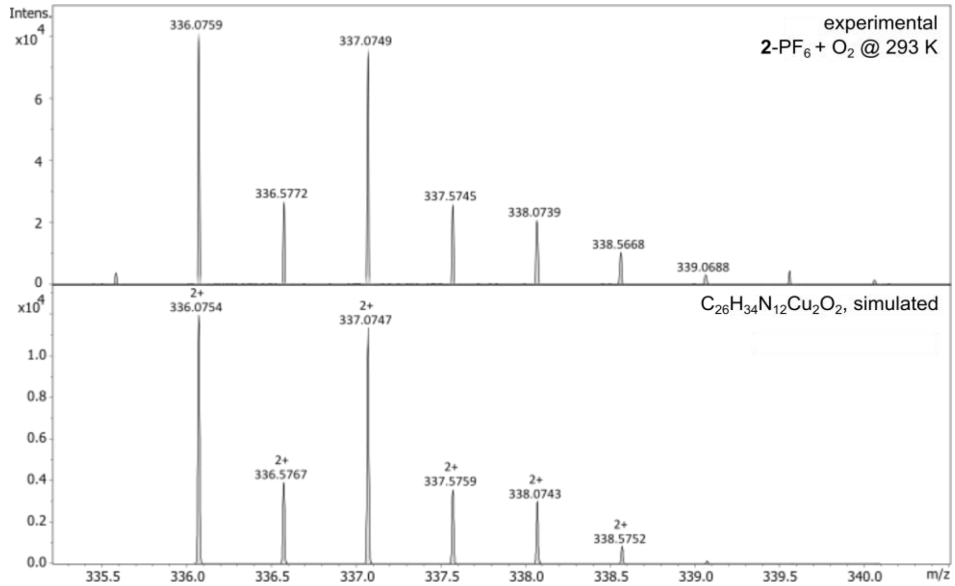

Figure 82: Cryo-UHR-ESI mass spectrum of **2**-PF_6 after reaction with O_2 at room temperature. The isotopic pattern and corresponding *m/z* value match the simulated spectrum for a **Cu_2O_2** species.

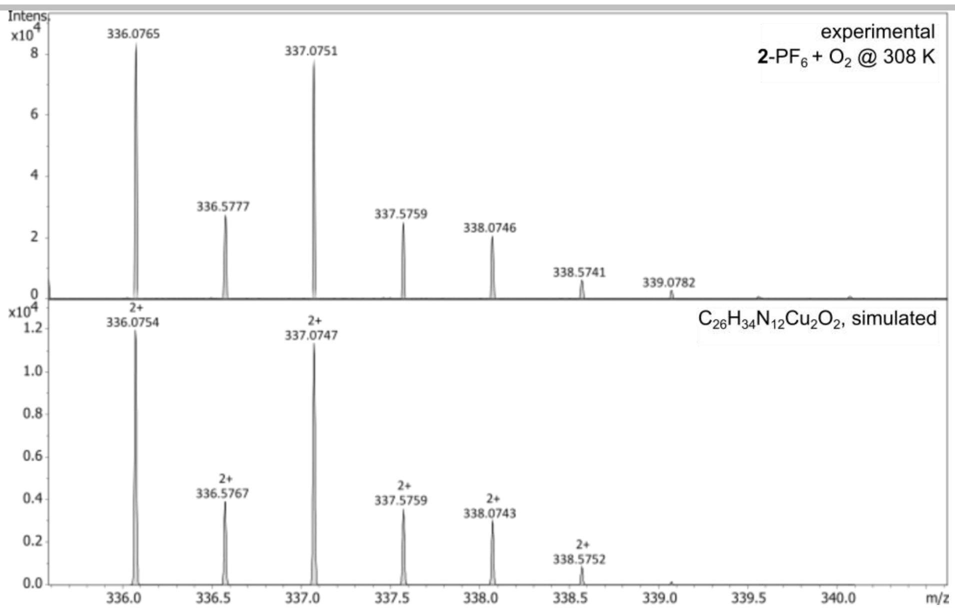

Figure S83: Cryo-UHR-ESI mass spectrum of **2**-PF_6 after reaction with O_2 at 308 K. The isotopic pattern and corresponding *m/z* value match the simulated spectrum for a **Cu_2O_2** species.

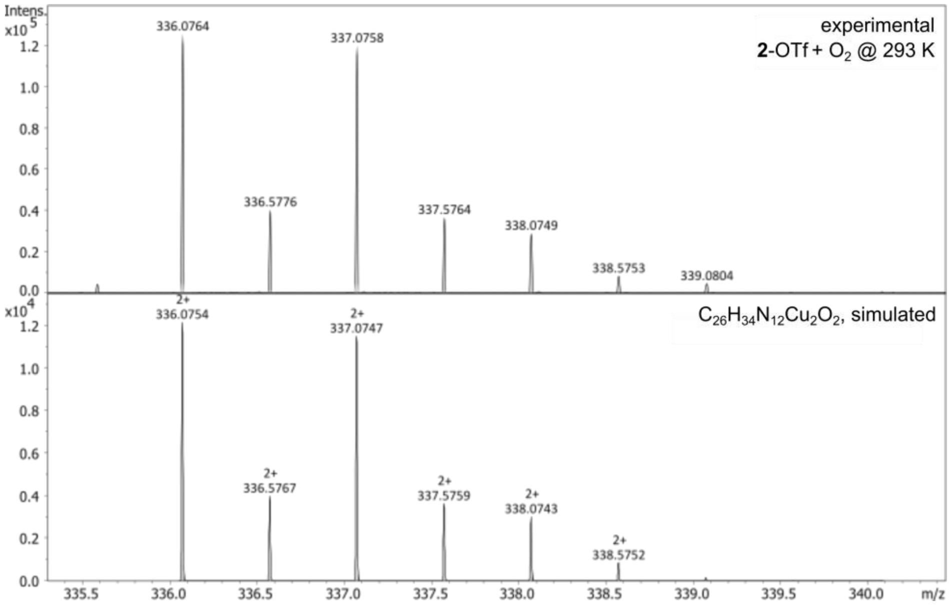

Figure S84: Cryo-UHR-ESI mass spectrum of **2**-OTf after reaction with O_2 at room temperature. The isotopic pattern and corresponding *m/z* value match the simulated spectrum for a **Cu_2O_2** species.

9.7. Ligand fragments as decomposition species upon reaction with O_2 and DMDO

Self-oxidized ligand fragments of **MO8** were observed in the UHR-ESI mass spectrum of **2**-PF_6 upon reaction with dioxygen at room temperature and at 308 K. Such self-oxidized ligand fragments or products from solvent oxidation are also reported for *μ*-1,1-hydroperoxo dicopper(II) complexes as decay products[28,29]. In contrast, KINDERMANN and MEYER observed and isolated a *μ*-hydroxo dicopper(II) complex as the only decay product of their **Cu_2OOH** species with the ligand **L^{et}**.[28] Here, we observed fragments of the ligand **MO8** after oxygenation with O_2 at room temperature or DMDO at 193 K (Figure S85 – S88). With an excess of DMDO or when added at room temperature, comparatively more of the fragments can be seen which shows the exceptional oxidizing strength of this oxygen-atom transfer reagent.

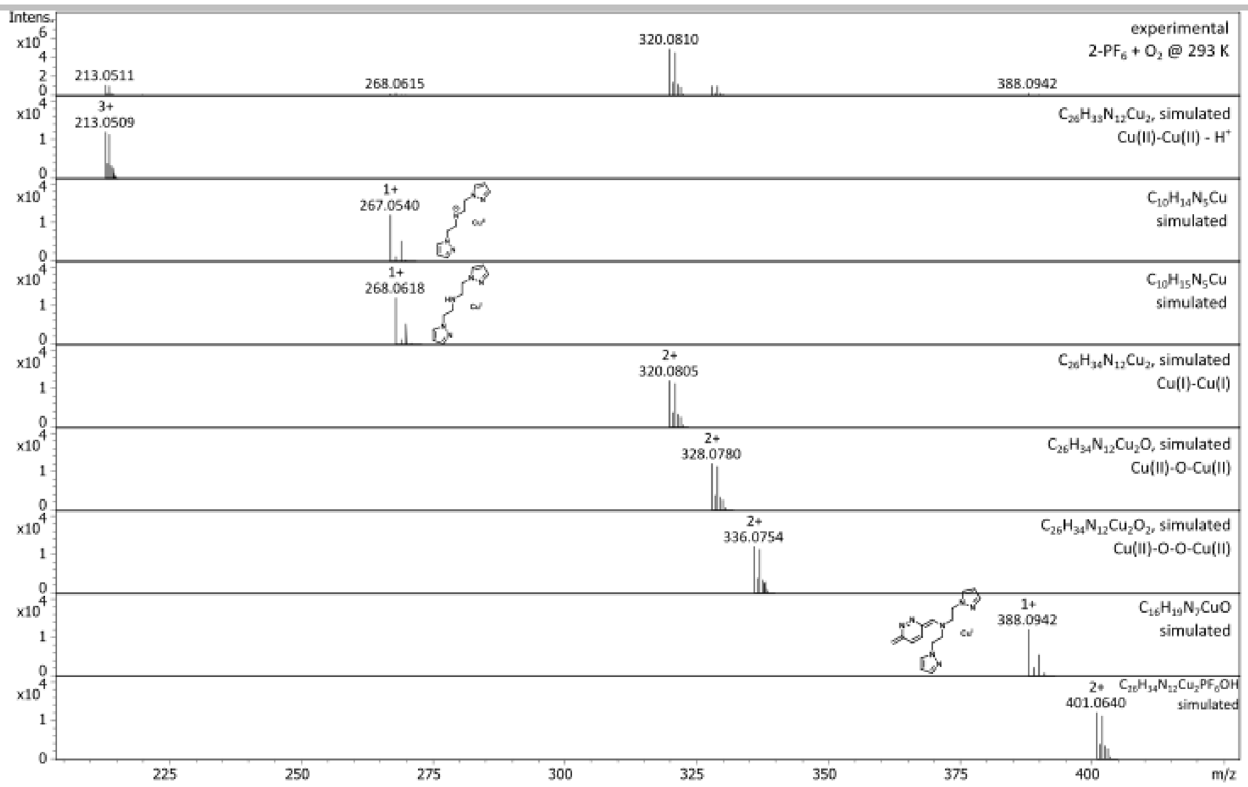

Figure S85: Cryo-UHR-ESI mass spectrum of **2**-PF_6 after 24 h reaction with $^{16}O_2$ at room temperature in acetone. The simulated spectra show different fragments of the ligand together with either copper(I) or copper(II). In addition, the dinuclear copper(I) complex containing the intact ligand **MO8** can be detected as well, representing the main species.

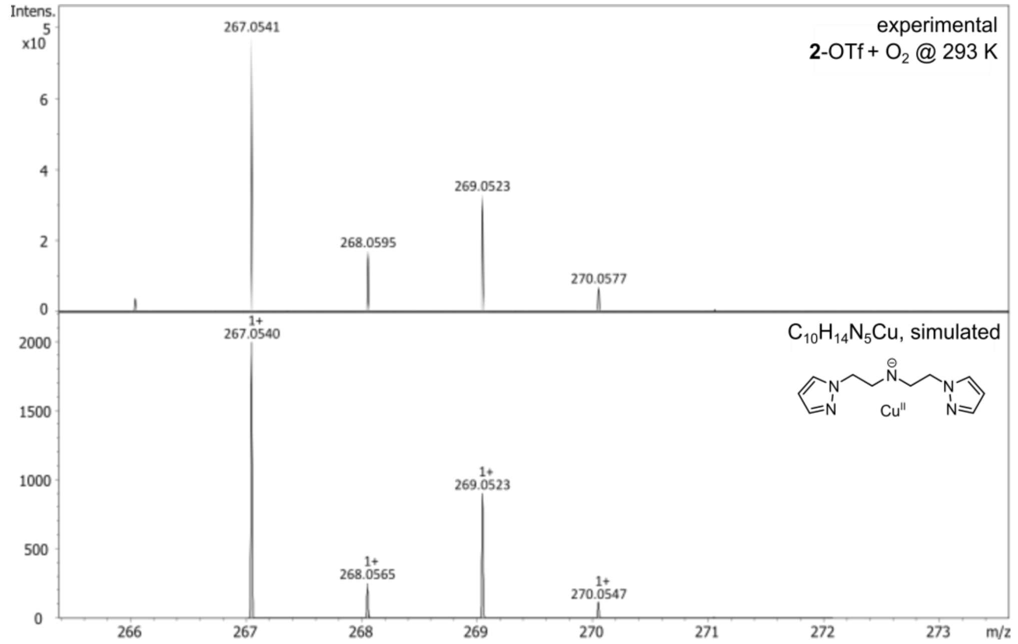

Figure S86: Cryo-UHR-ESI mass spectrum of **2**-OTf after reaction with $^{16}O_2$ at room temperature in acetone after 24 h. The cutout of the spectrum (top) matches the simulated spectrum for a ligand fragment and copper(II) (bottom).

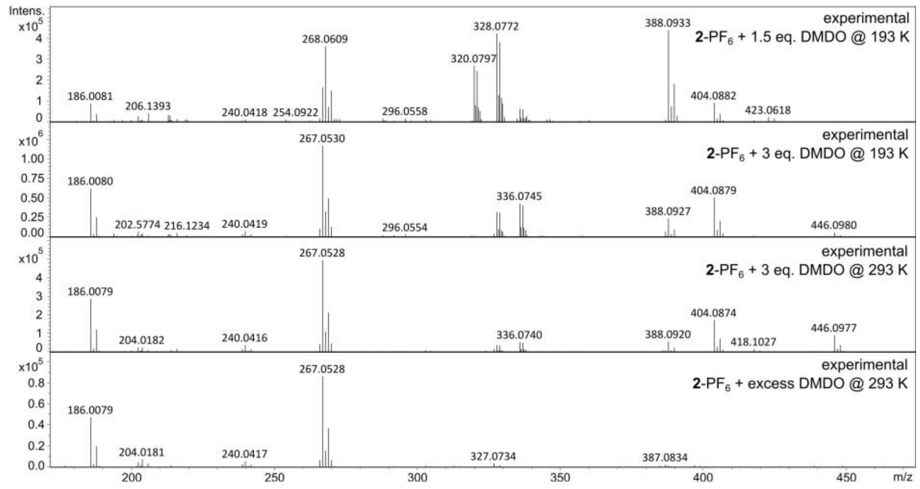

Figure S87: Cryo-UHR-ESI mass spectrum of **2**-PF_6 with different concentrations of DMDO at 193 K (and rt). The addition of a larger excess of DMDO or the addition at room temperature result in more decomposition products.

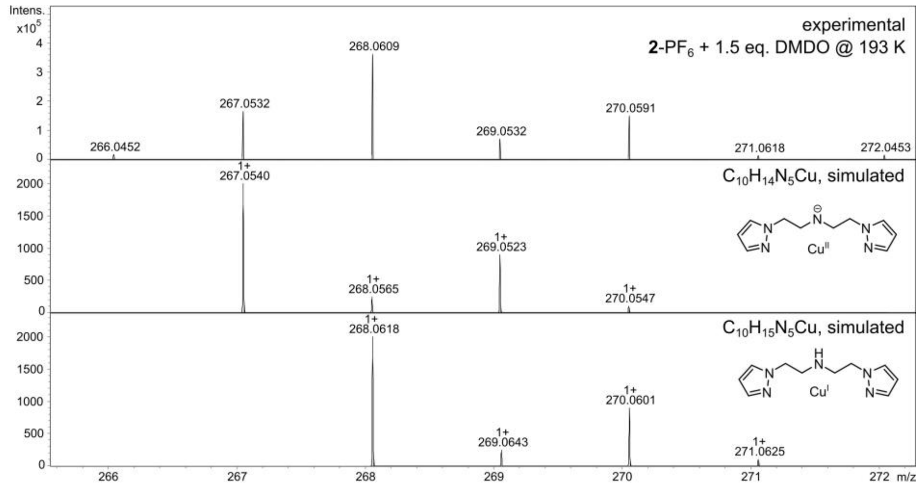

Figure S88: Cryo-UHR-ESI mass spectrum of **2**-PF_6 with DMDO at 193 K. The cutout of the spectrum (top) matches the simulated spectrum for a ligand fragment and copper (mid and bottom).

10. Electron Paramagnetic Resonance

10.1. EPR spectrum of 2-PF_6, 3-PF_6 and the green species

The X-band EPR spectra of the green species (Figure S89b) shows an EPR-active signal with g = 2.092 and thus provides evidence for the presence of Cu(II).[30] In contrast to the green species, the spectrum of the **Cu_2OH** complex **3** (Figure S89a, blue) does not show an EPR-active signal, which indicates an antiferromagnetic coupling of the Cu(II) centers.[31] However, it must be mentioned here that a weak signal is visible in the EPR spectrum of **3**-PF_6, which is already present in the EPR spectrum of the Cu(I) complex **2**-PF_6 (g = 2.045, Figure S89a, black). This signal indicates that traces of Cu(II) were already present in the Cu(I) complex.

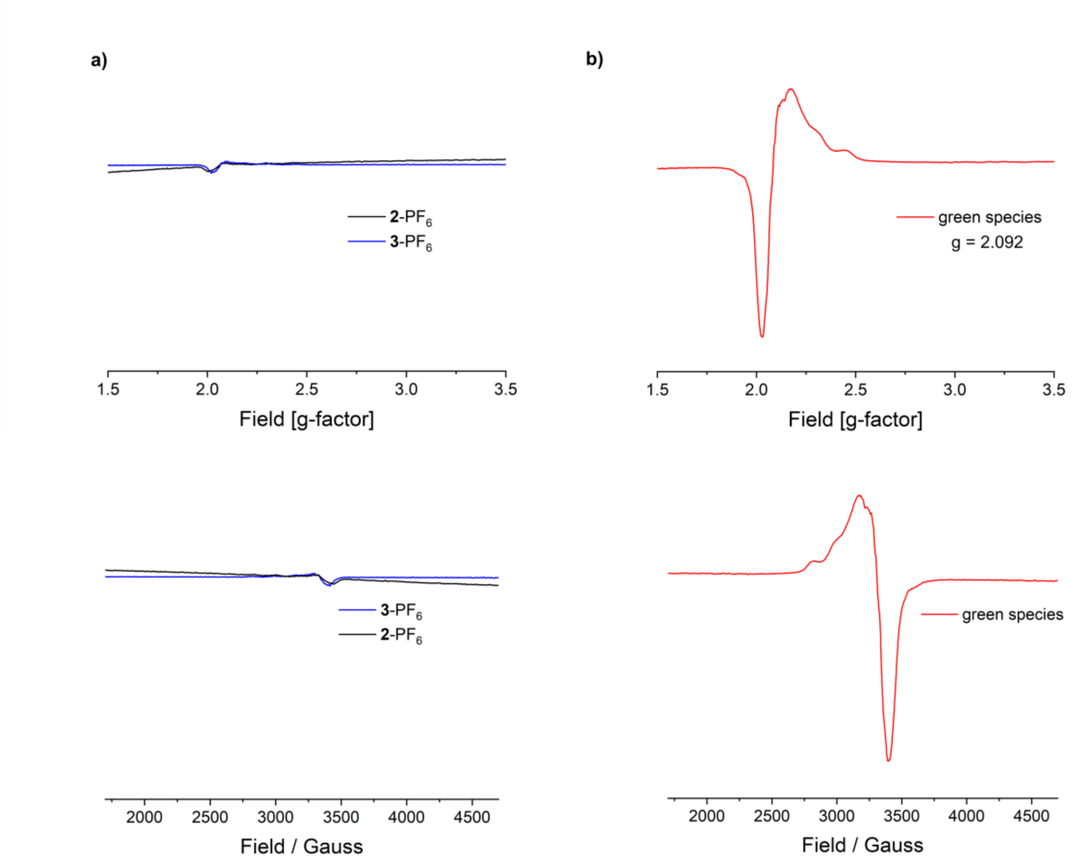

Figure S89: a) X-band EPR spectra of acetone solutions of **2**-PF_6 and **3**-PF_6 measured at 77 K. **b**) X-band EPR spectrum of the green species obtained after reaction of an acetone solution of **2**-PF_6 with O_2 at room temperature for 24 h (measured at 77 K). The spectrum of the green species exhibits an EPR signal with g = 2.092 which indicates the presence of Cu(II) species, whereas the **Cu_2OH** complex **3**-PF_6 seems to be EPR silent.

11. X-ray Absorption Spectroscopy

11.1.Precursor 2-PF_6

2-PF_6 has a XANES spectrum typical for Cu(I) complexes (Figures S90 and Figure S93) .[32] Maximum of the first derivative is at 8980.5 eV; 50 % of the edge jump occurs at 8983.9 eV. There is a shoulder at ~8981.5 eV with intensity ~0.35 and another weakly pronounced shoulder is at ~ 8985 eV. These transitions are assigned to the electric-dipole allowed transition from Cu 1s to Cu 4p orbitals.[33] This spectrum is very similar to the spectrum observed for a complex from the same ligand family with similar coordination.[34]

11.2.Reaction of 2-PF_6 with oxygen

In order to generate the **Cu_2OOH** species, the following treatment of the precursor solution was done. Initially, the solution of **2**-PF_6 was pre-frozen in liquid nitrogen, followed by thawing and subsequent oxidation by oxygen bubbling just above the melting temperature of the solution. Oxygen bubbling was limited to a few minutes to avoid excessive temperature elevation; the solution was frozen in liquid nitrogen again. This cyclic process was iterated, with various durations of oxygen bubbling (up to 15 minutes in total). Cryostat temperatures during the measurement were within the range of 183 K to 203 K. In one experiment, the solution was kept for 30 min at 195 K to complete the reaction. However, judging by the changes in the XANES region, only a small part of the precursor was converted into the oxidized species in all these conditions (Figure S90, orange and green curves).

For the generation of the green species, **2**-PF_6 was exposed to oxygen at RT followed by 24 hours of storage at ambient conditions. This treatment led to full oxidation of the complex to Cu(II): the edge shifted to higher energies by ~3.9 eV and a pre-edge emerged at ~8977.9 eV /Figure S90, red curve). Emergence for a pre-edge is characteristic for both Cu(II) and Cu(III). However, Cu(III) compounds have a pre-edge at ~2 eV higher energies (~8980 eV) than Cu(II) (~8978 eV)[35], which allows us to assign this as a Cu(II) species.

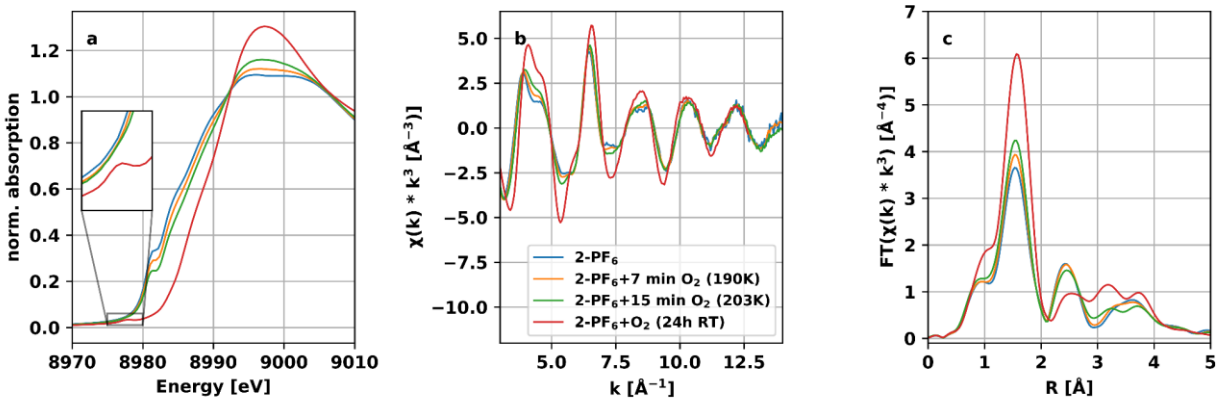

Figure S90: X-ray absorption spectra in XANES region (a), χ(k)*k^3 (b) and FT(χ(k)*k^3) (c) for precursor **2**-PF_6 and three oxidized species obtained by bubbling oxygen at different conditions. Blue: **2**-PF_6; orange: **2**-PF_6 oxidized for a total of 7 minutes (1-1.5 minutes of oxygen bubbling per cycle) and measured at 190 K; green: **2**-PF_6 oxidized for a total of 15 minutes (3 times 5 minutes). After waiting for 30 min at 195 K, the sample was measured at 203 K; red: **2**-PF_6 oxidized at RT and kept for 24 hours at ambient conditions.

To extract detailed structural information, EXAFS modeling for **2**-PF_6 and **2**-PF_6 + O_2 was done using EvAX software.[9] Within EvAX, ab initio real-space multiple-scattering FEFF8.50L code[36] was used to calculate theoretical phases and amplitudes of the scattering paths. Hydrogen atoms were excluded from the model structures. First, the first-shell fit was done with Larch[8] in order to check whether the model shows a good match in the first shell and determine the difference (dE_0) in energy scale between experiment and theoretical calculations and the scaling factor (S_0^2) between experiment and theoretical EXAFS amplitudes. These parameters were fixed during the following reverse Monte-Carlo (RMC) based fit with ExAX. Both single-scattering (SS) and multiple-scattering (MS) up to 8th order contributions were taken into account. Paths with amplitudes higher than 0.1% of the strongest path were included. Potential was calculated self-consistently for a sphere with radius of 4 Å around the absorbing atom.

Several RMC simulations with different starting pseudo-random number sequences were performed for each model. At each step of the RMC simulation, all atoms in the supercell were randomly displaced to account for thermal and/or static disorder. Maximum allowed displacement was set to be 0.4 Å. The difference between the Morlet wavelet transforms (WTs) of the experimental and simulated $\chi(k)*k^2$ spectra was used as a criterion for the optimization. The structural result of this fit is a set of atomic coordinates which were used to calculate a radial distribution function (RDF) around the absorbing atom (Cu). The resulting RDFs for each starting model were reproducible within the noise; finally, RDFs obtained in calculations with the same parameters for a certain model were averaged.

For **2**-PF_6, the crystallographic structure was used as a starting model to construct the RMC simulation box. The fitted RDF shows a good agreement with the model (Figure S95 and Figure S96). Fitting was done in wavelet(k,R)-space using R = 1 – 4.5 Å and k = 3 – 13.1 Å^{-1}; 10 calculations with 125 molecules each were done. FEFF calculations were done for paths with R < 5 Å.

Since the IR spectroscopic data of the green species give evidence to the presence of a hydroxo species, for the green species EXAFS spectra were fitted to several hydroxo models as well as the crystal structure of the **Cu_2OH** complex. Fitting was done in wavelet(k,R)-space using R = 1 – 4 Å and k = 2.8 - 12.6 Å^{-1}; for each model several calculations were carried out, each with 64 molecules. FEFF calculations were done for paths with R < 4.7 Å. During the fit, oxygen and nitrogen atoms of the first shell sometimes swapped places, since they are not distinguishable in EXAFS; thus, RDF(N+O) is plotted. Figure S91 shows the RDFs of the green species obtained in the fit with **$Cu_2(\mu$-OH)OH** and **Cu_2OH** (crystallographic) models, as well as corresponding EXAFS fits.

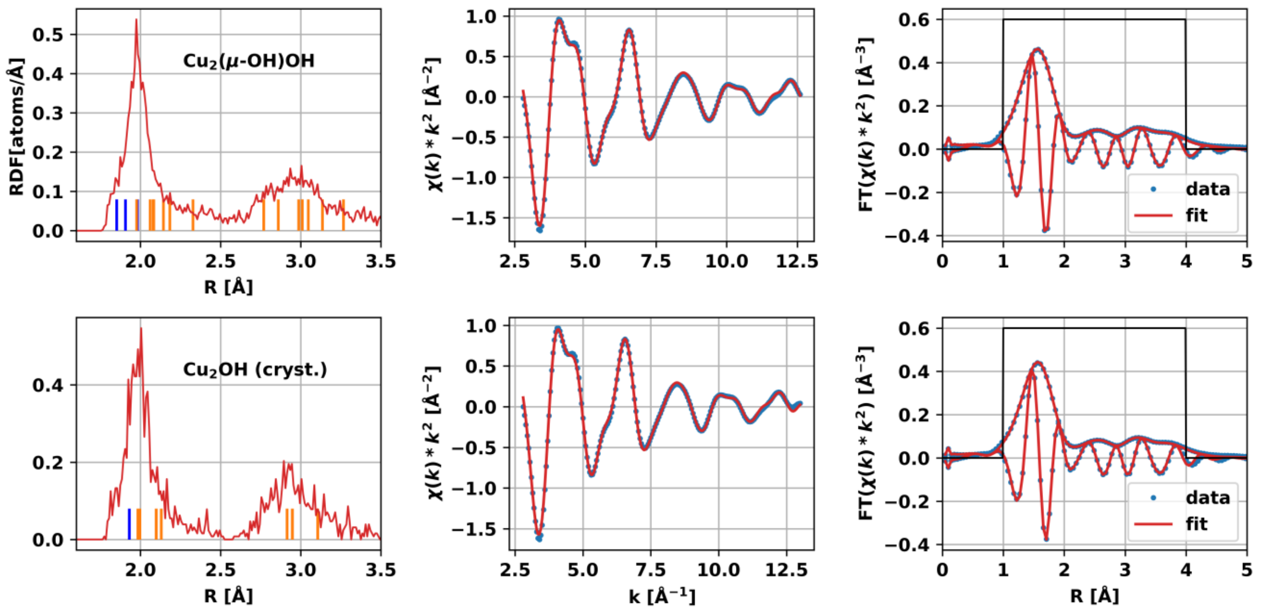

Figure S91: Left: Red lines show RDFs for N+O atoms obtained using RMC fits of the EXAFS spectra for dataset **2**-PF_6+O_2 with the **$Cu_2(\mu$-OH)OH** model (with pyrazoles in trans position) and **Cu_2OH** (crystallographic) model. Orange/blue vertical lines show distances from copper to nitrogen/oxygen atoms in the model. Middle/Right: Corresponding EXAFS fits in k- and R-spaces.

Based on our results we could make the following conclusions for the EXAFS fit of **2**-PF_6 + O_2 dataset:

1) Different starting models result in similar fitted RDFs: there are several N/O atoms in the first shell resulting in a peak around 2 Å, and there is also a tail at 2.3-2.5 Å
2) There is no model which fits the data perfectly: the shown **$Cu_2(\mu$-OH)OH** and **Cu_2OH** models should have more electron density of N/O around 2.1-2.2 Å than the RDFs obtained in the fit. At the same time the tail at 2.3-2.5 Å agrees with the observation from IR spectroscopy that **2**-PF_6 + O_2 could contain **$Cu_2(\mu$-OH)OH**, since it has a N atom in this range, unlike **Cu_2OH**.

11.3. Reaction of 2-PF_6 with DMDO

For the low-temperature experiment with DMDO, the cuvette with the **2**-PF_6 solution was cooled to 190 K in an ethanol cooling bath. Then a pre-cooled DMDO solution in acetone was added dropwise. Subsequently the cuvette was frozen in liquid nitrogen to prevent the solution from warming up.

The oxidation of **2**-PF_6 with DMDO was attempted twice. First, 1 eq. of DMDO was added. The color of the solution changed to green, but with a murky brownish tinge. The XANES spectrum (Figure S92) demonstrated that the compound was oxidized: the edge shifted to higher energy and a pre-edge feature started to emerge. However, the oxidation was probably not yet full, judging by the remaining shoulders resembling the precursor spectrum, which agreed with the observation of the color. In the second experiment, 1.5 eq. of DMDO was added resulting in a pure green solution. A stronger shift to the edge was observed and the pre-edge became more pronounced.

We assume that the content of DMDO was slightly smaller than nominal, resulting in less than full oxidation upon the addition of one equivalent of DMDO to **2**-PF_6. As a result, the addition of 1 eq. DMDO (ox1) resulted in a mix of the precursor and oxidized species. When we added 1.5 eq. of DMDO (ox2), we possibly could have created over-oxidized species, such as peroxo-species. In order to check for over-oxidizing, ox1 was modeled as a linear combination of **2**-PF_6 and ox2. The linear combination fit closely follows the spectrum, meaning that ox1 indeed contains a big contribution from ox2 (75%). All following analysis was done for **2**-PF_6 and ox2, further denoted as "**2**-PF_6 + DMDO". The XANES spectrum of **2**-PF_6 + DMDO (Figure S93, b) looks like a characteristic Cu(II) spectrum. The edge position shifts by ~3.6 eV relative to **2**-PF_6 and the pre-edge occurs at 8977.8 eV.

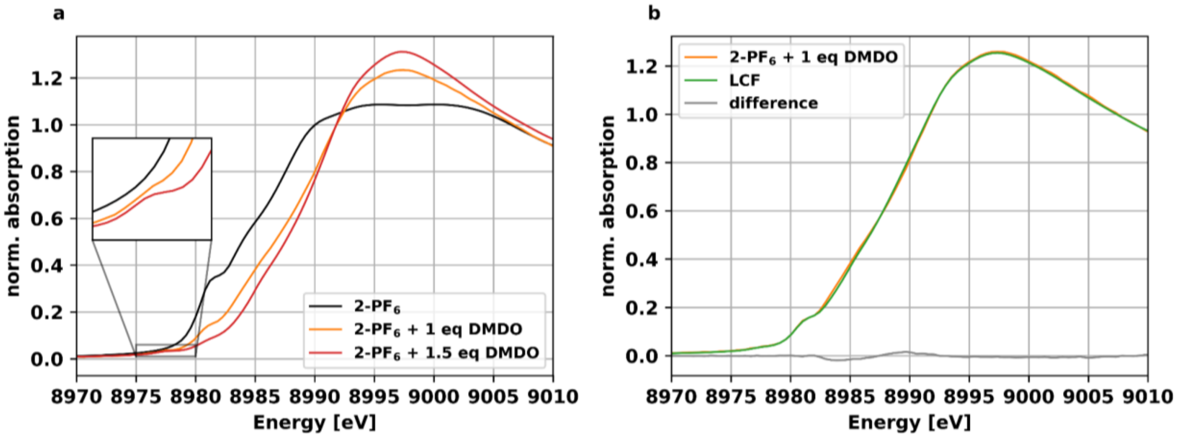

Figure S92: a) XANES spectra of **2**-PF_6 (black), **2**-PF_6 + 1 eq. DMDO (orange) and **2**-PF_6 + 1.5 eq. DMDO (red). b) XANES spectrum of **2**-PF_6 + 1 eq. DMDO (orange) and linear combination fit (LCF) of this spectrum using **2**-PF_6 and **2**-PF_6 + 1.5 eq. DMDO as standards (green).

EXAFS spectra of **2**-PF_6 and **2**-PF_6 + DMDO are shown in Figure S93. Upon oxidation of **2**-PF_6 with DMDO, there is a notable increase in the amplitude of χ(R). This confirms that an additional oxygen(s) is coordinated to Cu after the oxidation. There is no clear change of the first-shell distance.

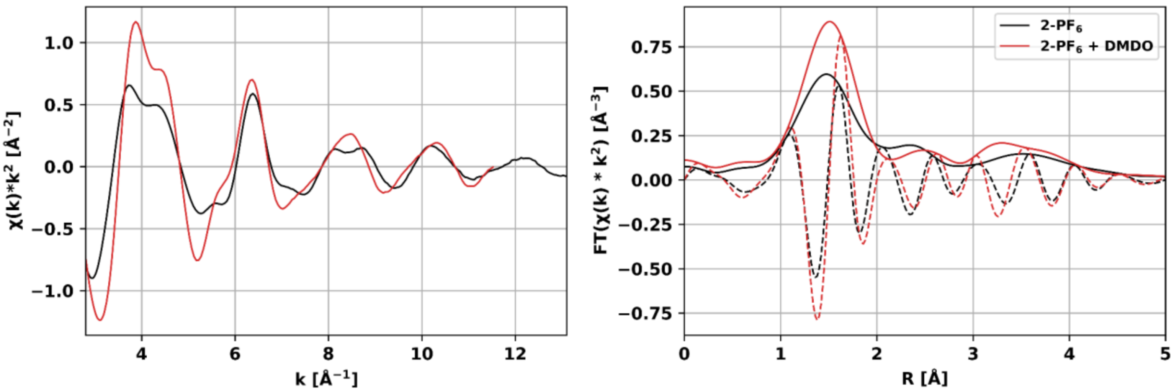

Figure S93: EXAFS spectra of **2**-PF_6 and **2**-PF_6+DMDO in k- and R-spaces.

A comparison between the spectra of **2**-PF_6 + O_2 (RT) and **2**-PF_6+DMDO is shown in Figure S94. **2**-PF_6 + O_2 has a stronger shift of the edge to higher energies at the lower part of the edge. Likely it means that with DMDO there is still some precursor left in the solution.

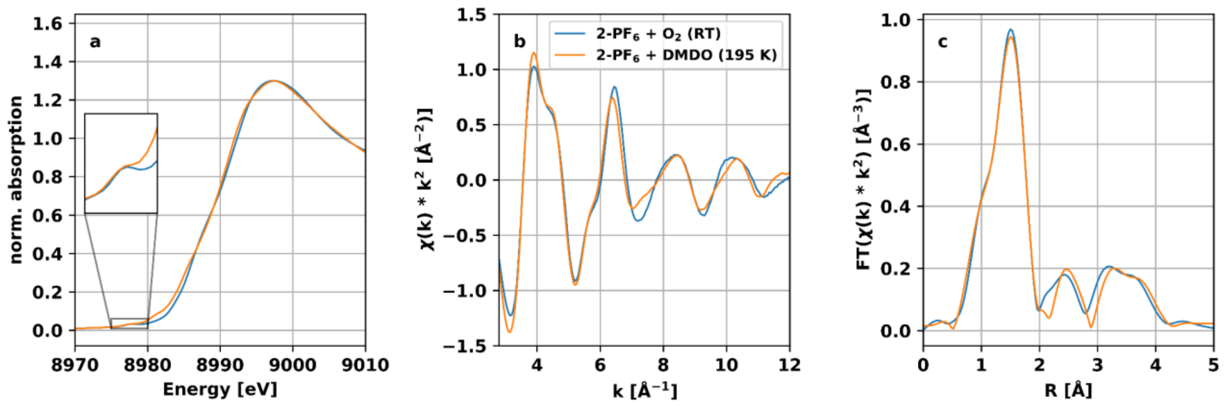

Figure S94: a) X-ray absorption spectra in XANES region (a), χ(k)*k^2 (b) and FT(χ(k)*k^2) (c) for **2**-PF_6 + O_2 (RT, 24 h) and **2**-PF_6 + DMDO (195 K).

For **2**-PF_6 + DMDO, several possible DFT-calculated models were used: **Cu_2O** (*μ*-oxo bridge between Cu atoms), **Cu_2O** + 1 acetone and 2 acetone molecules additionally coordinating to Cu, **Cu_2OH** (*μ*-hydroxo bridge between Cu atoms), **Cu_2OH** + 1 acetone as coligand; Cu(III) bis-*μ*-oxo (**Cu_2O_2**, two *μ*-oxo bridges between Cu atoms) and Cu(II) sideon-peroxo (**Cu_2O_2**, *μ*-*η*^{2}:η^2-peroxo bridge between Cu atoms). XANES spectra allowed us to identify **2**-PF_6 + DMDO as a Cu(II) complex, so Cu(III) models can be excluded, but we fitted the bis-*μ*-oxo model too to see how the resulting RDF will look with these starting models. Fitting results are shown in Figure S95 and Figure S96. Fitting was done in wavelet(k,R)-space using R = 1 - 3 Å and k = 2.8 - 11.4 Å^{-1}; 24 calculations were carried out, each with 64 molecules. Since it is the first shell that allows us to distinguish between the structural models, the fitting range was limited to R = 3 Å. FEFF calculations were done for paths with R < 3.5 Å. Additionally the **2**-PF_6 + DMDO EXAFS data were fitted with the different hydroxo models (**Cu_2OH** crystal structure, **Cu_2(*μ*-OH)OH** with pyrazoles in *trans* and in *cis* position, **$Cu_2(OH)_2$** open core, as well as with the **Cu_2(*μ*-OH)(OH)(OH)** structure, (Figure S97).

During the fit of **2**-PF_6 + DMDO, oxygen and nitrogen atoms of the first shell sometimes swapped places, since they are not distinguishable in EXAFS; thus, RDF(N+O) is plotted.

The approach of using EvAX for this kind of coordination complexes with large organic ligands (few absorbing atoms surrounded by many light atoms with no symmetry) was new: Typically, this software is used for solid-state materials. This software demonstrated that it can be used for this kind of materials too.

Based on our results we could make the following conclusions for the EXAFS fit of **2**-PF_6 + DMDO dataset:

3) Different starting models result in similar fitted RDFs: a large part of the electron density of N/O lies within 1.8 – 2.2 Å from Cu.
4) Many models have N/O atoms at 2.3 – 2.4 Å, however, fit results show that there is no electronic density at this distance. This allowed us to choose a Cu(II) model which is the best match for the fitted RDF: **Cu_2OH**, which does not have any N/O atoms at 2.3 – 2.4 Å.
5) Many fitted RDFs have an additional electronic density at 2.6 Å. It is possible that it is some fitting artifact or that there is indeed some amount of molecules, which have N/O at this position. However, this distance is too large to correspond to the coordination of acetone to Cu.
6) The sideon-peroxo (**Cu_2O_2**) model also shows a reasonable match with the fitted RDF, but we exclude this model since it is not realistic that with approximately 1:1 ratio of **2**-PF_6 to DMDO two oxygen atoms would be transferred to the oxidized complex, since one molecule of DMDO can only transfer one oxygen atom.
7) The RDF obtained with EvAX for **2**-PF_6 + DMDO often contains some “tails” or small sharp peaks on the sides of the first shell RDF, which are likely artifacts resulting from the limited informational content of the investigated system (light scattering atoms, no symmetry around Cu, range in k-space up to 11.4 Å^{-1}.

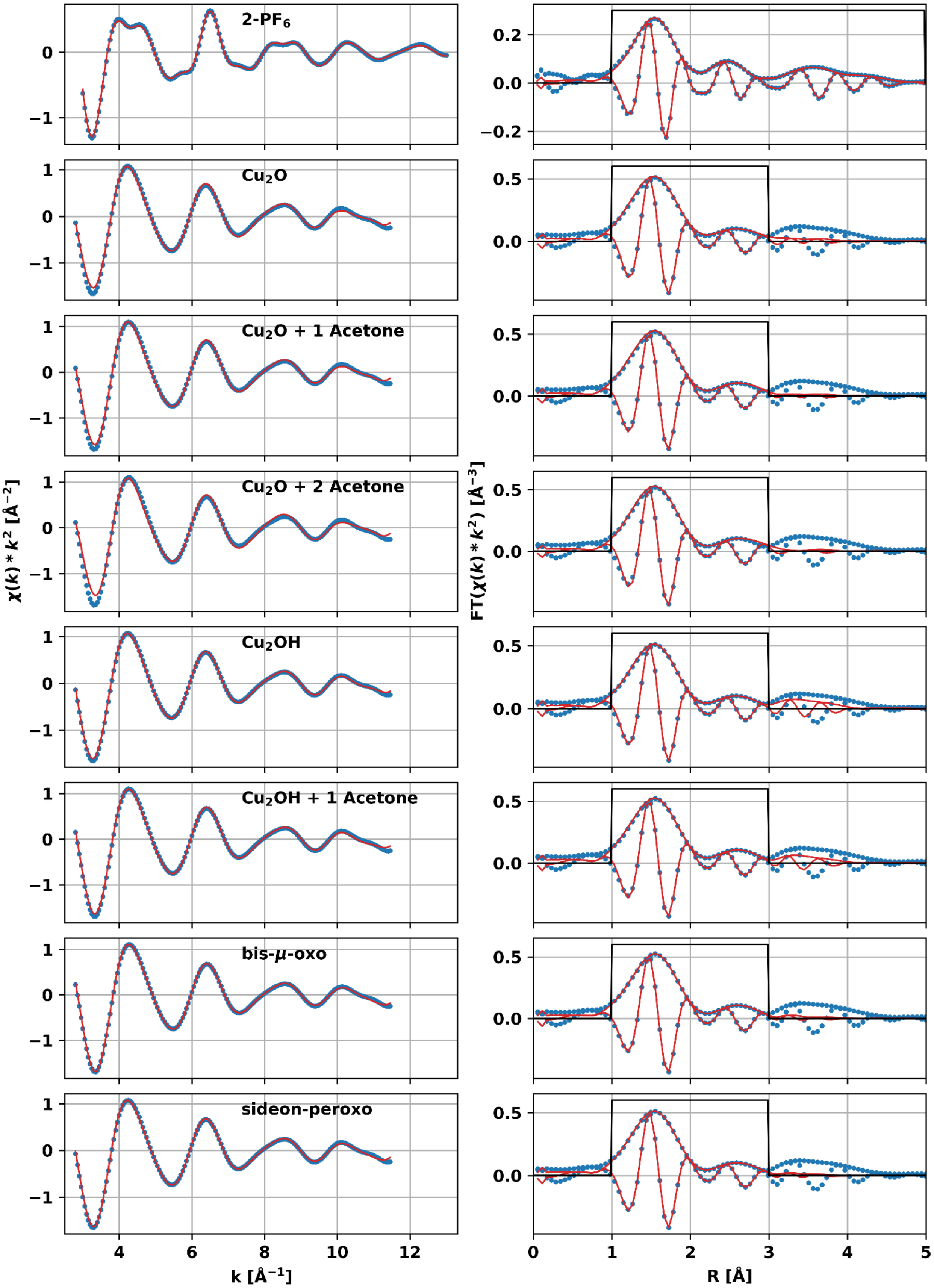

Figure S95: k- and R-space fits for **2**-PF_6 and **2**-PF_6 + DMDO. For **2**-PF_6 the crystallographic structure is used as a model. For **2**-PF_6 + DMDO, several DFT-calculated models were used. Blue dots: data, red line: fit; black rectangle: fitting window in R-space. Left column: χ(k), obtained by back Fourier transform of χ(R) in the chosen window (1 – 4.5 Å for **2**-PF_6; 1 – 3 Å for **2**-PF_6 + DMDO). Right column: real part and magnitude of χ(R). For **2**-PF_6 k = 3 – 13.1 $Å^{-1}$: for **2**-PF_6 + DMDO k = 2.8 – 11.4 $Å^{-1}$.

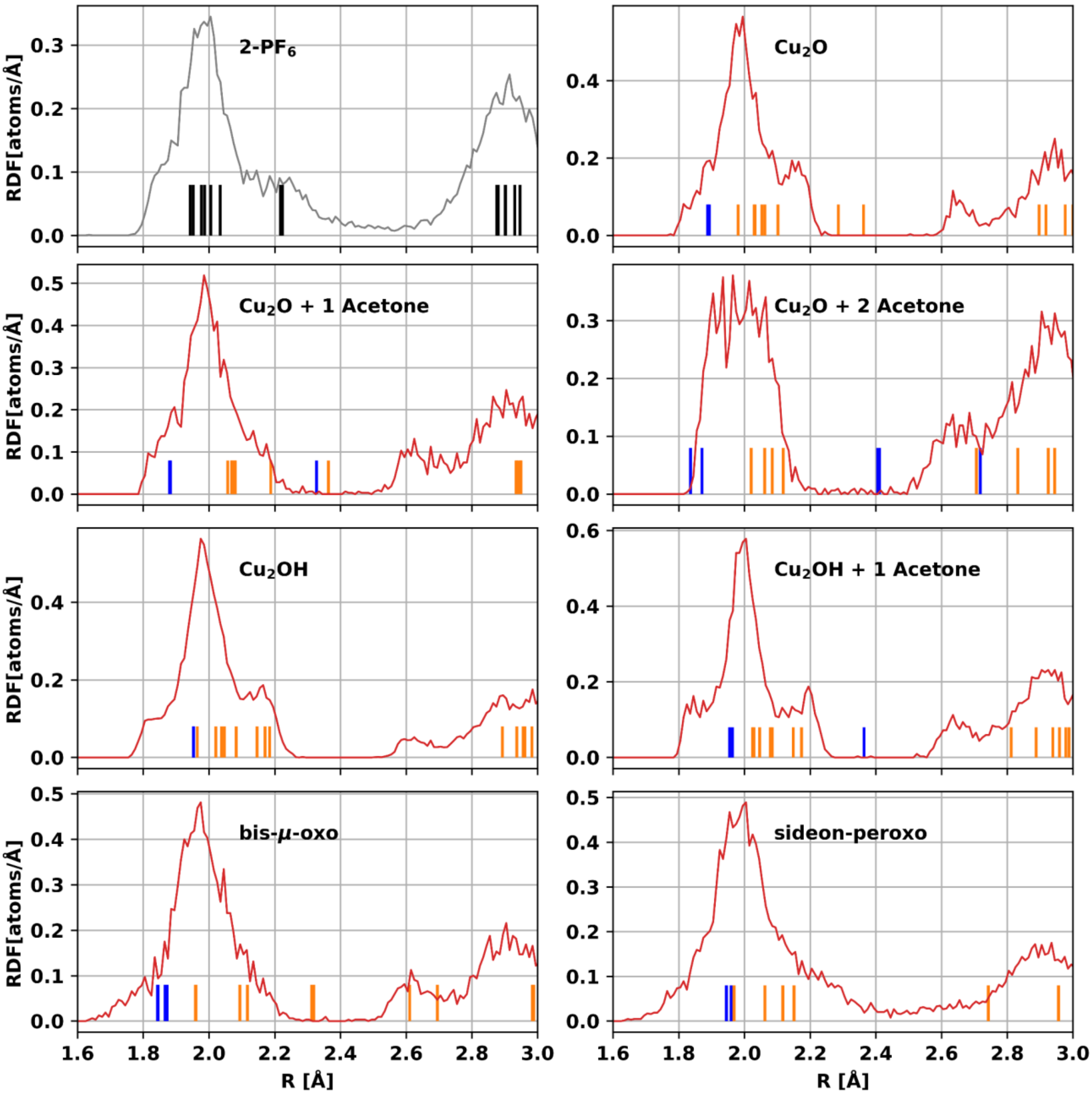

Figure S96: RDFs obtained using RMC fits of the Cu K-edge EXAFS spectra for **2**-PF_6 and **2**-PF_6 + DMDO. For **2**-PF_6 the crystallographic structure is used as a starting model. For **2**-PF_6 + DMDO, several DFT-calculated models were used. Grey: RDF for **2**-PF_6; black vertical lines: distances from copper to nitrogen atoms in **2**-PF_6 (crystallographic structure). Red: RDF for **2**-PF_6 + DMDO. Orange/blue vertical lines: distances from copper to nitrogen/oxygen atoms in various models for the oxidized species. EXAFS cannot distinguish between nitrogen and oxygen, so RDF is plotted as a sum of RDFs for nitrogen and oxygen. Different qualities of the resulting RDFs are due to different number of molecules placed in the simulation box during the RMC fit.

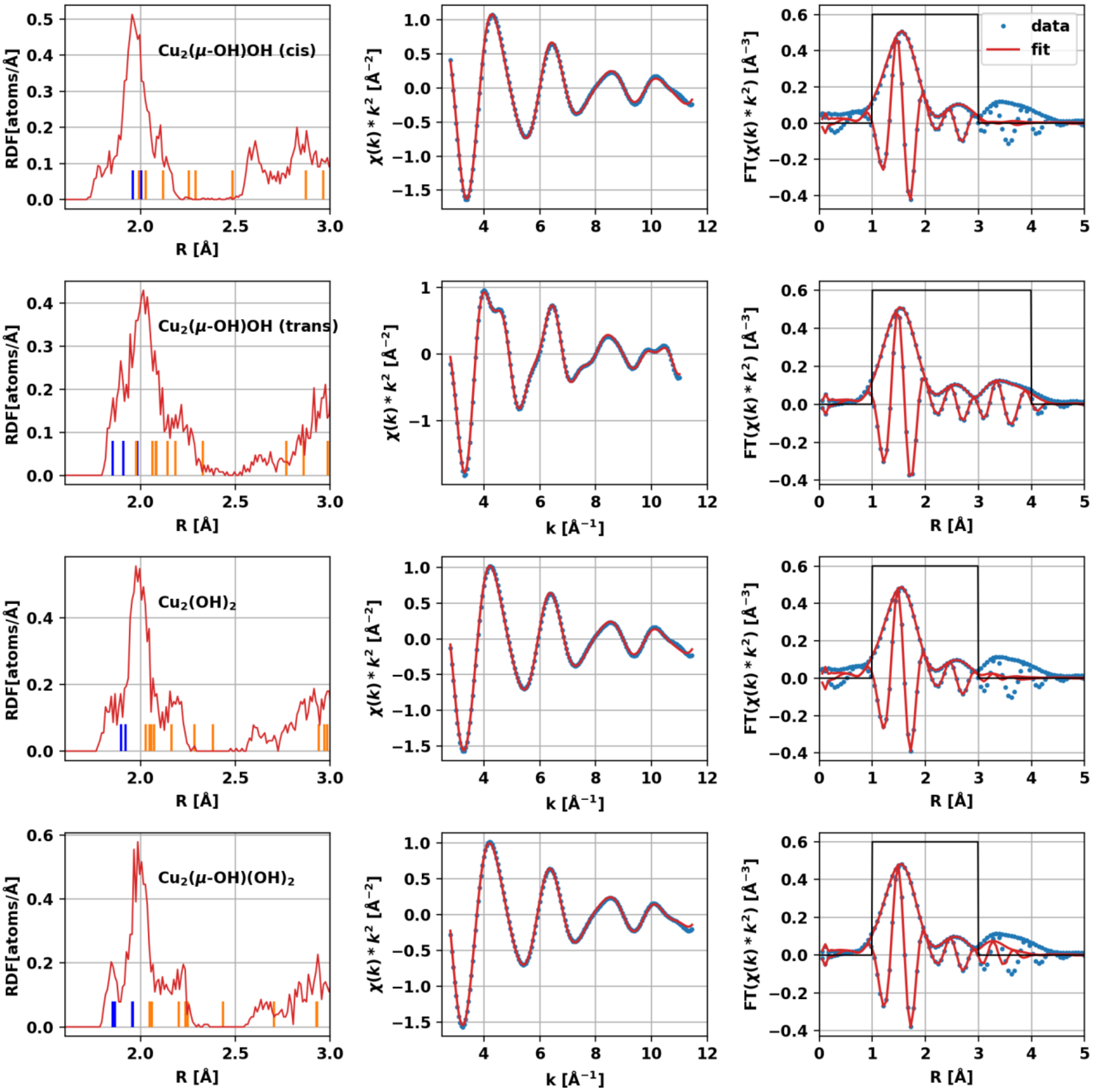

Figure S97: Left clolumn: RDFs (N+O) obtained using RMC fits of the Cu K-edge EXAFS spectra for **2**-PF_6 + DMDO using different hydroxo models. Middle, right: The corresponding fits in k- and R-space. From top to bottom: **Cu_2(*μ*-OH)OH** model with pyrazoles in *cis* position, **Cu_2(*μ*-OH)OH** model with pyrazoles in *trans* position, **$Cu_2(OH)_2$** open core, **Cu_2(*μ*-OH)$(OH)_2$**. Fitting radii R = 1-4 Å for **Cu_2(*μ*-OH)OH** (*trans*) and R = 1-3 Å for other models.

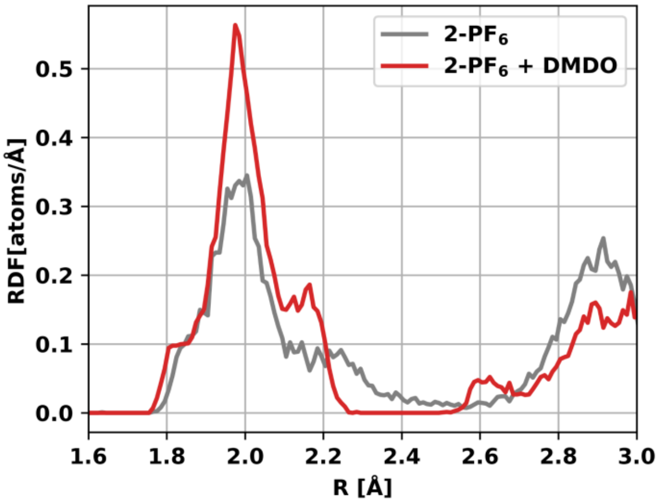

Figure S98: Fitted RDFs for **2**-PF_6 (grey, 1250 molecules) with crystallographic structure as model and **2**-PF_6 + DMDO (red, 1536 molecules) with DFT-calculated **Cu_2OH** species as model.

Since FEFFIT-based software such as Artemis is very common for fitting EXAFS data and was used in the previous study[34], we fitted the **2**-PF_6 + DMDO using FEFFIT-based Larch software in addition to EvAX. Fitting parameters are reported in (Table S9).

Table S9: Structural fitting parameters for the fit of **2**-PF_6 + DMDO with Larch using a **Cu_2OH** model.

Scattering path	R(DFT) [Å]	ΔR(fit - DFT) [Å]	σ^2 [Å]
O, $N_{pyrazole}$, $N_{pyridazine}$ (single scattering)	1.95 < R < 2.08	-0.03 ± 0.01	0.0040 ± 0.0008
N_{amine} (single scattering)	R = 2.18	-0.01 ± 0.04	
C, N (single scattering)	2.9 < R < 3.18	0.03 ± 0.05	0.018 ± 0.013
Multiple scattering (C,N)	3.15 < R < 3.5	0.08 ± 0.10	0.013 ± 0.037
Cu (single scattering)	R = 3.49	0 (fixed)	0.021 ± 0.067

Amplitude S_0^2 was fixed to be 1; E_0 was found to be 8981.0 ± 1.8 eV. Number of independent points in the fit was 12; the number of fitting parameters was 9. Paths up to R = 3.5 Å (35 paths) were included in order to do a fit up to R = 3 Å.
In general, Larch confirms that **Cu_2OH** model can be used to fit EXAFS data, but it is quite difficult to parametrize scattering paths in a reasonable way for a molecule with no symmetry and bulky ligands consisting of atoms, which look equal in EXAFS such as O, N, C. Cu-Cu distance was fixed, because otherwise other fitting parameters stopped being physically reasonable. "Debye-Waller factors" (σ^2) are quite large, especially for the single-scattering paths to C, N at 2.9 – 3.2 Å. Contribution of Cu-Cu scattering is also rendered quite small by a large σ^2. However, distance to Cu is 3.49 Å, so to make conclusions about the Cu-Cu distance, it would make sense to do a fit in a larger range than 1 – 3 Å. EvAX fit in 1 – 3 Å range was not sensitive to Cu-Cu distance.

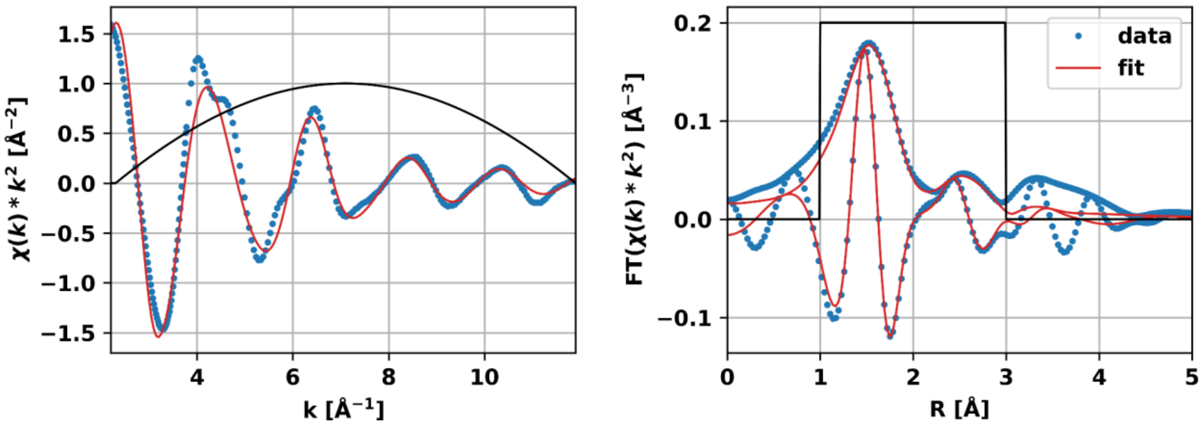

Figure S99: Fit of EXAFS of **2**-PF_6 + DMDO with Larch using **Cu_2OH** as a structural model. Left: k-space; fitting window was 2.8 – 11.4 $Å^{-1}$. Right: R-space; fitting window was 1 – 3 Å.

11.4. Cu_2OH complex 3-OTf and reaction with DBU and [Lut-H][OTf]

The XANES spectrum of the **Cu_2OH** complex **3**-OTf looks as expected for a Cu(II)-complex and **2**-OTf looks like a Cu(I)-complex and is very similar to **2**-PF_6. (Figure S100).

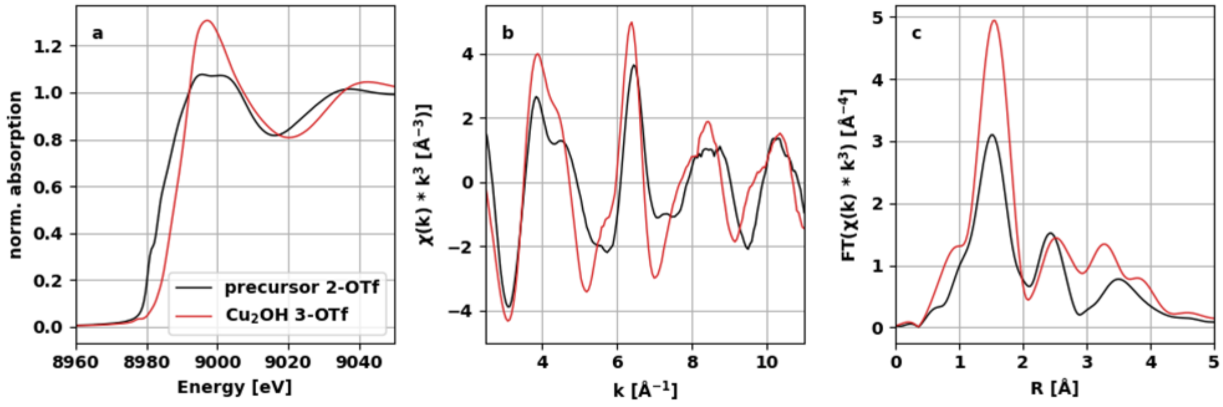

Figure S100: X-ray absorption spectra in XANES region (a), χ(k)*k^3 (b) and FT(χ(k)*k^3) (c) for the Cu(I)-complex **2**-OTf and the **Cu_2OH** complex **3**-OTf.

The EXAFS can be fitted well with crystallographic model and the fitted RDF matches the Cu-N/O bond lengths (Figure S101). Fitting was done with EvAX software in wavelet(k,R)-space using R = 1 - 4 Å and k = 2.8 - 11 $Å^{-1}$; 8 calculations were carried out, each with 64 molecules. FEFF calculations were done for paths with R < 4.5 Å.

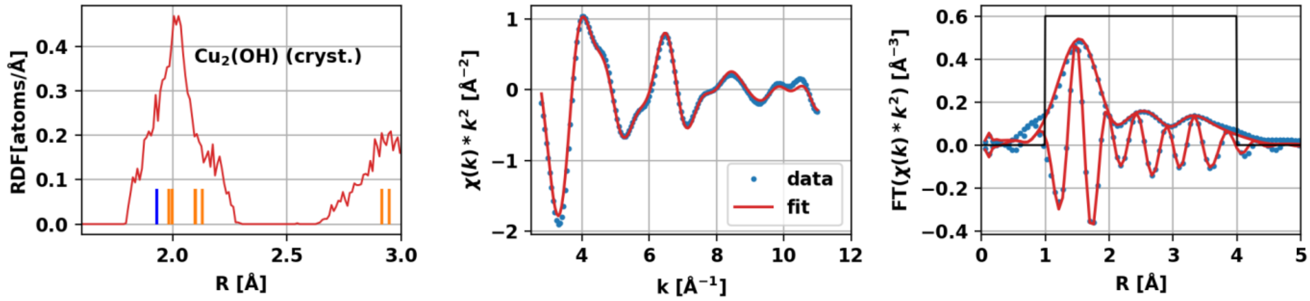

Figure S101: Left: RDF obtained in RMC fit of **3**-OTf solution using **Cu_2OH** crystallographic model. Middle/Right: Fit results in k- and R-spaces. Black line shows the fitting window in R-space.

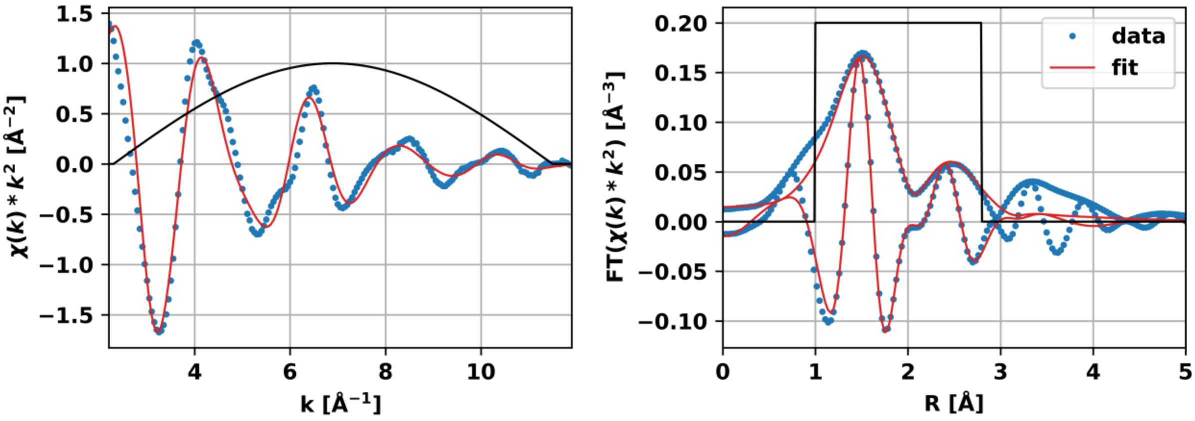

Figure S102: Fit of EXAFS of **3**-OTf with Larch using **Cu_2OH (cryst.)** as a structural model. Left: k-space; fitting window (black line) was 2.8 – 11 $Å^{-1}$. Right: R-space; fitting window (black line) was 1 – 2.8 Å.

As before, we fitted the **3**-OTf using FEFFIT-based Larch software in addition to EvAX. Crystallographic structure of **Cu_2OH** was used as a starting model. Fitting parameters are reported in (Table S10). Fit results are shown on Figure S102. The amplitude S_0^2 was fixed to be 1; E_0 was found to be 8980.3 ± 1.8 eV. The number of independent points in the fit was 10.4; the number of fitting parameters was 7. Paths up to R = 3.3 Å (25 paths) were included in order to do a fit up to R = 2.8 Å. Note the difference between the graphical representation of Larch and EvAX fit results in k-space: while EvAX reports χ(k) which is a back Fourier Transform of χ(R) in the R fitting window (for example, R = 1-4 Å), for Larch the fit is plotted vs the original χ(k) data, before the Fourier- and back Fourier transform. As a result, on the figure showing the fit with Larch, χ(k) data contains high frequencies which are not included in the fit.

Table S10: Structural fitting parameters for the fit of **3**-OTf with Larch using the **Cu_2OH** crystallographic model.

Scattering path	R(cryst.) [Å]	ΔR(fit – cryst.) [Å]	σ^2 [Å]
O, $N_{pyrazole}$, $N_{pyridazine}$, N_{amine} (single scattering)	1.95 < R < 2.18	-0.014 ± 0.014	0.0048 ± 0.0008
C, N (single scattering)	2.9 < R < 3.18	-0.01 ± 0.03	0.008 ± 0.005
Multiple scattering (C,N)	3.08 < R < 3.32	0.09 ± 0.09	0.01 ± 0.03

The resulting spectrum of **3**-OTf after reaction with DBU indicated that Cu(I) has formed. The LCF of **3**-OTf + DBU reveals that the spectrum is built of 64% of the precursor **2** and 36% of the **Cu_2OH** complex **3** (Figure S103).

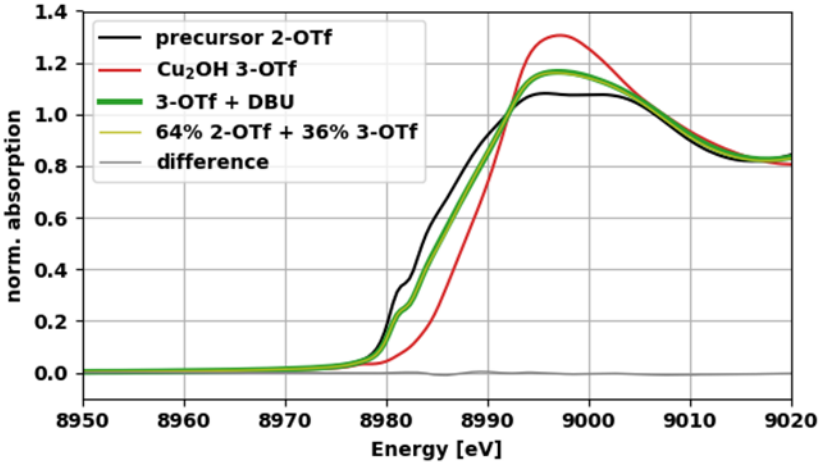

Figure S103: Comparison of the XANES spectra of precursor **2**-OTf (black), **Cu_2OH** complex **3**-OTf (red), solution obtained after deprotonation of 3-OTf with DBU (green) and LCF of **3**-OTf + DBU using **2**-OTf and **3**-OTf as standards (yellow). The grey line shows the difference between the LCF and the modeled spectrum.

After the addition of [Lut-H][OTf] the **Cu_2OH** complex **3** could only be partially restored. The LCF shows that the spectrum contains 35% of the precursor **2** and 65% of the **Cu_2OH** complex **3** (Figure S104).

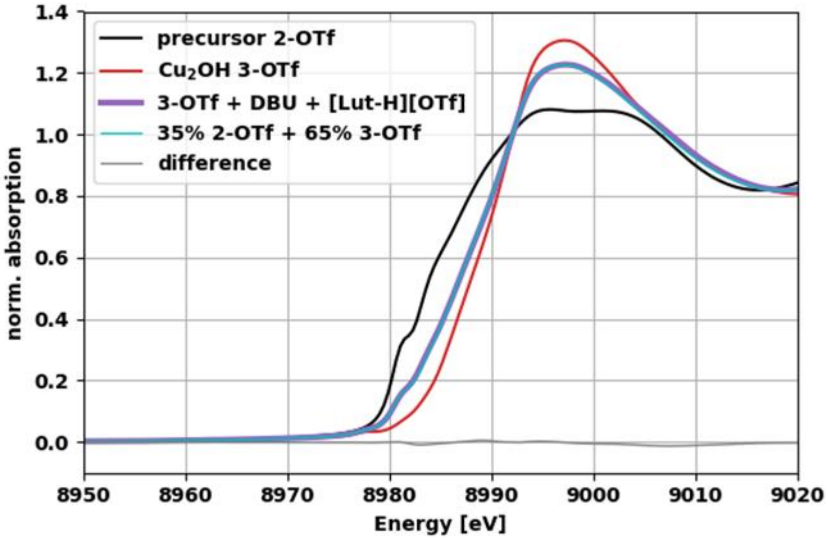

Figure S104: Comparison of the XANES spectra of precursor **2**-OTf (black), **Cu_2OH** complex **3**-OTf (red), solution obtained after reaction of **3**-OTf with DBU and [Lut-H][OTf] (violet) and LCF of **3**-OTf + DBU using **2**-OTf and **3**-OTf as standards (cyan). The grey line shows the difference between the LCF and the modeled spectrum.

Comparison of the RMC-fitted RDFs of **2**-PF_6+O_2, **2**-PF_6+DMDO and **Cu_2OH** is shown on Figure S105. For both models used there, there is the same observation: for **2**-PF_6+DMDO and **Cu_2OH** RDFs are very similar, while **2**-PF_6+O_2 has less electron density at 2.2 Å and a tail going towards 2.5 Å, confirming that the results of oxidation were different between these cases.

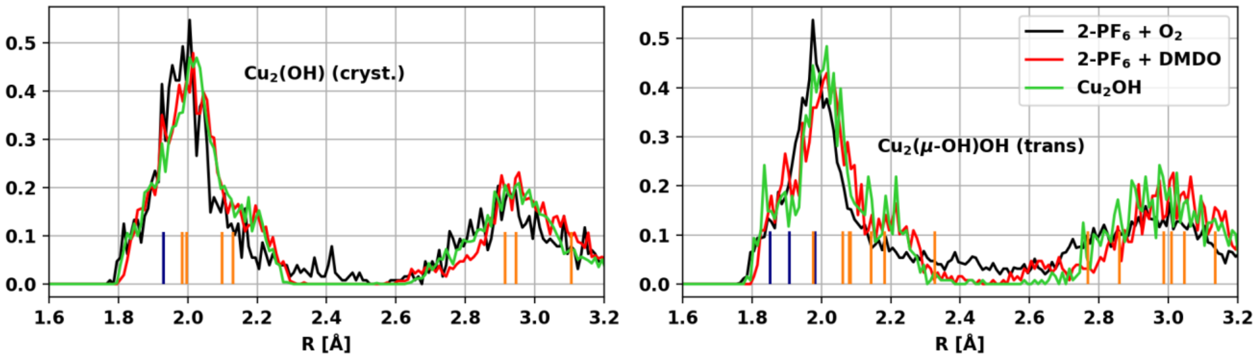

Figure S105: Comparison of RMC-fitted RDF (R = 1-4 Å) for datasets **2**-PF_6 + O_2 (black), **2**-PF_6 + DMDO (red) and **Cu_2OH** (green). Left: The starting model is the crystallographic structure of **Cu_2OH**; right: The starting model is DFT-calculated **Cu_2(μ-OH)OH** model with pyrazoles in *trans* position. Orange/blue vertical lines: distances from copper to nitrogen/oxygen atoms in the models.

12. Additional DFT Calculations

12.1. Cu_2O, Cu_2OH and Cu_2OOH: Geometry optimization, selected bond lengths and angles

To gain deeper insights into our **MO8** system, we performed calculations for the **Cu_2O**, **Cu_2OH** and **Cu_2OOH** complexes of **2** (Figure S106, Figure S107 and Figure S108). We decided to do calculations for solvent-free complexes and structures containing additional coligands, e.g. acetonitrile or acetone and compared these with the experimentally obtained data. In addition, the theoretically and experimentally obtained data show reasonable agreement given the known limitations of DFT.

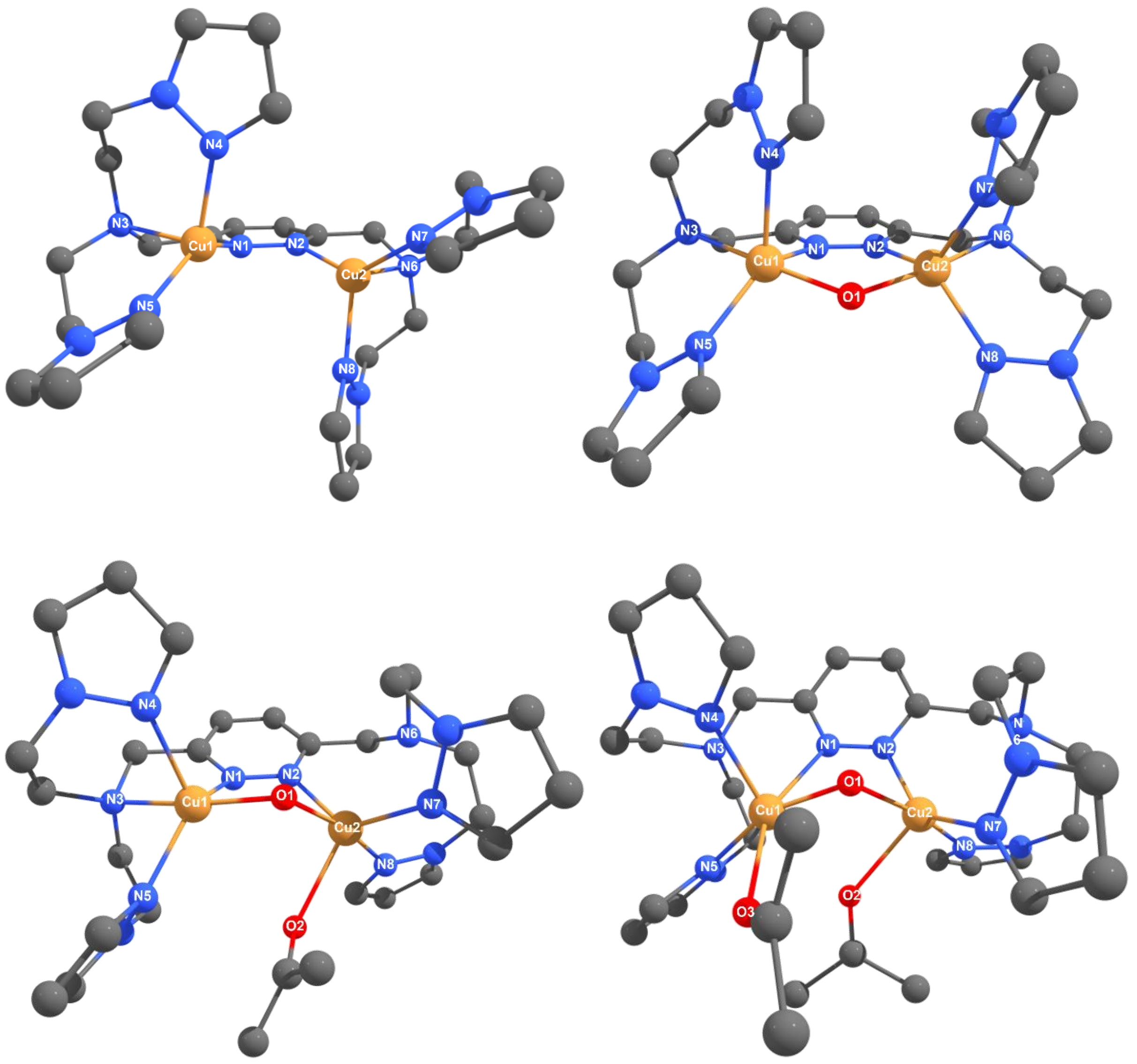

Figure S106: Geometry optimization of the **Cu_2** (top, left), **Cu_2O** (top, right) without and with one (bottom, left) and two (bottom, right) additional acetone molecules as coligands. The most promising DFT-structures are shown. All hydrogen atoms have been omitted for clarity. Selected bond lengths and angles are given in Table S11 (see below). DFT: RI-PBE-D3(BJ)/def2-SVP.

The distorted geometry of the **Cu_2O**, **Cu_2OH** and **Cu_2OOH** complexes with two additional acetone coligands indicate that very likely no or only one additional solvent ligand would coordinate. With the **Cu_2OH** and **Cu_2OOH** complex of Cu_2(**MO8**) the calculations showed that the second acetone molecule is bound only *via* hydrogen bonds. The crystal structure of the **Cu_2OH** complex **3**-PF_6 revealed that in fact no additional coligands coordinate to copper.

Table S11: Selected bond lengths (Å), including Cu···Cu distances (Å) and angles (°) for the DFT calculated **Cu_2O** species of the Cu_2(**MO8**) model system. DFT: RI-PBE-D3(BJ)/def2-SVP. Note: The numbering of the atoms is based on the DFT-optimized structures from Figure S106. The values in blue indicate that these are no longer bonds but distances between the corresponding atoms.

Cu_2(**MO8**) system	**Cu_2O**	**Cu_2O with one $CO(CH_3)_2$**	**Cu_2O with two $CO(CH_3)_2$**	Cu_2(**MO8**) system	**Cu_2O**	**Cu_2O with one $CO(CH_3)_2$**	**Cu_2O with two $CO(CH_3)_2$**
**Bond lengths / Å**				**Bond angles / °**			
Cu1···Cu2	3.241	3.191	3.133	Cu1-O1-Cu2	117.970	116.005	115.413
Cu1-N1	2.053	2.069	2.706	N1-Cu1-N3	78.735	75.573	-
Cu1-N3	2.286	2.364	3.286	N1-Cu1-N4	97.601	102.545	85.322
Cu1-N4	2.102	2.080	2.085	N1-Cu1-N5	138.963	135.755	111.632
Cu1-N5	1.981	2.070	2.020	N1-Cu1-O1	90.922	87.726	70.712
Cu2-N2	2.031	2.188	2.118	N4-Cu1-O1	93.915	99.183	93.780
Cu2-N6	2.362	2.942	2.832	N4-Cu1-N3	90.377	84.645	-
Cu2-N7	2.062	2.057	2.061	N4-Cu1-N5	123.067	114.831	107.630
Cu2-N8	2.029	2.078	2.061	N2-Cu2-N6	77.822	-	-
Cu1-O1	1.888	1.880	1.836	N2-Cu2-N7	125.642	132.675	139.252
Cu2-O1	1.894	1.883	1.871	N2-Cu2-N8	131.976	92.402	93.306
Cu1-O3	-	-	2.406	N2-Cu2-O1	91.809	83.628	82.109
Cu2-O2	-	2.327	2.412	N7-Cu2-O1	98.038	87.451	85.412
				N7-Cu2-N6	88.317	-	-
				N7-Cu2-N8	100.124	103.160	105.456
				N7-Cu2-O2	-	121.722	115.490
				N2-Cu2-O2	-	104.456	101.989
				O1-Cu2-O2	-	88.619	85.373
				N4-Cu1-O3	-	-	110.118
				N1-Cu1-O3	-	-	155.869
				O1-Cu1-O3	-	-	89.327

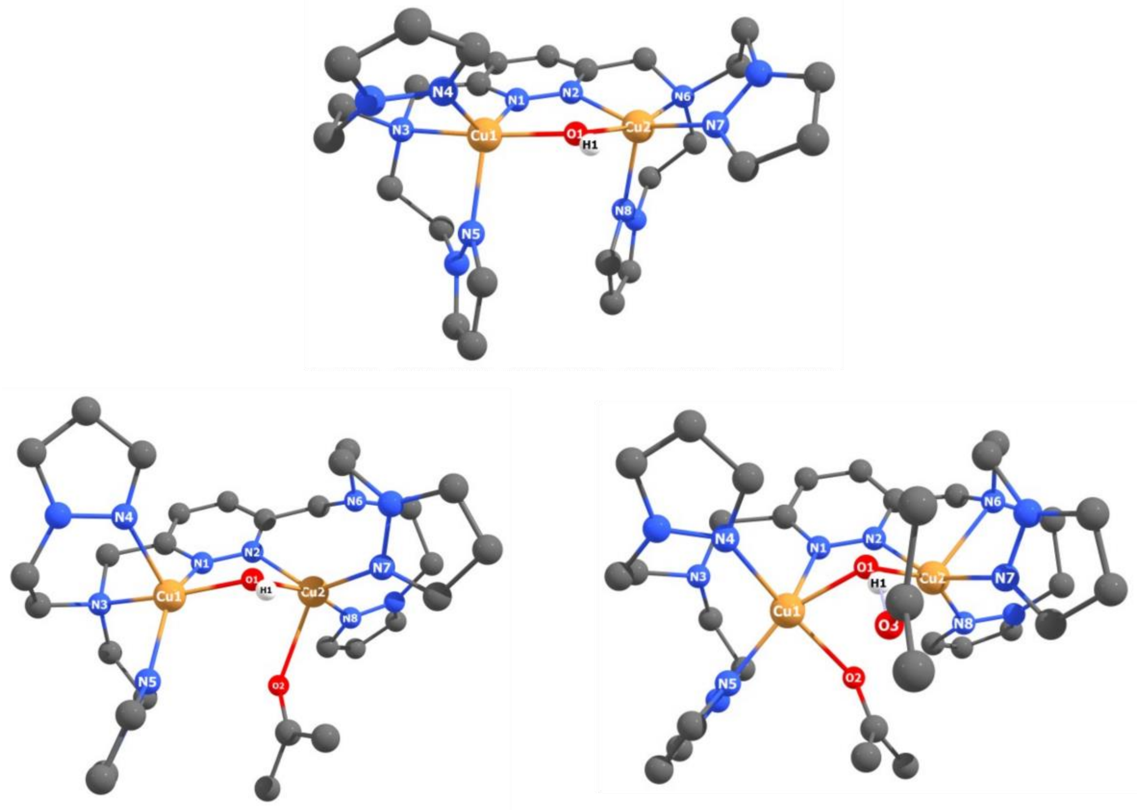

Figure S107: Geometry optimization of the **Cu_2OH** complex **3** without acetone (top), with one acetone coligand (bottom, left) and with two acetone coligands (bottom, right). All hydrogen atoms attached to carbon atoms have been omitted for clarity. Selected bond lengths and angles are given in Table S12 (see below). DFT: RI-PBE-D3(BJ)/def2-SVP.

Table S12: Selected bond lengths (Å), including Cu···Cu distances (Å) and angles (°) for the **Cu_2OH** species of the Cu_2(**MO8**) model system obtained by DFT.

Cu_2(**MO8**) system	**Cu_2OH**	**Cu_2OH with one $CO(CH_3)_2$**	**Cu_2OH with two $CO(CH_3)_2$**	Cu_2(**MO8**) system	**Cu_2OH**	**Cu_2OH with one $CO(CH_3)_2$**	**Cu_2OH with two $CO(CH_3)_2$**
**Bond lengths / Å**				**Bond angles / °**			
Cu1···Cu2	3.460	3.393	3.282	Cu1-O1-Cu2	124.722	120.012	113.837
Cu1-N1	2.047	2.078	2.462	N1-Cu1-N3	81.060	51.997	-
Cu1-N3	2.184	2.173	3.091	N1-Cu1-N4	132.297	100.223	92.859
Cu1-N4	2.021	2.085	2.048	N1-Cu1-N5	125.169	139.041	122.452
Cu1-N5	2.081	2.029	2.020	N1-Cu1-O1	83.838	82.806	76.242
Cu2-N2	2.038	2.149	2.041	N4-Cu1-O1	96.172	97.655	91.350
Cu2-N6	2.179	2.812	2.427	N4-Cu1-N3	95.575	87.844	-
Cu2-N7	1.964	2.046	2.015	N4-Cu1-N5	102.381	118.375	102.631
Cu2-N8	2.146	2.024	2.088	Cu1-O1-H1	117.223	117.620	107.593
Cu1-O1	1.953	1.955	1.953	N2-Cu2-N6	81.944	-	79.378
Cu2-O1	1.953	1.964	1.964	N2-Cu2-N7	155.176	136.500	152.562
Cu1-O2	-	-	2.214	N2-Cu2-N8	93.355	94.138	91.465
Cu2-O2	-	2.364	-	N2-Cu2-O1	83.717	80.518	84.155
O1-H1	0.978	0.979	1.006	N7-Cu2-O1	94.612	88.642	88.965

H1-O3	-	-	1.691	N7-Cu2-N6	94.004	-	83.573
				N7-Cu2-N8	111.436	105.768	107.310
				N7-Cu2-O2	-	118.937	-
				N2-Cu2-O2	-	101.641	-
				O1-Cu2-O2	-	83.577	-
				N4-Cu1-O2	-	-	174.943
				N1-Cu1-O2	-	-	87.505
				O1-Cu1-O2	-	-	83.834
				Cu2-O1-H1	117.394	112.401	112.699

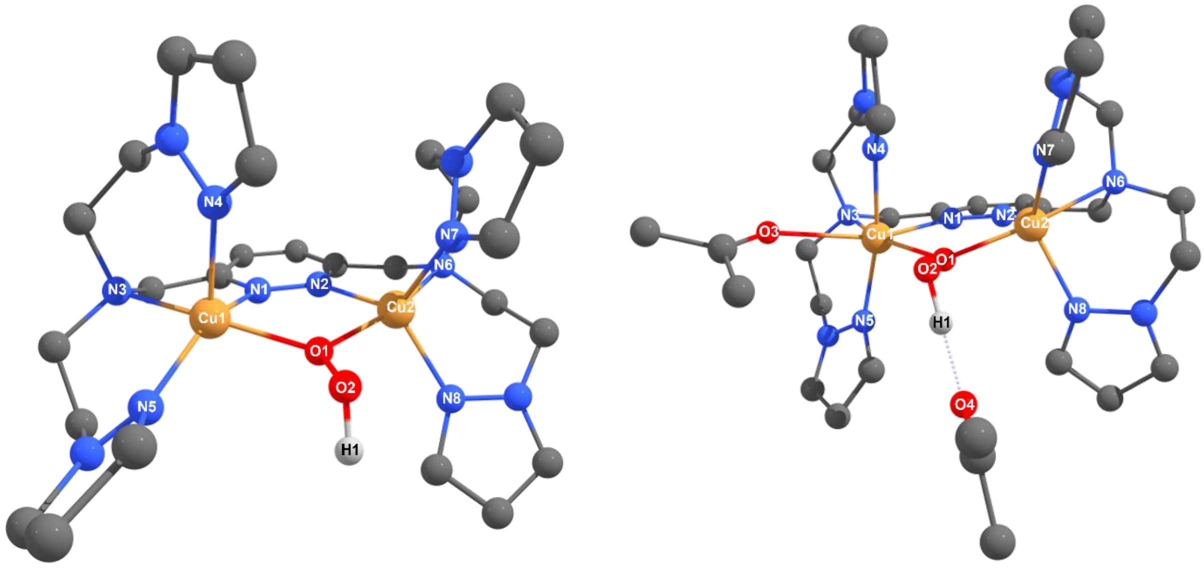

Figure S108: Geometry optimization of the **Cu_2OOH** complex **5** without (left) and with additional acetone ligands (right). The second acetone molecule is bound only *via* hydrogen bonds (dashed line in light blue) to the bridging hydroperoxo unit. The most promising DFT-structures are shown. All hydrogen atoms attached to carbon atoms have been omitted for clarity. Selected bond lengths and angles are given below in Table S13. DFT: RI-PBE-D3(BJ)/def2-SVP.

Table S13: Selected bond lengths (Å), including Cu···Cu distances (Å) and angles (°) for the **Cu_2OOH** species of the Cu_2(**MO8**) model system obtained by DFT. DFT: RI-PBE-D3(BJ)/def2-SVP. Note: The numbering of the atoms is based on the DFT-optimized structures from Figure S108. The value in green indicates that this is a hydrogen bond between the bridging hydroperoxo unit and the second acetone molecule.

Cu_2(**MO8**) system	**Cu_2OOH**	**Cu_2OOH with one $CO(CH_3)_2$**	Cu_2(**MO8**) system	**Cu_2OOH**	**Cu_2OOH with one $CO(CH_3)_2$**
**Bond lengths / Å**			**Bond angles / °**		
Cu1···Cu2	3.527	3.567	Cu1-O1-Cu2	122.478	121.393
Cu1-N1	2.041	2.250	N1-Cu1-N3	82.224	75.879
Cu1-N3	2.189	2.242	N1-Cu1-N4	96.817	89.859
Cu1-N4	2.119	2.056	N1-Cu1-N5	150.279	105.447
Cu1-N5	1.976	2.027	N1-Cu1-O1	83.065	82.148
Cu2-N2	2.025	2.035	N4-Cu1-O1	93.870	86.912

Cu2-N6	2.198	2.307	N4-Cu1-N3	95.070	94.763
Cu2-N7	2.061	2.019	N4-Cu1-N5	112.900	164.147
Cu2-N8	2.025	2.060	N2-Cu2-N6	82.280	78.648
Cu1-O1	2.024	2.080	N2-Cu2-N7	124.801	132.167
Cu2-O1	1.999	2.010	N2-Cu2-N8	128.314	105.103
Cu1-O3	-	2.421	N2-Cu2-O1	84.164	88.732
O1-O2	1.391	1.394	N7-Cu2-O1	94.620	99.643
O2-H1	0.988	1.038	N7-Cu2-N6	91.738	87.632
H1-O4	-	1.539	N7-Cu2-N8	106.804	120.320
			N4-Cu1-O3	-	81.963
			N1-Cu1-O3	-	156.106
			O1-Cu1-O3	-	119.505
			N3-Cu1-O3	-	82.442
			N5-Cu1-O3	-	85.394
			O1-Cu1-O3	-	119.505
			O2-H1-O4	-	176.764
			O1-O2-H1	103.873	104.643

The geometry optimization of a tetranuclear mixed-valent *μ*$_4$-peroxo complex (**Cu_4O_2**) led to a structure in which a **2**-PF_6 unit separates from the **Cu_4O_2** core resulting in a **Cu_2O_2** species. This could indicate that the **Cu_4O_2** species, as observed with the ligands **bdpdz**/**bdptz**, is not stabilized by the ligand **MO8**. The reason for this could be a higher steric demand of the ligand **MO8**.

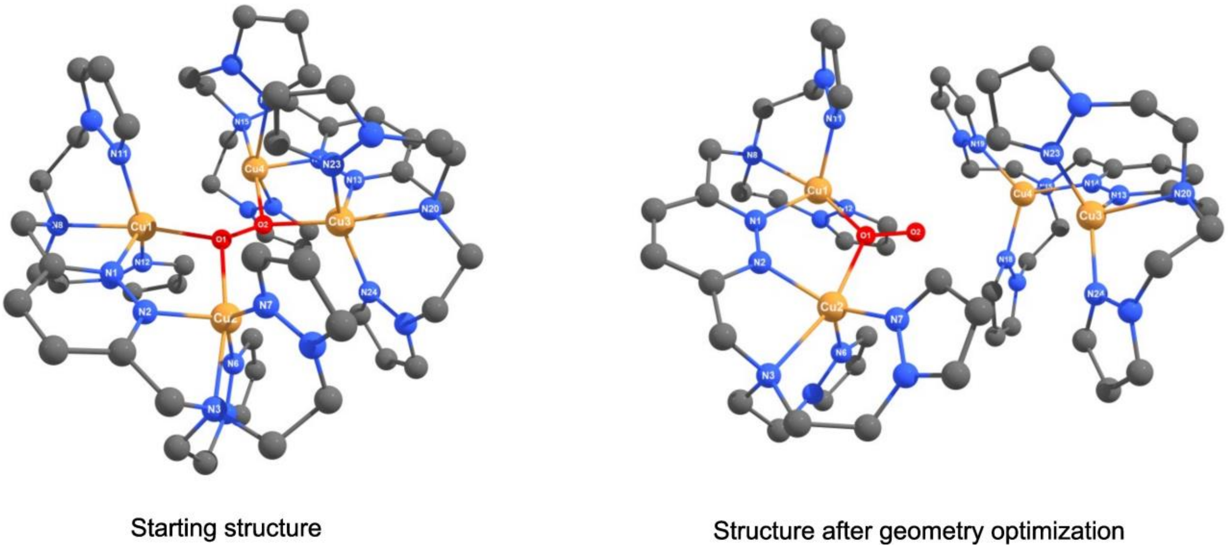

Figure S109: Geometry optimization of the **Cu_4O_2** complex. Starting structure before geometry optimization (left) and structure obtained after geometry optimization (right). The result is a structure in which a **2**-PF_6 separates from the **Cu_4O_2** core, leading to a **Cu_2O_2** species. All hydrogen atoms attached to carbon atoms have been omitted for clarity. DFT: RI-PBE-D3(BJ)/def2-SVP.

12.2.Comparison with the crystal structures 2-PF_6 and 3-PF_6

Confirmation of the quality of the performed geometry optimizations is also obtained from comparing selected bond length, Cu···Cu distances and angles with the obtained crystal structure for the dicopper(I) complex supported by the ligand **MO8** **2**-PF_6. In the solid form no additional acetonitrile coligands complete the coordination sphere of the dinuclear copper(I) complexes (as synthesized form, proven by use of NMR spectroscopy). Moreover, the bare copper(I) ions also appear to be stable in an acetone solution – only stabilized by the multidentate ligand **MO8** – and exhibit the same coordination sphere as in the solid state.

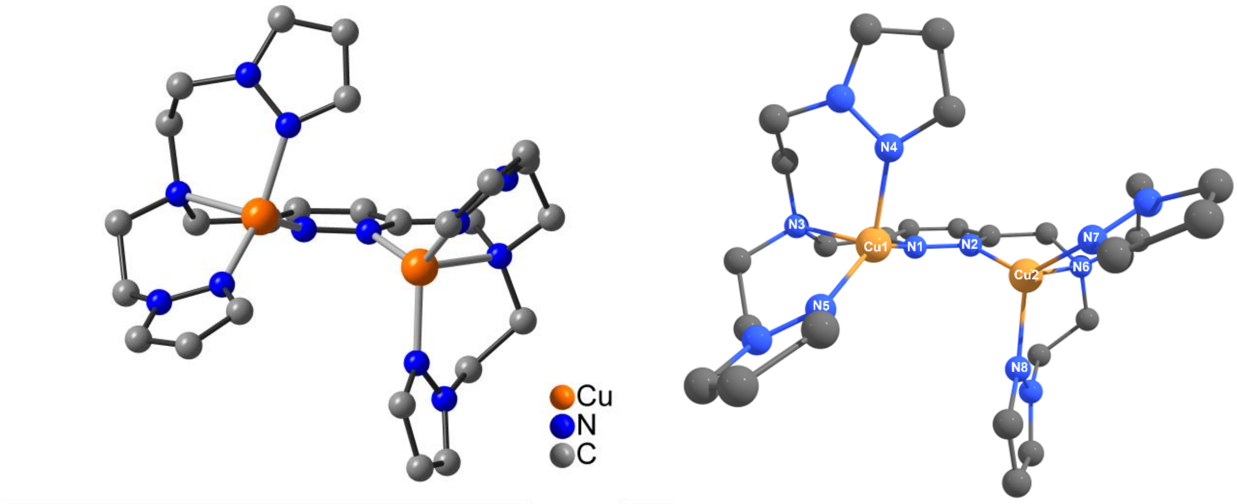

Figure S110: Left: Molecular structure of the dinuclear complex bearing **MO8** as ligand (= **2**-PF_6). Crystal system: monoclinic. Space group: $P2_1/n$. Right: DFT-optimized structure of the dicopper(I) complex **2**. All hydrogen atoms have been omitted for clarity. Selected bond lengths and angles are shown in Table S14. DFT: RI-PBE-D3(BJ)/def2-SVP.

Table S14: Comparison of the obtained data from the crystal structure of **2**-PF_6 and the theoretical data for the complex **2**. Selected bond lengths (Å), including Cu···Cu distances (Å) and angles (°) are given for these systems. DFT: RI-PBE-D3(BJ)/def2-SVP. Note: The numbering of the atoms is based on the DFT-optimized structures from Figure S110. To compare them with the numbering of the atoms in the crystal structure, these numbers are also given in brackets in blue color.

[Cu_2(**MO8**)] system	**Crystal structure** (**2**-PF_6)	**2** (DFT)	[Cu_2(**MO8**)] system	**Crystal structure** (**2**-PF_6)	**2** (DFT)
**Bond lengths / Å**			**Bond angles / °**		
Cu1···Cu2	3.4883	3.305	N1(N1)-Cu1-N3(N3)	81.31(5)	81.076
Cu1-N1(N1)	1.9862(12)	1.949	N1(N1)-Cu1-N4(N7)	128.78(5)	104.749
Cu1-N3(N3)	2.2229(12)	2.281	N1(N1)-Cu1-N5(N5)	112.94(5)	141.369
Cu1-N4(N7)	1.9515(12)	2.016	N2(N2)-Cu2-N6(N8)	79.93(5)	81.145
Cu1-N5(N5)	1.9771(13)	1.956	N2(N2)-Cu2-N7(N10)	98.62(5)	137.374
Cu2-N2(N2)	2.0340(12)	1.954	N2(N2)-Cu2-N8(N12)	125.76(5)	108.479
Cu2-N6(N8)	2.2164(12)	2.293			
Cu2-N7(N10)	2.0052(13)	1.963			
Cu2-N8(N12)	1.9424(13)	2.006			

The geometry optimization of the **Cu_2OH** complex **3** resulted in a structure which is slightly different compared to the crystal structure of **3**-PF_6. In both cases the copper centers exhibit a distorted square pyramidal geometry. However, the pyrazole units outside the square plane in the crystal structure point in opposite directions, whereas in the geometry optimization they point in the same direction. In general the crystal structure of the **Cu_2OH** complex **3** is more symmetrical than predicted by the DFT calculation.

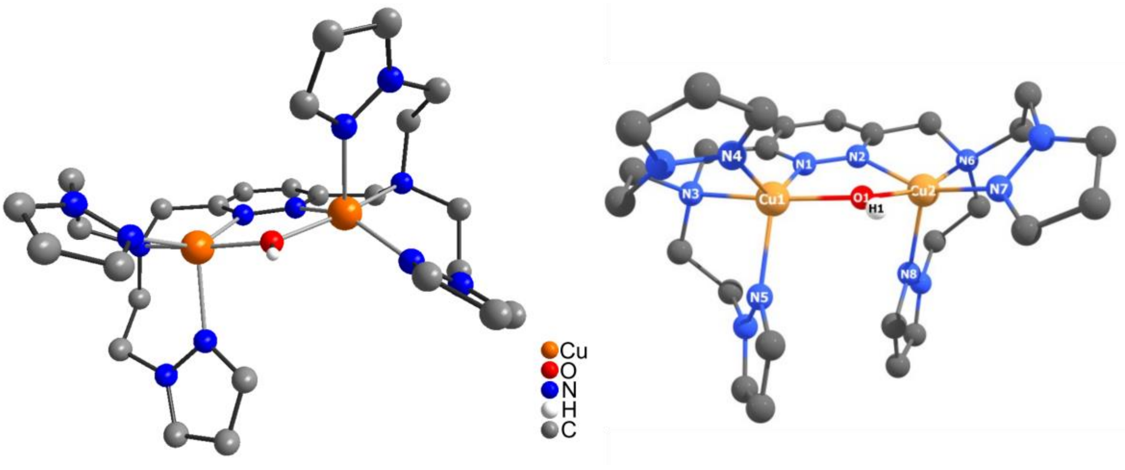

Figure S111: Left: Crystal structure of the **Cu_2OH** complex **3**-PF_6. Crystal system: orthorhombic. Space group: Pbcn. Right: DFT-optimized structure of the **Cu_2OH** complex **3**. All hydrogen atoms attached to carbon atoms have been omitted for clarity. Selected bond lengths and angles are shown below in in Table S15. DFT: RI-PBE-D3(BJ)/def2-SVP.

Table S15: Comparison of the obtained data from the crystal structure of **3**-PF_6 and the theoretical data for the **Cu_2OH** complex **3**. Selected bond lengths (Å), including Cu···Cu distances (Å) and angles (°) are given for these systems. DFT: RI-PBE-D3(BJ)/def2-SVP. Note: The numbering of the atoms is based on the DFT-optimized structures from Figure S107. To compare them with the numbering of the atoms in the crystal structure, these numbers are also given in brackets in blue color.

**Cu_2OH** complex	**Crystal structure (3**-PF_6**)**	**3** (DFT)	**Cu_2OH** complex	**Crystal structure (3**-PF_6**)**	**3** (DFT)
**Bond lengths / Å**			**Bond angles / °**		
Cu1···Cu2	3.4200	3.460	N2(N1 / N2)-Cu1-N1(N4 / N7)	111.26(6)	132.297 / 155.176
Cu1-N1(N4 / N7)	2.1296(15)	2.021 / 1.964	N2(N1 / N2)-Cu1-N6(N3 / N6)	81.65(6)	81.060 / 81.944
Cu1-N2(N1 / N2)	1.9964(12)	2.047 / 2.038	N3(N5 / N8)-Cu1-N1(N4 / N7)	99.09(6)	102.381 / 111.436
Cu1-N3(N5 / N8)	1.9843(15)	2.819 / 2.146	N3(N5 / N8)-Cu1-N2(N1 / N2)	149.49(6)	125.169 / 93.335
Cu1-N6(N3 / N6)	2.0984(14)	2.184 / 2.170	N3(N5 / N6)-Cu1-N6(N3 / N6)	93.34(6)	91.303 / 94.682
Cu1-O1	1.9301(8)	1.953	N6(N3 / N6)-Cu1-N1(N4 / N7)	124.74(9)	95.575 / 94.004
			O1(O1)-Cu1-N1(N4 / N7)	89.38(4)	96.172 / 94.612
			O1(O1)-Cu1-N2(N1 / N2)	85.84(6)	83.838 / 83.717
			O1(O1)-Cu1-N3(N5 / N8)	97.89(6)	95.645 / 96.583
			O1(O1)-Cu1-N6(N3 / N6)	167.46(6)	164.744 / 162.229
			Cu1-O1(O1)-Cu1	124.74(9)	124.722

12.3. Geometry optimization, selected bond lengths and angles of a *μ*-1,2 peroxo complex

The DFT-calculated structure of the *μ*-1,2 peroxo complex shows a similar twisted Cu-O-O-Cu unit as the dinuclear system by MEYER *et al*.[28] However, based on our measurements, the *μ*-1,2 **Cu_2O_2** species can be excluded as a copper oxygen intermediate in **MO8**, since such a species would probably provide a qualitatively similar UV/Vis spectrum. In addition, *μ*-1,2-**Cu_2O_2** species have characteristic Raman bands, which would be about 70-80 cm^{-1} lower than those of **Cu_2OOH** complexes.[28,37]

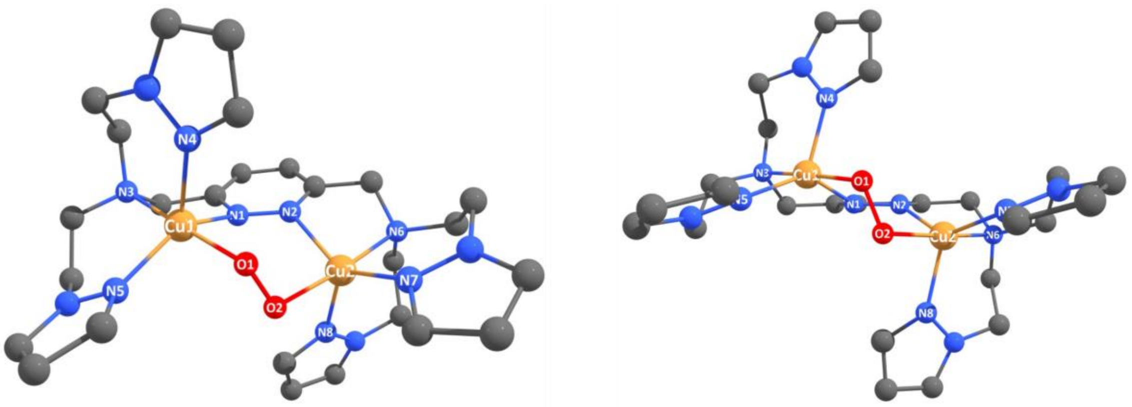

Figure S112: Geometry optimization of the *μ*-1,2 **Cu_2O_2** complex from an angled perspective (left) and for a better clarity of the Cu-O-O-Cu torsion angle from below (right). All hydrogen atoms attached to carbon atoms have been omitted for clarity. Selected bond lengths and angles are given below in Table S16. DFT: RI-PBE-D3(BJ)/def2-SVP.

Table S16: Selected bond lengths (Å), including Cu···Cu distances (Å) and angles (°) for the **Cu_2O_2** species of the Cu_2(**MO8**) model system obtained by DFT. DFT: RI-PBE-D3(BJ)/def2-SVP. Note: The numbering of the atoms is based on the DFT-optimized structure from Figure S112.

Cu_2(**MO8**) system	**Cu_2O_2**	Cu_2(**MO8**) system	**Cu_2O_2**
**Bond lengths / Å**		**Bond angles / °**	
Cu1···Cu2	3.864	Cu1-O1-O2-Cu2	89.275
Cu1-N1	2.082	N1-Cu1-N3	80.916
Cu1-N3	2.269	N1-Cu1-N4	101.327
Cu1-N4	2.058	N1-Cu1-N5	138.802
Cu1-N5	2.020	N1-Cu1-O1	94.801
Cu2-N2	2.082	N4-Cu1-O1	87.544
Cu2-N6	2.273	N4-Cu1-N3	94.380
Cu2-N7	2.020	N4-Cu1-N5	119.827
Cu2-N8	2.058	N2-Cu2-N6	80.889
Cu1-O1	2.051	N2-Cu2-N7	137.618
Cu2-O2	2.051	N2-Cu2-N8	102.362
O1-O2	1.301	N2-Cu2-O2	94.613
		N7-Cu2-O2	89.525
		N7-Cu2-N6	93.441
		N7-Cu2-N8	119.976

13. Reactivity of the Model Complex

13.1. Reactivity of the green species at rt: General procedure for reactivity studies of the copper(I) complexes towards external substrates

In a typical reaction the copper(I) precursor **2**-PF_6 or **2**-OTf (1-2 mM, 1 eq.) was dissolved in dry acetone (6 mL) under an inert gas atmosphere. O_2 was added at +35 °C (308 K) *via* a cannula through the septum with a pressure slightly above 1 atm for a slow gas flow through the reaction vessel. To ensure quantitative formation of the copper-oxygen intermediate, the addition of O_2 was continued for at least 2 h (this is equivalent to the use of oxygen saturated solvent). The substrate (10 eq.) was also applied directly when oxygen was added. The reaction mixture was stirred for 24 h at +35 °C without bubbling of O_2, but under an O_2 atmosphere. After treatment with a saturated solution of Na_2-EDTA (10 mL) the reaction was stopped and the organic phase was separated, the aqueous phase was extracted with dichloromethane (2x 20 mL), the combined organic phases were dried with sodium sulfate, filtered and solvents were removed under reduced pressure. Characterization of the obtained residues was done by means of GC mass spectrometric measurements (vide infra). Quantification of products was based on preparing calibration curves of authentic standards (see below). Reactions were carried out at least duplicate; values presented in this work are averaged. Blind reactions were also performed in the same manner but without the copper complexes (in case of AT) as well as using $[Cu(NCMe)_4]PF_6$ or $[Cu(NCMe)_4]OTf$ instead of **2**-PF_6 or **2**-OTf. All control experiments lead to poorer results compared to the activity with **2**-PF_6 or **2**-OTf (see below).

No reaction was observed upon addition of 9,10-dihydroanthracene (DHA; BDE = 78 kcal mol^{-1}) or diphenylmethane (DPM; BDE = 82 kcal mol^{-1}) to the complexes of **2**-PF_6 or **2**-OTf, suggesting that the BDE limit of our catalysts has been reached. Moreover, no reaction was observed upon addition of 9*H*-xanthene (XEN; BDE = 75 kcal mol^{-1}). XEN has a slightly lower BDE (C-H) than anthrone (AT; BDE = 76 kcal mol^{-1}). However, in solution the anthrone is in an equilibrium with the anthrol form *via* keto-enol tautomerism, which exhibits a BDE (O-H) of 72 kcal mol^{-1}.[38] In this case the H-atom abstraction might occur *via* an O-H abstraction.

13.2.Reactivity of the green species at rt with Anthrone (AT)

For the calibration we used different ratios of 10*H*-anthracen-9-one (anthrone, AT) and 9,10-anthraquinone (AQ) with 2 mL of a 2 mM mesitylene solution in dichloromethane (DCM). In all cases 2 µL of the reaction mixtures were injected into the GC-MS.

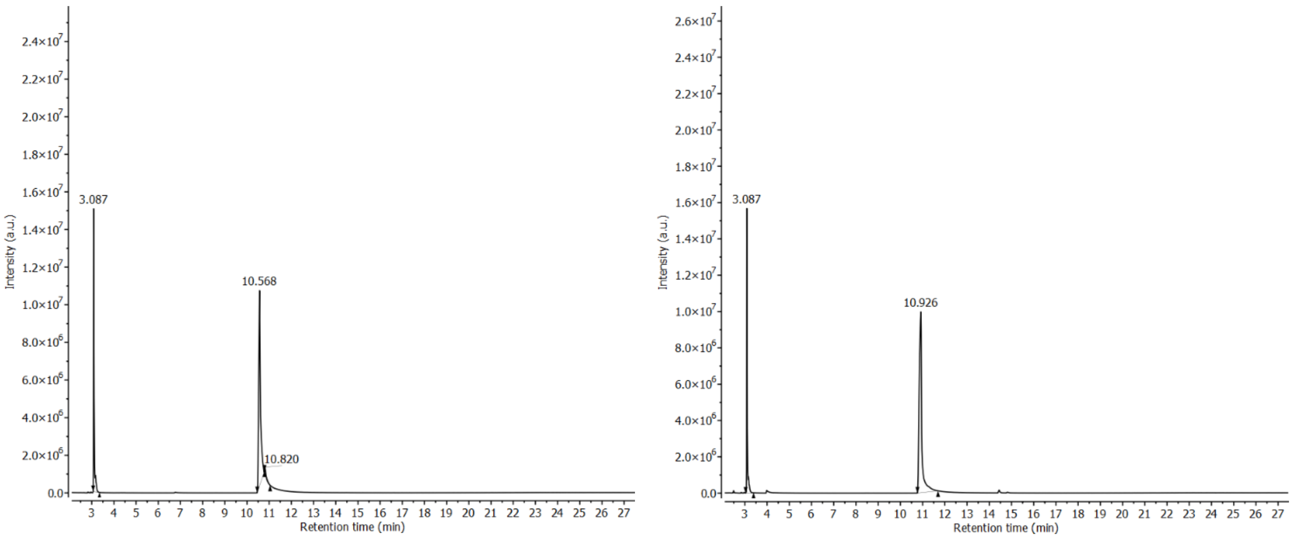

Figure S113: Chromatograms of the calibration for the reactivity towards anthrone; mesitylene (t_R = 3.09 min), anthrone (left chromatogram, t_R = 10.57 min) and anthraquinone (right chromatogram, t_R = 10.93 min).

The obtained results of the reactivity studies with the green species are summarized in the following table (Table S17). In blind experiments, the catalyst **2** was omitted or replaced by $[Cu(NCMe)_4]PF_6$ (**CuP**-PF_6) and $[Cu(NCMe)_4]OTf$ (**CuP**-OTf), respectively, leading to significantly less conversion. In the absence of copper, anthrone is already converted to anthraquinone to a small extent under the conditions of catalysis. However, the amount converted is significantly lower compared to the experiment with **2**. All control experiments lead to poorer results compared to the activity with **2**.

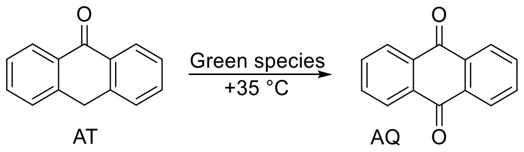

Scheme S1: Reactivity of the green species towards AT leading to AQ.

Table S17: Overview of the obtained results for the reactivity towards anthrone; 10 equiv. (excess) of AT were used.

System	AT / %	AQ / %	TON
**CuP**-OTf + O_2 (35 °C)	88.8	11.2	0.6[a]
**CuP**-PF_6 + O_2 (35 °C)	82.1	17.9	0.9[a]
**2**-OTf + O_2 (35 °C)	11.9	88.1	8.8
**2**-PF_6 + O_2 (35 °C)	7.4	92.6	9.3
Only AT + O_2 (35 °C)	94.0	6.0	-
**bdpdz**-OTf[b]	83.8	16.2	1.6
**bdptz**-OTf[b]	83.6	16.4	1.6
**CuP**-OTf[b]	80.1	19.9	2.0

TON = Turnover Number; is defined as the equiv. of product made per equiv. of catalyst; 10 equiv. of anthrone were used. Conditions for the Cu_2(**MO8**) model systems: +35 °C, 2 h O_2 bubbling in the beginning; total time of the reaction: 24 h. [a] Since the precursors are mononuclear complexes, factor 2 must be considered to compare the TON with the model systems. [b] The **Cu_2O** complex of the **bdpdz**/**bdptz** system was reacted at -35 °C with the substrate for 2 h and additional 2 h at room temperature. Total time of the reaction: 4 h. For additional information, see the text below.

To compare the results with respect to the reactivity toward anthrone with our previous model systems,[34] **bdpdz** and **bdptz** were also examined for their ability to convert anthrone to anthraquinone. In this case, the following protocol was used:
In a typical reaction the copper(I) precursor $[Cu_2($**bdpdz**$)(NCMe)_2](PF_6)_2$, $[Cu_2($**bdptz**$)(NCMe)_2](OTf)_2$ or $[Cu_2($**bdptz**$)(NCMe)_2](PF_6)_2$ $[Cu_2($**bdptz**$)(NCMe)_2](OTf)_2$ (1-2 mM, 1 eq.) was dissolved in dry acetone (15 mL) under an inert gas atmosphere. O_2 was added at -35 °C (238 K) *via* a cannula through the septum with a pressure slightly above 1 atm for a slow gas flow through the reaction vessel. Color change to green was observed after 30 seconds. To ensure quantitative formation of the **Cu_2O** complex the addition of O_2 was continued for 15 minutes. Then anthrone (10 eq.) was added. The reaction mixture was stirred for 2 h at -35 °C without bubbling O_2, but under an O_2 atmosphere, and for additional 2 h at room temperature. After 45 min at room temperature extra dioxygen was added via the cannula for 10 min; then, the solution was stirred for another hour at room temperature under O_2 atmosphere. After treatment with a saturated solution of Na_2-EDTA (10 mL) the reaction was stopped and the organic phase was separated, the aqueous phase was extracted with dichloromethane (2x 15 mL), the combined organic phases were dried with sodium sulfate, filtered and solvents were removed under reduced pressure. Characterization of the obtained residues was done by means of GC mass spectrometric measurements. Reactions were carried out at least duplicate; values presented in this work are averaged. Blind reactions were also performed in the same manner using $[Cu(NCMe)_4]PF_6$ or $[Cu(NCMe)_4]OTf$ instead of $[Cu_2($**bdpdz**$)(NCMe)_2](PF_6)_2$, $[Cu_2($**bdptz**$)(NCMe)_2](OTf)_2$ or $[Cu_2($**bdptz**$)(NCMe)_2](PF_6)_2$ $[Cu_2($**bdptz**$)(NCMe)_2](OTf)_2$. All control experiments lead to poorer results compared to the activity of the model systems **bdpdz** and **bdptz**. The obtained results are summarized in Table S17.

13.3. Reactivity of the Cu_2OOH complex: General procedure for reactivity studies of the copper(I) complexes towards external substrates at low temperatures

In a typical reaction the copper(I) precursor **2**-PF_6 or **2**-OTf (1-2 mM, 1 eq.) was dissolved in dry acetone (6 mL) under an inert gas atmosphere. O_2 was added at -90 °C (183 K) *via* a cannula through the septum with a pressure slightly above 1 atm for a slow gas flow through the reaction vessel. To ensure quantitative formation of the **Cu_2OOH** complex **5** the addition of O_2 was continued for at least 2 h (this is equivalent to the use of oxygen saturated solvent). The substrate (DHA: 5 eq.; AT: 5 eq.) was applied after the formation of the **Cu_2OOH** species. The reaction mixture was stirred for 6 h at -90 °C without bubbling of O_2, but under an O_2 atmosphere. After treatment with a saturated solution of Na_2-EDTA (10 mL) the reaction was stopped and the organic phase was separated, the aqueous phase was extracted with dichloromethane (2x 20 mL), the combined organic phases were dried with sodium sulfate, filtered and solvents were removed under reduced pressure. Characterization of the obtained residues was done by means of GC mass spectrometric measurements (vide infra). Quantification of products was based on preparing calibration curves of authentic standards (see above). Reactions were carried out at least duplicate; values presented in this work are averaged. Blind reactions were also performed in the same manner but without the copper complexes (in case of AT) as well as using $[Cu(NCMe)_4]PF_6$ or $[Cu(NCMe)_4]OTf$ instead of **2**-PF_6 or **2**-OTf. All control experiments lead to poorer results compared to the reactivity with **2**-PF_6 or **2**-OTf (see below).

Furthermore, the same experiment was performed using H_2O_2 as a source of reduction equivalents to achieve catalytic conversion of anthrone to anthraquinone. After formation of the **Cu_2OOH** complexes **5**-PF_6 or **5**-OTf and addition of the substrate, H_2O_2 was added (1.16 mM, 110 eq.) at -90 °C. The reaction mixture was stirred for 6 h at -90 °C without bubbling of O_2, but under an O_2 atmosphere in the presence of excess H_2O_2. The subsequent workup was analogous to that described above. Characterization of the obtained residues was done by means of GC mass spectrometric measurements. Blind reactions were also performed in the same manner but without the copper complexes as well as using $[Cu(NCMe)_4]PF_6$ or $[Cu(NCMe)_4]OTf$ instead of **2**-PF_6 or **2**-OTf. All control experiments lead to poorer results compared to the reactivity with **2**-PF_6 or **2**-OTf (see Table S18).

13.4. Reactivity of the Cu_2OOH complex with Anthrone (AT)

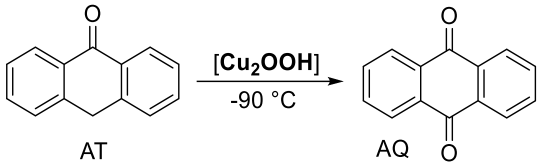

Scheme S2: Reactivity of the μ-1,1-hydroperoxo dicopper(II) complex towards AT at low temperatures leading to AQ as well.

Table S18: Overview of the obtained results for the reactivity of the **Cu_2OOH** species towards anthrone; 5 equiv. (excess) of AT were used.

System	AT / %	AQ / %	TON
Only AT + O_2 (-90 °C)	98.0	2.0	-
**CuP**-OTf + O_2 (-90 °C)	85.9	14.1	0.4[a]
**CuP**-PF_6 + O_2 (-90 °C)	87.6	12.4	0.3[a]
**2**-OTf + O_2 (-90 °C)	80.6	19.4	1.0
**2**-PF_6 + O_2 (-90 °C)	79.0	21.0	1.1
**CuP**-OTf + O_2 (-90 °C) + H_2O_2	91.2	9.8	0.2
**CuP**-PF_6 + O_2 (-90 °C) + H_2O_2	86.9	13.1	0.3
**2**-OTf + O_2 (-90 °C) + H_2O_2	78.3	21.7	1.1
**2**-PF_6 + O_2 (-90 °C) + H_2O_2	83.1	16.9	0.9

TON = Turnover Number; is defined as the equiv. of product made per equiv. of catalyst; 5 equiv. of anthrone were used. Reduction equivalents in the form of H_2O_2 were added to test whether catalytic conversion (TON > 1) could be achieved; 110 eq. of H_2O_2 were added at -90 °C. [a] Since the precursors are mononuclear complexes, factor 2 must be considered to compare the TON with the model systems.

13.5. Reactivity of the Cu_2OH complex with Anthrone (AT) at rt:

3 mg (2.71 µmol, 1 eq.) of the **Cu_2OH** complex **3**-OTf was dissolved in 3 mL dry acetone under an inert gas atmosphere. Then 2.63 mg (13.6 µmol 5 eq.) anthrone was added. In the first attempt, the reaction mixture was stirred for 24 h at room temperature under an inert gas atmosphere. In a second attempt, the reaction was carried out under the same conditions as the green species (addition of O_2 for 2 h and reaction for 24 h). This was followed by a quench with a saturated solution of Na_2-EDTA (10 mL). Then the aqueous phase was extracted with dichloromethane (3x 20 mL). The organic phase was dried with sodium sulfate, filtered and the solvents were removed under reduced pressure.

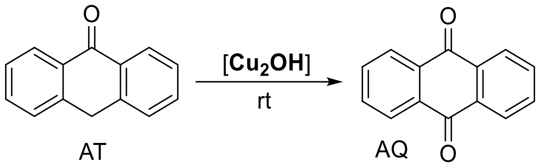

Scheme S3: Reactivity of the **Cu_2OH** complex **3**-OTf towards anthrone at room temperature leading to only small amounts of anthraquinone.

Table S19: Overview of the obtained results for the reactivity of the **Cu_2OH** species towards anthrone; 5 equiv. (excess) of AT were used.

System	AT / %	AQ / %	TON
Only AT (rt)	97.8	2.2	-
**3**-OTf (inert, rt)	95.5	4.5	0.1
**3**-OTf (+O_2, rt)	86.8	13.2	0.6

TON = Turnover Number; is defined as the equiv. of product made per equiv. of catalyst; 5 equiv. of anthrone were used.

Part IV: Appendix and References

14. Appendix

14.1. Data Availability

All data supporting the findings of this study are available within this article (main paper) and its Supporting Information or are available from the corresponding authors (M. R. or F. T.) upon reasonable requests. Crystallographic data for the structure reported in this article has been deposited at the Cambridge Crystallographic Data Centre, under the following deposition number: CCDC-2149824 (compound **2**-PF_6) and CCDC-2419891 (**3**-PF_6) and can be obtained free of charge *via* http://www.ccdc.cam.ac.uk/data_request/cif.

14.2. Author Contributions

F. T. and R. J. planned and executed the synthesis, characterization and reactivity studies. A. S. performed synthesis as well and also took care of reactivity studies. R. J. and A. S. performed the DFT calculations. Single Crystal Structure Determination was performed by C. N. and J. K. Cryo-UHR-ESI mass spectrometry was performed by L. S. and I. K. and supervised by I. I.-B. The GC-MS measurements were done by Y. A. Resonance Raman and XAS measurements were performed by B. G.-L., R. J., A. S., S. B. and M.A.N. and supervised by M. R. The analysis of the XAS data were done by M. A. N., B. G.-L. and M. R. All authors commented on the manuscript. F. T. and R. J. conceived the study and wrote the manuscript together with A. S., M. A. N., B. G.-L. and M. R.

((Please specify the contributions of each author including the type (e.g. data curation, funding acquisition, formal analysis, investigation, project administration, validation, writing of original draft) and the degree (e.g. lead, equal, supporting) of contribution.))

15. References

[1] G. M. Sheldrick, *Acta Crystallogr A Found Adv* **2015**, *71*, 3.
[2] G. M. Sheldrick, *Acta Crystallogr C Struct Chem* **2015**, *71*, 3.
[3] B. Schulz, J. Bäckström, D. Budelmann, R. Maeser, M. Rübhausen, M. V. Klein, E. Schoeffel, A. Mihill, S. Yoon, *Rev. Sci. Instrum.* **2005**, *76*.
[4] B. Grimm-Lebsanft, C. Brett, F. Strassl, D. Rukser, M. Biednov, F. Biebl, M. Naumova, A. Hoffmann, L. Akinsinde, D. Brückner et al., *Inorg. Chim. Acta.* **2018**, *481*, 176.
[5] E. Welter, R. Chernikov, M. Herrmann, R. Nemausat, *AIP Conf. Proc.* **2019**, *2054*, 40002.
[6] B. Ravel, M. Newville, *J. Synchrotron Rad.* **2005**, *12*, 537.
[7] "https://github.com/aklnk/xaesa".
[8] M. Newville, *J. Phys.: Conf. Ser.* **2013**, *430*, 12007.
[9] J. Timoshenko, A. Kuzmin, J. Purans, *J. Phys. Condens. Matter.* **2014**, *26*, 55401.
[10] a) F. Neese, *Wiley Interdiscip. Rev.: Comput. Mol. Sci.* **2012**, *2*, 73; b) F. Neese, *Wiley Interdiscip. Rev.: Comput. Mol. Sci.* **2018**, *8*; c) F. Neese, F. Wennmohs, U. Becker, C. Riplinger, *J. Chem. Phys.* **2020**, *152*, 224108.
[11] J. P. Perdew, K. Burke, M. Ernzerhof, *Phys. Rev. Lett.* **1996**, *77*, 3865.
[12] F. Weigend, R. Ahlrichs, *Phys. Chem. Chem. Phys.* **2005**, *7*, 3297.
[13] a) S. Grimme, J. Antony, S. Ehrlich, H. Krieg, *J. Chem. Phys.* **2010**, *132*, 154104; b) S. Grimme, S. Ehrlich, L. Goerigk, *J. Comput. Chem.* **2011**, *32*, 1456.
[14] a) F. Neese, *J. Comput. Chem.* **2003**, *24*, 1740; b) K. Eichkorn, F. Weigend, O. Treutler, R. Ahlrichs, *Theor. Chem. Acc.* **1997**, *97*, 119; c) K. Eichkorn, O. Treutler, H. Öhm, M. Häser, R. Ahlrichs, *Chem. Phys. Lett.* **1995**, *240*, 283.
[15] F. Weigend, *Phys. Chem. Chem. Phys.* **2006**, *8*, 1057.
[16] A. D. Becke, *J. Chem. Phys.* **1993**, *98*, 5648.
[17] V. Barone, M. Cossi, *J. Phys. Chem. A* **1998**, *102*, 1995.
[18] F. Neese, F. Wennmohs, A. Hansen, U. Becker, *Chem. Phys.* **2009**, *356*, 98.
[19] J. Kuzelka, S. Mukhopadhyay, B. Spingler, S. J. Lippard, *Inorg. Chem.* **2004**, *43*, 1751.
[20] J. R. Cubanski, S. A. Cameron, J. D. Crowley, A. G. Blackman, *Dalton Trans.* **2013**, *42*, 2174.
[21] Woll et al, *United States Patent US 9,617,268 B2* **2017**.
[22] V. Gierz, C. Maichle-Mössmer, D. Kunz, *Organometallics* **2012**, *31*, 739.
[23] R. Bhowmick, A. S. M. Islam, A. Giri, A. katarkar, M. Ali, *New J. Chem.* **2017**, *41*, 11661.
[24] J. Kuzelka, S. Mukhopadhyay, B. Spingler, S. J. Lippard, *Inorg. Chem.* **2004**, *43*, 1751.
[25] H. Saltzman, J. G. Sharefkin, *Org. Synth.* **1963**, *43*, 60.
[26] W. Adam, J. Bialas, L. Hadjiarapoglou, *Chem. Ber.* **1991**, *124*, 2377.
[27] a) W. Adam, Y. Y. Chan, D. Cremer, J. Gauss, D. Scheutzow, M. Schindler, *J. Org. Chem.* **1987**, *52*, 2800; b) D. F. Taber, P. W. DeMatteo, R. A. Hassan, *Org. Synth.* **2013**, *90*, 350.
[28] N. Kindermann, S. Dechert, S. Demeshko, F. Meyer, *J. Am. Chem. Soc.* **2015**, *137*, 8002.
[29] a) K. Itoh, H. Hayashi, H. Furutachi, T. Matsumoto, S. Nagatomo, T. Tosha, S. Terada, S. Fujinami, M. Suzuki, T. Kitagawa, *J. Am. Chem. Soc.* **2005**, *127*, 5212; b) K. D. Karlin, R. W. Cruse, Y. Gultneh, *J. Chem. Soc., Chem. Commun.* **1987**, 599; c) L. Li, A. A. Narducci Sarjeant, M. A. Vance, L. N. Zakharov, A. L. Rheingold, E. I. Solomon, K. D. Karlin, *J. Am. Chem. Soc.* **2005**, *127*, 15360; d) M. Mahroof-Tahir, N. N. Murthy, K. D. Karlin, N. J. Blackburn, S. N. Shaikh, J. Zubieta, *Inorg. Chem.* **1992**, *31*, 3001; e) N. N. Murthy, M. Mahroof-Tahir, K. D. Karlin, *Inorg. Chem.* **2001**, *40*, 628; f) T. N. Sorrell, V. A. Vankai, *Inorg. Chem.* **1990**, *29*, 1687.
[30] a) N. D. Yordanov, D. Shopov, *J. Inorg. Nucl. Chem.* **1976**, *38*, 137; b) S. Torelli, C. Belle, I. Gautier-Luneau, J. L. Pierre, E. Saint-Aman, J. M. Latour, L. Le Pape, D. Luneau, *Inorg. Chem.* **2000**, *39*, 3526; c) R. Pogni, M. C. Baratto, E. Busi, R. Basosi, *J. Inorg. Biochem.* **1999**, *73*, 157; d) J. Klingele, B. Moubaraki, K. S. Murray, J. F. Boas, S. Brooker, *Eur. J Inorg. Chem.* **2005**, 1530; e) A. Kumar Singh, J. I. van der Vlugt, S. Demeshko, S. Dechert, F. Meyer, *Eur. J Inorg. Chem.* **2009**, 3431.
[31] a) H. V. Obias, Y. Lin, N. N. Murthy, E. Pidcock, E. I. Solomon, M. Ralle, N. J. Blackburn, Y.-M. Neuhold, A. D. Zuberbühler, K. D. Karlin, *J. Am. Chem. Soc.* **1998**, *120*, 12960; b) E. I. Solomon, D. E. Heppner, E. M. Johnston, J. W. Ginsbach, J. Cirera, M. Qayyum, M. T. Kieber-Emmons, C. H. Kjaergaard, R. G. Hadt, L. Tian, *Chem. Rev.* **2014**, *114*, 3659; c) W. B. Tolman, *Acc. Chem. Res.* **1997**, *30*, 227; d) K. D. Karlin, P. Ghosh, R. W. Cruse, A. Farooq, Y. Gultneh, R. R. Jacobson, N. J. Blackburn, R. W. Strange, J. Zubieta, *J. Am. Chem. Soc.* **1988**, *110*, 6769.
[32] L. S. Kau, D. J. Spira-Solomon, J. E. Penner-Hahn, K. O. Hodgson, E. I. Solomon, *J. Am. Chem. Soc.* **1987**, *109*, 6433.
[33] R. K. Hocking, E. I. Solomon (Eds.) *Ligand Field and Molecular Orbital Theories of Transition Metal X-ray Absorption Edge Transitions.* In: D. Mingos, P. Day, J. Dahl (eds) Molecular Electronic Structures of Transition Metal Complexes I. Structure and Bonding, vol 142. Springer, Berlin, Heidelberg., **2011**.
[34] R. Jurgeleit, B. Grimm-Lebsanft, B. M. Flöser, M. Teubner, S. Buchenau, L. Senft, J. Hoffmann, M. Naumova, C. Näther, I. Ivanović-Burmazović et al., *Angew. Chem. Int. Ed.* **2021**, *60*, 14154.
[35] a) J. L. DuBois, P. Mukherjee, A. M. Collier, J. M. Mayer, E. I. Solomon, B. Hedman, T. D. P. Stack, K. O. Hodgson, *J. Am. Chem. Soc.* **1997**, *119*, 8578; b) J. L. DuBois, P. Mukherjee, T. D. P. Stack, B. Hedman, E. I. Solomon, K. O. Hodgson, *J. Am. Chem. Soc.* **2000**, *122*, 5775.
[36] A. L. Ankudinov, B. Ravel, J. J. Rehr, S. D. Conradson, *Phys. Rev. B* **1998**, *58*, 7565.
[37] C. E. Elwell, N. L. Gagnon, B. D. Neisen, D. Dhar, A. D. Spaeth, G. M. Yee, W. B. Tolman, *Chem. Rev.* **2017**, *117*, 2059.
[38] a) H.-G. Korth, P. Mulder, *J. Org. Chem.* **2020**, *85*, 2560; b) H.-G. Korth, P. Mulder, *J. Org. Chem.* **2013**, *78*, 7674.
